# Proteomics Profiling Reveals Carbohydrate Metabolic Enzymes and 14-3-3 Proteins Play Important Roles for Starch Accumulation during Cassava Root Tuberization

**DOI:** 10.1038/srep19643

**Published:** 2016-01-21

**Authors:** Xuchu Wang, Lili Chang, Zheng Tong, Dongyang Wang, Qi Yin, Dan Wang, Xiang Jin, Qian Yang, Liming Wang, Yong Sun, Qixing Huang, Anping Guo, Ming Peng

**Affiliations:** 1 Key Laboratory of Biology and Genetic Resources for Tropical Crops, Institute of Tropical Biosciences and Biotechnology, Chinese Academy of Tropical Agricultural Sciences, Haikou, Hainan 571101, China; 2 College of Agriculture, Hainan University, Haikou, Hainan 570228, China

## Abstract

Cassava is one of the most important root crops as a reliable source of food and carbohydrates. Carbohydrate metabolism and starch accumulation in cassava storage root is a cascade process that includes large amounts of proteins and cofactors. Here, comparative proteomics were conducted in cassava root at nine developmental stages. A total of 154 identified proteins were found to be differentially expressed during starch accumulation and root tuberization. Many enzymes involved in starch and sucrose metabolism were significantly up-regulated, and functional classification of the differentially expressed proteins demonstrated that the majority were binding-related enzymes. Many proteins were took part in carbohydrate metabolism to produce energy. Among them, three 14-3-3 isoforms were induced to be clearly phosphorylated during storage root enlargement. Overexpression of a cassava 14-3-3 gene in *Arabidopsis thaliana* confirmed that the older leaves of these transgenic plants contained higher sugar and starch contents than the wild-type leaves. The 14-3-3 proteins and their binding enzymes may play important roles in carbohydrate metabolism and starch accumulation during cassava root tuberization. These results not only deepened our understanding of the tuberous root proteome, but also uncovered new insights into carbohydrate metabolism and starch accumulation during cassava root enlargement.

Cassava (*Manihot esculenta* Crantz) is one of the most important root crops, providing food for more than 600 million people worldwide^1^,^2^,^3^. Cassava is a drought-tolerant tropical crop that can grow well in poor soils. Its root can accumulate significant quantities of starch and persist in the soil for 1–2 years without decay^4^,^5^. These agronomic attributes allow cassava to provide a reliable source of food during famine periods in many developing countries^6^,^7^. Cassava tuberous roots contain more than 80% dry tuberous starch material, which can also produce ethanol for use as fuel^8^,^9^. Tuberization in cassava root primarily involves storage root formation, induction, development and resource storage^10^,^11^. The development of tuberous roots from primary roots *via* secondary growth and subsequent starch accumulation are determined by a balance between starch biosynthesis and degradation^12^.

Many gene expression studies performed in potatoes and sweet potatoes have revealed the regulatory mechanisms of carbohydrate metabolism and starch accumulation during tuberization^1^,^7^. Both endogenous factors and environmental factors can induce tuberous root formation. However, the mechanism underlying this process in cassava may differ substantially from that of potato, as the potato tuber originates from an underground stem and the cassava storage root is a part of the root system^7^. Several molecular markers related to cassava root yield and varieties exist in the cassava genome^13^,^14^,^15^. Studies using microarrays have identified many differentially expressed transcripts from both cassava leaves^2^,^16^ and storage roots^9^,^17^ at different developmental stages. After the roles of some of these starch accumulation-related genes in storage roots were clarified^18^,^19^, these genes were transformed into cassava to produce new genetically modified organisms^6^,^20^,^21^.

Recent studies in cassava proteomics have identified regulatory mechanisms involved in the development of somatic embryos^22^,^23^, leaves^10^,^24^, and roots^11^,^12^,^23^,^25^,^26^. Sheffield *et al.* presented the primary two-dimensional gel electrophoresis (2-DE) protein profiles of cassava fibrous and tuberous roots, identifying 237 proteins involved in these processes^12^. Baba *et al.* performed 2-DE gel analysis on samples during cassava somatic embryogenesis, digested most of the abundant spots, and positively identified 86 proteins, including several enzymes for energy metabolism^22^. Li *et al.* investigated the proteome of somatic embryos, plantlets and tuberous roots, finding high levels of tubulin expression level in tuberous roots^23^. Comparative proteomics of cassava leaves during the fibrous to tuberous root transition, suggesting that the possible metabolic switches in the leaf may trigger or regulate storage root initiation and growth in cassava^10^. Using isobaric tags for relative and absolute quantification (iTRAQ), Owit *et al.* investigated the changes in proteins during the physiological deterioration of cassava root and found that post-harvest physiological deterioration (PPD) in cassava root was an active process that included candidate enzymes with the potential to reduce deterioration^25^. Using label-free quantitative proteomics, these researchers further identified nearly 300 differentially expressed proteins during PPD. Finally, they verified that glutathione peroxidase can reduce PPD in cassava storage roots^11^.

Despite these studies, the proteomics changes in cassava tuberous roots during starch accumulation remain unclear. In the present study, the morphological changes in tuberous root at nine developmental stages were determined. The root proteins were separated by 2-DE and 2D-DIGE (two-dimensional differential in-gel electrophoresis). More than 1,500 spots were detected, and 154 differentially expressed proteins (DEPs) were identified by mass spectrometry (MS). Our results revealed that enzymes involved in the carbohydrate metabolic pathway and those with binding activities are important for sucrose metabolism. Functional analysis revealed that the cassava 14-3-3 gene may be important for starch accumulation in cassava tuberous roots.

## Results

### Morphological changes and starch accumulation during cassava root development

**Figure 1:**
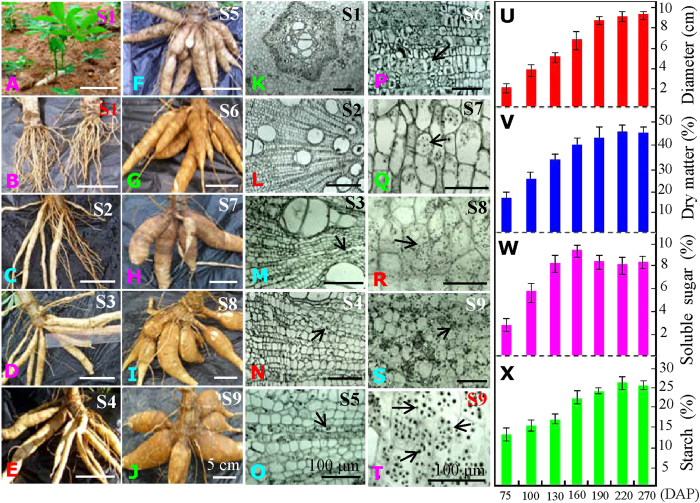
Morphological changes and starch accumulation patterns in cassava roots during tuberization. The seedlings (**A**) and roots (**B**) growing for 30 days after planting (DAP) were presented, respectively. Typical whole tuberous roots growing for 60 (**C**), 75 (**D**), 100 (**E**), 130 (**F**), 160 (**G**), 190 (**H**), 220 (**I**) and 270 (**J**) DAP were also presented. White bar, 5 cm. The middle parts of the main roots from the plants growing for 30 (**K**), 60 (**L**), 75 (**M**), 100 (**N**), 130 (**O**), 160 (**P**), 190 (**Q**), 220 (**R**) and 270 (**S**) DAP (S1–S9, respectively) were observed under a light microscope. Typical starch granules in the endoderm of 270 DAP roots are highlighted (**T**). Black bar, 100 μm. The arrows indicate the positions of the typical starch granules. The corresponding changes in root diameter (**U**), dry matter (**V**), soluble sugar (**W**) and total starch (**X**) in the main tuberous roots of the 75, 100, 130, 160, 190, 220 and 270 DAP plants are also shown.

Cassava tuberous roots are of significant importance for starch production; they are the consumed part of the cultivated plant. In our preliminary experiments, the cassava roots began to develop into tuberous roots in approximately 2 months, and the starch granules clearly appeared at approximately 3 months under a light microscope. The highest starch content was detected in the tuberous roots after planting for 7 months. Although the fresh weight and dry matter of the starchy roots steadily increased, the starch content slightly decreased with continued tuberous root development (data not shown). Therefore, we analyzed the growth patterns for the cassava roots at the following 9 stages (S1–S9) on the 30, 60, 75, 100, 130, 160, 190, 220 and 270 days after planting (DAP) (Fig. 1A–J). We found that the main roots began to develop into tuberous roots at S3 (Fig. 1D).

The typical starch granules were examined under a light microscope in the middle section of main roots at S3, and then they accumulated dramatically in the main roots during tuberization (Fig. 1M–T). With storage root development, both the tuberous root diameter (Fig. 1U) and dry matter (Fig. 1V) were significantly increased. The concentrations of soluble sugar (Fig. 1W) and starch (Fig. 1X) were significantly increased during tuberous root development, with the highest concentration of soluble sugar at S6 tuberous roots and the highest concentration of starch in tuberous roots at S8.

### Determination of differentially expressed proteins during cassava root tuberization by 2-DE and 2-D DIGE

Comparative proteomics of the cassava main roots were performed to analyze the regulation mechanisms involving in starch accumulation during tuberization at the protein level. Using our modified Borax/PVPP/Phenol (BPP) protocol^27^, the protein content in the fresh root tissues was less than 1 mg/g (Fig. S1A), which was much lower than the protein contents of other plants such as *Thellungiella halophila*^28^, *Sesuvium portulacastrum*^29^, and *Salicornia europaea*^27^. Although the protein profiles on 1-DE gels were similar, the patterns of several main protein bands, as marked with arrows, showed higher expression levels during root enlargement (Fig. S1B). These results illustrated the existence of some cassava root tuberization-specific proteins.

**Figure 2:**
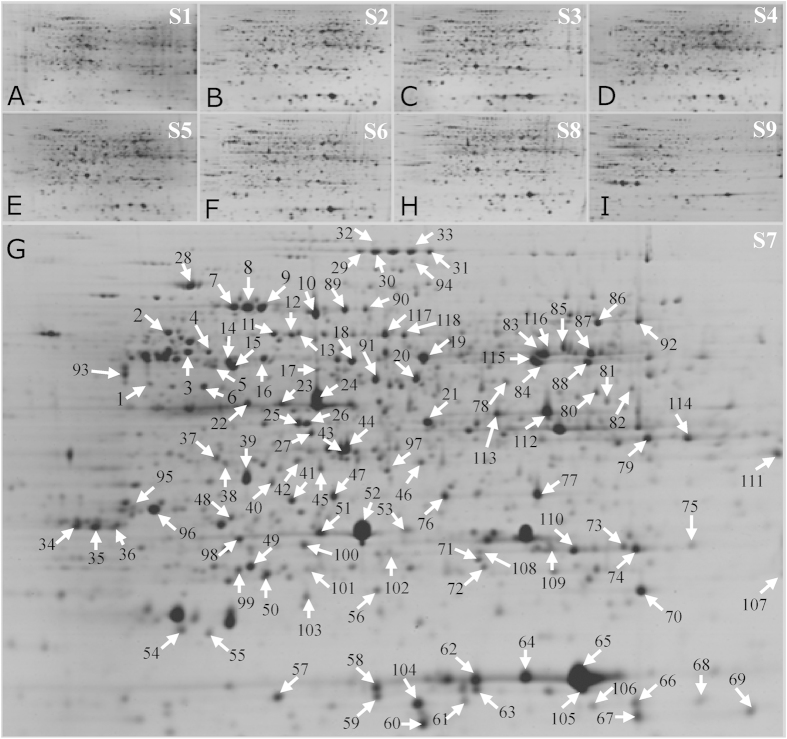
Common 2-DE gels of the tuberous root proteome at different developmental stages. Typical 2-DE images of the tuberous root proteins from the nine developmental stages (S1–S9) are presented in panels (**A–I**), respectively. The 118 differentially expressed proteins are highlighted in the typical 2-DE gel for S7 (**G**). Arrows indicate the differentially expressed protein spots that were positively identified by MALDI TOF MS/MS, and their identities are listed in Table S1 and Fig. S2.

**Figure 3:**
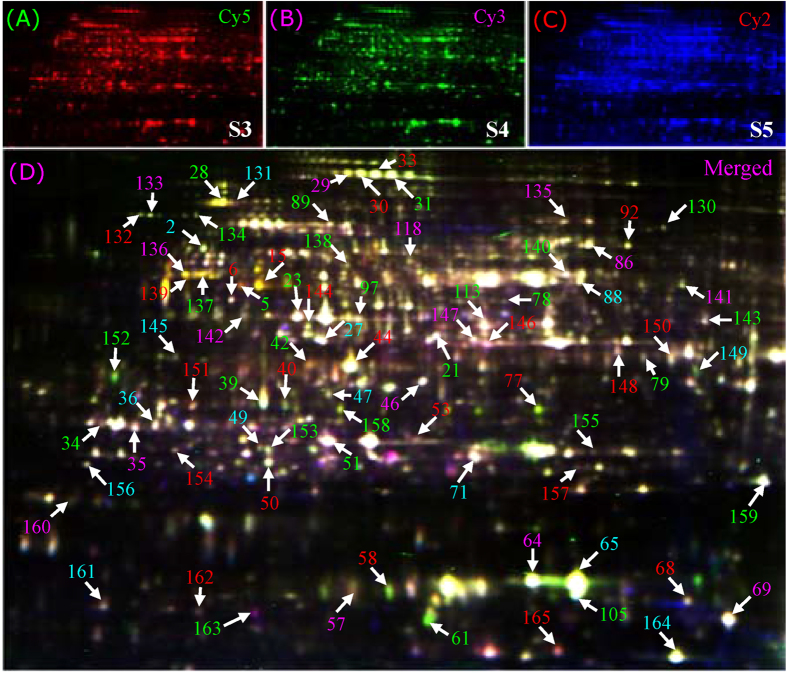
Typical 2-D DIGE images of the cassava root proteins at different developmental stages. Equal amounts (50 μg) of proteins obtained from cassava roots at S3, S4 and S5 (75, 100 and 130 DAP, respectively) were labeled with the Cy2, Cy3 and Cy5 fluorescence dyes, respectively. The three labeled and quenched samples were combined, and a total 150 μg of proteins were mixed and added to the rehydration buffer to perform 2-DE. The labeled proteins were visualized using parameters appropriate for the individual cyanine dyes. Red, Cy5 for S5 (**A**); Green, Cy3 for S4 (**B**); Blue, Cy2 for S3 (**C**). The combination image (**D**) of the three stages is highlighted. Most of the numbered protein spots correspond to the samples on the CBB-stained 2-DE gels. All of these protein spots were subjected to MS, and their identities are listed in detail in Table S2 and Fig. S3.

These proteins were further separated by 2-DE, and more than 1,300 protein spots were detected with good reproducibility on 2-DE gels (Fig. 2). As the starch granules were first observed in S3 (Fig. 1M), only the protein spots with good reproducibility, a fold-change of >2.0 in abundance and p < 0.01 for at least one stage compared to S3 were determined to be differentially expressed spots. Using this approach, 152 differentially expressed proteins were identified. We further performed 2-D DIGE to determine the differential protein expression profiles in the enlarged cassava roots during three (S3, S4, and S5) key developmental stages. The typical DIGE gels for proteins from the three stages (Fig. 3A–C) and the combined image (Fig. 3D) were presented to show the different proteins in the same image. The global protein profiles on the DIGE gels remained ~1,550 ± 60 protein spots, and a total of 115 protein spots demonstrated more than 1.5-fold changes (p < 0.01) during tuberous root development (Fig. 3).

### Identification and functional pathway analysis of DEPs during root development

**Figure 4:**
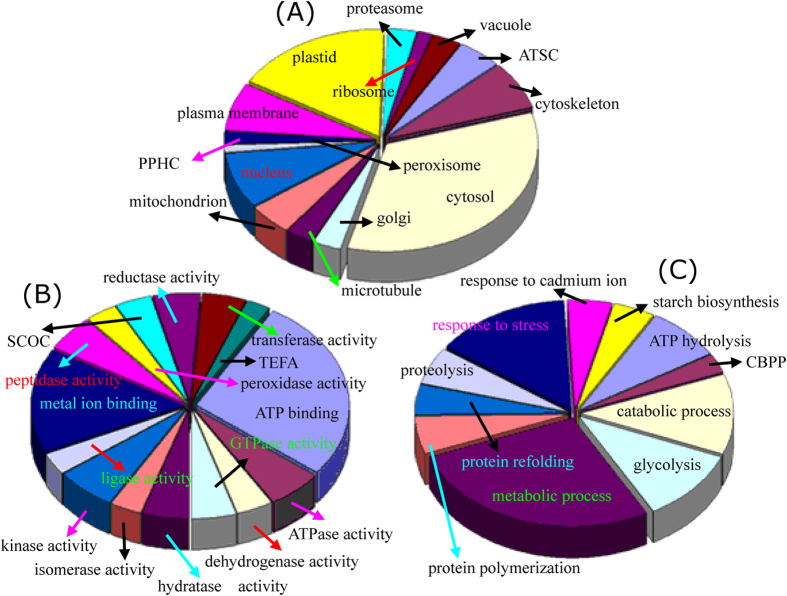
Classification and functional analysis of DEPs during cassava root development. Results of the GO classification and pathway analysis based on cellular component (**A**), molecular function (**B**), and biological process (**C**) are highlighted. Abbreviations: SCOC, structural constituent of cytoskeleton; TEFA, translation elongation/initiation factor activity; CBPP, carbohydrate phosphorylation; PPHC, phosphopyruvate hydratase complex; ATSC, ATP synthase complex.

Of the 152 differentially expressed protein spots in 2-DE gels, 118 were positively identified by MS (Fig. 2; Tables S1; Fig. S2). Whereas 80 out of 115 DEPs on the 2-D DIGE gels were identified, 44 of the same proteins were identified on the 2-DE and DIGE gels, and 36 proteins (spots 130–165) were only obtained from the DIGE gels (Fig. 3; Fig. S3; Table S2). GO analysis was further performed to obtain deeper insights into the cellular components (Fig. 4A), molecular function (Fig. 4B), and biological processes (Fig. 4C) of these proteins. The cellular components of the 154 identified proteins were further determined by Blast2GO, while some of the homologous proteins were obtained by searching the TAIR database. Their corresponding AGI (*Arabidopsis* Gene Identifier) and TAIR location information were presented in the supplementary Tables S1 and S2. The results revealed that the largest number of proteins (66 proteins) were localized to the cytoplasm, though many proteins were in the plastid (33 proteins), nucleus (16 proteins), cytoskeleton (9 proteins), and plasma membrane (9 proteins). There were 9 mitochondrial proteins localized to the proton-transporting ATP synthase complex (GO: 0045261), indicating that large amounts of ATP were consumed during starch accumulation and cassava root enlargement. Moreover, the remaining proteins were localized to vacuoles (6 proteins), the proteasome (6 proteins), microtubules (6 proteins), etc. (Fig. 4A).

At the molecular function level, the majority part including 66 proteins were over-represented in binding activity. Of these proteins, 42 participated in ATP binding, and 24 proteins were involved in metal ion binding (Fig. 4B). In addition, 6 of these metal binding enzymes were specific for magnesium ion binding, 4 enzymes were involved in zinc ion binding, one was involved in copper ion binding, and one was specific for ferric iron binding (Tables S1 and S2). Furthermore, 10 proteins showed kinase activity, including glucan phosphorylase and a hypothetical protein with possible phosphorylase activity. Some enzymes with ATPase activity, GTPase activity, peptidase activity, reductase activity and transferase activity were also identified (Fig. 4B; Tables S1 and S2). The biological process analysis revealed that the largest group of proteins contained 30 proteins involved in metabolic processes, including many enzymes (eg., malate dehydrogenase, spots 79 and 114, and isocitrate dehydrogenase, spot 81) that participate in cellular carbohydrate metabolic processes. The second largest group contained 16 proteins involved in the stress response pathway. Some other important biological processes, such as catabolism process, glycolysis, and ATP hydrolysis, were also represented. Notably, 9 protein species were involved in the starch biosynthetic process, including 3 unique proteins: ADP-glucose pyrophosphorylase (spot 20), glucan phosphorylase (GLUP, spots 29–33), and fructokinase (spots 37, 38 and 151) (Fig. 4C; Tables S1 and S2). These results illustrated that the enzymes involved in metabolism, catabolism and starch biosynthesis were induced during starch accumulation in the cassava storage roots.

### Clustering and comparison of the changes in the gene and protein expression patterns

**Figure 5:**
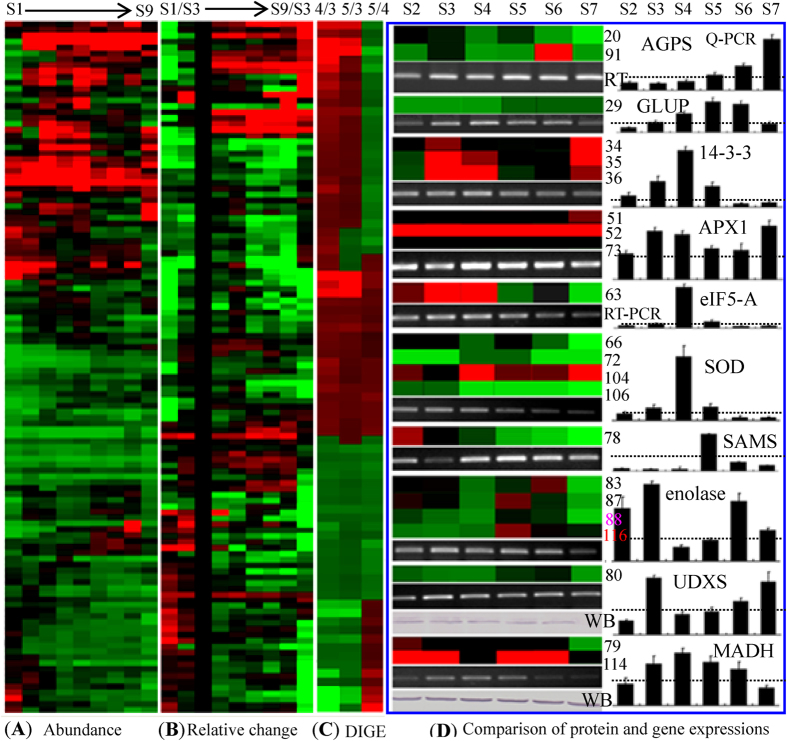
Clustering of the differentially expressed proteins and a comparison of the expression patterns for ten proteins and their transcripts. The hierarchical clustering of the abundance volume of the protein spots (**A**) and their relative volume ratios (**B**) at S3 on 2-DE gels are shown. The average changed abundance ratios of the identified protein spots on 2-D DIGE gels are also presented (**C**). The high- or low-abundance protein spots or the induced- or reduced-values for the relative changed ratios were indicated in red or green, respectively. The details for hierarchical clustering of the changed patterns of the differential protein spots on 2-DE and 2-D DIGE are presented in Table S2 and Table S3, respectively. Then, 10 DEPs with sharp changes were selected to determine the correlations between their gene and protein expression levels using RT-PCR and qRT-PCR. The spot numbers for a specific protein are given on the right side of the heat plot next to the same protein in the heat plot. For each treatment, the expression of each gene is presented as a ratio between the gene value and the corresponding 18SRNA value. The gray dot line in each rectangle graph represents a 1.0 ratio value. The error bars indicate the SD of three independent PCR reactions for three biological replicates. For UDXS and MADH, antibodies were used to determine the protein expression, revealing that the two proteins had similar expression patterns to those on the 2-DE gels (**D**). Abbreviations: AGPS, ADP-glucose pyrophosphorylase small subunit; GLUP, glucan phosphorylase; APX1, ascorbate peroxidase-1; initiation factor eIF5-A; SOD, superoxide dismutase; SAMS, S-adenosylmethionine synthetase; UDXS, UDP-D-apiose/UDP-D-xylose synthase; MADH, malate dehydrogenase; WB, Western blotting.

Hierarchical clustering of the 118 identified proteins on 2-DE gels was performed based on their expression abundance (Fig. 5A). The relatively expression patterns of each stage were also compared to those at S3 (Fig. 5B). Five main clusters were obtained using the abundance-based clustering approach. The first cluster contained 18 proteins with low abundance at S1 and S9, while these proteins were dramatically up-regulated during the developmental stages from S2–S8. This cluster included heat shock protein (HSP, spots 60, 65 and 105), initiation factor (spots 58 and 63), tubulin (spots 14 and 15), ATP synthase (spot 19), and others. The second cluster contained 26 proteins with very high abundance from S1 to S8 but somewhat decreased abundance in S9. The absolute abundance of the proteins in these two clusters was very high, and their abundance was dramatically increased from S3–S6. The third cluster contained the largest group of proteins with low abundance, and most of these were down-regulated during tuberous root development. The fourth cluster was the smallest group and consisted of 12 proteins that showed the highest abundance in the late developmental stages of tuberization. The fifth cluster included 27 proteins with high abundance during the early developmental stages (S1 and S2) and sharply decreased abundance during root enlargement (Fig. 5A; Table S1).

Clustering of the relative expression patterns of these proteins resulted in four main clusters. Cluster I contained 20 proteins with higher relative abundance compared to those in S3 (Fig. 5B). These proteins were initiation factor (spots 58 and 59), thiazole synthase (spots 76 and 77), glucan phosphorylase (GLUP, spots 29 and 30), phosphate adenylyltransferase (spot 91), and a few others. Among these proteins, several enzymes, including glucose pyrophosphorylase, GLUP and amylase, are known to be crucial factors for starch biosynthesis. Cluster II contained 22 proteins with high abundance in S3. The proteins in cluster III demonstrated similar changes in their ratios as those in cluster II, but these proteins had a high ratio in later stages. Proteins with high relative ratios in the early developmental stages (S1–S3) were included in cluster four (Fig. 5B; Table S1). Compared to the relative ratios obtained from the common 2-DE gels, the protein spots on 2-D DIGE showed similar clustering patterns. The changes in the patterns of some important proteins, such as the 14-3-3 protein species (spots 34-36) and GLUP (spot 29), also demonstrated a positive correlation with the starch accumulation during root enlargement (Fig. 5C; Table S2).

In this study phase, 10 typical proteins exhibited significant changes during tuberization and were thus deemed to play important roles in starch biosynthesis in cassava roots. The gene and protein expression patterns were compared for these selected proteins (Fig. 5D). In the six developmental stages (S2, S3, S5, S6, S8 and S9), the protein and gene expression levels were positively correlated. The most abundant protein spot for ascorbate peroxidase-1 (APX1) was observed in S9; its mRNA expression level increased with tuberous root development, reaching its highest expression level in S5. The 14-3-3 protein showed the highest abundance in S3, but the 14-3-3 gene was highly expressed in S4. Although the protein abundance was very high in S9, the expression of the 14-3-3 gene decreased to a very low level in that stage. The protein and gene expression levels for ADP-glucose pyrophosphorylase small subunit (AGPS), GLUP, UDP-D-xylose synthase (UDXS), and MADH, which are involved in starch and sucrose metabolism, were very high during starch accumulation (Fig. S4). Western blotting confirmed that UDXS and MADH were most abundant in S3, and their genes were also highly expressed in the same stage (Fig. 5D).

### Comparison of 14-3-3 protein expression in cassava tuberous roots and transgenic *Arabidopsis* plants

**Figure 6:**
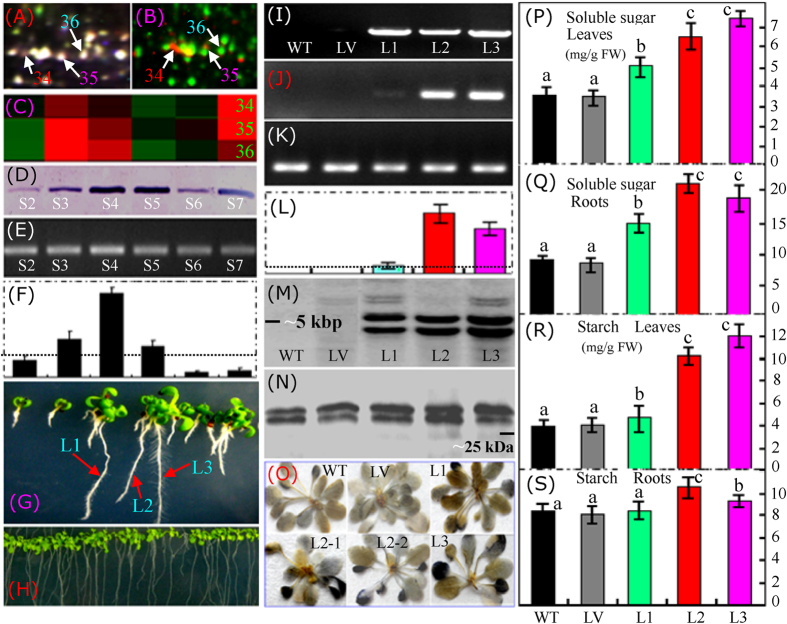
Determination of the induced changes of the 14-3-3 protein in both cassava tuberous roots and transgenic *Arabidopsis* plants. The typical area of the 14-3-3 isoforms on the 2-D DIGE gel (**A**) and the merged Pro-Q Diamond- and SYPRO Ruby-stained gels (**B**) are highlighted. Abundance clusters of the three identified 14-3-3 protein spots (**C**) at different tuberous root developmental stages (S2-S7) are also presented. The total protein abundance detected by Western blotting (**D**) and the general gene expression detected by RT-PCR (**E**) and qRT-PCR (**F**) demonstrated similar changes in the expression patterns during tuberous root development. Three lines (L1-3) of hygromycin-resistant (**G**) transgenic *Arabidopsis* plants were selected to produce T3 generation seeds. In standard MS medium (**H**), the T3 seedlings showed no difference compared to the wild type (WT) or the line overexpressing the P-super 1300^+^ vector (LV). PCR analysis verified that the cassava 14-3-3 gene was subcloned into the genomes of the three lines, but not into the WT or LV plants (**I**). RT-PCR (**J**) and qRT-PCR (**L**) revealed that the target gene had different expression levels. The *Arabidopsis* actin gene was used as a reference (**K**). The fragment number of T-DNA insertions was determined by Southern blotting (**M**). The protein abundances in the different plant lines were determined by Western blotting using a polyclonal antibody against cassava 14-3-3 protein (**N**). Starch accumulation in the leaves of different plants was visualized using an iodine solution (**O**). The soluble sugar (**P,Q**) and starch (**R,S**) concentrations in both the leaves (**P,R**) and roots (**Q,S**) of the different *Arabidopsis* lines (WT, LV, and L1-3) were also determined. The gray dot line in each qRT-PCR rectangle graph represents a 1.0 ratio value. In figures **P–S**, the columns labeled with a and b or b and c indicate significant difference (p < 0.05), the columns labeled with a and c stand for more significant difference (p < 0.01) between these treatments.

On both the common 2-DE (Fig. 2G) and 2-D DIGE (Fig. 3D; Fig. 6A) gels, at least three differential spots (spots 34–36) were identified as the 14-3-3 protein. We further detected the phosphorylated proteins in cassava tuberous roots. In the merged image of the gels stained with Pro-Q Diamond and SYPRO Ruby containing proteins extracted from the S3 cassava tuberous roots, specific phosphorylated protein spots were clearly visualized in red (Fig. 6B), and at least two 14-3-3 isoforms (spots 34 and 35) were detected (Fig. 6B), indicating that the 14-3-3 proteins were phosphorylated. During tuberous root development, the three 14-3-3 isoforms showed similar changes in their expression, and the highest protein abundance was observed in stages 3 and 4, in which the starch began to accumulate in the tuberous storage cells (Fig. 1). During the later tuberous root enlargement, the abundance of the 14-3-3 protein decreased to some extent at S5 and S6 but increased to a higher level with continued tuberous storage root development (Fig. 6C,D). Similar 14-3-3 gene and protein expression patterns were observed, and the highest gene expression level was detected in the S4 tuberous roots (Fig. 6E,F).

To determine the function of the cassava 14-3-3 protein, this gene was overexpressed in the model plant *Arabidopsis thaliana*. Three typical transgenic *Arabidopsis* lines harboring *35S::14-3-3* were selected to produce the T3 generation seeds. PCR analysis of the genomic DNA confirmed that *Me14-3-3* was subcloned into the three lines, but this gene was not detected in the wild-type (WT) plant or the line overexpressing the P-super 1300^+^ vector (LV). Both RT-PCR and qRT-PCR revealed that *Me14-3-3* demonstrated very different expression patterns among the different transgenic lines. The expression of *Me14-3-3* was low in the L1 plants, but very high in the L2 and L3 plants (Fig. 6G–L). A Southern blot of the genomic DNA digests using the *Me14-3-3* coding region as a probe showed two main hybridization bands in the three transgenic lines (Fig. 6M), indicating that two fragments of the T-DNA insertions were integrated into *Arabidopsis* genome in these transgenic plants. As expected, there was no hybridization bands detected in the WT and LV plants, since the target gene was not inserted into their genomes. Western blotting using a polyclonal antibody against the cassava 14-3-3 protein showed that two main hybridization bands were detected in both the three overexpression lines and the controls (WT and LV) (Fig. 6N).

We further performed Blast analysis of the cassava 14-3-3 protein to the Arabidopsis proteins, and found that there are two kinds of 14-3-3 proteins in Arabidopsis. Among them, most are about 30 kDa, and several other members are about 28 kDa. The two hybridization bands in these plants may include both the overexpressed cassava 14-3-3 protein (about 29.84 kDa) and the other14-3-3 protein members in Arabidopsis. The abundance of the upper band was increased obviously in these three overexpression lines than that in the WT and LV, and the abundance of the lower band was not changed significantly among the detected plants (Fig. 6N). These results indicated that the increased abundance of the cassava 14-3-3 protein could be observed in the *Me14-3-3-*overexpressing plants.

The starch in the leaves of different plants was visualized using an iodine solution, which showed increased starch concentrations in the transgenic plants. Notably, much more starch was accumulated primarily in the older leaves of these transgenic plants (Fig. 6O), indicating that the cassava 14-3-3 protein could enhance starch storage in the mature and older plant tissues. Quantitative measurements of the starch in the leaves (Fig. 6R) and roots (Fig. 6S) of plants grown in MS medium revealed an approximately 2-fold increase in the total starch content of the leaves from trangenic plants. The highest starch content (~12 mg/g FW) was detected in the L3 plants, followed by the L2 (~10 mg/g FW) and L1 plants. Although significantly increased starch content was detected in the roots of the L2 and L3 plants, the increased was not large and the roots contained similar starch content compared to the WT and LV plants, even in the specific line (L1) with a low *Me14-3-3* expression (Fig. 6R). Correspondingly, the total soluble sugar content was sharply increased in the leaves and roots of the three transgenic plants. The highest soluble sugar content (~7.5 mg/g FW) was detected in the leaves of the L3 plants (Fig. 6P), which was increased by approximately 2.5-fold compared to the WT and LV. In the roots, a much higher concentration of soluble sugar was observed, and the highest value (~22 mg/g FW) was detected in the L2 plants, which was approximately 3-fold higher than the concentration of soluble sugar detected in the WT and LV plants (Fig. 6Q). These results confirmed that the accumulation of starch and soluble sugar was positively correlated with the expression level of *Me14-3-3* in both the leaves and roots of the transgenic plants.

**Figure 7:**
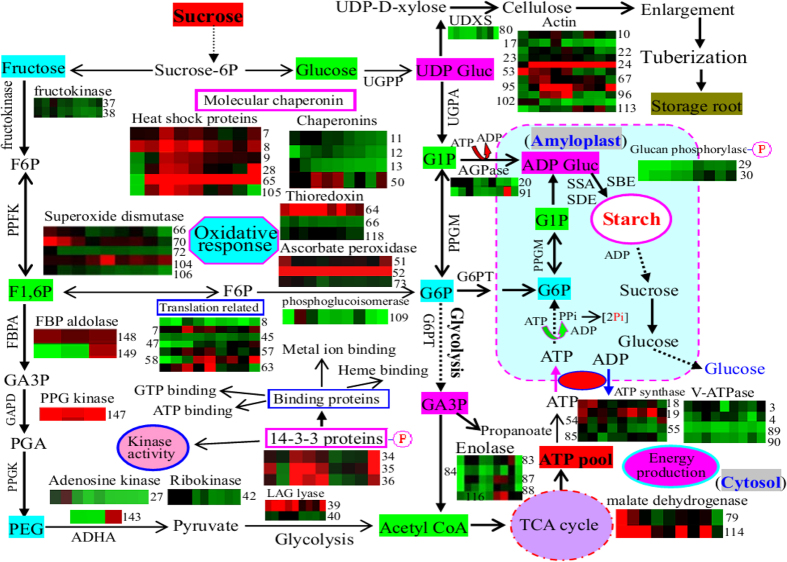
Schematic representation of the enzymes and major biological pathways involved in carbohydrate metabolism and starch accumulation during cassava storage root enlargement. The graphs represent the expression profile of individual proteins. The numbers on the right or left side in each colored rectangle indicate the protein spots. The abbreviations: Sucrose-6P, sucrose 6 phosphate; F6P, fructose 6 phosphate; F1,6P, fructose 1,6 bisphosphate; PEP, phosphoenolpyruvate; TCA, tricarboxylic acid; G1P, glucose 1 phosphate; G6P, glucose 6-phosphate; GA3P, glycerate-3-phosphate; PGA, phosphoglyceric acid; UDXS, UDP-D-xylose synthase; UDP Gluc, UDP Glucose; ADP Gluc, ADP glucose; Suc synthase, sucrose synthase; UGPP, UDP-Gluc pyrophosphorylase; PPFK, pyrophosphate dependent phosphofructokinase; PPGA, 6-phosphogluconolactonase /phosphoglucoisomerase; PPGM, phosphoglucomutase; UGPA, UDP-Gluc pyrophosphorylase; G6PT, glucose 6 phosphate/phosphate translocator; AGPase, ADP-glucose pyrophosphorylase; SSA, starch synthase; SBE, starch-branching enzyme; SDE, starch-debranching enzyme; GPPA, glycogen/starch/glucan phosphorylase; Pi, inorganic phosphate; PPi, inorganic pyrophosphatase; Hsp, heat shock protein; FBPA, fructose-bisphosphate aldolase; GAPD, glyceraldehyde-3-phosphate dehydrogenase; PPGK, phosphoglycerate kinase; ADHA, alcohol dehydrogenase; LAG lyase, lactoylglutathione lyase; ROS, reactive oxygen species.

Given this proteomics of cassava tuberous roots and the results of recently published results^5^,^17^,^31^,^32^, we summarized the potential positions and main functions of the identified cassava root proteins, and then proposed a possible schematic regulation of the DEPs involved in starch accumulation and enlargement in the cassava storage roots (Fig. 7). These results revealed that many enzymes involved in carbohydrate-related metabolic processes, some binding proteins, and some phosphorylated 14-3-3 protein isoforms may play crucial roles in controlling the accumulation of starch during cassava tuberous root development.

## Discussion

### In-depth proteomics revealed that the enzymes involved in energy metabolism and binding activity may play important roles in cassava storage roots

Starch accumulation requires coordinated interactions between environmental, biochemical, and genetic factors. Many biological processes, including carbon partitioning, signal transduction and meristem determination, are involved in tuberization. Compared to gene transcription, the protein-mediated post-transcriptional and post-translational processes lead to the final cell products. Thus, proteomics allows for the global analysis of gene products in the cellular physiological state^30^. In this comparative proteomics study, we focused on the 62 proteins that were sharply up-regulated during starch accumulation in tuberous roots, finding that these proteins were primarily involved in glycolysis, metabolic processes, energy production and starch biosynthesis (Figs 4 and 5). In the developing tuberous roots, carbohydrate metabolism and glycolysis are important platforms for carbon assimilation and energy production^30^. Our results verified that enolase (spot 88), a key enzyme in glycolysis, was significantly induced upon starch accumulation (Fig. 7), which has also been observed in the storage roots of potato^33^, sweet potato^34^ and cassava^17^,^23^,^35^. Enolase plays important roles in carbohydrate metabolism as well as in the regulation of stress-inducible gene expression, in which it acts as a transcriptional regulator^36^. Notably, during tuberous root development, many cytosolic ATPases were induced while almost all detected vacuolar-type ATPases were inhibited (Fig. 7; Tables S1 and S2). This result indicated that more cytosolic ATPases were required to provide energy for starch accumulation in the cassava storage roots.

Among the 62 induced proteins, more than half (35 proteins) had strong binding activities, such as ATP binding, metal ion binding, GTP binding, ribosome binding, phosphate binding, and heme binding (Tables S1 and S2). Most ATP binding proteins were HSPs and groes chaperonins. Our results revealed that most of the large HSPs maintained high expression levels during tuberous root development, but some small HSPs tended to decline in abundance during the late developmental stages. These changes in expression patterns were similar to those observed during PPD in cassava roots^25^. Actin and tubulin are two important cytoskeletal proteins with ATP binding and GTP binding abilities, respectively. The accumulation of tubulin was high in the tuberous roots, but very low in the cassava somatic embryos^23^. Our results (Fig. 7) verified that the expression levels of several actin and tubulin proteins were positively correlated with starch accumulation, which ultimately improved the tuberous root enlargement.

Several metal ion binding proteins involved in the stress response, including enolase, ubiquinone oxidoreductase, thiazole synthase, and SOD, showed a high abundance during tuberous root enlargement. The generation of reactive oxygen species (ROS) is an inevitable process in all aerobic organisms, particularly when they encounter pathological and physiological stress conditions^30^. Our proteomic data revealed that various antioxidant proteins, including oxidoreductase, SOD, ascorbate peroxidase, peroxiredoxin and aldehyde dehydrogenase, were increased during cassava tuberous root development. Similar results have been observed in potato tubers^30^. These enzymes are involved in minimizing the deleterious effects of ROS during tuberous root development^30^ and are thought to play key roles in the protection against PPD in cassava roots^11^,^25^,^37^. Notably, two thiazole biosynthetic enzymes (spots 76 and 77) were sharply induced. Thiazole synthase has dual functions in thiamine biosynthesis and DNA damage tolerance, and can generate the thiazole portion of thiamine to cope with ROS. In a previous proteomics study, thiazole synthase was also identified in cassava storage roots^12^. A positive correlation between late embryogenesis abundant (LEA) protein (spot 152) and the onset of starch accumulation were observed in cassava (Fig. 7; Table S2), which was similar to the results obtained for the cold-induced development of the potato tuber^30^. Three isoforms (spots 58, 59 and 63) of eukaryotic translation initiation factor 5A (eIF5A) were induced, indicating that profound cellular reorganization and gene expression occurred during cassava tuberous root enlargement. These initiation factors were also identified as the 14-3-3 binding proteins in the proteomics analyses^38^,^39^.

### Proteomic analysis revealed that the enzymes involved in starch and sucrose metabolism were overexpressed during starch accumulation and tuberous root enlargement

The cassava starchy root is an important source of carbohydrates because it accumulates a large amount of starch in its tuberous roots^5^. Uncovering the expression patterns of proteins during the development of the storage root can provide important information on storage root formation and starch accumulation as well as unlock new traits for improving starch yield^7^. Our proteomic data revealed that many enzymes involved in starch and sucrose metabolism were significantly induced during cassava tuberous root development. Among these induced enzymes, AGPase is the main component in the starch biosynthesis pathway in nearly all plant species^40^. Because AGPase can convert ADP-glucose to glucose-1-phosphate, this enzyme is involved in sucrose and starch biosynthesis^10^. During storage root formation, AGPase is also up-regulated in cassava leaves^10^. It is also a 14-3-3 binding protein^41^, and the phosphorylation-induced regulation of AGPase influences both the levels and composition of the starch^42^. AGPase is induced in actively developing potato tubers, and its activation reflects the stimulation of starch synthesis and decreases levels of glycolytic intermediates. This likely by linking starch synthesis to the sucrose supply, indicating the importance of AGPase in starch accumulation^30^. Overexpression of the AGPase gene has altered the carbohydrate source-sink relationship, leading to increased starch accumulation and biomass in cassava storage roots^6^.

Starch phosphorylase plays an important role in the formation of primer molecules involved in starch biosynthesis, including amylose, amylopectin and a maltodextrin with at least three glucose residues^43^. Plants possess two types of glucan phosphorylases with different cellular location^43^. Both of these enzymes catalyze the transfer of glucosyl units from glucopyranosyl phosphate (Glc-1-P) to the non-reducing end of 1,4-linked glucan chains^44^. Although the traditional view indicates that GLUP acts in degradation rather than biosynthesis in starch metabolism, recent studies have verified that GLUP activity coincides with starch accumulation in developing maize and wheat endosperms^44^. This was also verified by our proteomics data, which showed that GLUP is positively correlated with starch accumulation in cassava roots (Fig. 7; Tables S1 and S2). It has been reported that granule-associated starch phosphorylase can be phosphorylated^43^. The regulation of the glucan phosphorylase activity is considered to be involved in phosphorylation, leading to the formation of a starch synthesizing protein complex with 14-3-3 proteins to complete the regulatory transition^42^,^45^. Dimerized 14-3-3 proteins can bind to phosphorylated target proteins, serving as a scaffold protein to hold the plastidic enzymes of starch synthesis together in a phosphorylation-dependent complex^41^.

Fructokinase, GPPA (glycogen/starch/glucan phosphorylase), and glucan synthase, which are crucial to glycolysis, facilitate the conversion of sucrose or sucrose breakdown products (i.e., glucose and fructose) into cytosolic hexose^32^. During carbohydrate metabolism, cytosolic GPPA can incorporate glucose from glucose-1-P into the cytosolic heteroglycan^46^. The increased induction of fructokinase and phosphorylase attributed to the increased synthesis of sucrose during tuber development^30^. Another study showed that the GPPA gene is obviously up-regulated in storage roots, indicating that GPPA is a key enzyme required for starch accumulation in the cassava tuberous root^17^. The activities of glucan synthase (also named cellulose synthase) and GPPA can account for the rates of starch accumulation occurring in the leaves^32^. In cassava roots, glucan synthase, a cell wall-modifying protein, has been shown to be up-regulated during early and late PPD^25^. Our results revealed that the abundance of glucan synthase and xylose synthase were sharply increased in the enlarged storage roots (Fig. 7), suggesting that the cell wall undergoes significant remodeling during tuberization.

### The 14-3-3 protein isoforms and their binding proteins are important for starch accumulation during cassava tuberization

The 14-3-3 proteins are major regulators of the plant primary metabolism and other cellular processes^47^. In *Arabidopsis*, at least 12 expressed members and 15 genes of the 14-3-3 family were examined^42^,^47^,^48^. In the present study, at least three 14-3-3 protein isoforms (spots 34-36) were induced during cassava root enlargement (Fig. 6; Tables S1 and S2). The 14-3-3 family of proteins includes multiple isoforms that can interact with various cellular phosphoproteins through a calcium-dependent protein kinase^49^, regulating several cell signaling cascades that play crucial roles in energy metabolism and biosynthetic reaction^48^,^50^. This regulation is conducted by affecting direct protein-protein interactions, which are mediated by the phosphorylation of a serine or threonine residue in specific sequence motifs on the target protein^49^,^51^,^52^. The 14-3-3 proteins are associated with the ATP synthases in a phosphorylation-dependent manner through direct interaction with the F1 subunit of ATPase^53^,^54^. Our results demonstrated that at least two 14-3-3 isoforms (spots 34 and 35) were obviously phosphorylated during tuberization (Fig. 6B), indicating the important role of the phosphorylation of 14-3-3 isoforms during starch accumulation. A number of potential targets for 14-3-3 proteins have been characterized in animals and plants. These include metabolic targets and regulators (e.g., several protein kinases, phosphatase, and phospholipase)^52^,^54^. The recent proteomics of 14-3-3 binding proteins using a specific peptide elution from 14-3-3 affinity columns resulted in the identification of many proteins with roles in signal transduction, carbohydrate metabolism, and protein folding^38^,^39^,^55^. In *Arabidopsis*, the starch synthase III family is a potential target for the 14-3-3 protein^45^. By preventing interaction with 14-3-3 protein, a protein kinase can obviously inhibit the plasma membrane H^+^-ATPase activity in *Arabidopsis*^56^.

In this study, several potential 14-3-3 binding proteins were identified. Among these, lactoylglutathione lyase (spots 39 and 40), MADH (spot 79) and glutathione S-transferase (spot 107) were predicted to participate in carbohydrate metabolism in the cassava storage tuberous roots. Lactoylglutathione lyase is a glyoxalase I homolog responsible for the first step in methylglyoxal catabolism in *Arabidopsis*, this enzyme has also been identified *via* proteomic analysis during secondary somatic embryogenesis in cassava^22^ and potato tubers^33^. Malate dehydrogenase (MADH), a key component of the TCA cycle, is a direct target of the 14-3-3 protein. It is highly likely that interactions between the 14-3-3 protein, MADH, and isocitrate dehydrogenase (spot 81) are crucial factors for controlling the activities of key enzymes in the TCA cycle^57^. The mitochondrial MADH has been mechanistically linked to an altered redox status of AGPase in the plastid, and strong correlations between the changes in the cellular malate concentration, NADP reduction state, and starch synthesis have been observed in tomato fruit^31^. MADH is highly induced during the development of potato tubers^30^,^33^ and cassava leaves^10^. In cassava storage roots, a total of 4 MADH isoforms have been identified in a proteomic analysis^12^, which is in consistent with our observations that 2 MADH isoforms (spots 79 and 114) changed in expression levels during tuberization (Fig. 5; Tables S1 and S2). Glutathione S-transferase (GST), which catalyzes the formation of glutathione from L-Glu, has also been identified as a target of 14-3-3 binding protein by affinity purification and tandem mass spectrometry in barley seedlings^38^ and transgenic mice^39^. In cassava storage roots, 5 GST isoforms have been identified^12^. Our results verified that GST (spot 107) was sharply induced during root development (Fig. 5; Tables S1 and S2). It has been reported that the gene expression^58^, protein abundance and enzyme activity^11^ of GST are obviously increased during PPD in cassava storage roots, indicating a key role for GST in the ascorbate/glutathione cycle^11^.

Functional analyses in rice have shown that 14-3-3 proteins are involved in plant defense and responses to stresses^59^. In lily pollen grains, 14-3-3 proteins can regulate the turgor pressure by modulating the activity of the plasma membrane H^+^-ATPase^60^. In rice seedlings, a 14-3-3 protein was shown to be activated by calcium-dependent protein kinase 1 under sugar starvation and gibberellin treatment, thereby conferring drought tolerance^61^. In *Arabidopsis*, two 14-3-3 members have been shown to be important regulators of salt tolerance; these proteins can inhibit the salt overly sensitivity pathway by activating binding kinase activity in planta^62^.

A general pattern of reduced 14-3-3 levels has been shown to lead to increased starch, protein and lipid contents, although these results have not always been consistent depending on the experimental methodologies used^63^. In potato tubers, the overexpression of 14-3-3 proteins disturbed the starch content and amino acid composition, and an inverse correlation between antisense expression of these proteins and senescence was observed. Moreover, individual plant 14-3-3 proteins demonstrate differing affinities for specific substrates^50^. As components of the photosensory system, the 14-3-3 proteins of *Arabidopsis* regulate root growth and chloroplast development^54^. A previous study showed that the levels of starch, sucrose, fructose, malate and amino acids were significantly decreased in plants overexpressing 14-3-3, but the enzyme activities of sucrose-phosphate synthase and fructose-bisphosphate aldolase were obviously improved in the wild-type plants^57^. However, in the present study, the overexpression of a cassava 14-3-3 protein in *Arabidopsis* significantly improved the soluble sugar and starch contents in both the roots and leaves, with a particularly noticeable effect in older leaves (Fig. 6O–S). These results revealed that different 14-3-3 isoforms from different plants may have different functions. The detailed regulation and expression patterns for the different 14-3-3 isoforms in the cassava storage roots should be determined in the future.

In conclusion, we compared the starch accumulation patterns during 9 stages of cassava root development and used 2-DE and MS to identify 154 DEPs during tuberization. Functional analysis demonstrated that the majority of these proteins were binding proteins, and biological process analysis revealed that the largest group was involved in carbohydrate metabolism to produce energy. Many enzymes involved in starch and sucrose metabolism were overexpressed during root enlargement. Among the DEPs, at least three 14-3-3 isoforms were induced during tuberization, and they were obviously phosphorylated during starch accumulation in cassava storage roots. The overexpression of the cassava 14-3-3 gene in *Arabidopsis* confirmed that the cassava 14-3-3 protein isoforms and their binding proteins may play crucial roles in carbohydrate metabolism and starch accumulation during cassava root tuberization.

## Materials and Methods

### Plant growth conditions and root collection

Cassava cultivar SC8 was grown under the field-grown condition in our experimental farm in Hainan Province, China. The stem cuttings for vegetative propagation were obtained from identical regions of stems with similar sizes and ages. These cuttings were planted in a field with ten rows. Each row contained ten plants that were used as different biological replicates. Different plants in each row were used as materials for technical replicates. The typical main roots from plants at 30, 60, 75, 100, 130, 160, 190, 220 and 270 (the nine stages, termed as S1–S9) days after planting (DAP) were collected from each row, rinsed with tapped water, blotted dry on filter paper, dissected into approximately 5-mm-thick slices, immediately frozen in liquid nitrogen and subsequently stored at −80 °C for further analysis.

### Morphological analysis and determination of root diameter and dry matter

A 0.5-cm-long middle section of the typical main root was used to perform the morphological analysis as previously described^28^. The root diameter was measured with a ruler directly after harvesting. The fresh weights of the cassava roots were determined immediately after harvesting. The dry matter content was determined after 72 h of drying in an oven at 60 °C. Five biological replicates including 3 technical replicates were performed and the results are shown as the means ± SD.

### Protein extraction, 2-DE and 2-D DIGE analysis

The total proteins from the middle part of the cassava roots were extracted by a modified phenol protocol^27^. Protein concentration was determined using the Bradford assay. BSA was used as standard. The common 2-DE assay was performed as previously described^28^,^29^. In brief, 1000 μg of proteins were loaded onto an IPG strip holder with 24 cm, pH 4–7 linear gradient IPG strips, and rehydrated for 24 h at room temperature. Then the strips were run on an Ettan IPGphor isoelectric focusing system following the manufacturer’s instruction (GE Healthcare, USA). After focusing, the strips were equilibrated with an equilibration solution containing 1% dithiothreitol, and subsequently 4% iodoacetamide. In the second dimension, the proteins were separated using SDS polyacrylamide gels. Each separation was repeated 3 times to ensure the protein pattern reproducibility. Then, the gels were visualized by the revised Coomassie Brilliant Blue G-250 staining as described^28^, and image analysis was performed with the ImageMaster 2D Platinum Software (Version 5.0, GE Healthcare). The apparent *M*r of each protein in gel was determined by protein markers.

2-D DIGE was performed as described^29^. Each protein samples were labeled with a ratio of 250 pmol of the Cy2, Cy3 or Cy5 protein minimal labeling dye (GE Healthcare) for each 50 μg of proteins. The pooled mixtures of all samples were mixed with 6 μL of Cy2 and were then incubated on ice for 45 min in the dark. The reactions were quenched by the addition of 1 μL (Cy3 or Cy5 reactions) or 6 μL (Cy2 reactions) of 10 mM lysine, vortexed, and incubated on ice for 10 min in the dark. The three labeled and quenched samples were combined with a rehydration buffer (7 M urea, 2 M thiourea, 2% w/v CHAPS) containing 0.5% IPG buffer to bring the final volume to 450 μL. To increase the protein quantity needed for the ensuing protein identification, the preparative gels (IEF/SDS-PAGE) were performed using approximately 1 mg of unlabelled proteins. Then, the Cy2 for S3, Cy3 for S4 and Cy5 for S5 labeled images were acquired using a Typhoon Trio scanner (GE Healthcare). Finally, the 2-D DIGE images were analyzed using the DeCyder 7.0 software (GE Healthcare). Differential in-gel analysis module with an estimated spot number of 5,000 was used for spot detection, and the biological variation analysis module was employed to identify spots differentially expressed in different samples with statistically significant differences. Three biological repeats were examined in each comparison, and the spots of interest were manually checked to confirm spot matching between different gels.

Furthermore, phosphoprotein analysis of the tuberous root proteins was performed. The 2-DE gels were first stained with the Pro-Q Diamond dye to detect phosphoproteins according to the manufacturer’s instructions (Molecular Probes, USA). Then, the gels were restained with the SYPRO Ruby fluorescence stain to visualize the total proteins. Finally, the same gels were restained with Coomassie blue dye G-250 as described^28^,^29^. The images of these Pro-Q, SYPRO Ruby and G-250 stained 2-DE gels were analyzed using the ImageMaster software (GE Healthcare). After spot detection and background subtraction, the protein spots were aligned and matched. The target phosphoprotein spots were excised for MS analysis.

### Protein identification via MALDI TOF/TOF MS

Proteins were identified by MALDI TOF/TOF MS analysis as previously described^28^. First, the proteins were in-gel digested with bovine trypsin as described^27^. After digestion, the protein peptides were collected, and the mass spectra were obtained on an ultraflex III MALDI TOF/TOF MS instrument (Bruker Daltonics, Billerica, MA) equipped with a pulsed N_2_ laser (337 nm). The spectra were analyzed with FlexAnalysis software (Version 3.2, Bruker Daltonics) and searched against the cassava genome database (http://www.cassava-genome.cn/data.html) constructed from the W14 and KU50 clones of cassava^5^ using Mascot software (Version 2.3). The peptide mass fingerprinting (PMF) search parameters were as follows: 100 ppm maximum mass error, MH^+^ monoisotopic mass values, oxidation modifications allowed, 1 missed cleavage allowed, trypsin as the enzyme, and fixed modification of cysteine by carbamidomethylation. In addition, an MS/MS ion search was performed using the above search criteria except with an ion tolerance of ±30 ppm. All MS/MS data from the LIFT spectra and PMF data were combined for database searching. Matches were classified as good if they had a threshold score (p < 0.05) higher than 61 for PMF and 33 for MS/MS ion search. In this research, the scores of PMF and the combined ions were respectively improved to as high as 71 and 50 that means the confidence interval is above 99% (p < 0.01). The identification focused on proteins with higher Mascot scores, maximum peptide coverage, matched peptides, spot position on 2-DE gels, and peptides that were consistently identified with both PMF-based searches and LIFT evidences. In addition, a reversed decoy database searching with a maximum of 0.1% was performed to estimate the percentage of false positive identifications for all the samples. Detailed information for the identified proteins is provided in the Supporting Information (Tables S1 and S2 and Fig. S1–S3). Finally, an in-house BlastP searching of the UniProt database was performed for each protein to identify homologues and to confirm their cellular component, biological process and molecular function.

### Protein function classification, hierarchical clustering and pathway analysis

The sequences of the identified proteins were searched against the UniProt database (http://www.ebi.uniprot.org) to identify their functions. Hierarchical clustering of the expression profiles was performed with a self-organizing tree algorithm (SOTA) using the Cluster Software (version 3.0). Then, gene ontology (GO) analysis was performed by Blast2GO (http://www.blast2go.com), using the GO annotation search tool and data from the NCBI (http://www.ncbi.nlm.nih.gov) and TAIR (http://www.arabidopsis.org) databases. Finally, we performed KEGG pathway analysis (http://www.genome.jp/kegg/pathway) on DEPs (more than 2.0-fold changes in 2-DE and 1.5-fold changes in DIGE).

### Semi-quantitative reverse transcription PCR (RT-PCR) and quantitative RT-PCR (qRT-PCR) analysis

Total RNA was used to generate cDNAs using the reverse transcriptase kit reagents (TaKaRa, Tokyo, Japan). The primer pairs used for both RT-PCR and qRT-PCR were provided in Table S3. Both semi-quantitative RT-PCR and qRT-PCR were repeated at least three times. For PCR, 1 μg of RNA was used for reverse transcription. The cDNA samples were diluted to 5–8 ng/μL. Triplicate quantitative assays were performed by adding 5 μL of each cDNA dilution with the SYBR Green PCR Master mix (TaKaRa), and the measurements were conducted on an Mx3005P sequence detection system according to the manufacturer’s instructions. The gene fragment encoding the cassava 18S RNA was used as the internal control to normalize the amount of template cDNA (Table S3).

### Overexpression of a cassava 14-3-3 gene in *Arabidopsis thaliana*

The cDNA sequence of the cassava 14-3-3 gene (accession No. 67107028) containing the complete open reading frame (*Me14-3-3*) was directly amplified using the 1300F and 1300R primers. The 14-3-3 gene was subcloned into the P-super 1300^+^ vector between the *Xba* I and *Kpn* I sites, resulting in a construct for overexpression of 14-3-3 gene under the control of the CaMV 35S promoter in plants. The construct was introduced into *Agrobacterium tumefaciensstrain* GV 3101 through electroporation. The *A. thaliana* (Columbia ecotype) wild-type plants were transformed by floral dipping. Transgenic plants harboring *35S::14-3-3* were screened on MS medium (Sigma-Aldrich, USA) containing 25 mg/L hygromycin, and the integrity of the transformed gene was further confirmed by PCR amplification using primers for the vector and the 14-3-3 gene.

### Determination of starch and soluble sugar contents

Starch and soluble sugar were extracted and quantified as described^28^ with minor modifications. Fresh samples (approximately 0.5 g) were taken from 15-day-old plants in the morning and were powdered in liquid nitrogen. Then, the samples were boiled with 5 mL of double-distilled water for 30 min, and the extraction procedure was repeated once. The supernatants were filtered and combined in a volumetric flask. Double-distilled water was added to the product to maintain a constant volume for measuring the amount of soluble sugar, which was determined with the anthrone reagent using glucose as the standard. The remaining pellets were boiled for 10 min with 5 mL double-distilled water in 10 mL centrifuge tubes. Then, the samples were cooled to room temperature, and 2 mL HClO_4_ (9.2 mol.L^−1^) was added to decompose the starch. Starch in the paste was hydrolyzed for 15 min, and the amount of soluble glucose was determined at 630 nm^64^. Three biological replicates for each line were made and five technical replicates for each sample. The results were indicated as means ± SD. At the same time, the starch content in the 14-3-3 transgenic *Arabidopsis* was directly visualized by Lugol’s iodine staining reagent (Sigma). The leaves from the mature plants were decolorized with 80% ethanol and immersed in Lugol solution (0.4% KI/0.02% I_2_) for 10 min. The leaves were rinsed with double-distilled water and photographed with a digital camera.

### Southern blotting and Western blotting analyses

Southern blotting was conducted using the Manual DIG Application for Filter Hybridization method. The genomic DNA from the wild type *Arabidopsis* (WT), the transgenic *Arabidopsis* line with the empty vector (LV), and the three 14-3-3 transgenic *Arabidopsis* lines (L1–3) were digested with *Xba* I, electrophoresed on a 0.8% agarose gel, transferred onto a positively charged nylon membrane, and hybridized to a DIG-labeled 14-3-3 gene probe as described in Table S3. Hybridization was performed at 42 °C. The DNA blots were visualized using an ImageQuant LAS 4000 mini.

Western blotting was performed as previously described^28^. Approximately 10 μg of proteins were loaded per lane and 5% non-fat milk was used to block non-specific protein binding. Next, after which the nitrocellulose membrane was incubated with an anti-14-3-3, anti-UDXS and anti-MADH polyclonal antibodies obtained from rabbit serum. The bound rabbit IgGs were detected in separate blots with alkaline phosphatase-conjugated anti-rabbit IgG. For validation of protein expression levels, 3 biological replicates including 3 technical replicates were performed for all Western blotting experiments.

### Statistical analysis

For all generated data, at least three biological replicates were performed for each sample. Then, one-way ANOVA and Duncan’s multiple range tests were performed at a 5% significance level using the SPSS software (version 13.0). The statistical results were reported as the means ± SD.

## Additional Information

**How to cite this article**: Wang, X. *et al.* Proteomics Profiling Reveals Carbohydrate Metabolic Enzymes and 14-3-3 Proteins Play Important Roles for Starch Accumulation during Cassava Root Tuberization. *Sci. Rep.* **6**, 19643; doi: 10.1038/srep19643 (2016).

## Supplementary Material

Supplementary Information
