# Supplementary material for: Proteomics Profiling Reveals Carbohydrate Metabolic Enzymes and 14-3-3 Proteins Play Important Roles for Starch Accumulation during Cassava Root Tuberization
Source: Sci Rep. 2016 Jan 21;6:19643. doi: 10.1038/srep19643 (PMC4726164; doi:10.1038/srep19643)
Supplement: Supplementary Information [file srep19643-s1.pdf]

Supplemental Information

Title: Proteomics Profiling Reveals Carbohydrate Metabolic Enzymes and 14-3-3 Proteins Play Important Roles for Starch Accumulation during Cassava Root Tuberization

Authors:

Xuchu Wang (Corresponding author)

1. Key Laboratory of Biology and Genetic Resources for Tropical Crops, Institute of Tropical Biosciences and Biotechnology, Chinese Academy of Tropical Agricultural Sciences, Haikou Hainan 571101, China

2. College of Agriculture, Hainan University, Haikou, Hainan 570228, China

E-mail: xchwanghainan@163.com

Lili Chang

1. Key Laboratory of Biology and Genetic Resources for Tropical Crops, Institute of Tropical Biosciences and Biotechnology, Chinese Academy of Tropical Agricultural Sciences, Haikou Hainan 571101, China

2. College of Agriculture, Hainan University, Haikou, Hainan 570228, China

Zheng Tong

Key Laboratory of Biology and Genetic Resources for Tropical Crops, Institute of Tropical Biosciences and Biotechnology, Chinese Academy of Tropical Agricultural Sciences, Haikou Hainan 571101, China

Dongyang Wang

1. Key Laboratory of Biology and Genetic Resources for Tropical Crops, Institute of Tropical Biosciences and Biotechnology, Chinese Academy of Tropical Agricultural

Sciences, Haikou Hainan 571101, China

2. College of Agriculture, Hainan University, Haikou, Hainan 570228, China

Qi Yin

1. Key Laboratory of Biology and Genetic Resources for Tropical Crops, Institute of Tropical Biosciences and Biotechnology, Chinese Academy of Tropical Agricultural Sciences, Haikou Hainan 571101, China

2. College of Agriculture, Hainan University, Haikou, Hainan 570228, China

Dan Wang

Key Laboratory of Biology and Genetic Resources for Tropical Crops, Institute of Tropical Biosciences and Biotechnology, Chinese Academy of Tropical Agricultural Sciences, Haikou Hainan 571101, China

Xiang Jin

Key Laboratory of Biology and Genetic Resources for Tropical Crops, Institute of Tropical Biosciences and Biotechnology, Chinese Academy of Tropical Agricultural Sciences, Haikou Hainan 571101, China

Qian Yang

Key Laboratory of Biology and Genetic Resources for Tropical Crops, Institute of Tropical Biosciences and Biotechnology, Chinese Academy of Tropical Agricultural Sciences, Haikou Hainan 571101, China

Liming Wang

Key Laboratory of Biology and Genetic Resources for Tropical Crops, Institute of Tropical Biosciences and Biotechnology, Chinese Academy of Tropical Agricultural

Sciences, Haikou Hainan 571101, China

Yong Sun

Key Laboratory of Biology and Genetic Resources for Tropical Crops, Institute of Tropical Biosciences and Biotechnology, Chinese Academy of Tropical Agricultural Sciences, Haikou Hainan 571101, China

Qixing Huang

Key Laboratory of Biology and Genetic Resources for Tropical Crops, Institute of Tropical Biosciences and Biotechnology, Chinese Academy of Tropical Agricultural Sciences, Haikou Hainan 571101, China

Anping Guo (**Co-corresponding author**)

Key Laboratory of Biology and Genetic Resources for Tropical Crops, Institute of Tropical Biosciences and Biotechnology, Chinese Academy of Tropical Agricultural Sciences, Haikou Hainan 571101, China

E-mail: xchwanghainanlab@163.com

Ming Peng (**Co-corresponding author**)

1. Key Laboratory of Biology and Genetic Resources for Tropical Crops, Institute of Tropical Biosciences and Biotechnology, Chinese Academy of Tropical Agricultural Sciences, Haikou Hainan 571101, China

2. College of Agriculture, Hainan University, Haikou, Hainan 570228, China

E-mail: xchwanghainanlab@163.com

Supplementary Figures and Tables

Supplementary Fig. S1. Yields and 1-DE profiles of total proteins extracted from cassava main roots at different development stages.

Supplementary Fig. S2. Typical MS spectra and identification information of proteins from common 2-DE gels.

Supplementary Fig. S3. Typical MS spectra and identification of proteins from DIGE gels.

Supplementary Fig. S4. Main pathway ways overexpressed in all the differential proteins.

Supplementary Table S1. Information for differential protein spots on 2-DE gels and their corresponding MS identities.

Supplementary Table S2. Information for differential proteins on 2-D DIGE gels and the corresponding MS identities.

Supplementary Table S3. Primers used for RT and Q-PCR analysis.

Supplemental Figure S1: Yields (A) and 1-DE profiles (B) of total proteins extracted from cassava main roots at different development stages.

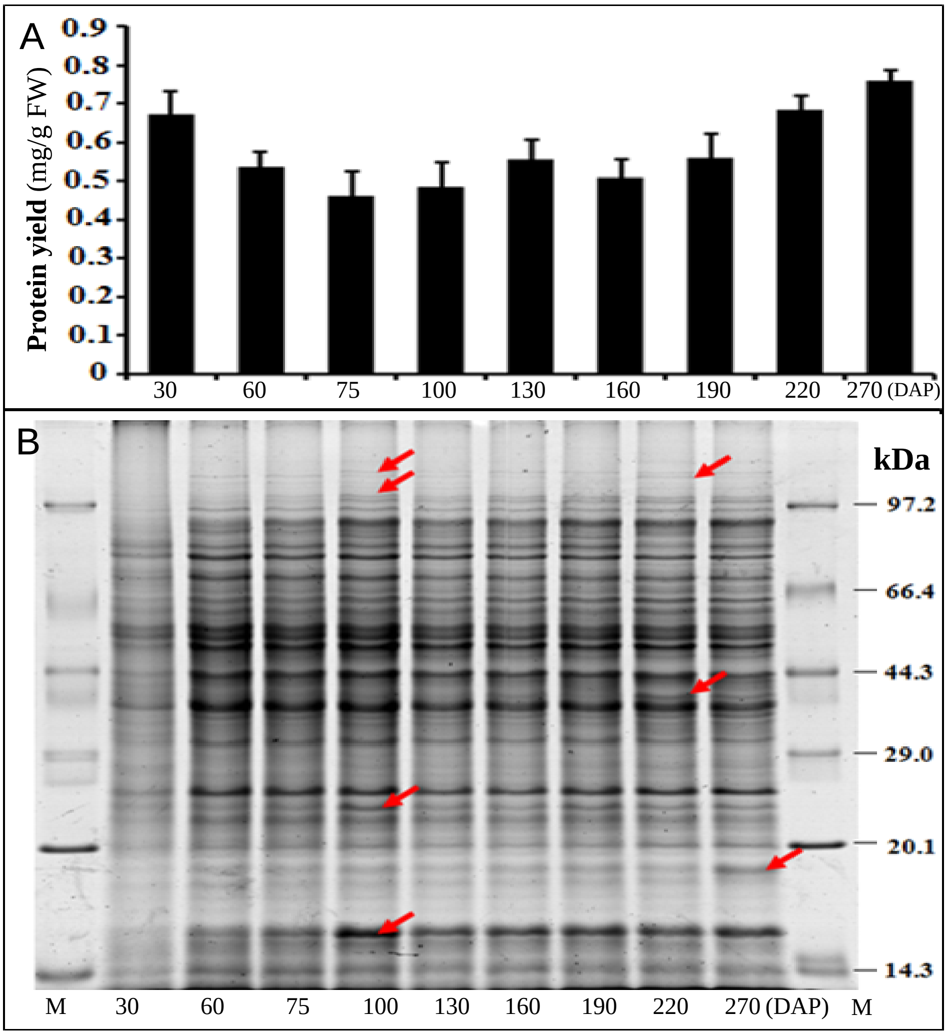

The cassava main roots were collected at 30, 60, 75, 100, 130, 160, 190, 220 and 270 (Stages S1-S9, respectively) days after planting. M, molecular weight markers. Arrows indicated the major differential protein bands on the 1-DE gels.

Supplemental Figure S2:
Supplemental spectra and MALDI TOF/TOF MS/MS identification information from common 2-DE gels

Annotated spectra for the 118 proteins identified by PMF and PFF.

CID: collision induced dissociation

MALDI TOF:

matrix assisted laser desorption/ionization time of flight

MS: mass spectrometry

PMF: peptide mass fingerprinting

PFF: peptide fragment fingerprinting

Spot No.: **1**

NCBI accession No.: **cassava4.1_006707m|PACid:17966570**

Protein name： **hypothetical protein**

Plant species: ***Manihot esculenta*** Mascot score: **92**

Sequence coverage %: **27** p value: **0.0011**

Matched peptides No.: **13** Total peptides No.: **44**

Calculated Mr: **55987** Calculated *p*I: **9.32**

Annotated PMF **spectra:**

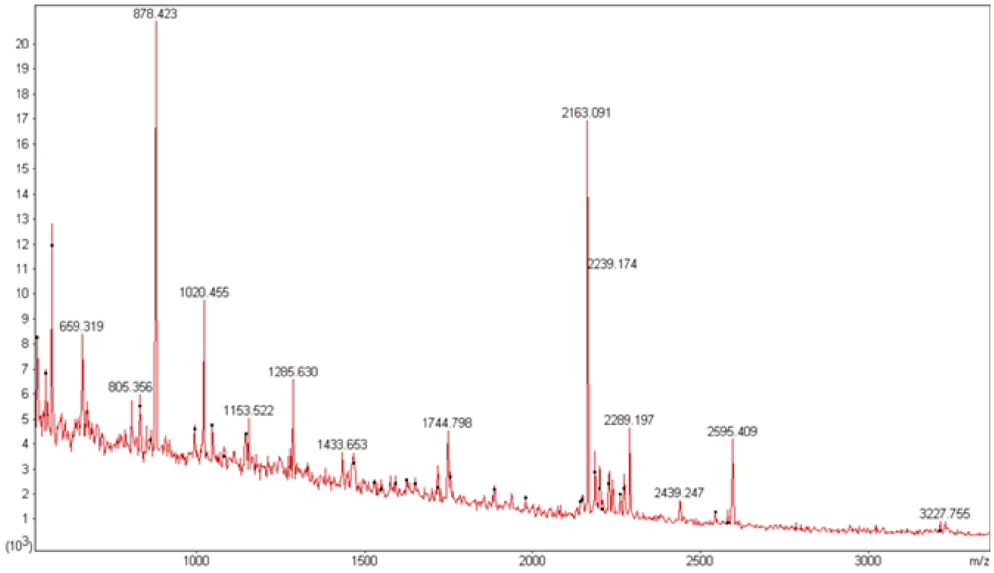

Probability Based Mowse Score:

Protein score is -10*Log(P), where P is the probability that the observed match is a random event.
Protein scores greater than 61 are significant (p<0.05).

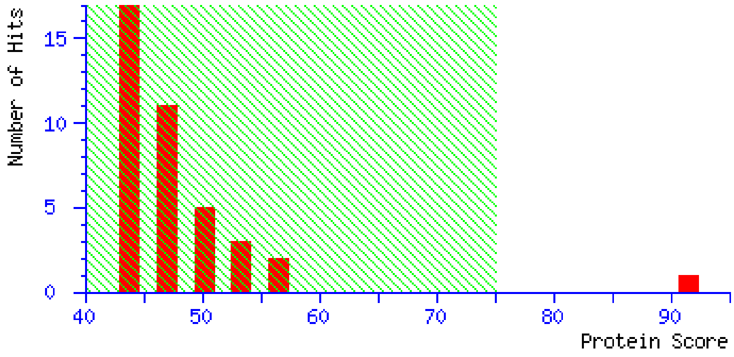

Spot No.: **2**

NCBI accession No.: **cassava4.1_004263m|PACid:17968763**

Protein name： **Chaperonin 60 subunit alpha 1, chloroplastic**

Plant species: ***Manihot esculenta***

Mascot score: **215**

Sequence coverage %: **47** p value: **2.3e-017**

Matched peptides No.: **29** Total peptides No.: **64**

Calculated Mr: **62302** Calculated *p*I: **5.34**

Annotated PMF **spectra:**

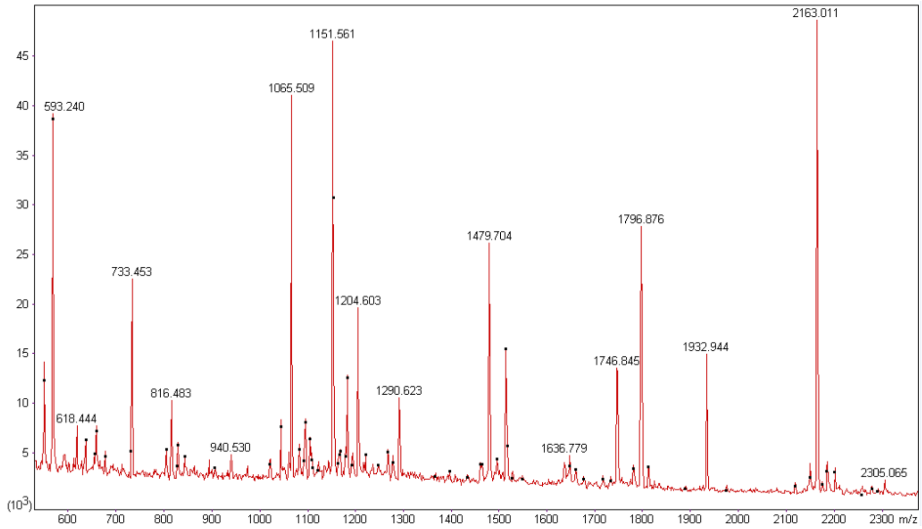

Probability Based Mowse Score:

Protein score is -10*Log(P), where P is the probability that the observed match is a random event. Protein scores greater than 61 are significant (p<0.05).

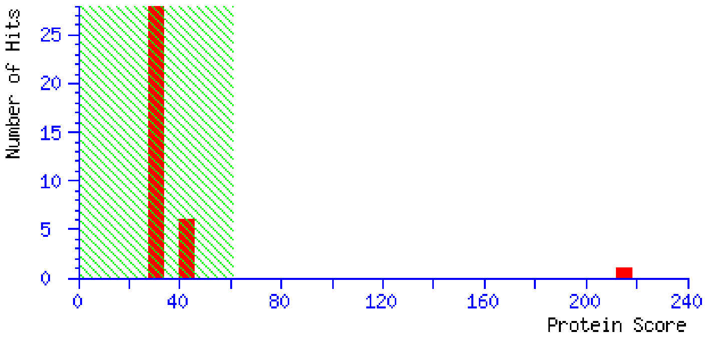

Spot No.: **3**

NCBI accession No.: **cassava4.1_006418m|PACid:17966937**

Protein name: **V-type proton ATPase subunit B1**

Plant species: ***Manihot esculenta*** Mascot score: **97**

Sequence coverage %: **46** p value: 1.5e-005

Matched peptides No.: **18** Total peptides No.: **75**

Calculated Mr: **54476** Calculated *p*I: **4.99**

Annotated PMF **spectra:**

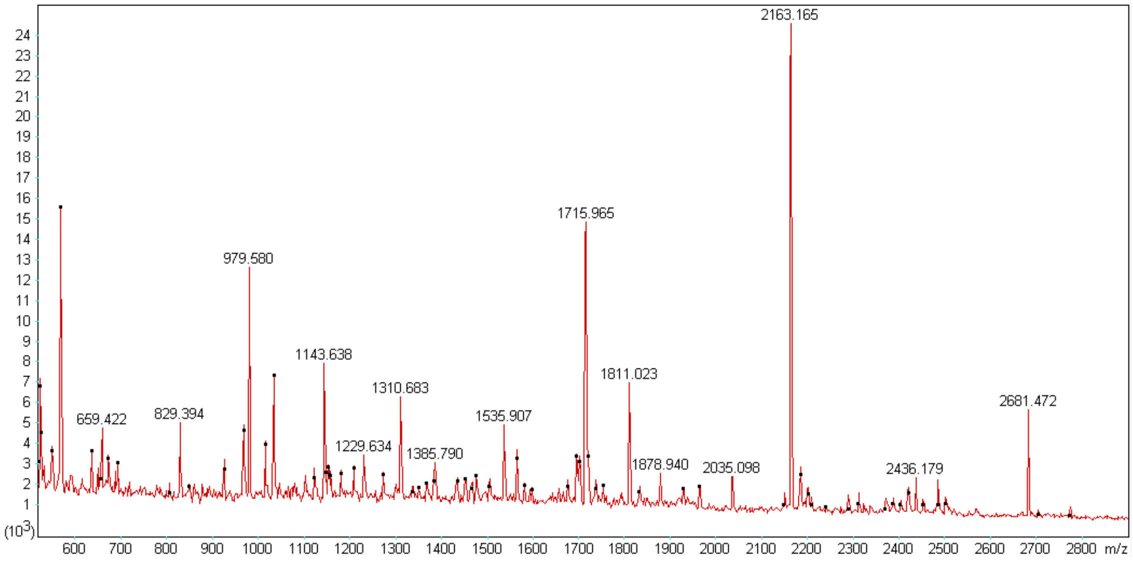

Probability Based Mowse Score:

Protein score is -10*Log(P), where P is the probability that the observed match is a random event. Protein scores greater than 61 are significant (p<0.05).

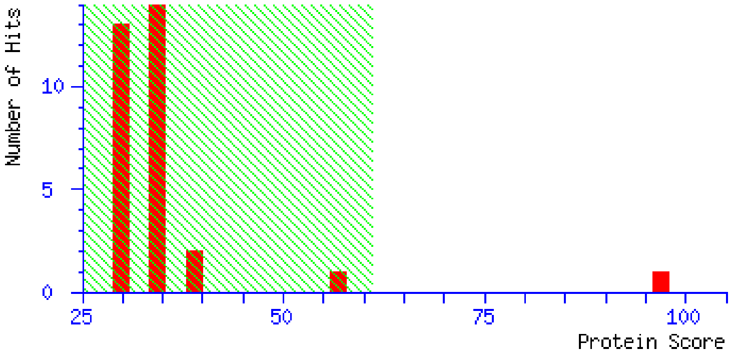

Spot No.: **4**

NCBI accession No.: **cassava4.1_006418m|PACid:17966937**

Protein name： **V-type proton ATPase subunit B2**

Plant species: ***Manihot esculenta*** Mascot score: **187**

Sequence coverage %: **48** p value: **1.5e-014**

Matched peptides No.: **27** Total peptides No.: **77**

Calculated Mr: **54476** Calculated p*I*: **4.99**

Annotated PMF **spectra:**

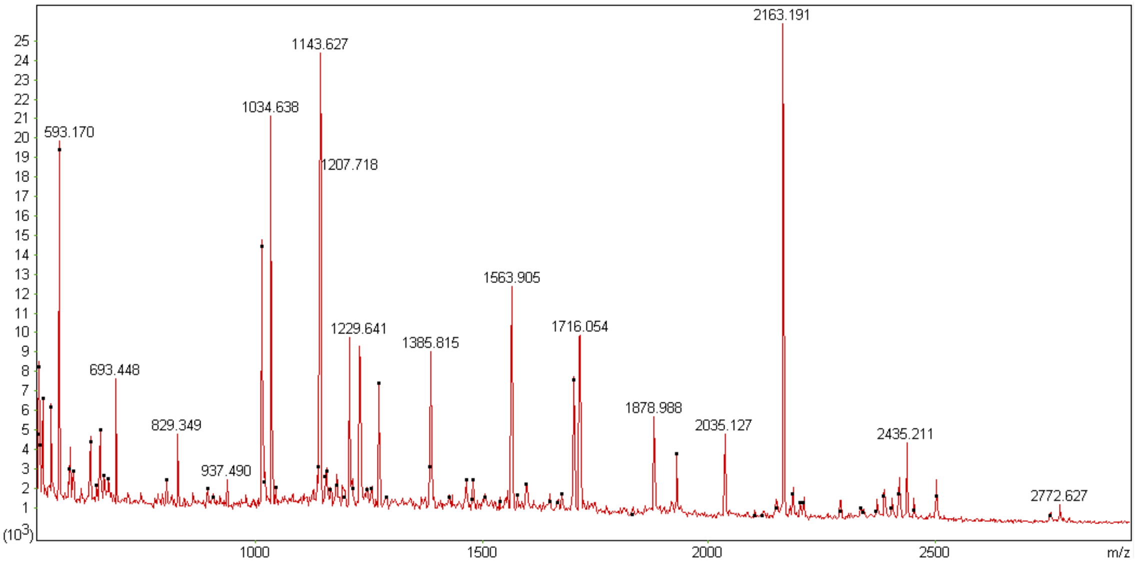

Probability Based Mowse Score:

Protein score is -10*Log(P), where P is the probability that the observed match is a random event. Protein scores greater than 61 are significant (p<0.05).

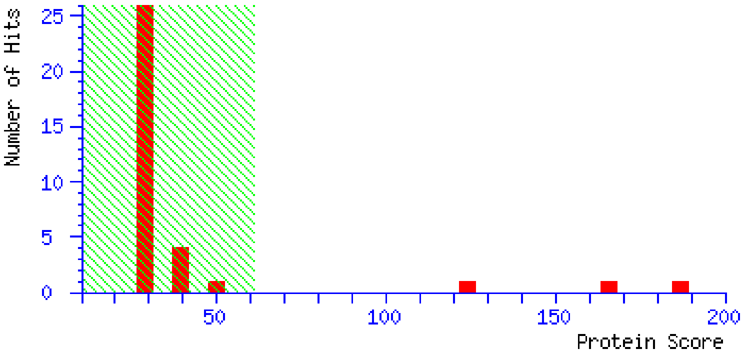

Spot No.: **5**

NCBI accession No.: **cassava4.1_007971m|PACid:17991617**

Protein name：**Tubulin alpha-6 chain**

Plant species: ***Manihot esculenta*** Mascot score: **101**

Sequence coverage %: **51** p value: **5.8e-006**

Matched peptides No.: **15** Total peptides No.: **65**

Calculated Mr: **48294** Calculated *p*I: **4.95**

Annotated PMF **spectra:**

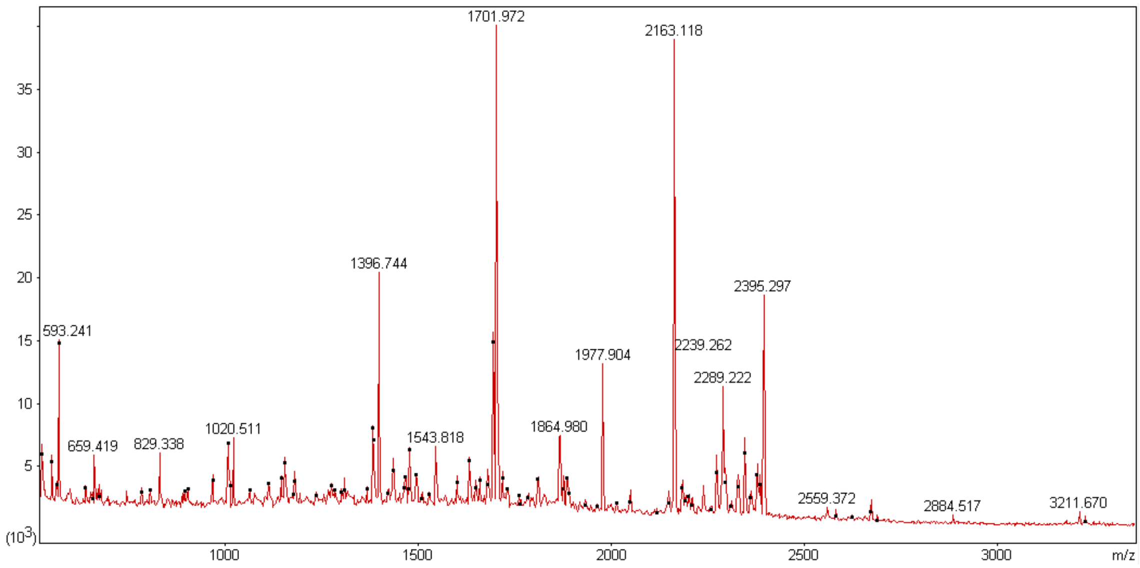

Probability Based Mowse Score:

Protein score is -10*Log(P), where P is the probability that the observed match is a random event.
Protein scores greater than 61 are significant (p<0.05).

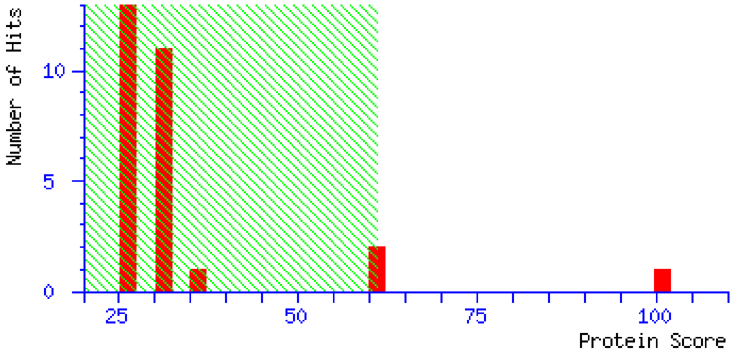

Spot No.: **6**

NCBI accession No.: **cassava4.1_008310m|PACid:17966609**

Protein name： **26S protease regulatory subunit 6A homolog A**

Plant species: ***Manihot esculenta*** Mascot score: **154**

Sequence coverage %: **59** p value: **2.9e-11**

Matched peptides No.: **29** Total peptides No.: **93**

Calculated Mr: **47700** Calculated *p*I: **4.99**

Annotated PMF **spectra:**

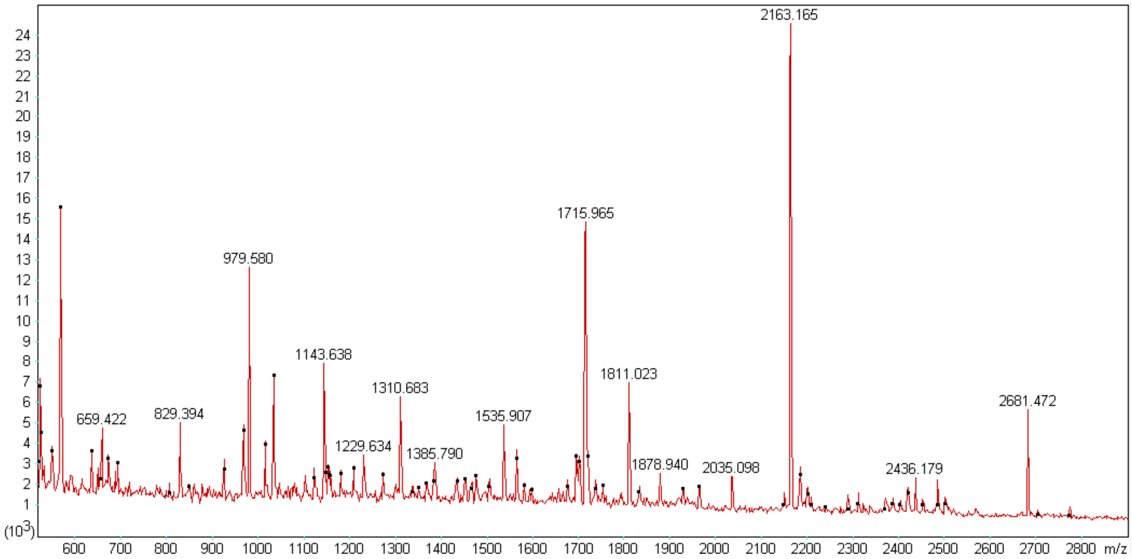

Probability Based Mowse Score:

Protein score is -10*Log(P), where P is the probability that the observed match is a random event. Protein scores greater than 61 are significant (p<0.05).

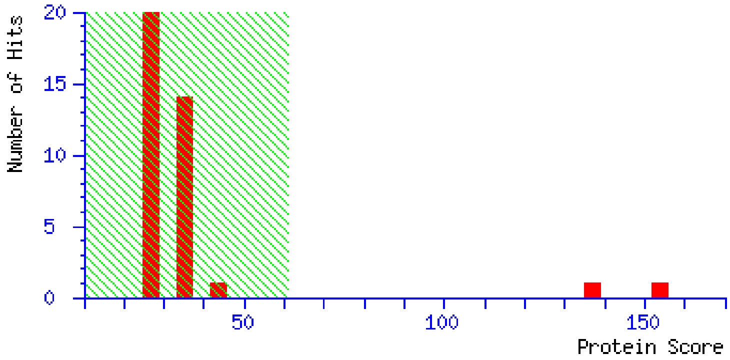

Spot No.: **7**

NCBI accession No.: **cassava4.1_003343m|PACid:17989500**

Protein name： **Probable mediator of RNA polymerase II transcription subunit 37e** Plant species: ***Manihot esculenta***

Mascot score: **115**

Sequence coverage %: **32** p value: 2. 3e-007

Matched peptides No.: **22** Total peptides No.: **60**

Calculated Mr: **71515** Calculated *p*I: **5.10**

Annotated PMF **spectra:**

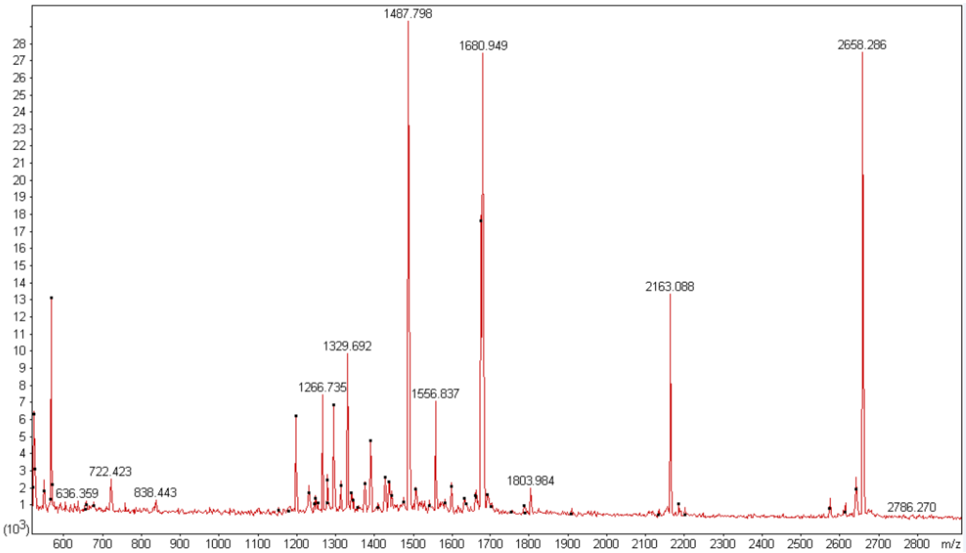

Probability Based Mowse Score:

Protein score is -10*Log(P), where P is the probability that the observed match is a random event.
Protein scores greater than 61 are significant (p<0.05).

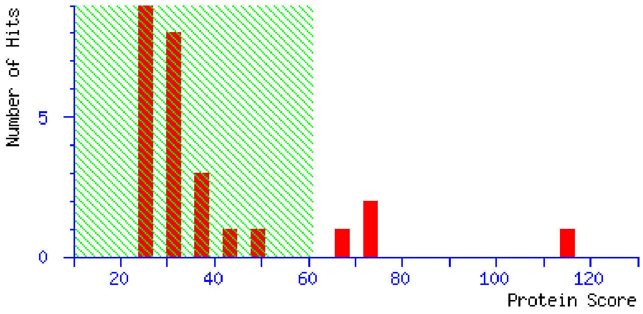

Spot No.: **8**

NCBI accession No.: **cassava4.1_003331m|PACid:17988720**

Protein name：**Probable mediator of RNA polymerase II transcription subunit 37e**

Plant species: ***Manihot esculenta*** Mascot score: **98**

Sequence coverage %: **40** p value: 1. 2e−005

Matched peptides No.: **23** Total peptides No.: **78**

Calculated Mr: **71431** Calculated *p*I: **5.14**

Annotated PMF **spectra:**

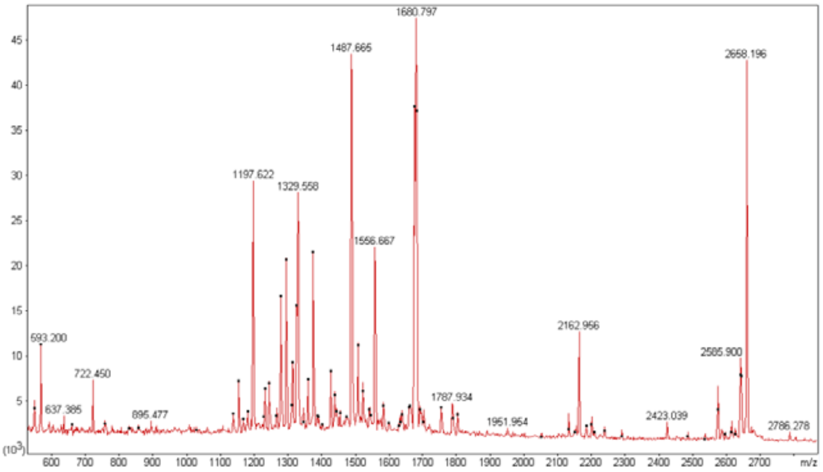

Probability Based Mowse Score:

Protein score is -10*Log(P), where P is the probability that the observed match is a random event. Protein scores greater than 61 are significant (p<0.05).

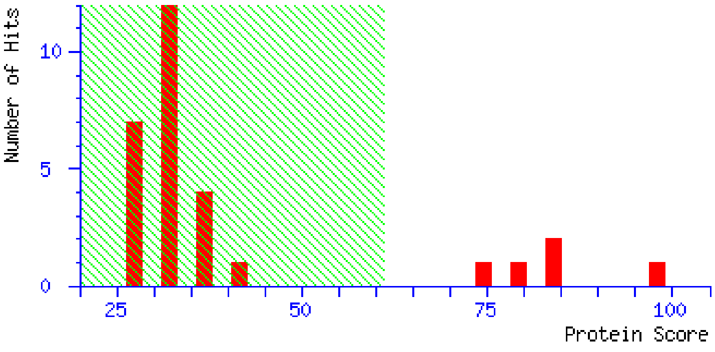

Spot No.: **9**

NCBI accession No.: **cassava4.1_003340m|PACid:17988693**

Protein name：**Probable mediator of RNA polymerase II transcription subunit 37c**

Plant species: ***Manihot esculenta***Mascot score: **128**

Sequence coverage %: **40** p value: 1. 2e−008

Matched peptides No.: **29** Total peptides No.: **74**

Calculated Mr: **71461** Calculated *p*I: **5.17**

Annotated PMF **spectra:**

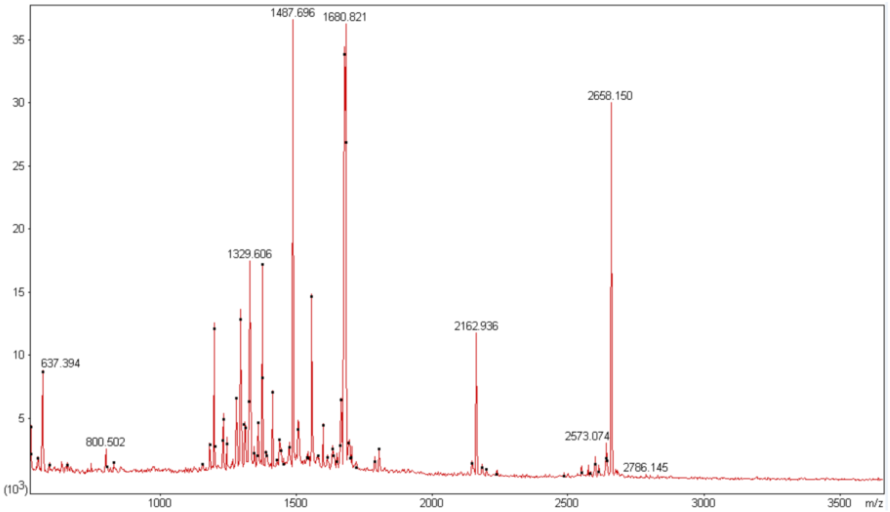

Probability Based Mowse Score:

Protein score is -10*Log(P), where P is the probability that the observed match is a random event. Protein scores greater than 61 are significant (p<0.05).

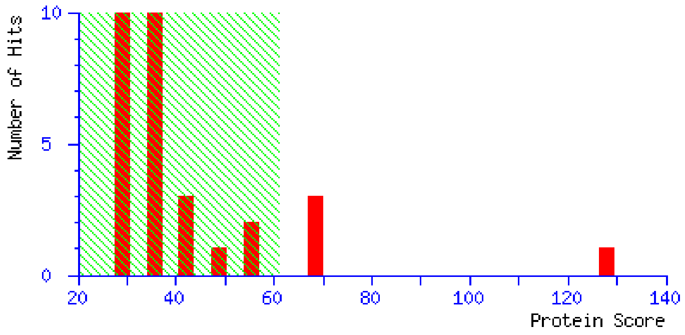

Spot No.: **10**

NCBI accession No.: **cassava4.1_033108m|PACid:17973616**

Protein name： **Actin-7** Plant species: ***Manihot esculenta***

Mascot score: **127**

Sequence coverage %: **48** p value: 1. 5e−008

Matched peptides No.: **15** Total peptides No.: **62**

Calculated Mr: **41897** Calculated *p*I: **5.31**

Annotated PMF **spectra:**

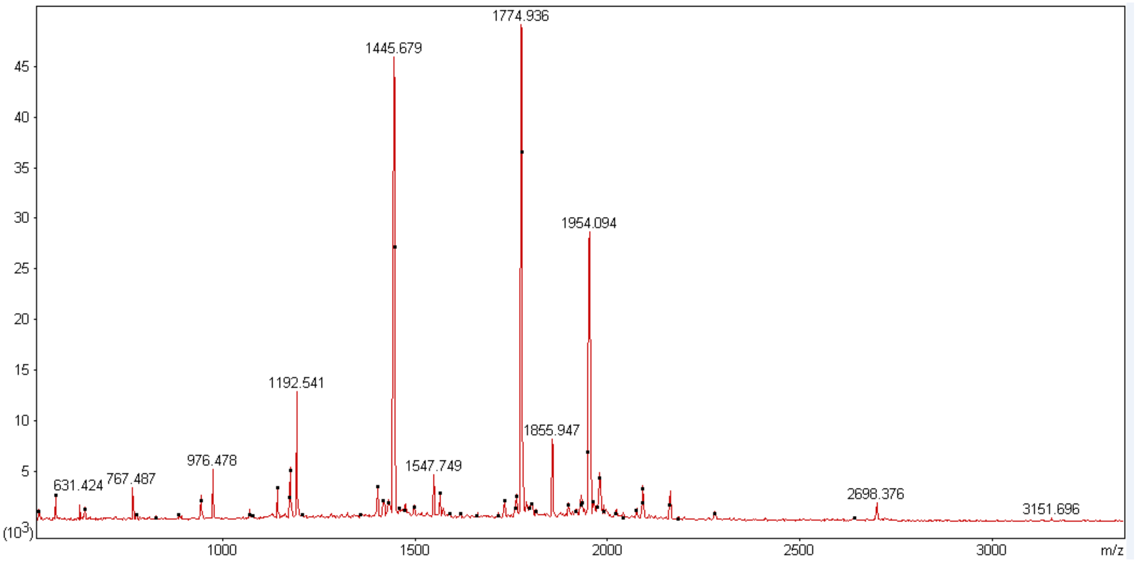

Probability Based Mowse Score:

Protein score is -10*Log(P), where P is the probability that the observed match is a random event. Protein scores greater than 61 are significant (p<0.05).

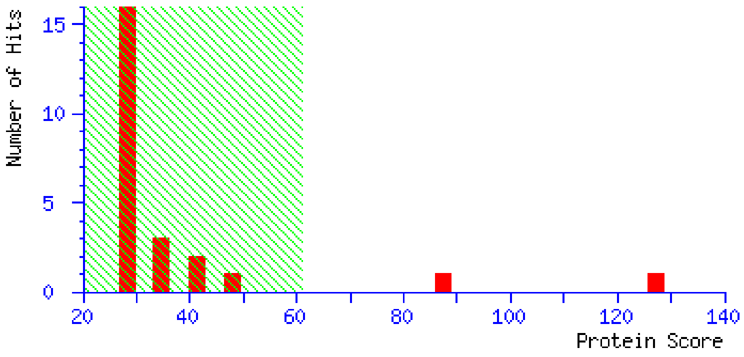

Spot No.: **11**

NCBI accession No.: **cassava4.1_003907m|PACid:17972056**

Protein name: **Chaperonin 60 subunit beta 2, chloroplastic** Plant species: ***Manihot esculenta*** Mascot score: **147**

Sequence coverage %: **46** p value: 1. 5e−010

Matched peptides No.: **23** Total peptides No.: **63**

Calculated Mr: **64742** Calculated *p*I: **5.62**

Annotated PMF **spectra:**

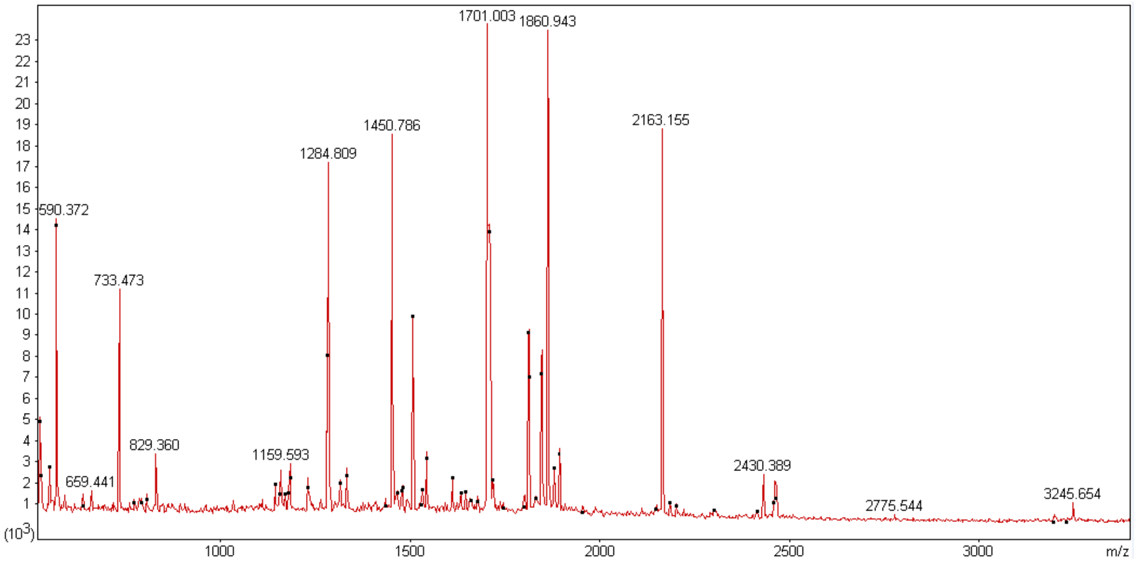

Probability Based Mowse Score:

Protein score is -10*Log(P), where P is the probability that the observed match is a random event. Protein scores greater than 61 are significant (p<0.05).

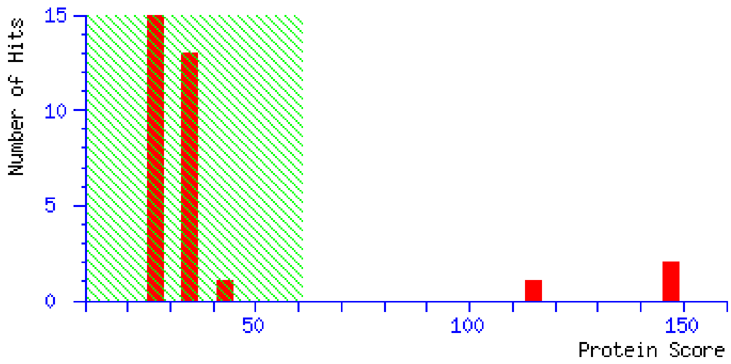

Spot No.: **12**

NCBI accession No.: **cassava4.1_003883m|PACid:17972412**

Protein name：**Chaperonin 60 subunit beta 1, chloroplastic**

Plant species: ***Manihot esculenta*** Mascot score: **73**

Sequence coverage %: **31**   p value: 0. 0035

Matched peptides No.: **15**   Total peptides No.: **57**

Calculated Mr: **64920**   Calculated *p*I: **5.68**

Annotated PMF **spectra:**

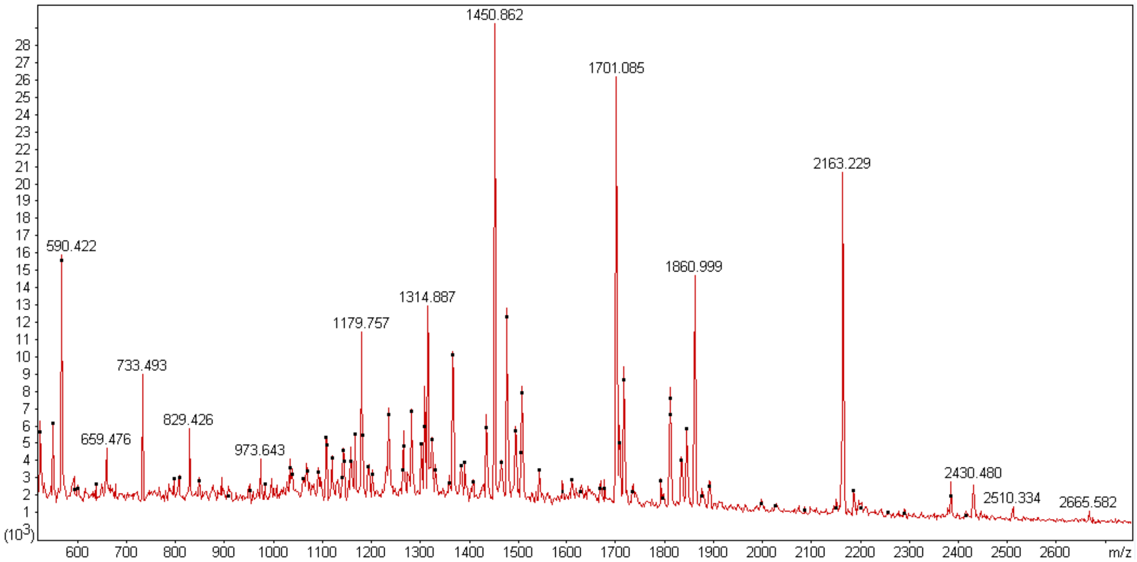

Probability Based Mowse Score:

Protein score is -10*Log(P), where P is the probability that the observed match is a random event. Protein scores greater than 61 are significant (p<0.05).

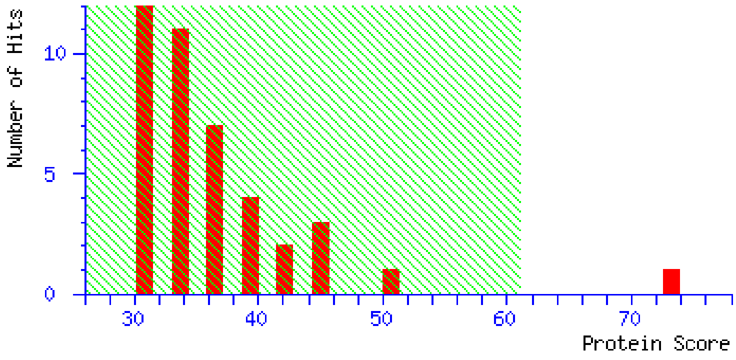

Spot No.: **13**

NCBI accession No.: **cassava4.1_004458m|PACid:17962184**

Protein name：**Chaperonin CPN60, mitochondrial**

Plant species: ***Manihot esculenta*** Mascot score: **143**

Sequence coverage %: **49** p value: 3. 7e−010

Matched peptides No.: **28** Total peptides No.: **73**

Calculated Mr: **61582** Calculated *p*I: **5.71**

Annotated PMF **spectra:**

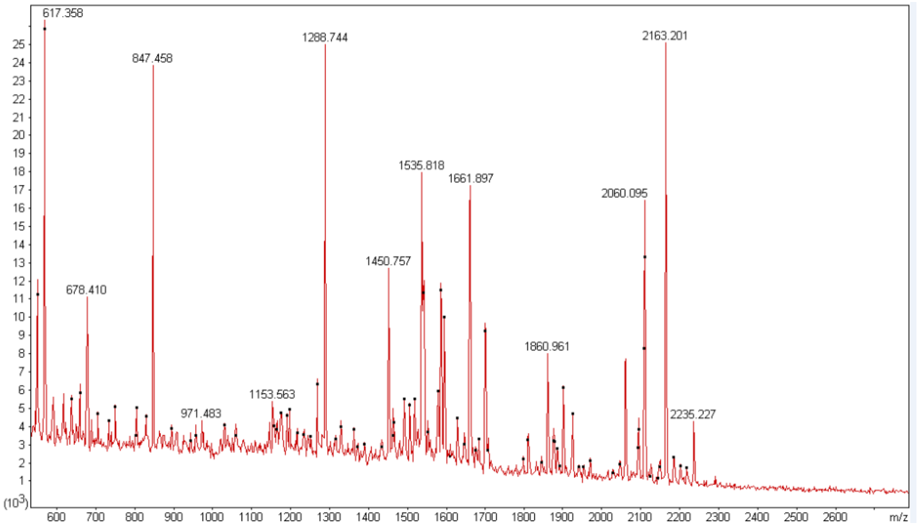

Probability Based Mowse Score:

Protein score is -10*Log(P), where P is the probability that the observed match is a random event.
Protein scores greater than 61 are significant (p<0.05).

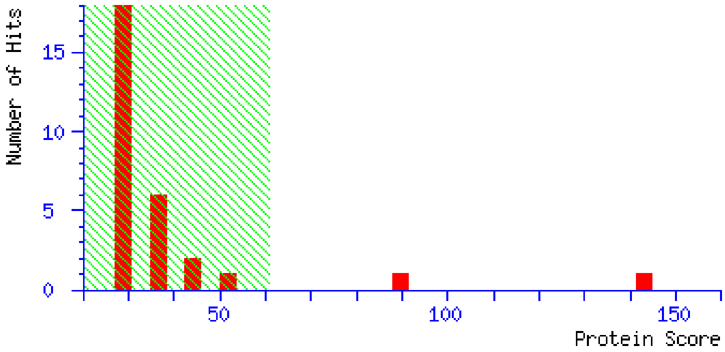

Spot No.: **14**

NCBI accession No.: **cassava4.1_007513m|PACid:17961628**

Protein name：**Tubulin alpha-6 chain** Plant species: ***Manihot esculenta*** Mascot score: **87**

Sequence coverage %: **40** p value: 0. 00014

Matched peptides No.: **19** Total peptides No.: **80**

Calculated Mr: **50342** Calculated *p*I: **4.90**

Annotated PMF **spectra:**

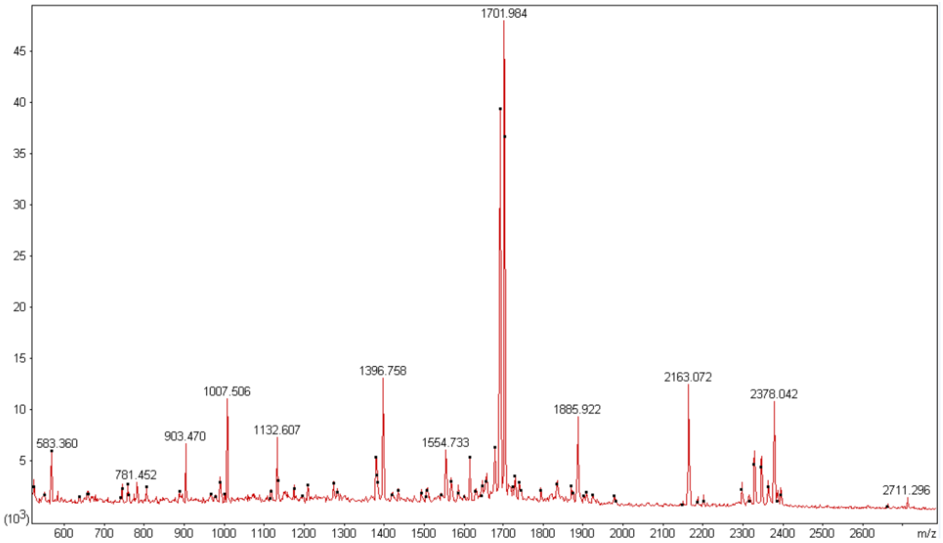

Probability Based Mowse Score:

Protein score is -10*Log(P), where P is the probability that the observed match is a random event.
Protein scores greater than 61 are significant (p<0.05).

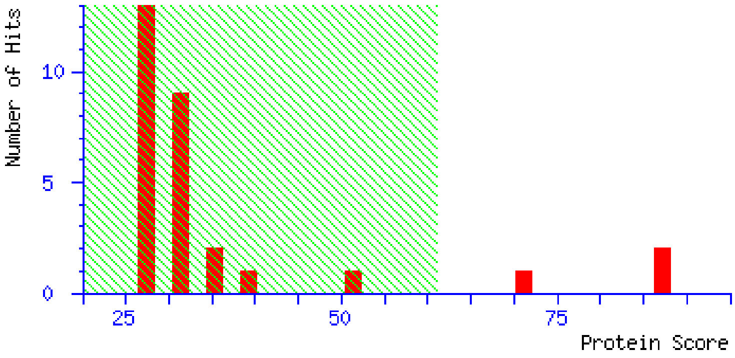

Spot No.: **15**

NCBI accession No.: **cassava4.1_007971m|PACid:17991617**

Protein name： **Tubulin alpha-6 chain**

Plant species: ***Manihot esculenta*** Mascot score: **103**

Sequence coverage %: **48** p value: 3. 7e−006

Matched peptides No.: **18** Total peptides No.: **79**

Calculated Mr: **48294** Calculated *p*I: **4.95**

Annotated PMF **spectra:**

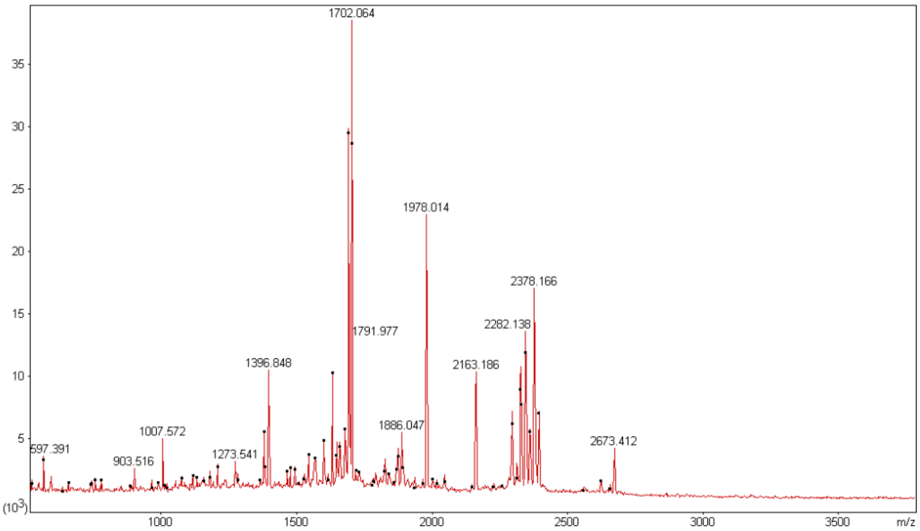

Probability Based Mowse Score:

Protein score is -10*Log(P), where P is the probability that the observed match is a random event. Protein scores greater than 61 are significant (p<0.05).

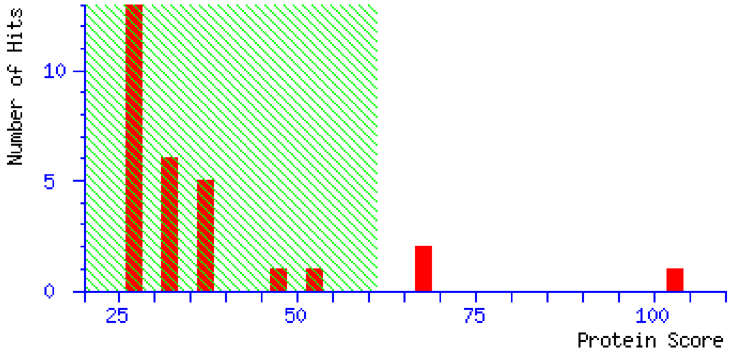

Spot No.: **16**

NCBI accession No.: **cassava4.1_007560m|PACid:17972004**

Protein name：**Tubulin alpha-6 chain**

Plant species: ***Manihot esculenta*** Mascot score: **89**

Sequence coverage %: **41** p value: 9e−005

Matched peptides No.: **17** Total peptides No.: **75**

Calculated Mr: **50295** Calculated *p*I: **5.00**

Annotated PMF **spectra:**

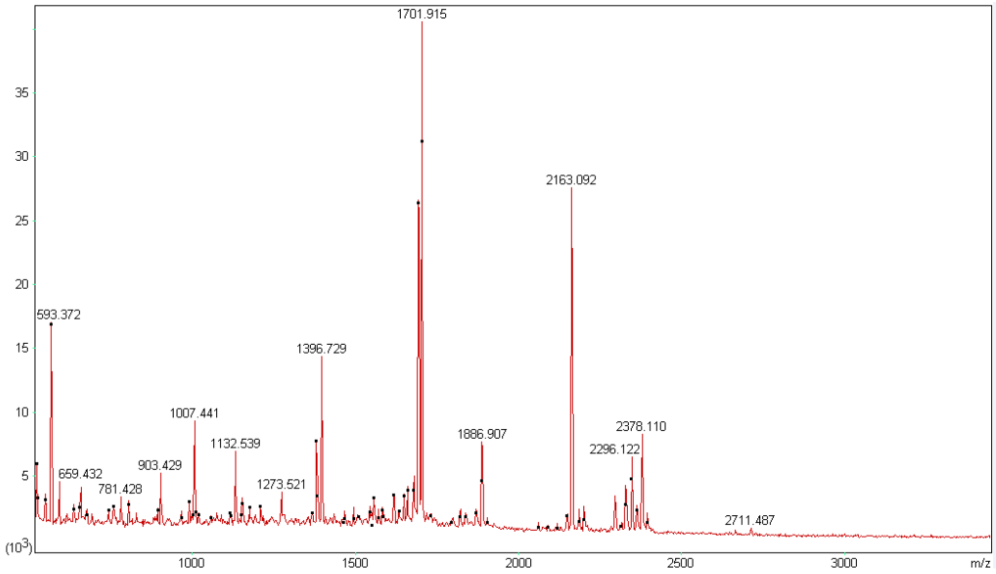

Probability Based Mowse Score:

Protein score is -10*Log(P), where P is the probability that the observed match is a random event.
Protein scores greater than 61 are significant (p<0.05).

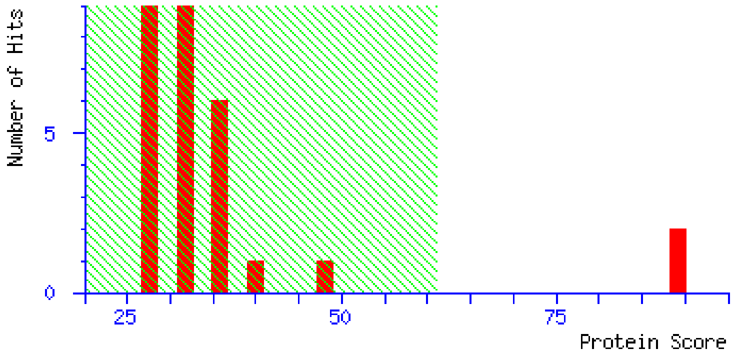

Spot No.: **17**

NCBI accession No.: **cassava4.1_033108m|PACid:17973616**

Plant species: ***Manihot esculenta***

Protein name: **Actin-7** Mascot score: **173**

Sequence coverage %: **70** p value: **3.7e-013**

Matched peptides No.: **24** Total peptides No.: **90**

Calculated Mr: **41897** Calculated *p*I: **5.31**

Annotated PMF **spectra:**

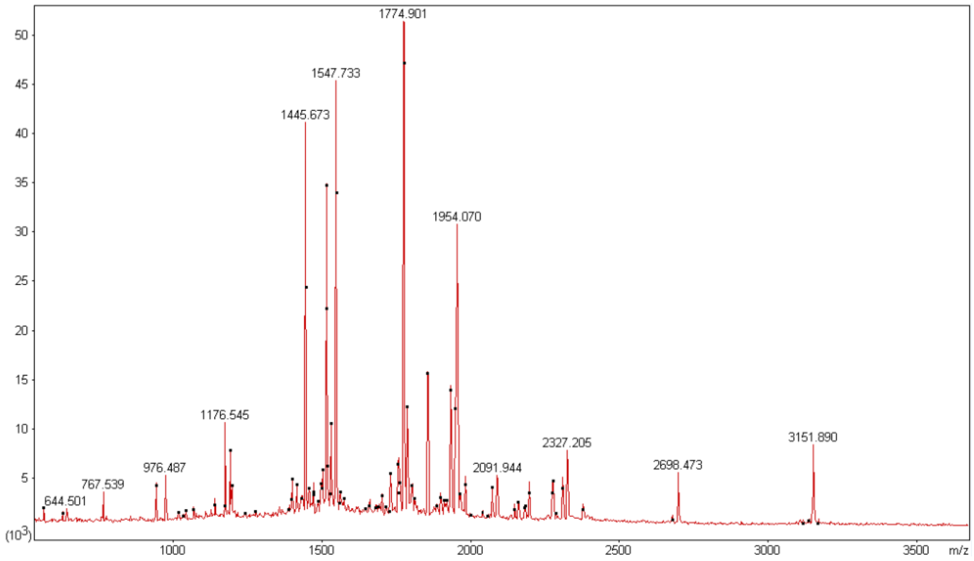

Probability Based Mowse Score:

Protein score is -10*Log(P), where P is the probability that the observed match is a random event. Protein scores greater than 61 are significant (p<0.05).

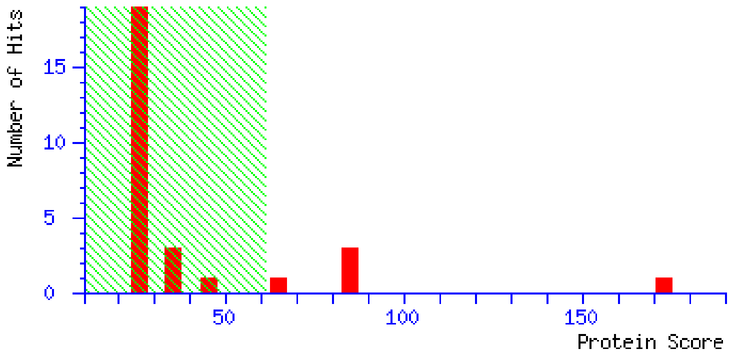

Spot No.: **18**

NCBI accession No.: **cassava4.1_004726m|PACid:17976529**

Protein name：**ATP synthase beta subunit 1** Plant species:

Manihot esculenta Mascot score: **190**

Sequence coverage %: **62** p value: 7.3e-015

Matched peptides No.: **29** Total peptides No.: **89**

Calculated Mr: **60085** Calculated *p*I: **6.13**

Annotated PMF **spectra:**

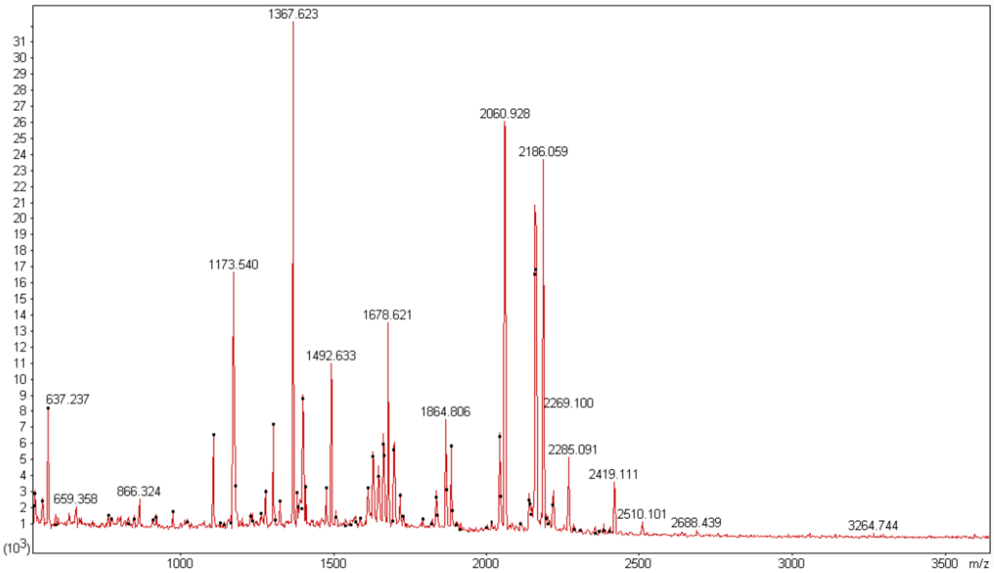

Probability Based Mowse Score:

Protein score is -10*Log(P), where P is the probability that the observed match is a random event.
Protein scores greater than 61 are significant (p<0.05).

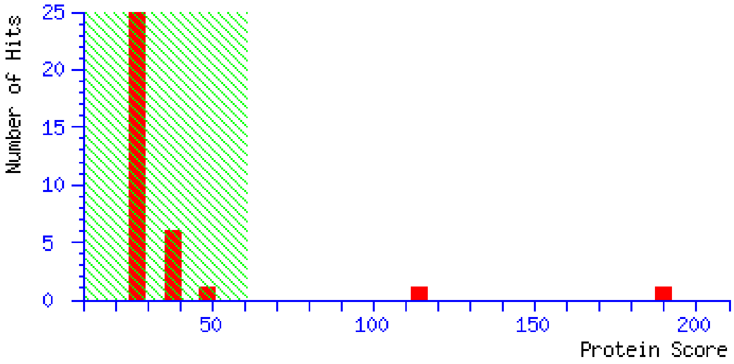

Spot No.: **19**

NCBI accession No.: **cassava4.1_004630m|PACid:17991144**

Plant species: ***Manihot esculenta***

Protein name: **ATP synthase subunit beta-3, mitochondrial**

Mascot score: **175**

Sequence coverage %: **61** p value: **2.3e-013**

Matched peptides No.: **29** Total peptides No.: **63**

Calculated Mr: **60594** Calculated *p*I: **6.26**

Annotated PMF **spectra:**

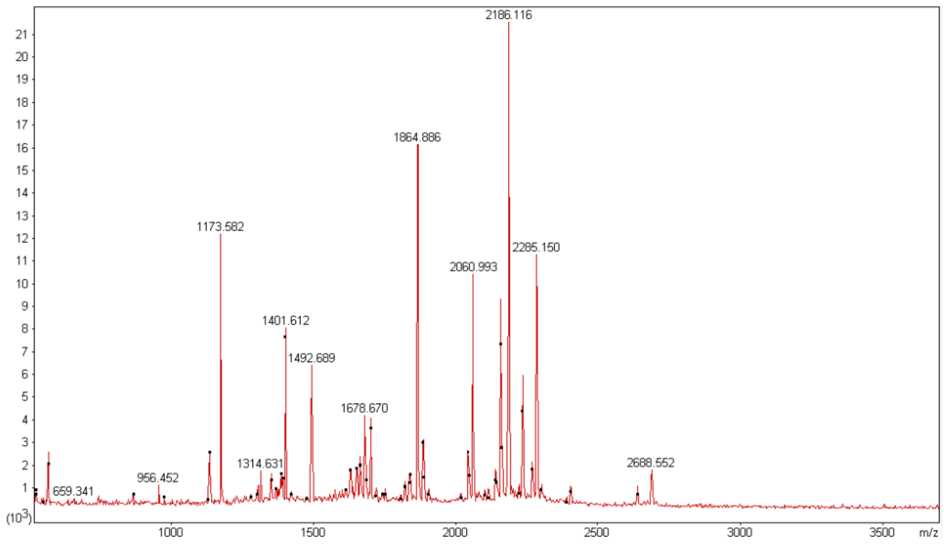

Probability Based Mowse Score:

Protein score is -10*Log(P), where P is the probability that the observed match is a random event. Protein scores greater than 61 are significant (p<0.05).

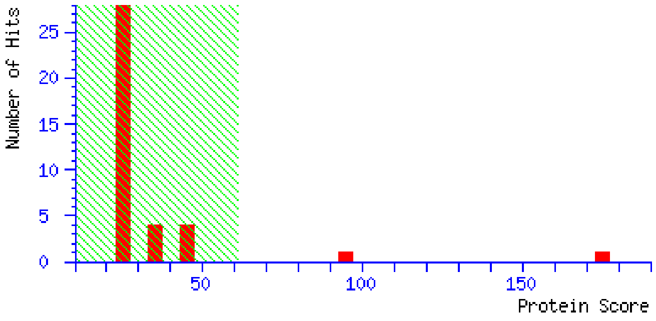

Spot No.: **20**

NCBI accession No.: **cassava4.1_005518m|PACid:17993865**

Plant species: ***Manihot esculenta***

Protein name：**Glucose-1-phosphate adenylyltransferase**

Mascot score: **182**

Sequence coverage %: **58**   p value: **4.6e-014**

Matched peptides No.: **30**   Total peptides No.: **84**

Calculated Mr: **57581**   Calculated *p*I: **6.69**

Annotated PMF **spectra:**

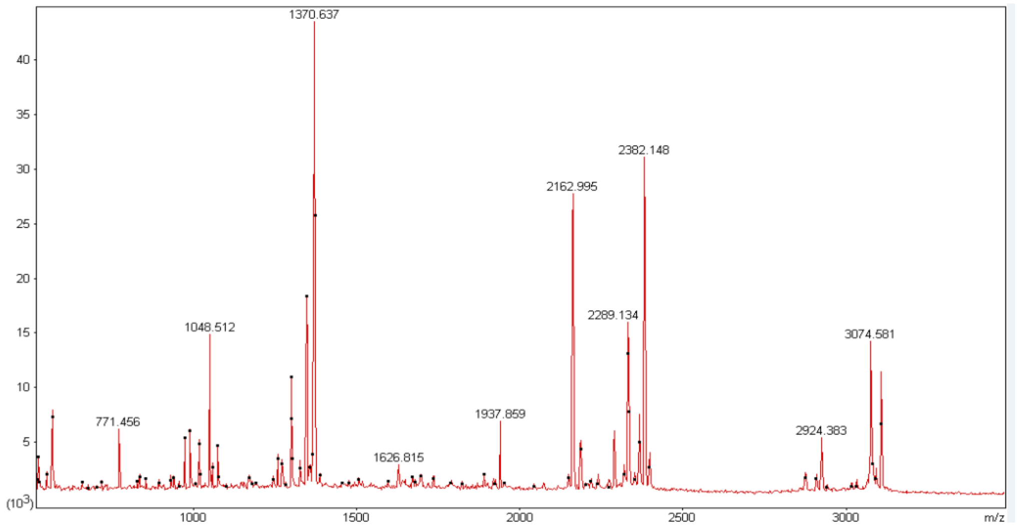

Probability Based Mowse Score:

Protein score is -10*Log(P), where P is the probability that the observed match is a random event.
Protein scores greater than 61 are significant (p<0.05).

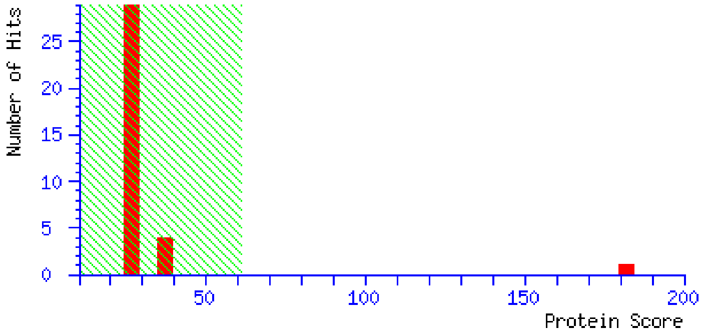

Spot No.: **21**

NCBI accession No.: **cassava4.1_008086m|PACid:17989319**

Plant species: ***Manihot esculenta***

Protein name: **Glutamine synthetase cytosolic isozyme 1-1**

Mascot score: **101**

Sequence coverage %: **33** p value: **5.8e-006**

Matched peptides No.: **14** Total peptides No.: **54**

Calculated Mr: **47502** Calculated *p*I: **6.27**

Annotated PMF **spectra:**

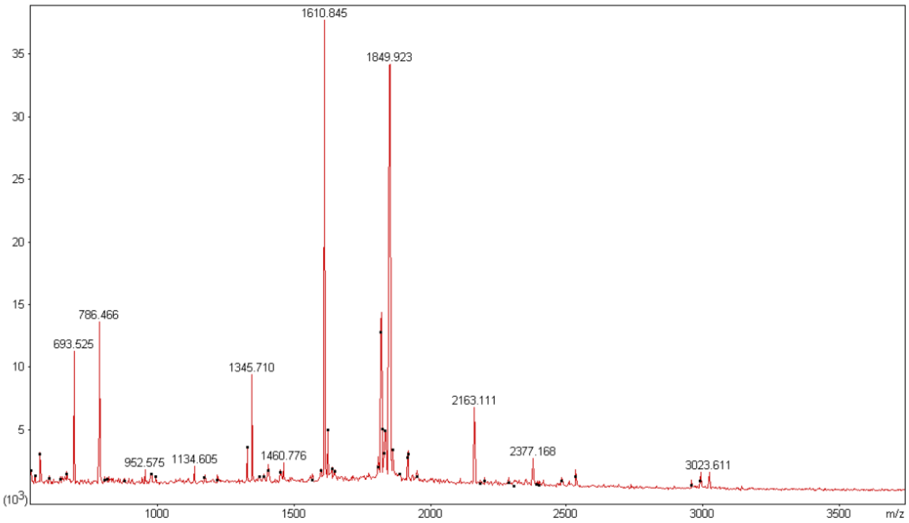

Probability Based Mowse Score:

Protein score is -10*Log(P), where P is the probability that the observed match is a random event. Protein scores greater than 61 are significant (p<0.05).

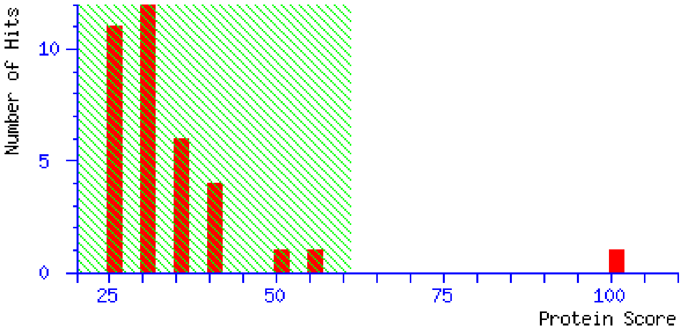

Spot No.: **22**

NCBI accession No.: **cassava4.1_033108m|PACid:17973616**

Plant species: ***Manihot esculenta***

Protein name： **Actin-7**

Mascot score: **96**

Sequence coverage %: **44**　　　p value: **2e-005**

Matched peptides No.: **15**　　　Total peptides No.: **64**

Calculated Mr: **41897**　　　Calculated *p*I: **5.31**

Annotated PMF **spectra:**

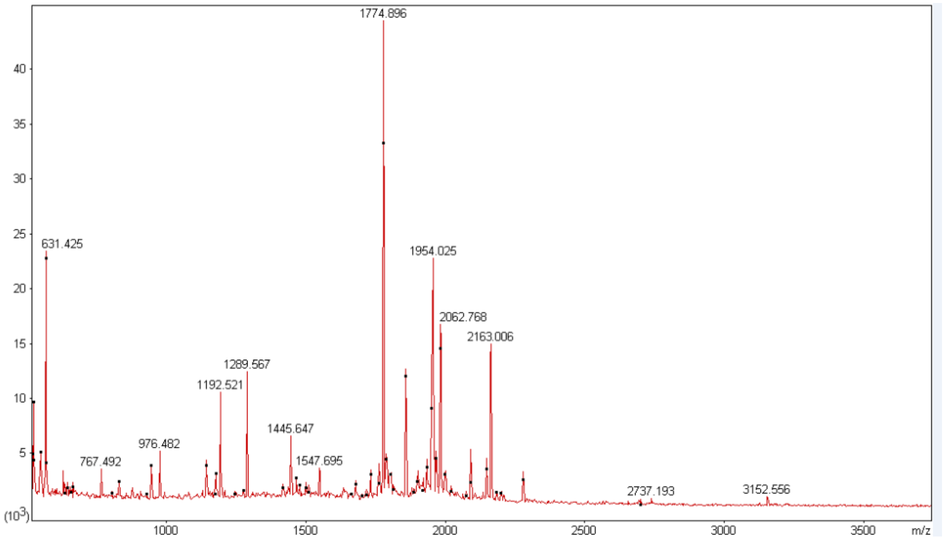

Probability Based Mowse Score:

Protein score is -10*Log(P), where P is the probability that the observed match is a random event.
Protein scores greater than 61 are significant (p<0.05).

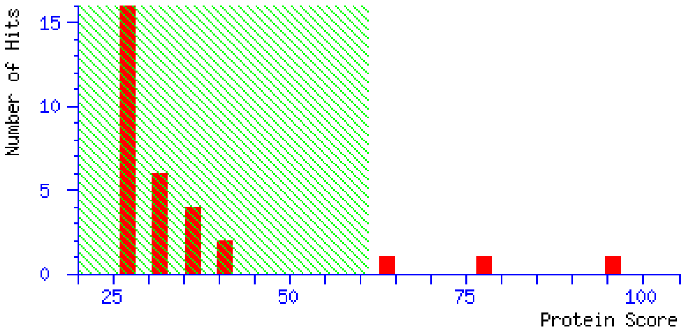

Spot No.: **23**

NCBI accession No.: **cassava4.1_033108m|PACid:17973616**

Plant species: ***Manihot esculenta***

Protein name：**Actin-7**

Mascot score: **93**

Sequence coverage %: **52** p value: **3.4e-005**

Matched peptides No.: **18** Total peptides No.: **87**

Calculated Mr: **41897** Calculated *p*I: **5.31**

Annotated PMF **spectra:**

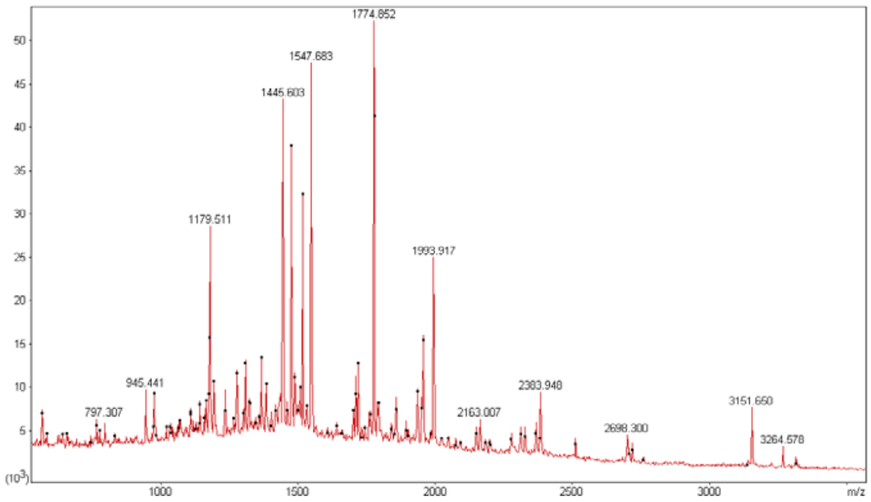

Probability Based Mowse Score:

Protein score is -10*Log(P), where P is the probability that the observed match is a random event.
Protein scores greater than 61 are significant (p<0.05).

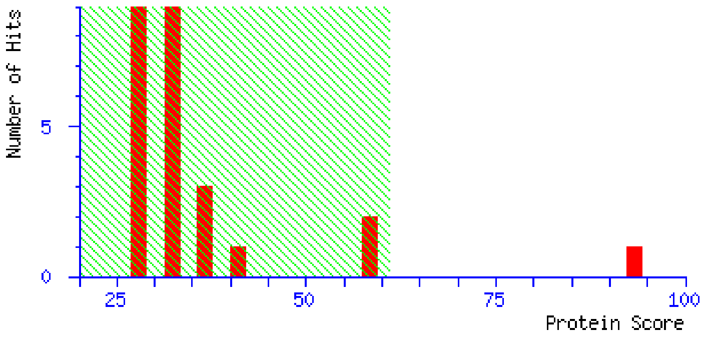

Spot No.: 24

NCBI accession No.: **cassava4.1_033108m|PACid:17973616**

Plant species: ***Manihot esculenta***

Protein name： **Actin-7** Mascot score: **195**

Sequence coverage %: **63** p value: **2.3e-015**

Matched peptides No.: **24** Total peptides No.: **85**

Calculated Mr: **41897** Calculated *p*I: **5.31**

Annotated PMF **spectra:**

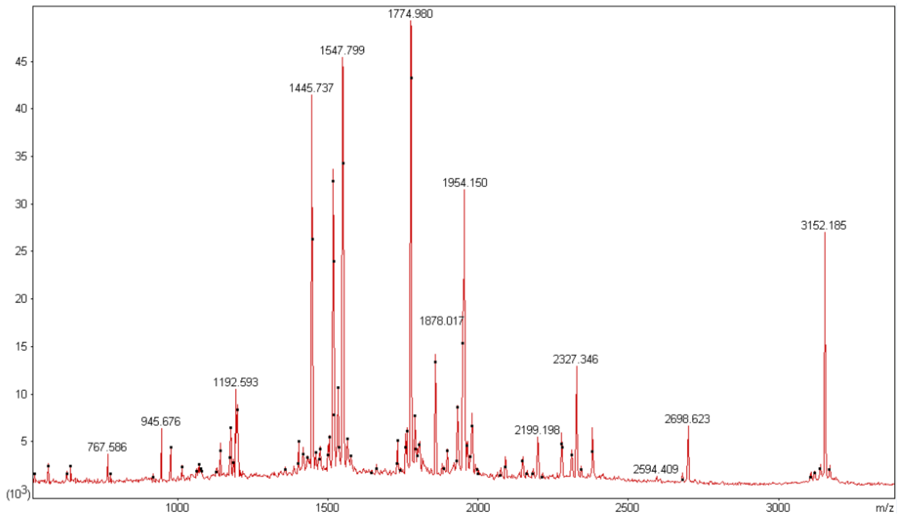

Probability Based Mowse Score:

Protein score is -10*Log(P), where P is the probability that the observed match is a random event. Protein scores greater than 61 are significant (p<0.05).

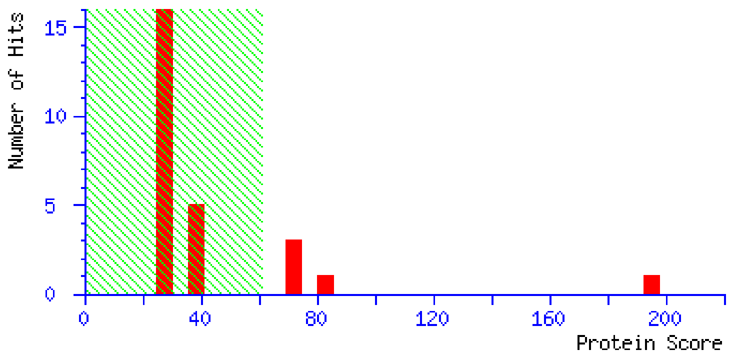

Spot No.: **25**

NCBI accession No.: **cassava4.1_008387m|PACid:17968420**

Plant species: ***Manihot esculenta***

Protein name：**Succinyl-CoA ligase [ADP-forming] subunit beta, mitochondrial**

Mascot score: **128**

Sequence coverage %: **47**

p value: **1.2e-008**

Matched peptides No.: **22**

Total peptides No.: **78**

Calculated Mr: **45532**

Calculated *p*I: **5.88**

Annotated PMF **spectra:**

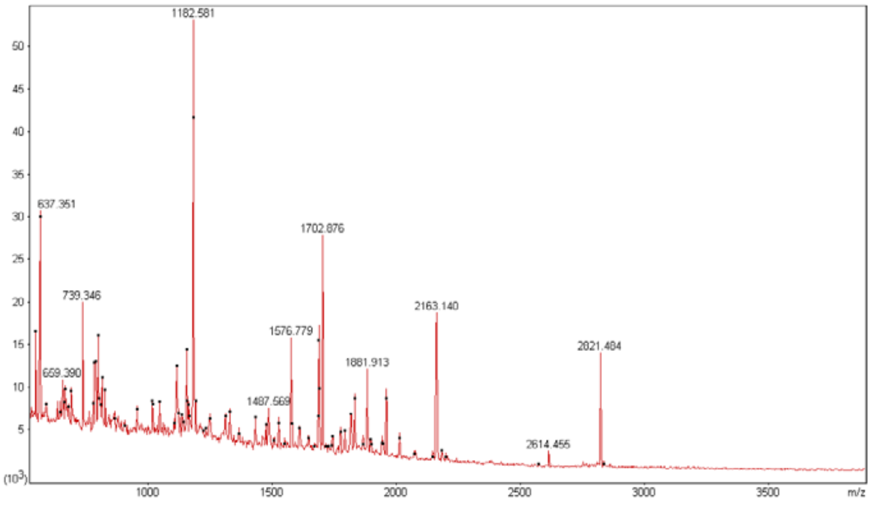

Probability Based Mowse Score:

Protein score is -10*Log(P), where P is the probability that the observed match is a random event.
Protein scores greater than 61 are significant (p<0.05).

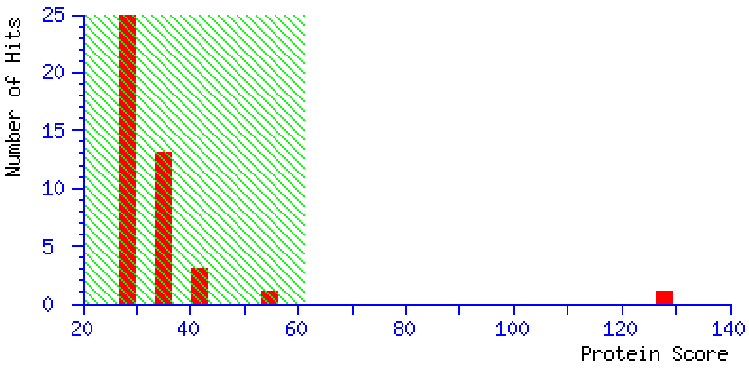

Spot No.: **26**

NCBI accession No.: **cassava4.1_010597m|PACid:17964465**

Plant species: **Manihot esculenta**

Protein name：**Glutamine synthetase cytosolic isozyme 1-1**

Mascot score: **107**

Sequence coverage %: **51**   p value: **1.5e-006**

Matched peptides No.: **15**   Total peptides No.: **80**

Calculated Mr: **39359**   Calculated *p*I: **5.37**

Annotated PMF **spectra:**

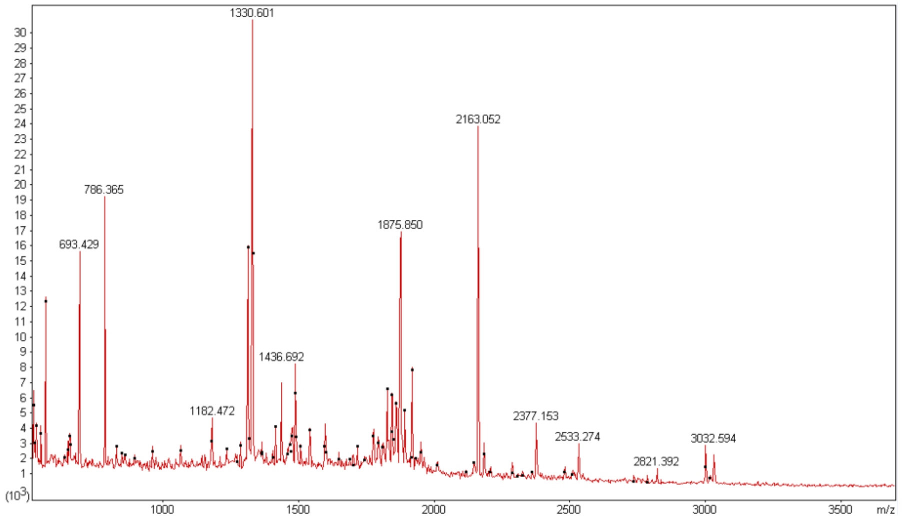

Probability Based Mowse Score:

Protein score is -10*Log(P), where P is the probability that the observed match is a random event.
Protein scores greater than 61 are significant (p<0.05).

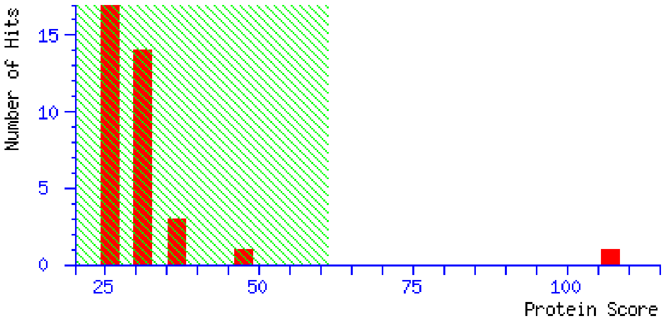

Spot No.: **27**

NCBI accession No.: **cassava4.1_011177m|PACid:17972447**

Protein name： **Adenosine kinase 1** Plant species: ***Manihot esculenta***

PFF Mascot score: [113] **Sequence coverage %:** [7]

Matched peptides No.: [**2**] p value: 3.1e-006

Calculated Mr: **38107** Calculated *p*I: **5.38**

Annotated PMF **spectra:**

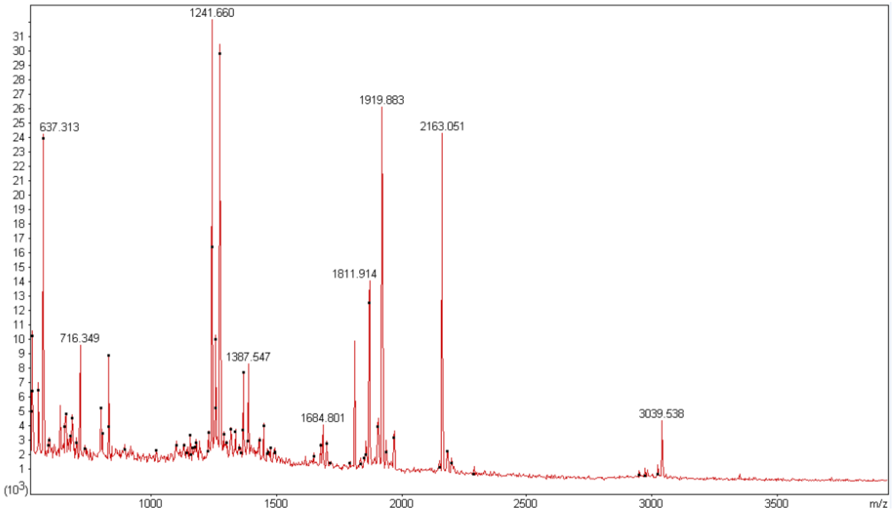

PFF Searched Score:

Ions score is -10*Log(P), where P is the probability that the observed match is a random event.
Individual ions scores > 33 indicate identity or extensive homology (p<0.05).
Protein scores are derived from ions scores as a non-probabilistic basis for ranking protein hits.

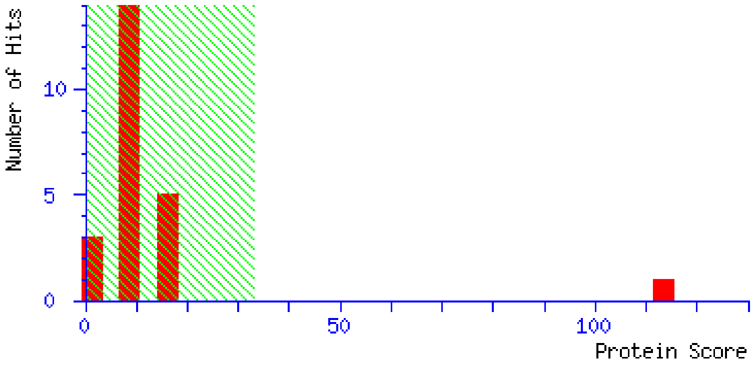

Spot No.: **28**

NCBI accession No.: **cassava4.1_002747m|PACid:17960141**

Plant species: ***Manihot esculenta***

Protein name: **Heat shock protein 90-3** Mascot score: **127**

Sequence coverage %: **37** p value: **1.5e-008**

Matched peptides No.: **33** Total peptides No.: **77**

Calculated Mr: **80250** Calculated *p*I: **4.99**

Annotated PMF **spectra:**

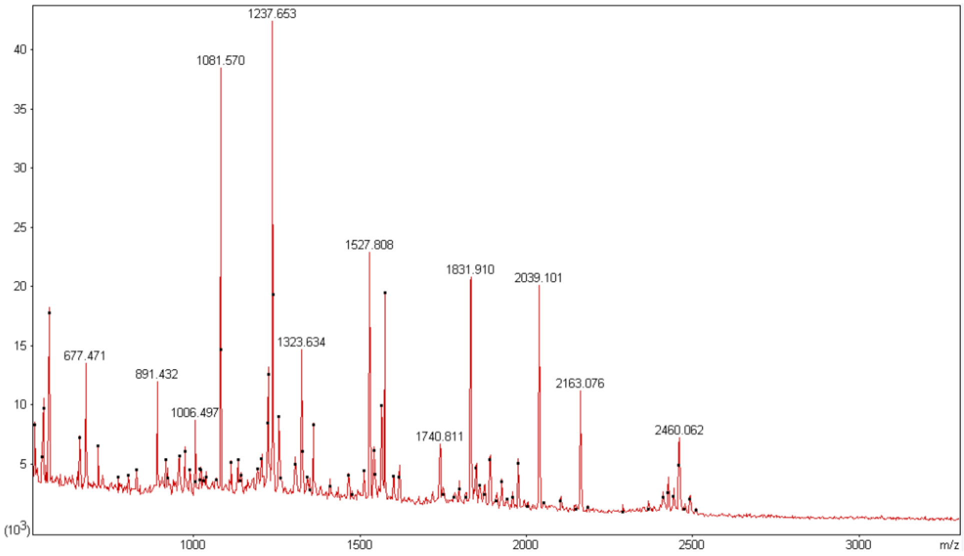

Probability Based Mowse Score:

Protein score is -10*Log(P), where P is the probability that the observed match is a random event.
Protein scores greater than 61 are significant (p<0.05).

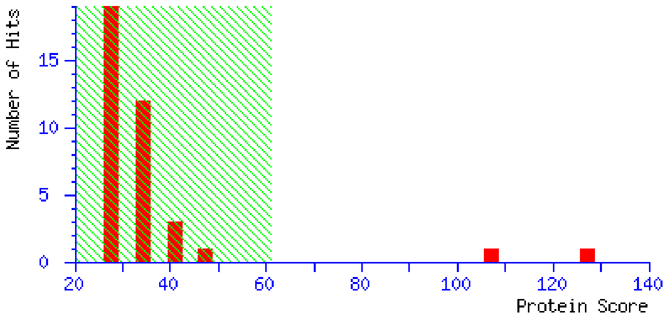

Spot No.: **29**

NCBI accession No.: **cassava4.1_002466m|PACid:17978799**

Plant species: ***Manihot esculenta***

Protein name： **Alpha-glucan phosphorylase 1**

Mascot score: **81**

Sequence coverage %: **24** p value: **0.00058**

Matched peptides No.: **24** Total peptides No.: **95**

Calculated Mr: **81918** Calculated *p*I: **8.50**

Annotated PMF **spectra:**

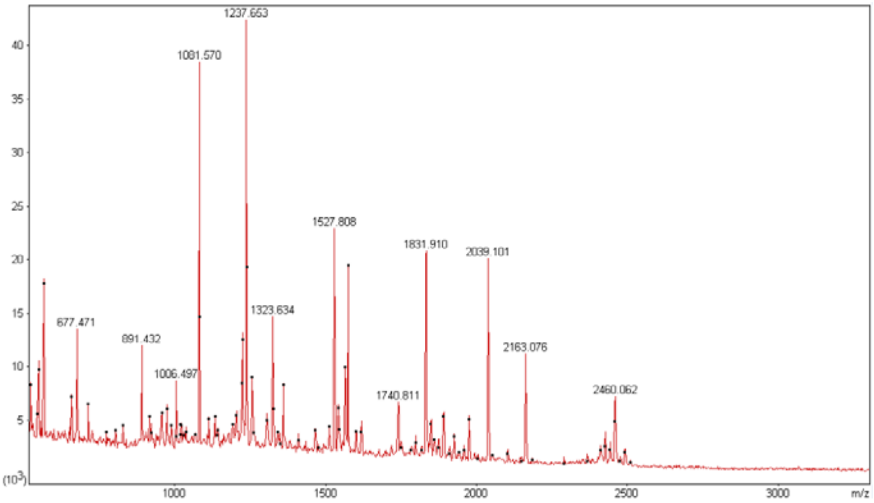

Probability Based Mowse Score:

Protein score is -10*Log(P), where P is the probability that the observed match is a random event.
Protein scores greater than 61 are significant (p<0.05).

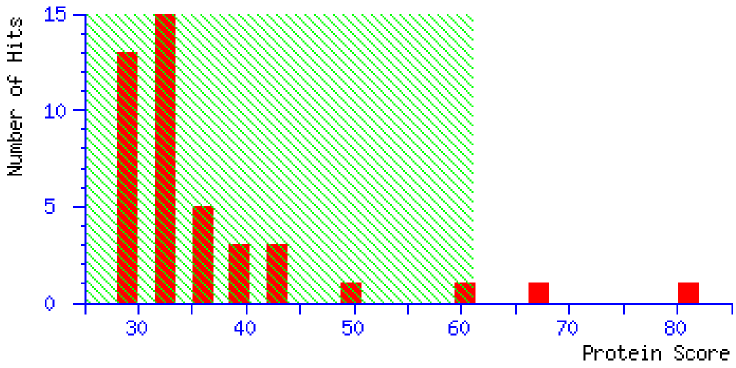

Spot No.: **30**

NCBI accession No.: **cassava4.1_002466m|PACid:17978799**

Plant species: **Manihot esculenta**

Protein name: **Alpha-glucan phosphorylase 1**

PFF **Mascot** score: **[387]** Sequence coverage %: **[12]**

Matched peptides No.: **[7]** p value: 3.5e-008

Calculated Mr: 81918 Calculated *p*I: **8.50**

Annotated PMF **spectra:**

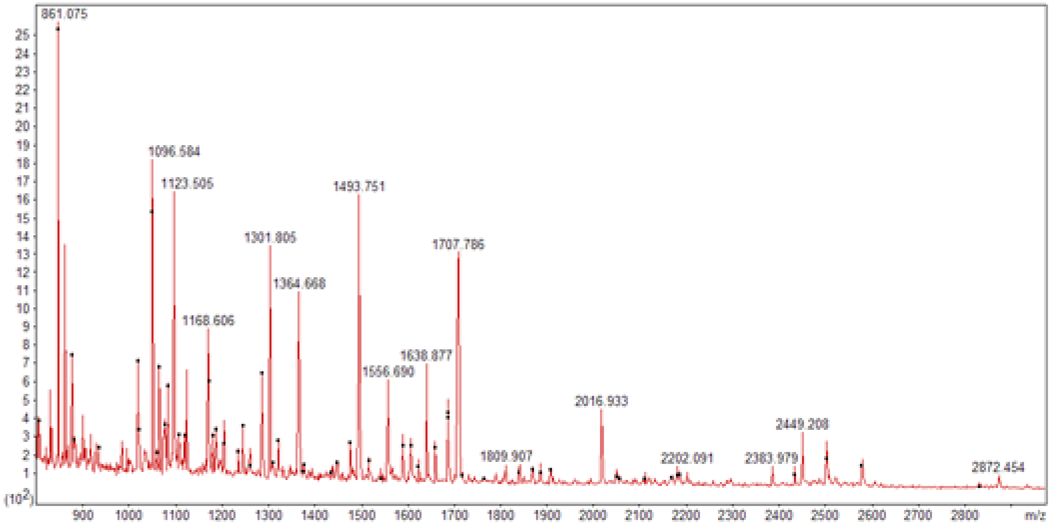

PFF Searched Score:

Ions score is -10*Log(P), where P is the probability that the observed match is a random event.
Individual ions scores > 33 indicate identity or extensive homology (p<0.05).
Protein scores are derived from ions scores as a non-probabilistic basis for ranking protein hits.

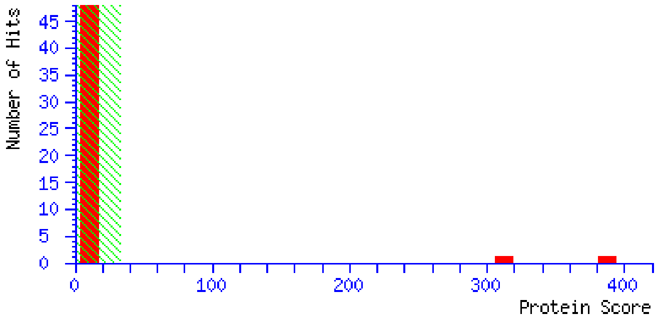

Spot No.: **31**

NCBI accession No.: **cassava4.1_002614m|PACid:17978880**

Plant species: ***Manihot esculenta***

Protein name： **Alpha-glucan phosphorylase 1**

Mascot score: **76** p value: **0.019**

Sequence coverage %: **32**

Matched peptides No.: **16** Total peptides No.: **58**

Calculated Mr: **80611** Calculated *p*I: **6.71**

Annotated PMF **spectra:**

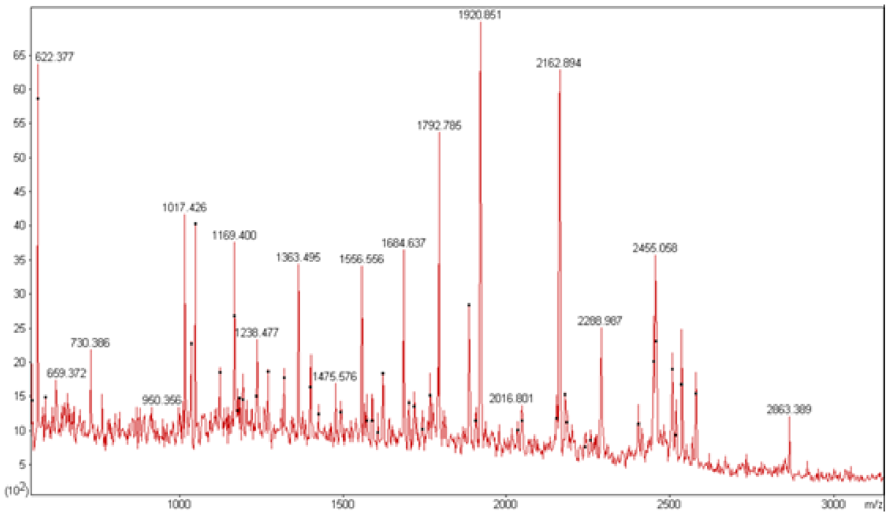

Probability Based Mowse Score:

Protein score is -10*Log(P), where P is the probability that the observed match is a random event.
Protein scores greater than 61 are significant (p<0.05).

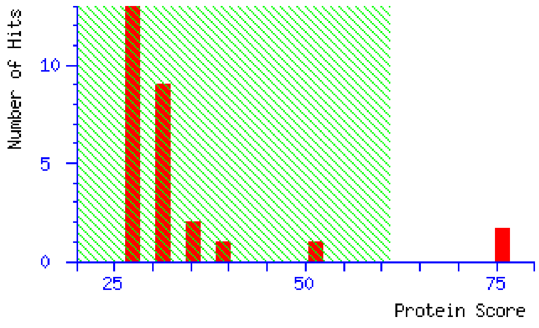

Spot No.: **32**

NCBI accession No.: **cassava4.1_002466m|PACid:17978799**

Plant species: ***Manihot esculenta***

Protein name： **Alpha-glucan phosphorylase 1**

Sequence coverage %: **[2]** Matched peptides No.: **[5]**

PFF **Mascot** score: **[110]** p value: **6.8e-005**

Calculated Mr: **81918** Calculated *p*I: **8.50**

Annotated PMF **spectra:**

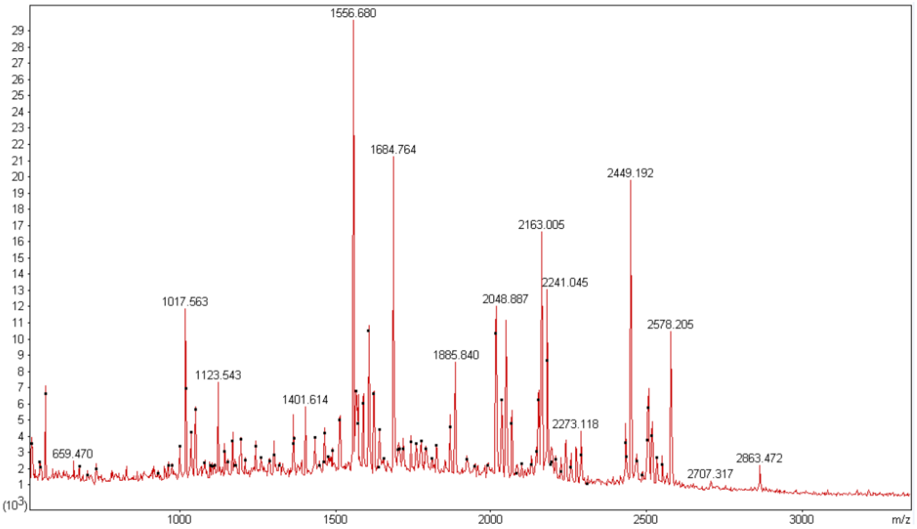

PFF Searched Score:

Ions score is -10*Log(P), where P is the probability that the observed match is a random event.
Individual ions scores > 33 indicate identity or extensive homology (p<0.05).
Protein scores are derived from ions scores as a non-probabilistic basis for ranking protein hits.

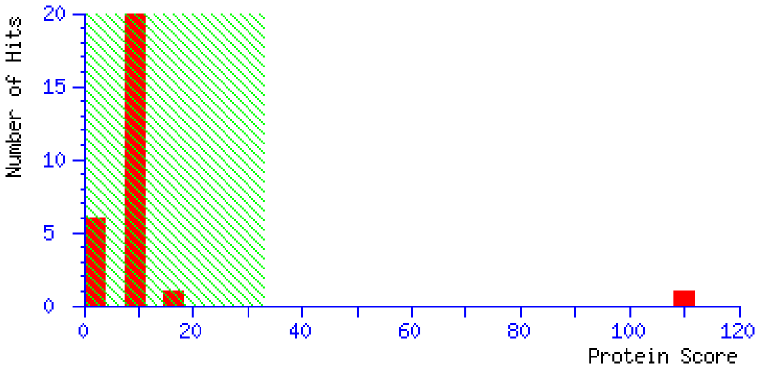

Spot No.: **33**

NCBI accession No.: cassava4.1_002466m|PACid:17978799

Plant species: ***Manihot esculenta***

Protein name： **Alpha-glucan phosphorylase 1**

Mascot score: **101**

Sequence coverage %: **40** p value: 5.8e-006

Matched peptides No.: **24** Total peptides No.: **98**

Calculated Mr: **81918** Calculated *p*I: **8.50**

Annotated PMF **spectra:**

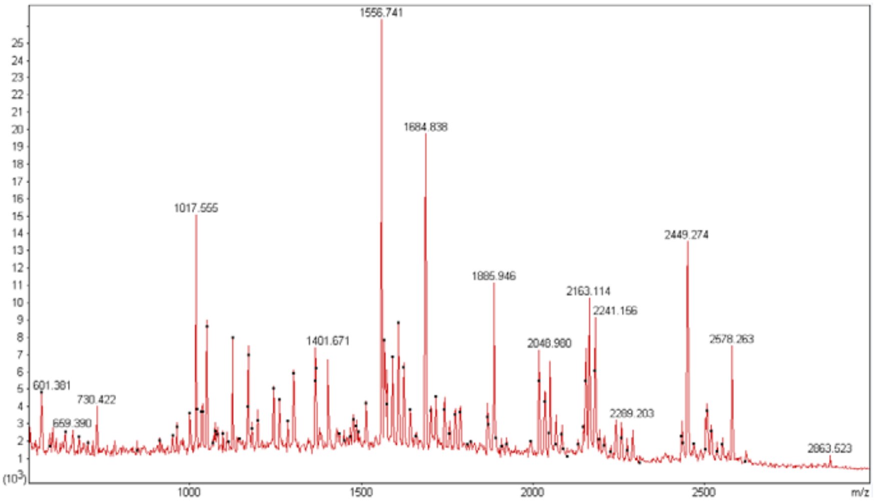

Probability Based Mowse Score:

Protein score is -10*Log(P), where P is the probability that the observed match is a random event. Protein scores greater than 61 are significant (p<0.05).

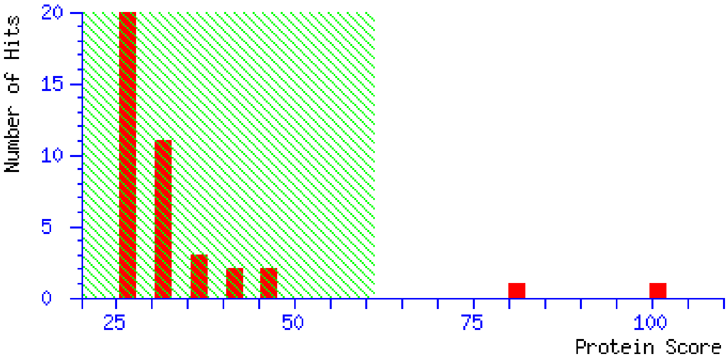

Spot No.: **34**

NCBI accession No.: **cassava4.1_014251m|PACid:17989805**

Plant species: ***Manihot esculenta***

Protein name：**14-3-3-like protein GF14 omega**

Mascot score: **115**

Sequence coverage %: **44** p value: **2.3e-007**

Matched peptides No.: **16** Total peptides No.: **46**

Calculated Mr: **29447** Calculated *p*I: **4.77**

Annotated PMF **spectra:**

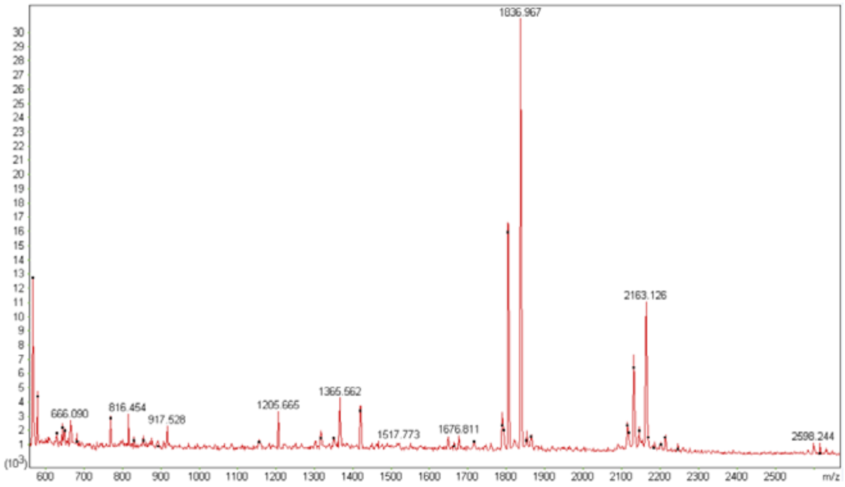

Probability Based Mowse Score:

Protein score is -10*Log(P), where P is the probability that the observed match is a random event.
Protein scores greater than 61 are significant (p<0.05).

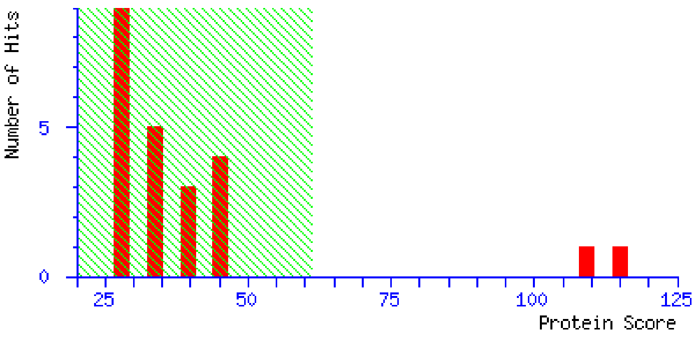

Spot No.: **35**

NCBI accession No.: **cassava4.1_014208m|PACid:17964823**

Plant species: **Manihot esculenta**

Protein name： **14-3-3-like protein GF14 omega**

Mascot score: **159** p value: **9.2e-012**

Sequence coverage %: **54**

Matched peptides No.: **21** Total peptides No.: **56**

Calculated Mr: **29548** Calculated *p*I: **4.76**

Annotated PMF **spectra:**

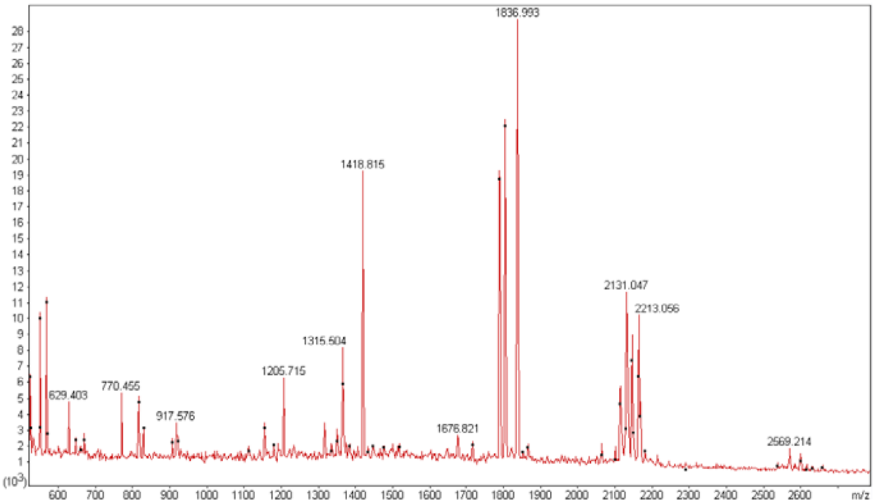

Probability Based Mowse Score:

Protein score is -10*Log(P), where P is the probability that the observed match is a random event.
Protein scores greater than 61 are significant (p<0.05).

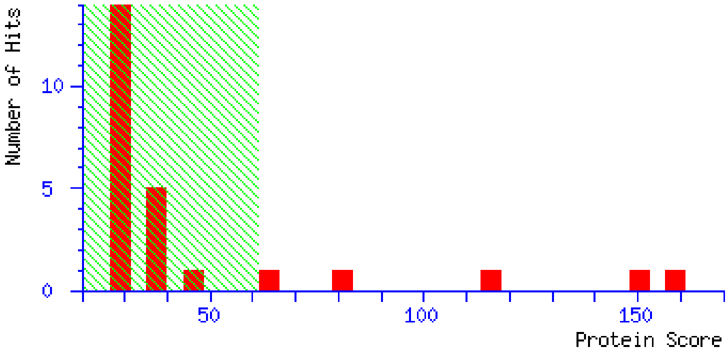

Spot No.: **36**

NCBI accession No.: **cassava4.1_014065m|PACid:17989625**

Plant species: ***Manihot esculenta***

Protein name：**14-3-3-like protein GF14 psi** Mascot score: **111**

Sequence coverage %: **60** p value: **5.8e-07**

Matched peptides No.: **16** Total peptides No.: **67**

Calculated Mr: **29927** Calculated *p*I: **4.75**

Annotated PMF **spectra:**

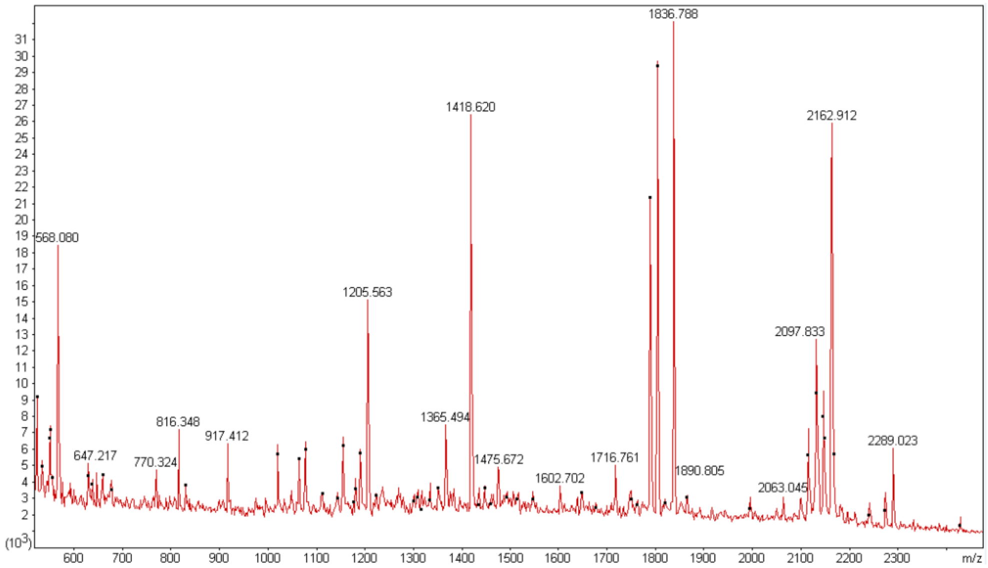

Probability Based Mowse Score:

Protein score is -10*Log(P), where P is the probability that the observed match is a random event. Protein scores greater than 61 are significant (p<0.05).

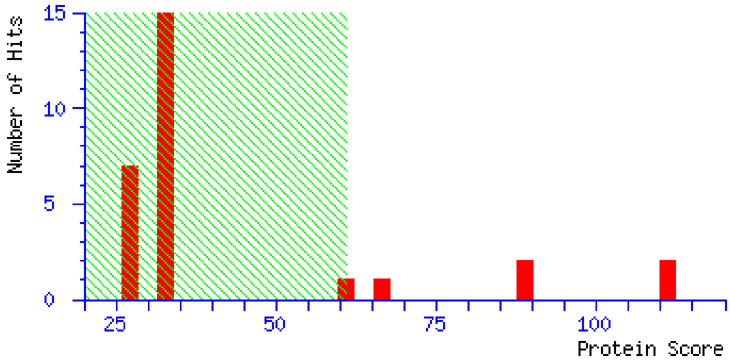

Spot No.: **37**

NCBI accession No.: **cassava4.1_011584m|PACid:17982732**

Protein name：**Probable fructokinase-1**

Plant species: ***Manihot esculenta***

PFF Mascot score: [154] Sequence coverage %: [7]

Matched peptides No.: [**2**] p value: **6.9e-007**

Calculated Mr: **35621** Calculated *p*I: **5.12**

Annotated PMF **spectra:**

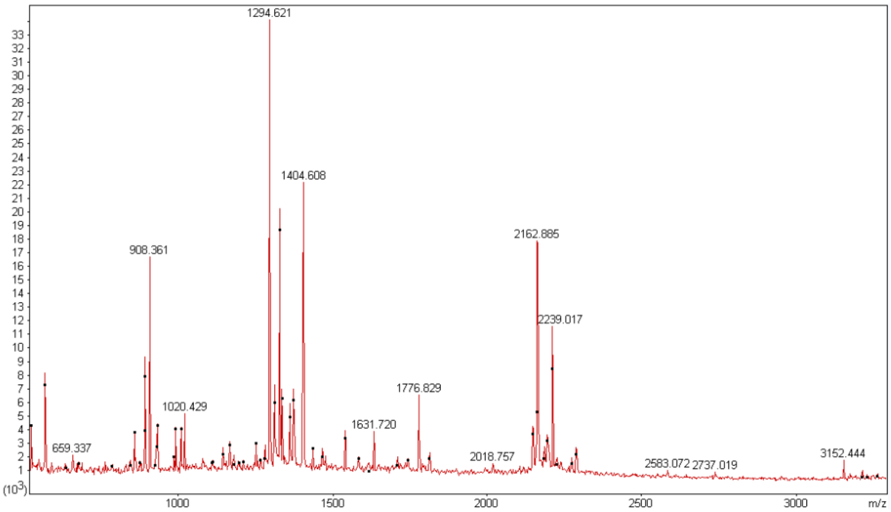

PFF Searched Score:

Ions score is -10*Log(P), where P is the probability that the observed match is a random event.
Individual ions scores > 34 indicate identity or extensive homology (p<0.05).
Protein scores are derived from ions scores as a non-probabilistic basis for ranking protein hits.

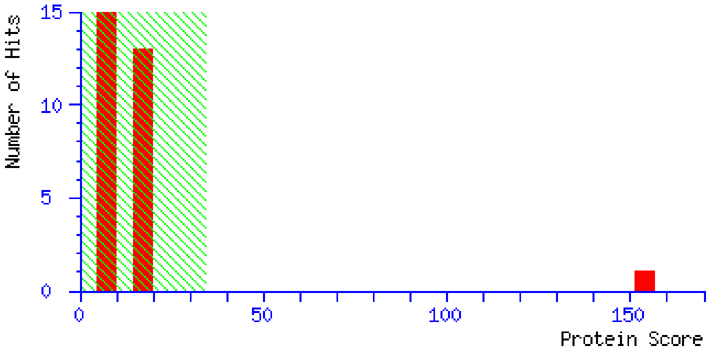

Spot No.: **38**

NCBI accession No.: **cassava4.1_011584m|PACid:17982732**

Plant species: ***Manihot esculenta***

Protein name：**Probable fructokinase-1**

PFF Mascot score: [147] **Sequence coverage %: [11]**

Matched peptides No.: **[3]** p value: **9.6e-006**

Calculated Mr: **35621** Calculated *p*I: **5.12**

Annotated PMF **spectra:**

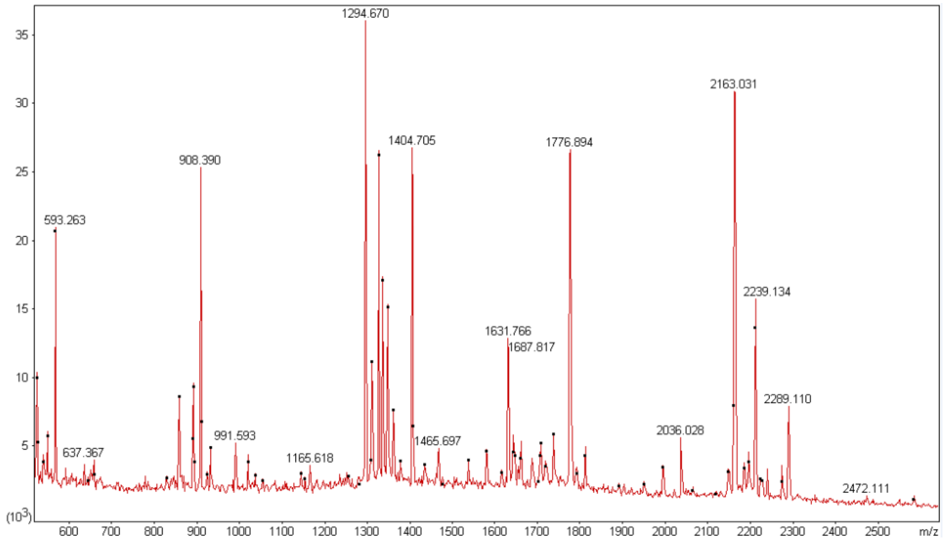

PFF Searched Score:

Ions score is -10*Log(P), where P is the probability that the observed match is a random event.
Individual ions scores > 34 indicate identity or extensive homology (p<0.05).
Protein scores are derived from ions scores as a non-probabilistic basis for ranking protein hits.

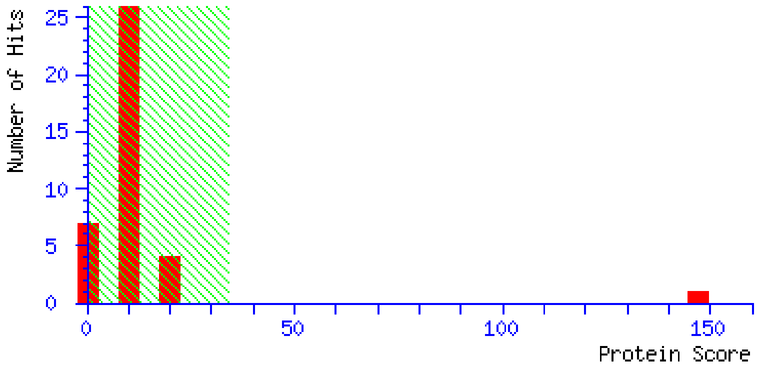

Spot No.: **39**

NCBI accession No.: **cassava4.1_013158m|PACid:17968853** p value: **0.0017**

Plant species: ***Manihot esculenta***

Protein name：**Probable lactoylglutathione lyase, chloroplast**

Mascot score: **151**

Sequence coverage %: **55** p value: **5.8e-011**

Matched peptides No.: **19** Total peptides No.: **77**

Calculated Mr: **32759** Calculated *p*I: **5.17**

Annotated PMF **spectra:**

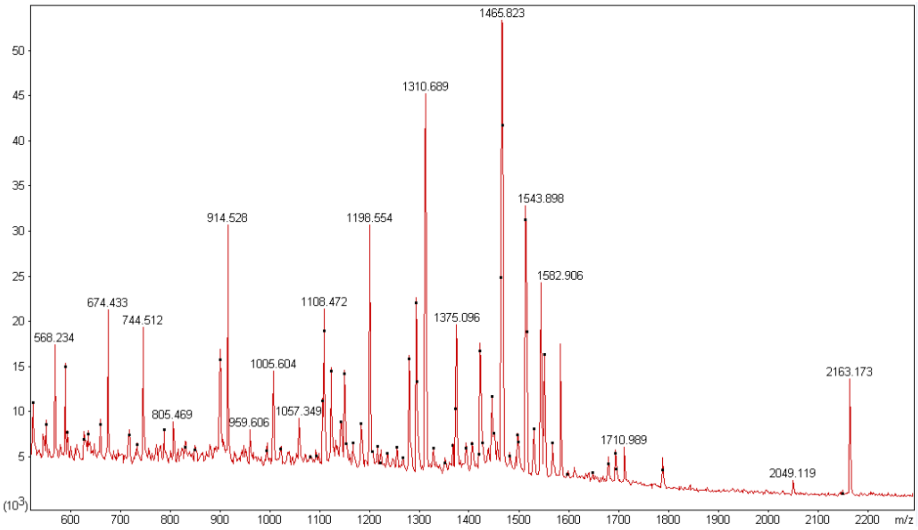

Probability Based Mowse Score:

Protein score is -10*Log(P), where P is the probability that the observed match is a random event.
Protein scores greater than 61 are significant (p<0.05).

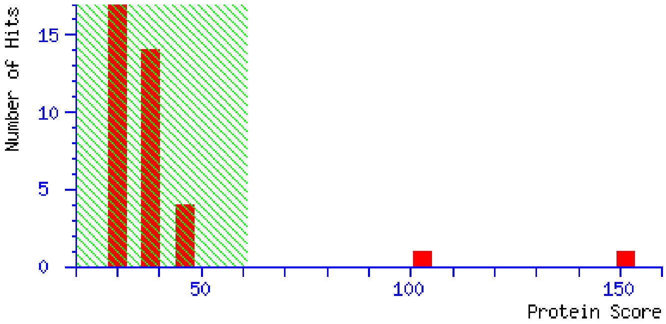

Spot No.: **40**

NCBI accession No.: **cassava4.1_013158m|PACid:17968853**

Plant species: ***Manihot esculenta***

Protein name：**Probable lactoylglutathione lyase, chloroplast**

PFF Mascot score: **[73]** Sequence coverage %: **[8]**

Matched peptides No.: **[2]** p value: 6.1e-006

Calculated Mr: **32759** Calculated *p*I: **5.17**

Annotated PMF **spectra:**

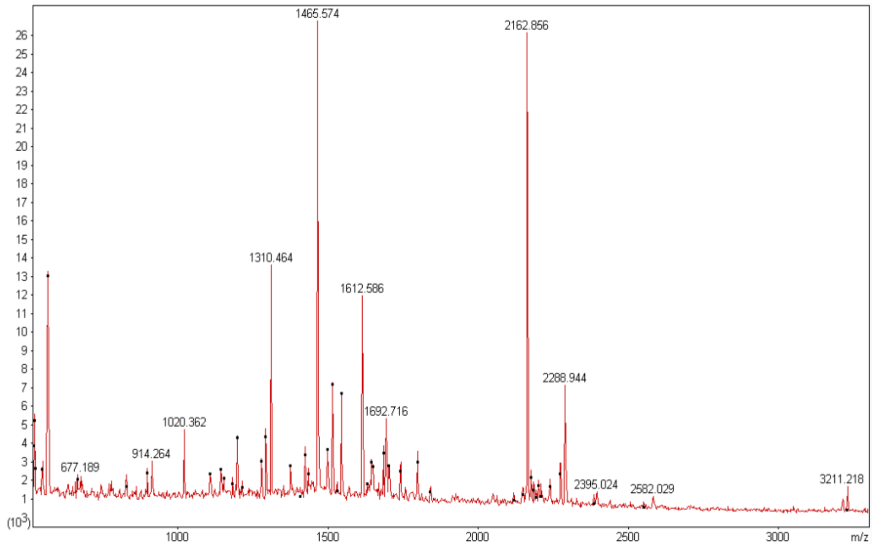

PFF Searched Score:

Ions score is -10*Log(P), where P is the probability that the observed match is a random event.
Individual ions scores > 33 indicate identity or extensive homology (p<0.05).
Protein scores are derived from ions scores as a non-probabilistic basis for ranking protein hits.

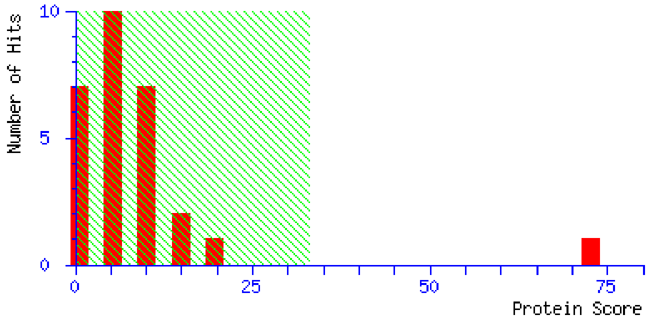

Spot No.: **41**

NCBI accession No.: **cassava4.1_012601m|PACid:17970211**

Plant species: ***Manihot esculenta***

Protein name：**Putative carboxyphosphonoenolpyruvate mutase**

PFF Mascot score: [187] **Sequence coverage %: [10]**

Matched peptides No.: **[2]** p value: **1.2e-009**

Calculated Mr: **32938** Calculated *p*I: **5.32**

Annotated PMF **spectra:**

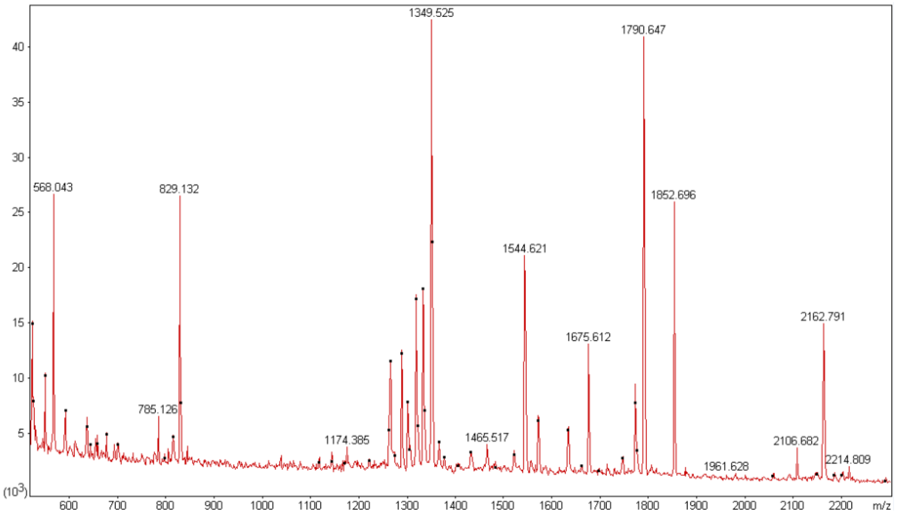

PFF Searched Score:

Ions score is -10*Log(P), where P is the probability that the observed match is a random event.
Individual ions scores > 33 indicate identity or extensive homology (p<0.05).
Protein scores are derived from ions scores as a non-probabilistic basis for ranking protein hits.

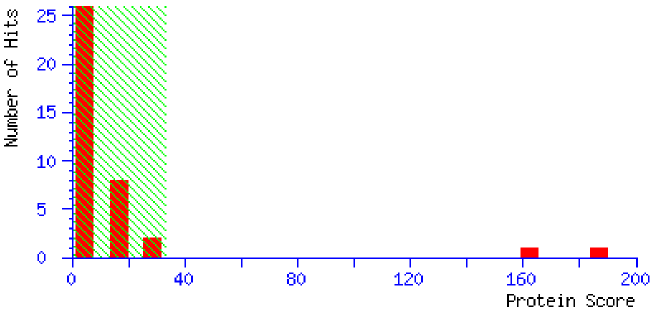

Spot No.: **42**

NCBI accession No.: **cassava4.1_011578m|PACid:17975802**

Plant species: ***Manihot esculenta***

Protein name：**Probable fructokinase-1**

PFF Mascot score: **[146]** Sequence coverage %: **[11]**

Matched peptides No.: **[3]** p value: **0.00019**

Calculated Mr: **35491** Calculated *p*I: **5.29**

Annotated PMF **spectra:**

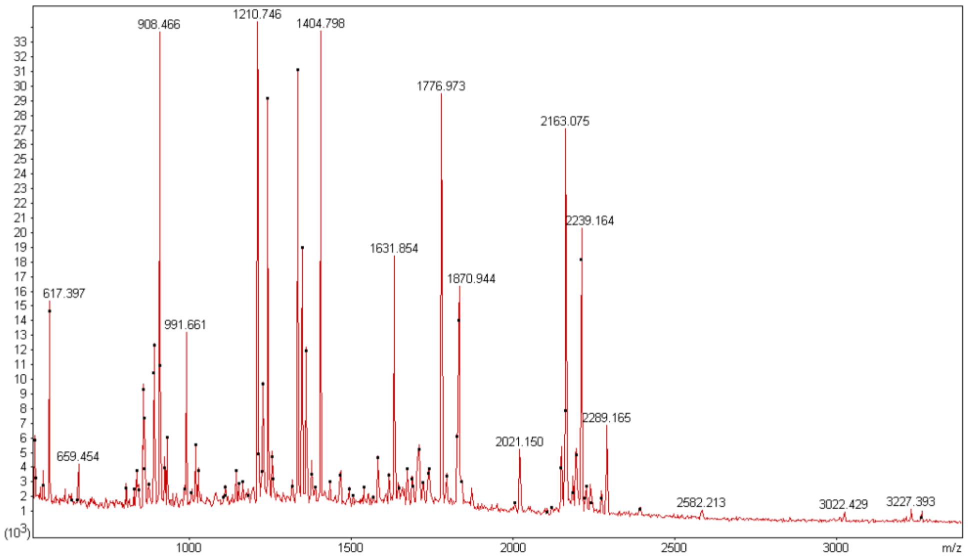

PFF Searched Score:

Ions score is -10*Log(P), where P is the probability that the observed match is a random event.
Individual ions scores > 33 indicate identity or extensive homology (p<0.05).
Protein scores are derived from ions scores as a non-probabilistic basis for ranking protein hits.

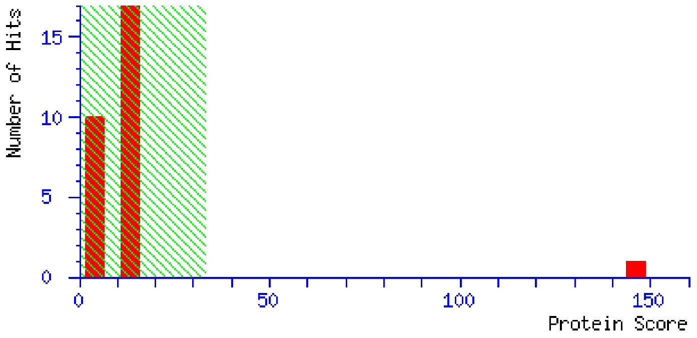

Spot No.: **43**

NCBI accession No.: **cassava4.1_010863m|PACid:17977552**

Plant species: **Manihot esculenta**

Protein name: NADP-dependent alkenal double bond reductase P1

PFF Mascot score: **[81]** Sequence coverage %: **[7]**

Matched peptides No.: **[2]** p value: **3.6e-006**

Calculated Mr: **38818** Calculated *p*I: **5.41**

Annotated PMF **spectra:**

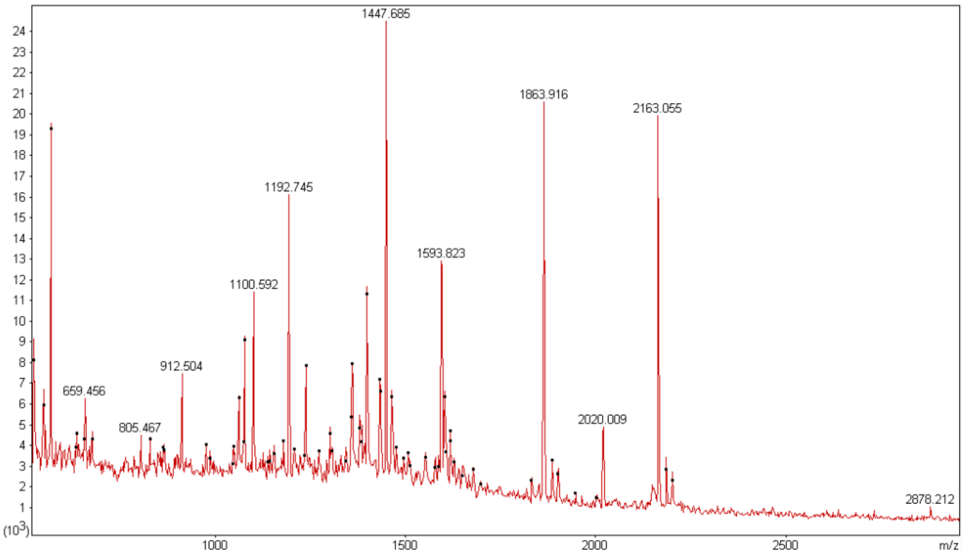

PFF Searched Score:

Ions score is -10*Log(P), where P is the probability that the observed match is a random event.
Individual ions scores > 33 indicate identity or extensive homology (p<0.05).
Protein scores are derived from ions scores as a non-probabilistic basis for ranking protein hits.

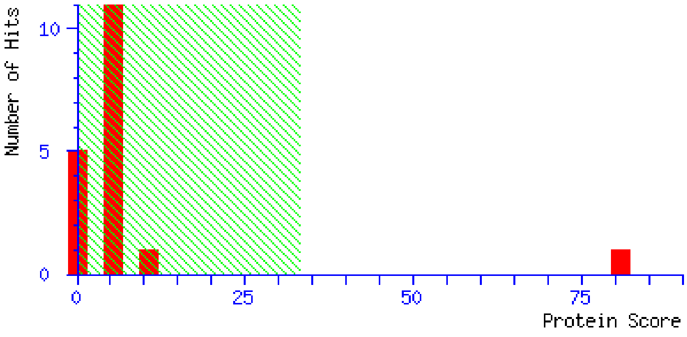

Spot No.: **44**

NCBI accession No.: **cassava4.1_010863m|PACid:17977552**

Plant species: ***Manihot esculenta***

Protein name：**NADP-dependent alkenal double bond reductase**

PFF Mascot score: **[99]**　　Sequence coverage %: **[7]**

Matched peptides No.: **[2]**　　p value: **7.3e-008**

Calculated Mr: **38818**　　Calculated *p*I: **5.41**

Annotated PMF **spectra:**

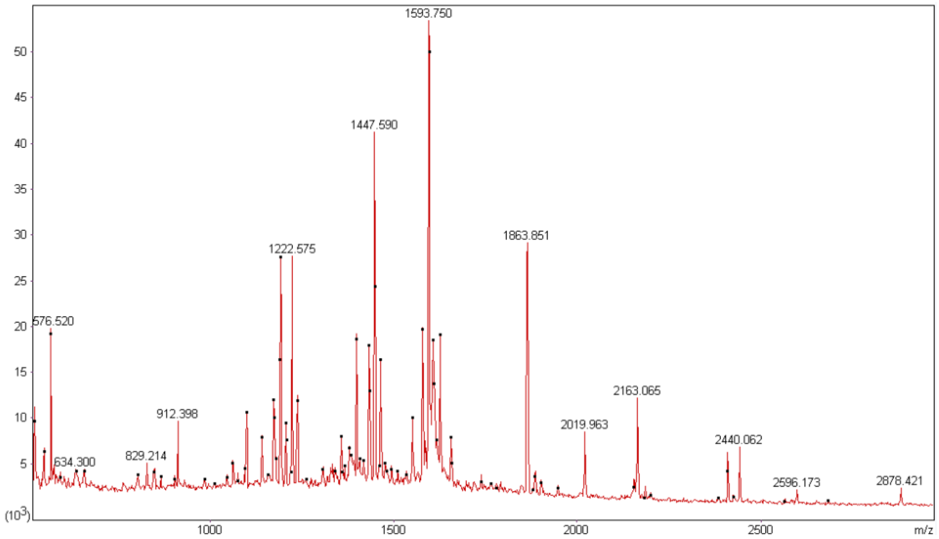

PFF Searched Score:

Ions score is -10*Log(P), where P is the probability that the observed match is a random event.
Individual ions scores > 33 indicate identity or extensive homology (p<0.05).
Protein scores are derived from ions scores as a non-probabilistic basis for ranking protein hits.

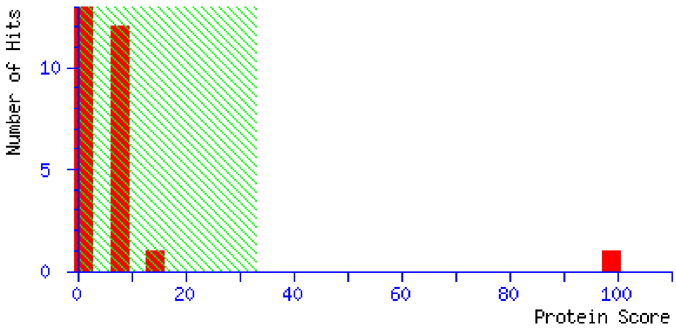

Spot No.: **45**

NCBI accession No.: **cassava4.1_011869m|PACid:17965954**

Plant species: ***Manihot esculenta***

Protein name：**DNA-damage-repair/toleration protein DRT102**

PFF Mascot score: **[151]** Sequence coverage %: **[8]**

Matched peptides No.: **[2]** p value: **1.6e-008**

Calculated Mr: 35467 Calculated *p*I: **5.12**

Annotated PMF **spectra:**

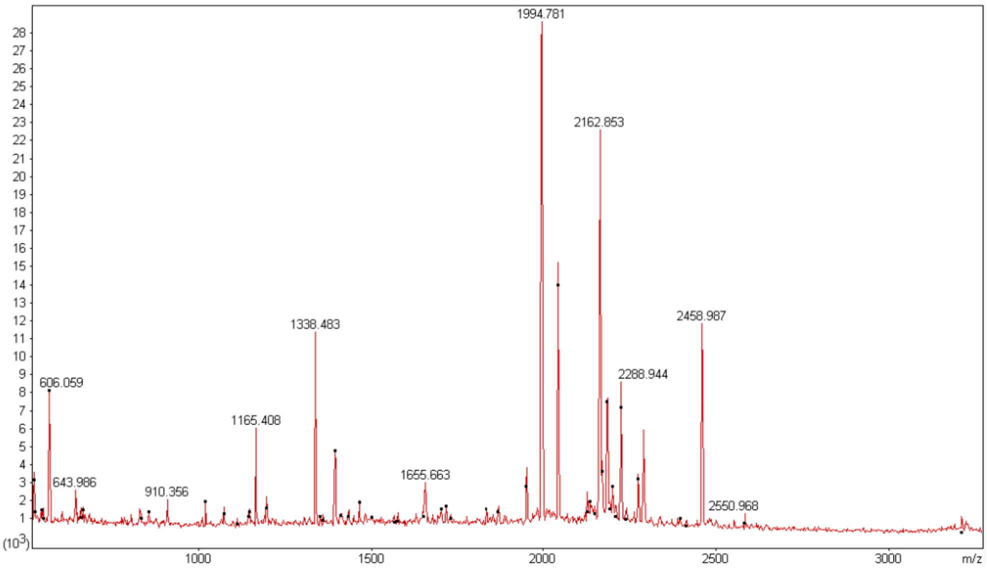

PFF Searched Score:

Ions score is -10*Log(P), where P is the probability that the observed match is a random event.
Individual ions scores > 33 indicate identity or extensive homology (p<0.05).
Protein scores are derived from ions scores as a non-probabilistic basis for ranking protein hits.

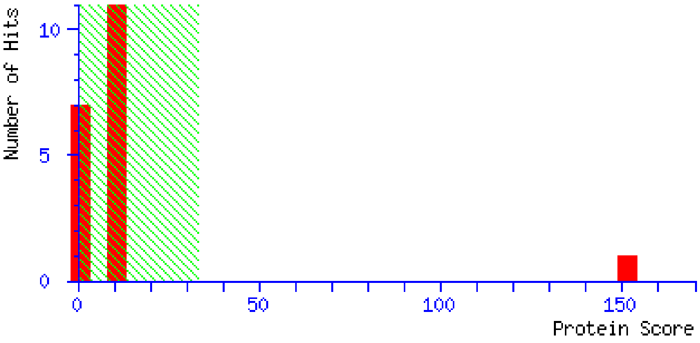

Spot No.: **46**

NCBI accession No.: **cassava4.1_012448m|PACid:17968446** Plant species: ***Manihot esculenta*** Protein name: **Putative NAD(P)H oxidoreductase, isoflavone reductase**

PFF Mascot score: **[254]** Sequence coverage %: **[15]**

Matched peptides No.: **[3]** p value: **4.7e-012**

Calculated Mr: 33873 Calculated *p*I: **5.47**

Annotated PMF **spectra:**

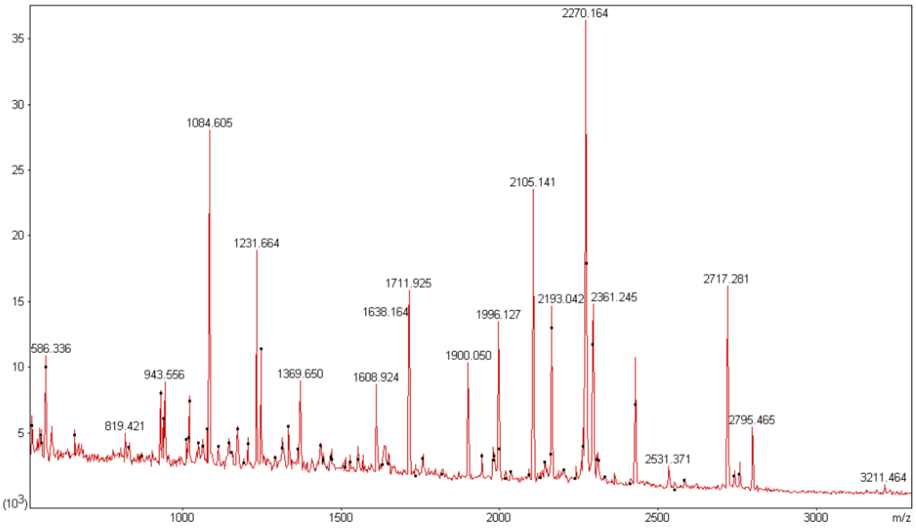

PFF Searched Score:

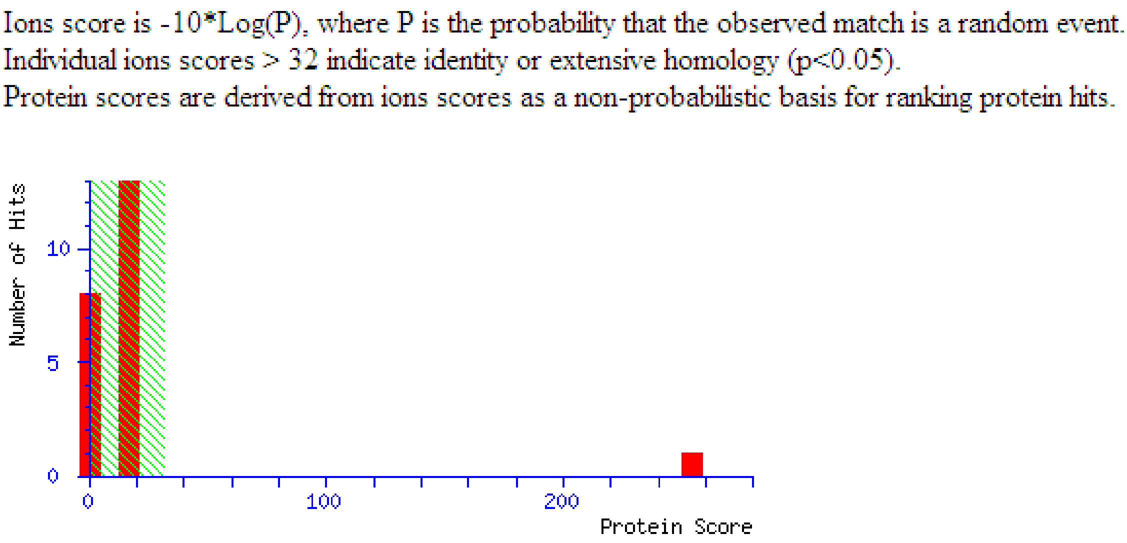

Spot No.: **47**

NCBI accession No.: **cassava4.1_010620m|PACid:17987783**

Plant species: ***Manihot esculenta***

Protein name: **Thiamine thiazole synthase, chloroplastic**

PFF Mascot score: [133] **Sequence coverage %: [8]**

Matched peptides No.: **[2]** p value: **6.7e-006**

Calculated Mr: **37625** Calculated *p*I: **6.07**

Annotated PMF **spectra:**

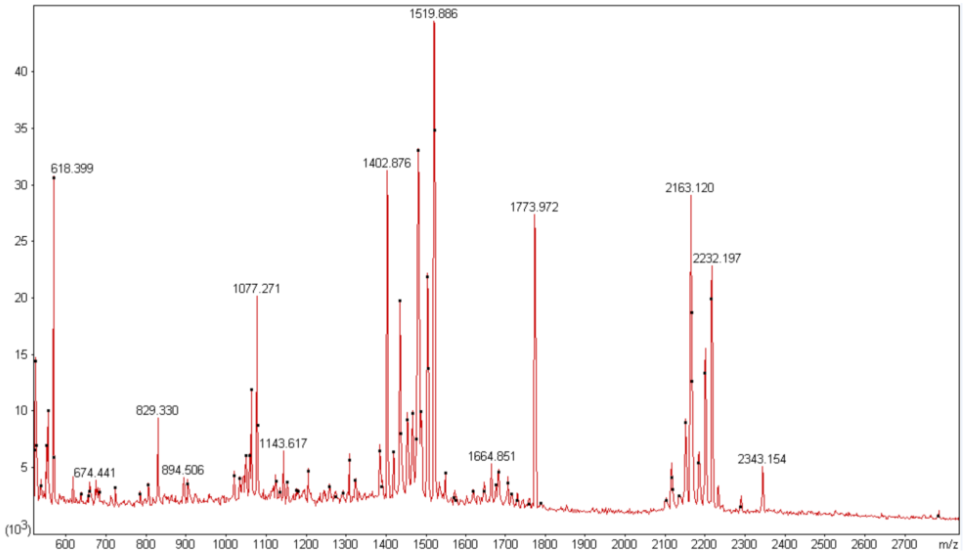

PFF Searched Score:

Ions score is -10*Log(P), where P is the probability that the observed match is a random event.
Individual ions scores > 33 indicate identity or extensive homology (p<0.05).
Protein scores are derived from ions scores as a non-probabilistic basis for ranking protein hits.

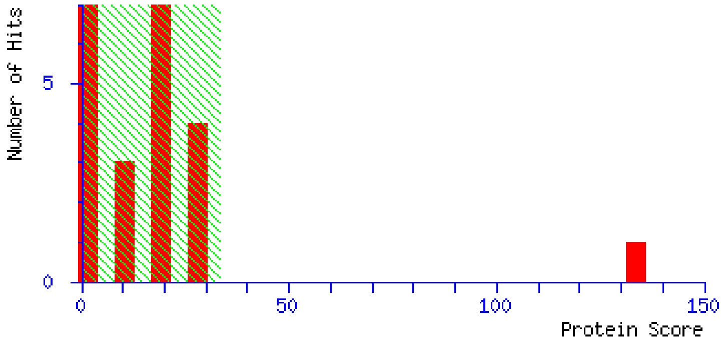

Spot No.: **48**

NCBI accession No.: **cassava4.1_013135m|PACid:17983192**

Plant species: ***Manihot esculenta***

Protein name: **Alpha-soluble NSF attachment protein 2**

PFF Mascot score: [209] **Sequence coverage %: [12]**

Matched peptides No.: **[3]** p value: **9.1e-007**

Calculated Mr: **32780** Calculated *p*I: **5.13**

Annotated PMF **spectra:**

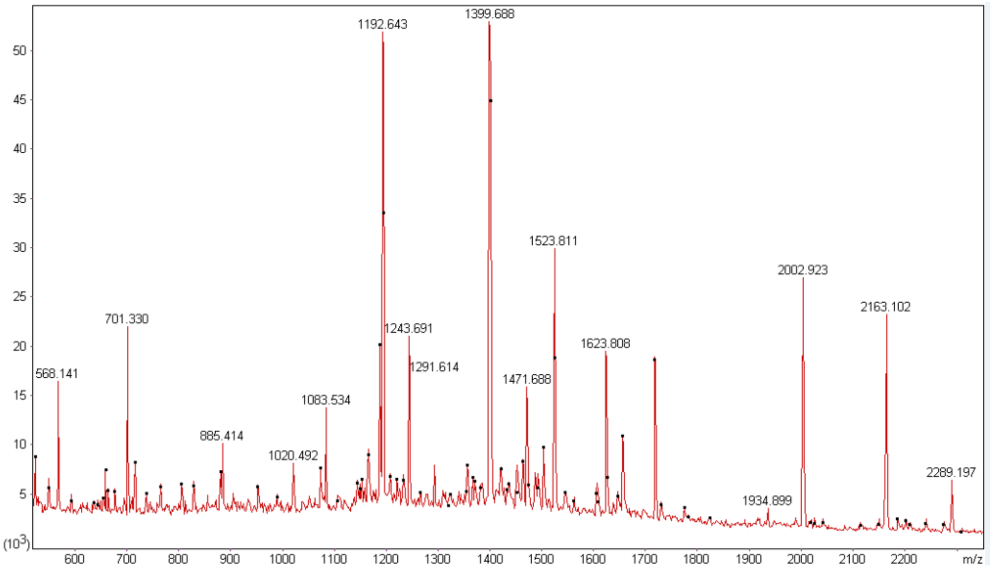

PFF Searched Score:

Ions score is -10*Log(P), where P is the probability that the observed match is a random event.
Individual ions scores > 34 indicate identity or extensive homology (p<0.05).
Protein scores are derived from ions scores as a non-probabilistic basis for ranking protein hits.

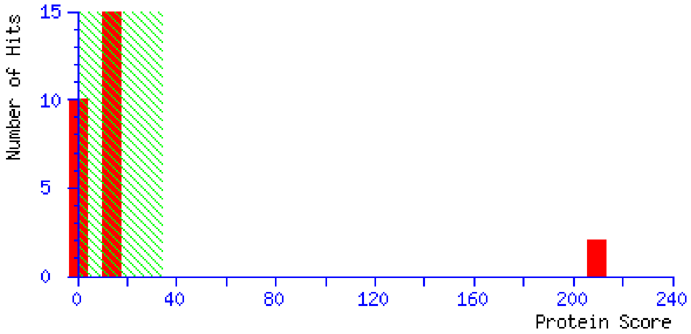

Spot No.: **49**

NCBI accession No.: **cassava4.1_014185m|PACid:17964106**

Plant species: ***Manihot esculenta***

Protein name: **Ferritin-4, chloroplastic**

Mascot score: **125**

Sequence coverage %: **61** p value: **2.3e-008**

Matched peptides No.: **18** Total peptides No.: **68**

Calculated Mr: **29470** Calculated *p*I: **5.30**

Annotated PMF **spectra:**

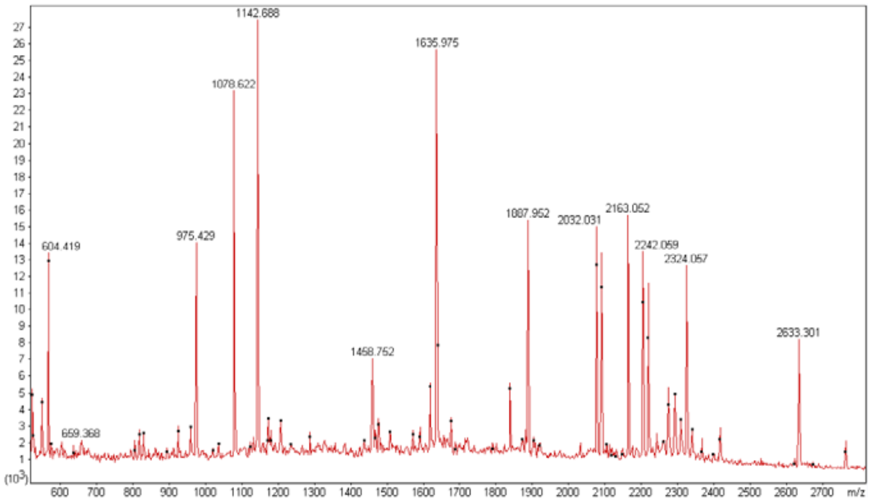

Probability Based Mowse Score:

Protein score is -10*Log(P), where P is the probability that the observed match is a random event. Protein scores greater than 61 are significant (p<0.05).

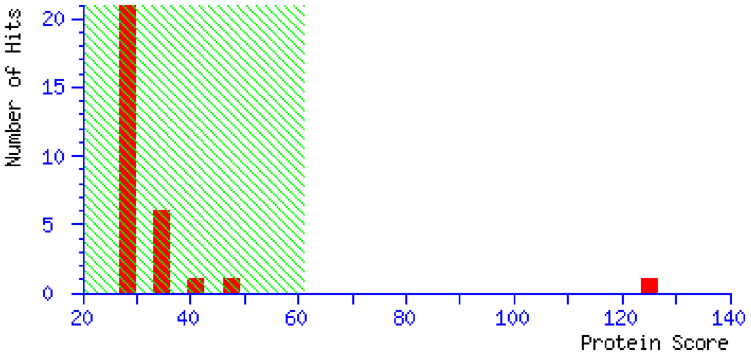

Spot No.: **50**

NCBI accession No.: **cassava4.1_014410m|PACid:17965277**

Plant species: ***Manihot esculenta***

Protein name： **20 kDa chaperonin, chloroplastic**

PFF Mascot score: **[172]** Sequence coverage %: **[21]**

Matched peptides No.: **[3]** p value: **5e-005**

Calculated Mr: **26695** Calculated *p*I: **7.79**

Annotated PMF **spectra:**

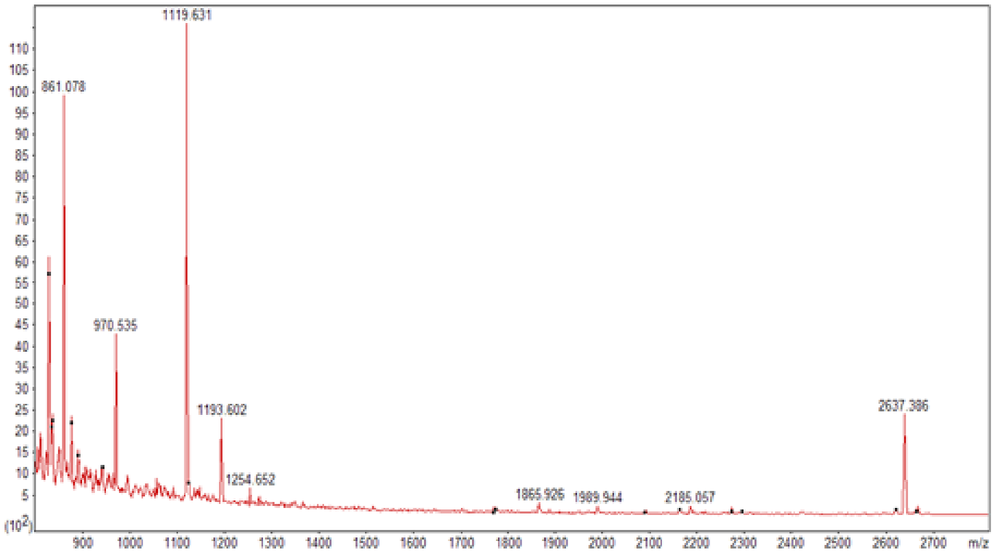

PFF Searched Score:

Ions score is -10*Log(P), where P is the probability that the observed match is a random event.
Individual ions scores > 33 indicate identity or extensive homology (p<0.05).
Protein scores are derived from ions scores as a non-probabilistic basis for ranking protein hits.

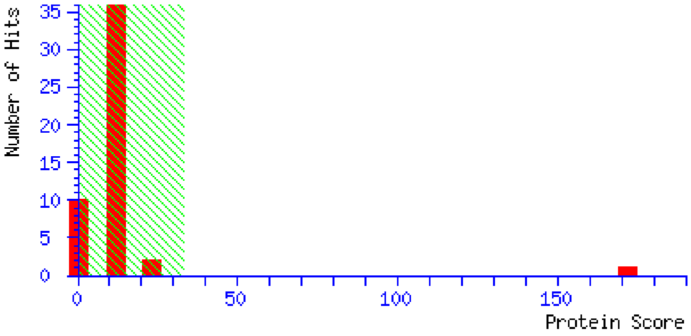

Spot No.: **51**

NCBI accession No.: **cassava4.1_014643m|PACid:17991662**

Plant species: ***Manihot esculenta***

Protein name：**L-ascorbate peroxidase 1, cytosolic**

Mascot score: **82** p value: **0.00046**

Sequence coverage %: **40** Matched peptides No.: **13**

Calculated Mr: **27766** Calculated *p*I: **5.31**

Annotated PMF **spectra:**

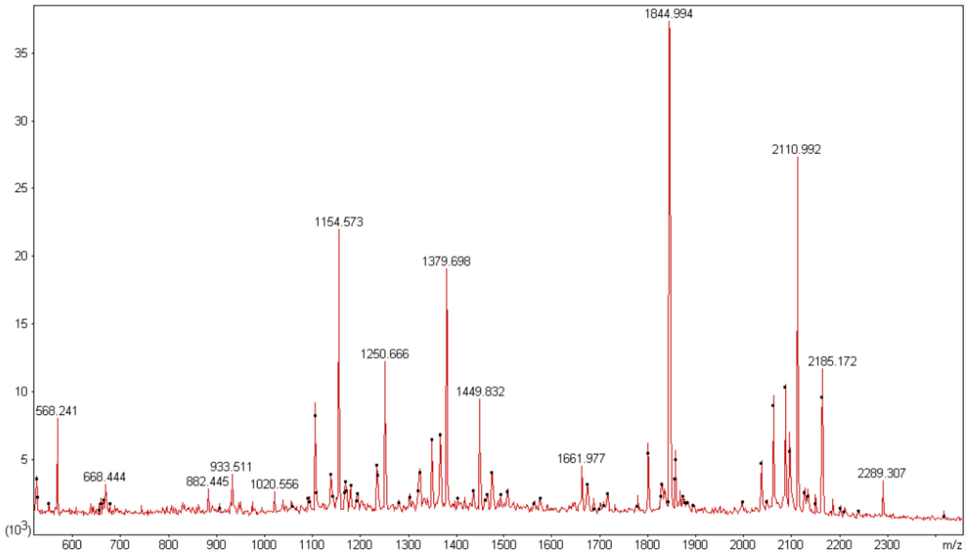

Probability Based Mowse Score:

Protein score is -10*Log(P), where P is the probability that the observed match is a random event. Protein scores greater than 61 are significant (p<0.05).

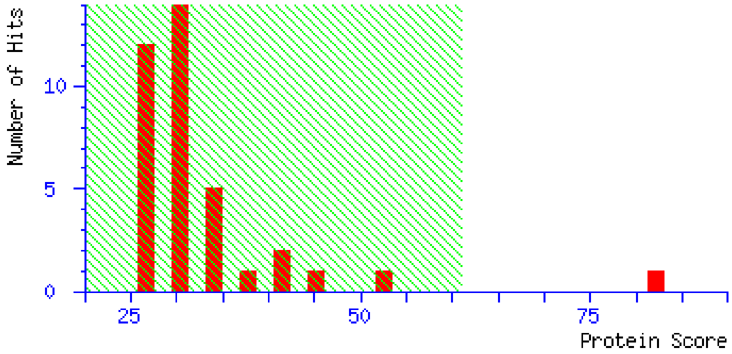

Spot No.: **52**

NCBI accession No.: **cassava4.1_014643m|PACid:17991662**

Plant species: ***Manihot esculenta***

Protein name：**L-ascorbate peroxidase 1, cytosolic**

Mascot score: **133**　　　p value: **3.7e-009**

Sequence coverage %: **50**

Matched peptides No.: **17**　　　Total peptides No.: **52**

Calculated Mr: **27766**　　　Calculated *p*I: **5.31**

Annotated PMF **spectra:**

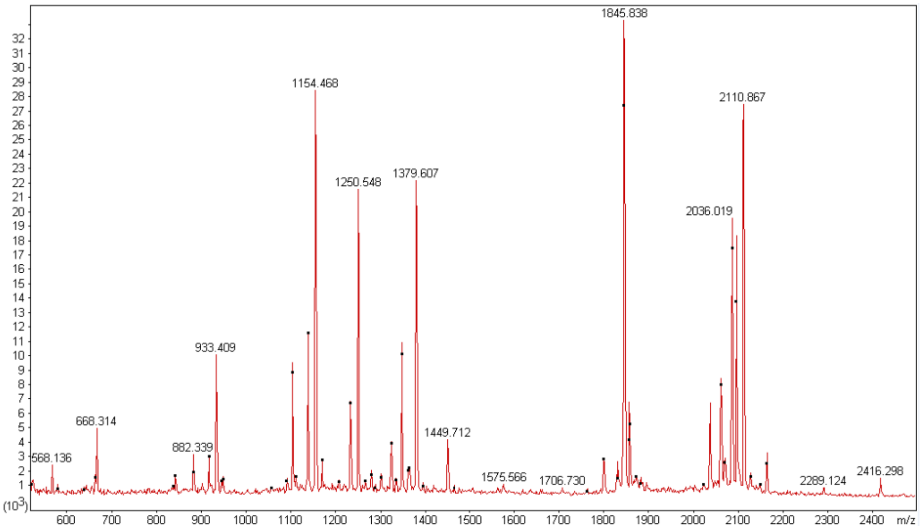

Probability Based Mowse Score:

Protein score is -10*Log(P), where P is the probability that the observed match is a random event.
Protein scores greater than 61 are significant (p<0.05).

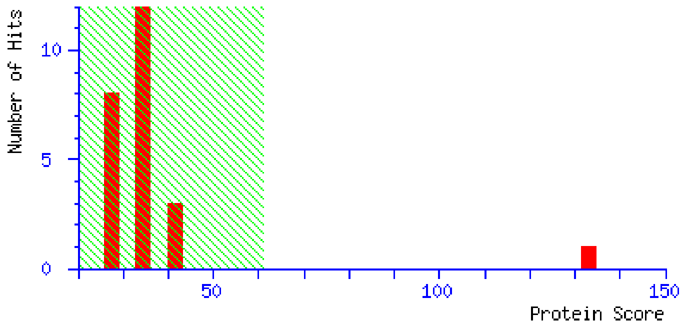

Spot No.: **53**

NCBI accession No.: **cassava4.1_009779m|PACid:17990964**

Plant species: ***Manihot esculenta***

Protein name: **Actin-7**

PFF Mascot score: [96] **Sequence coverage %:** [7]

Matched peptides No.: [**2**] p value: **0.00065**

Calculated Mr: 41897 Calculated *p*I: **5.31**

Annotated PMF **spectra:**

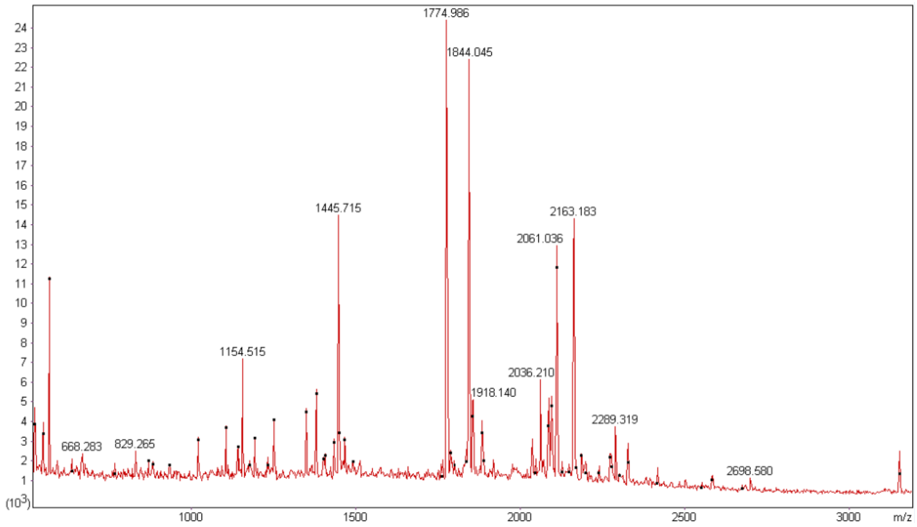

PFF Searched Score:

Ions score is -10*Log(P), where P is the probability that the observed match is a random event.
Individual ions scores > 33 indicate identity or extensive homology (p<0.05).
Protein scores are derived from ions scores as a non-probabilistic basis for ranking protein hits.

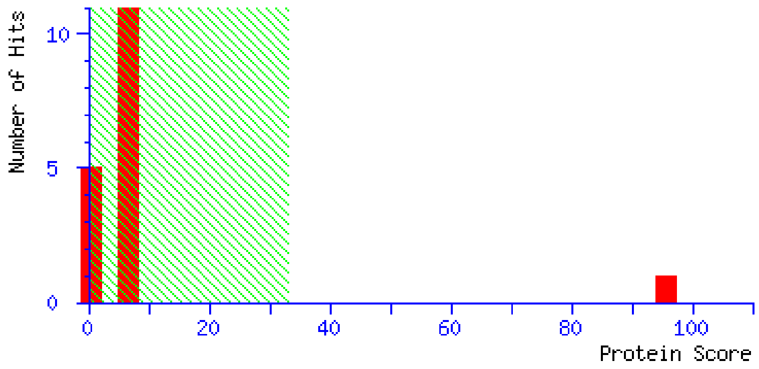

Spot No.: **54**

NCBI accession No.: **cassava4.1_017771m|PACid:17966880**

Plant species: ***Manihot esculenta***

Protein name: **ATP synthase subunit d, mitochondrial**

Mascot score: **113** p value: **3.7e-007**

Sequence coverage %: **57**

Matched peptides No.: **13** Total peptides No.: **39**

Calculated Mr: **19663** Calculated *p*I: **5.11**

Annotated PMF **spectra:**

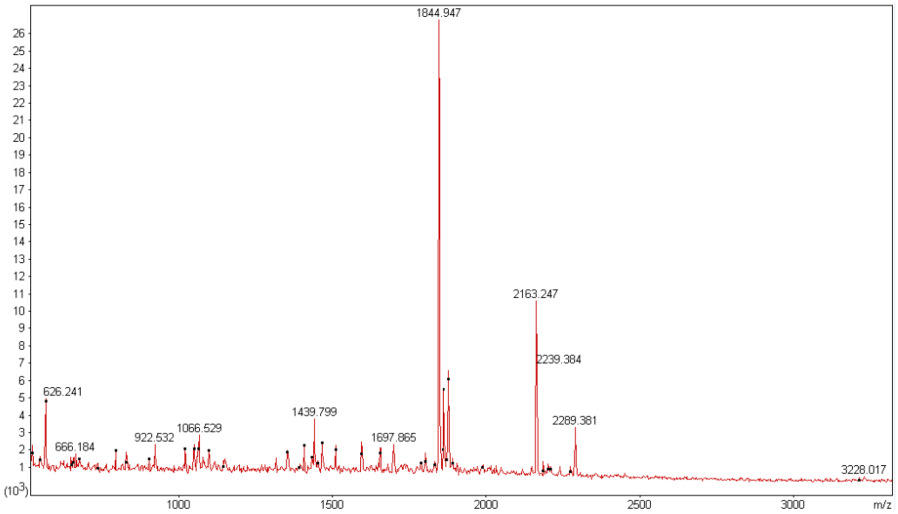

Probability Based Mowse Score:

Protein score is -10*Log(P), where P is the probability that the observed match is a random event. Protein scores greater than 61 are significant (p<0.05).

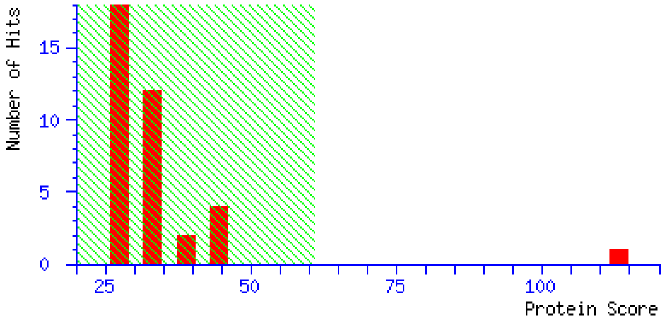

Spot No.: **55**

NCBI accession No.: **cassava4.1_017771m|PACid:17966880**

Plant species: ***Manihot esculenta***

Protein name: **ATP synthase subunit d, mitochondrial**

Mascot score: **184** p value: **2.9e-014**

Sequence coverage %: **70**

Matched peptides No.: 19 Total peptides No.: **50**

Calculated Mr: **19663** Calculated *p*I: **5.11**

Annotated PMF **spectra:**

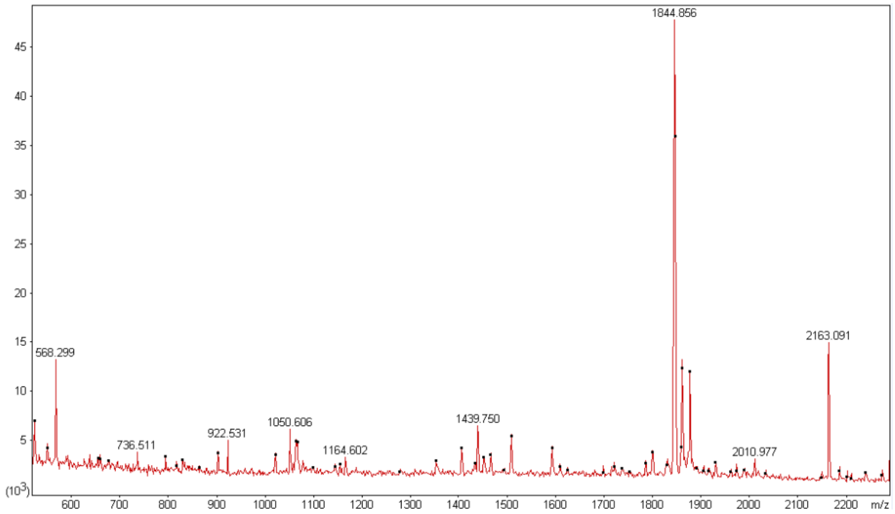

Probability Based Mowse Score:

Protein score is -10*Log(P), where P is the probability that the observed match is a random event.
Protein scores greater than 61 are significant (p<0.05).

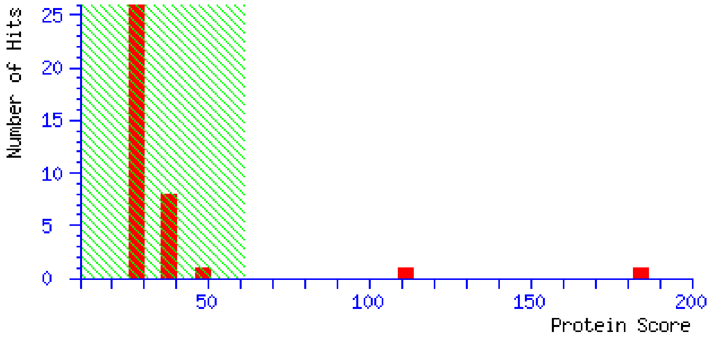

Spot No.: **56**

NCBI accession No.: **cassava4.1_015349m|PACid:17959817**

Plant species: ***Manihot esculenta***

Protein name： **Proteasome subunit beta type**

PFF Mascot score: [137] **Sequence coverage %:** [11]

Matched peptides No.: **[2]** p value: **9.5e-006**

Calculated Mr: **24988** Calculated *p*I: **5.51**

Annotated PMF **spectra:**

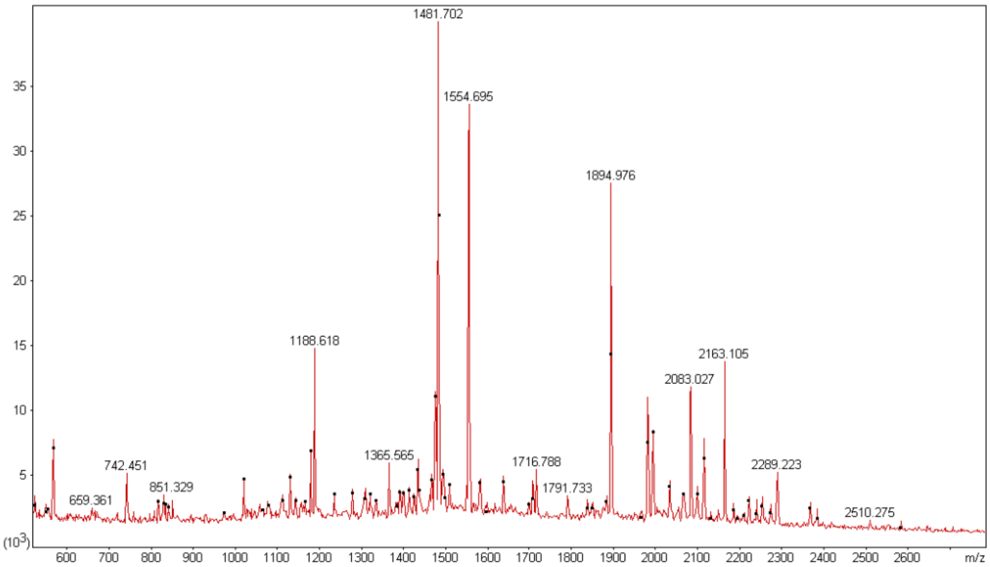

PFF Searched Score:

Ions score is -10*Log(P), where P is the probability that the observed match is a random event.
Individual ions scores > 33 indicate identity or extensive homology (p<0.05).
Protein scores are derived from ions scores as a non-probabilistic basis for ranking protein hits.

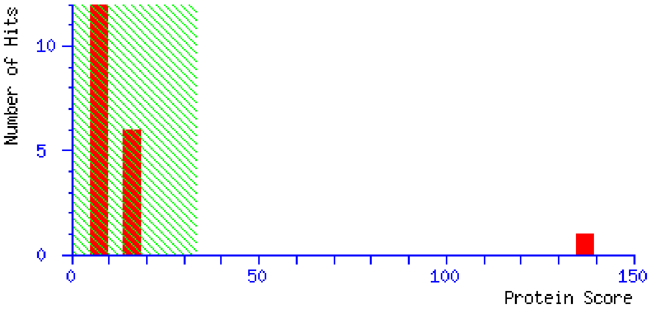

Spot No.: **57**

NCBI accession No.: **cassava4.1_017832m|PACid:17961591**

Plant species: ***Manihot esculenta***

Protein name：**regulator of ribonuclease activity a**

PFF Mascot score: [133] **Sequence coverage %: [16]**

Matched peptides No.: **[2]** p value: **8.1e-007**

Calculated Mr: 18015 Calculated *p*I: **5.39**

Annotated PMF **spectra:**

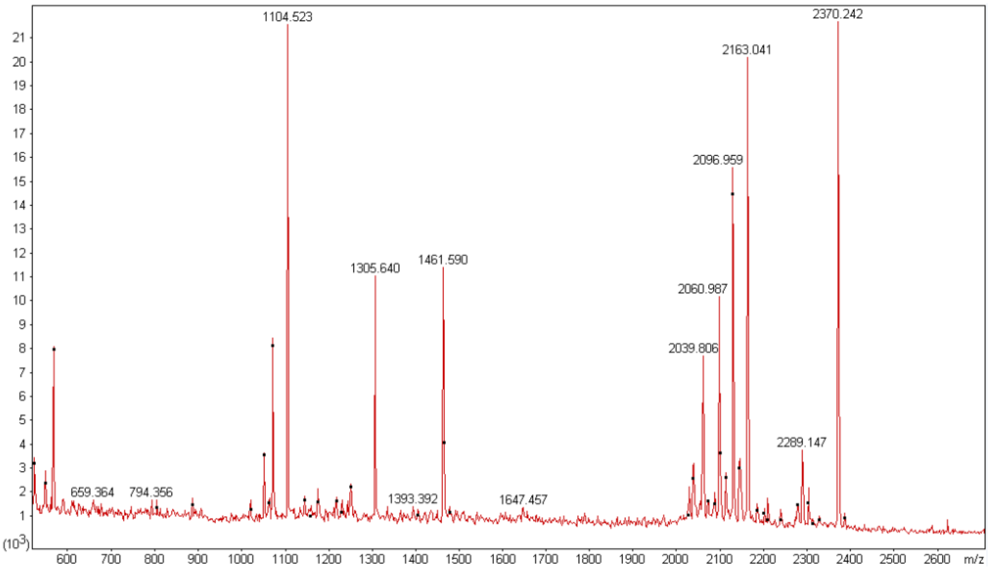

PFF Searched Score:

Ions score is -10*Log(P), where P is the probability that the observed match is a random event.
Individual ions scores > 33 indicate identity or extensive homology (p<0.05).
Protein scores are derived from ions scores as a non-probabilistic basis for ranking protein hits.

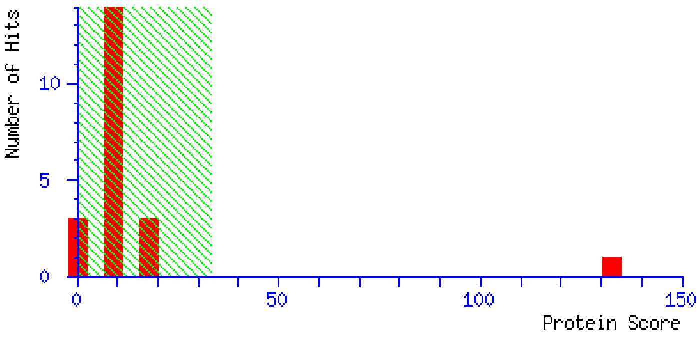

Spot No.: **58**

NCBI accession No.: **cassava4.1_018059m|PACid:17963195**

Plant species: ***Manihot esculenta***

Protein name： **Eukaryotic translation initiation factor 5A-2**

PFF Mascot score: [66] **Sequence coverage %:** [**18]**

Matched peptides No.: [**2]** p value: **0.00026**

Calculated Mr: **17706** Calculated *p*I: **5.60**

Annotated PMF **spectra:**

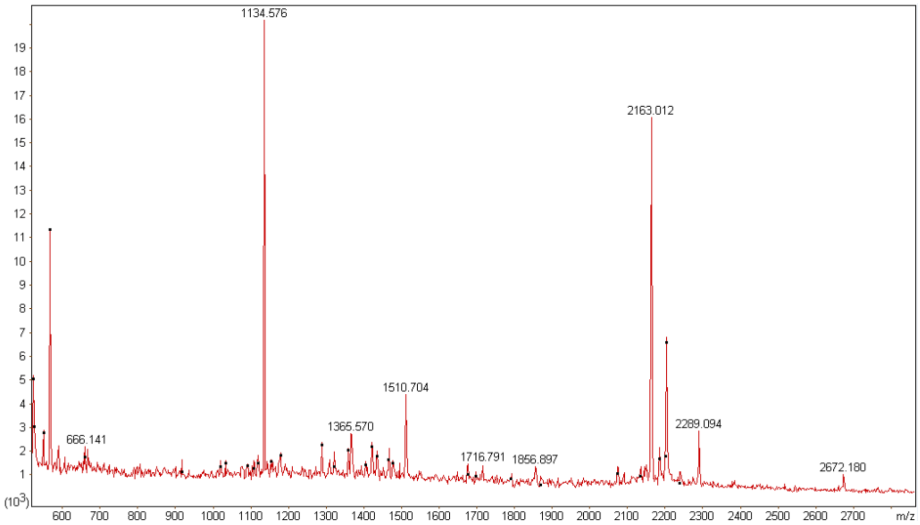

PFF Searched Score:

Ions score is -10*Log(P), where P is the probability that the observed match is a random event.
Individual ions scores > 33 indicate identity or extensive homology (p<0.05).
Protein scores are derived from ions scores as a non-probabilistic basis for ranking protein hits.

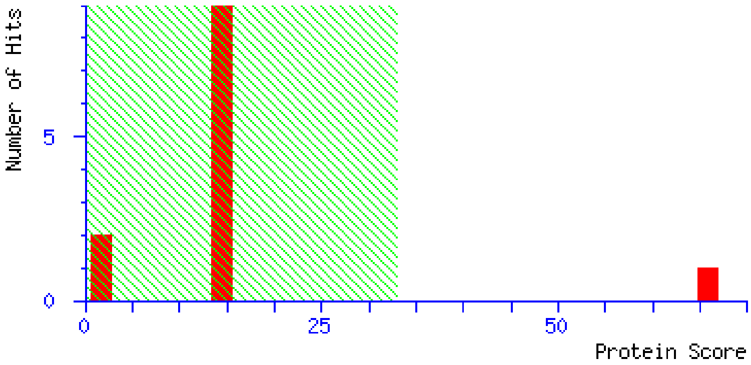

Spot No.: **59**

NCBI accession No.: **cassava4.1_018026m|PACid:17990511**

Plant species: ***Manihot esculenta***

Protein name： **Eukaryotic translation initiation factor 5A-2**

Mascot score: **72**   p value: **0.041**

Sequence coverage %: **44**

Matched peptides No.: **7**   Total peptides No.: **33**

Calculated Mr: **17805**   Calculated *p*I: **5.60**

Annotated PMF **spectra:**

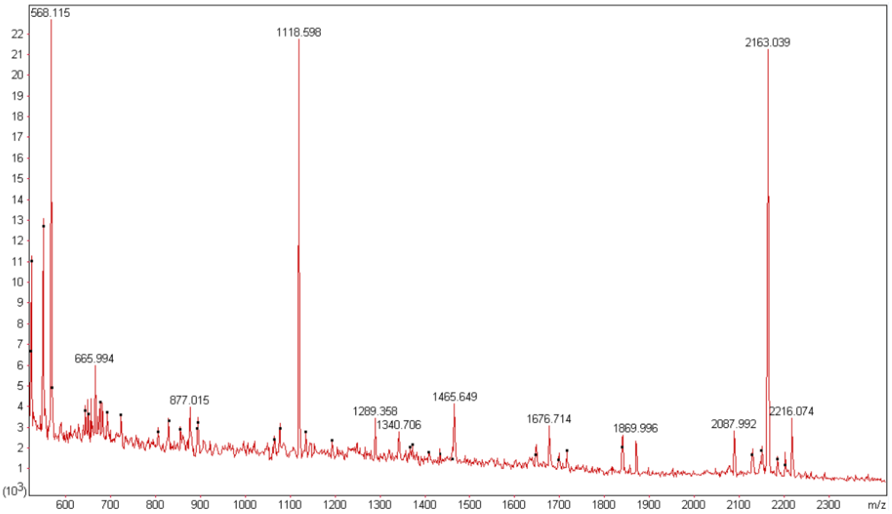

Probability Based Mowse Score:

Protein score is -10*Log(P), where P is the probability that the observed match is a random event.
Protein scores greater than 61 are significant (p<0.05).

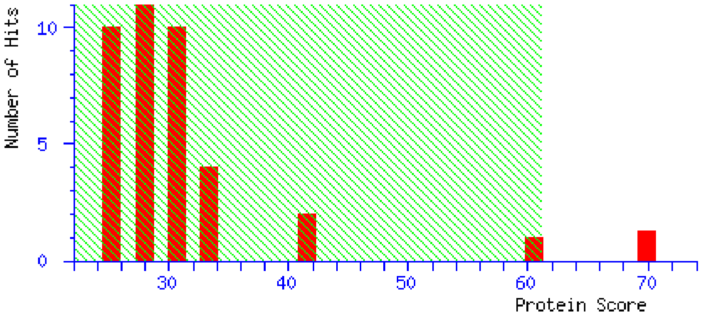

Spot No.: **60**

NCBI accession No.: **cassava4.1_018158m|PACid:17970560**

Plant species: ***Manihot esculenta***

Protein name： **heat-shock protein, putative**

PFF Mascot score: [196] **Sequence coverage %:** [**19]**

Matched peptides No.: [**2]** p value: **4.4e-011**

Calculated Mr: **17672** Calculated *p*I: **5.78**

Annotated PMF **spectra:**

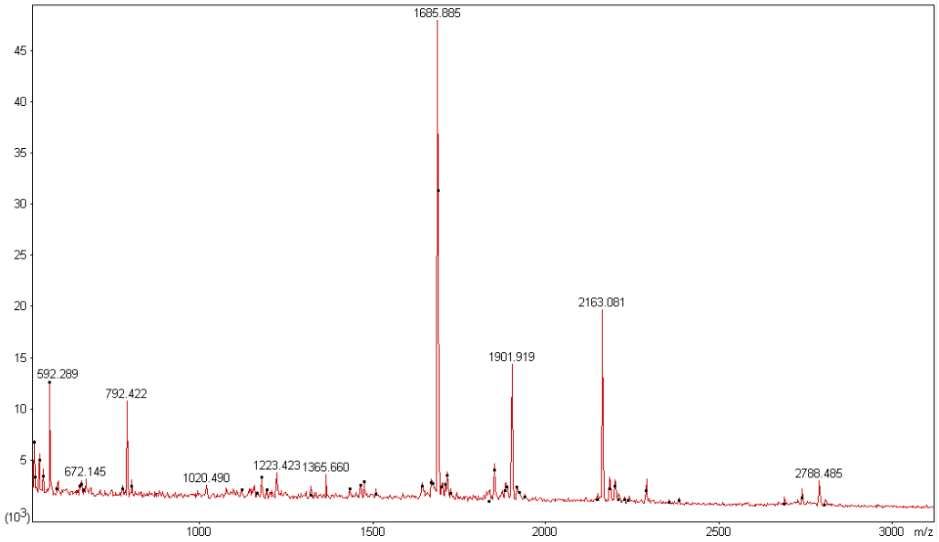

PFF Searched Score:

Ions score is -10*Log(P), where P is the probability that the observed match is a random event.
Individual ions scores > 32 indicate identity or extensive homology (p<0.05).
Protein scores are derived from ions scores as a non-probabilistic basis for ranking protein hits.

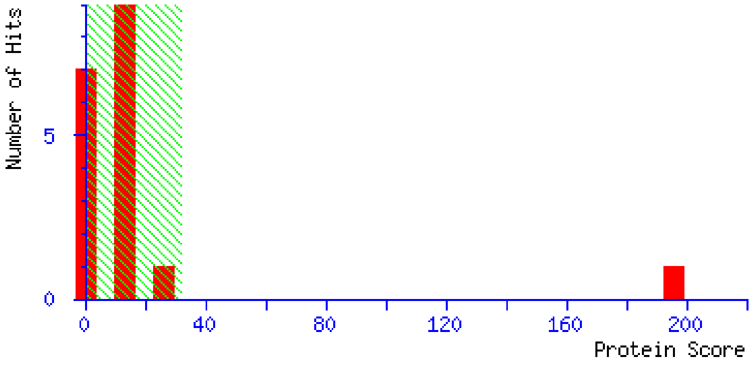

Spot No.: **61**

NCBI accession No.: **cassava4.1_018737m|PACid:17986123**

Plant species: ***Manihot esculenta***

Protein name：**Actin-depolymerizing factor 3**

PFF Mascot score: [112]   Sequence coverage %: [11]

Matched peptides No.: **[3]**   p value: **5.3e-010**

Calculated Mr: **16253**   Calculated *p*I: **6.60**

Annotated PMF **spectra:**

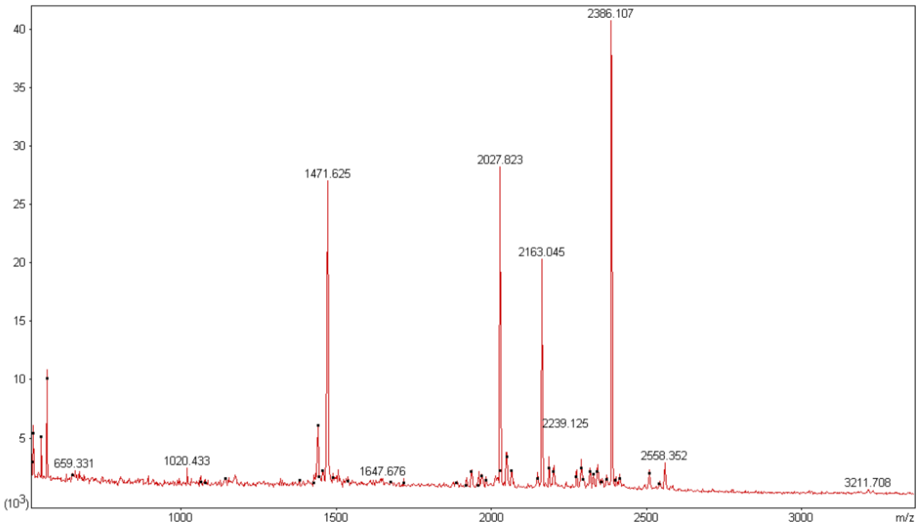

PFF Searched Score:

Ions score is -10*Log(P), where P is the probability that the observed match is a random event.
Individual ions scores > 32 indicate identity or extensive homology (p<0.05).
Protein scores are derived from ions scores as a non-probabilistic basis for ranking protein hits.

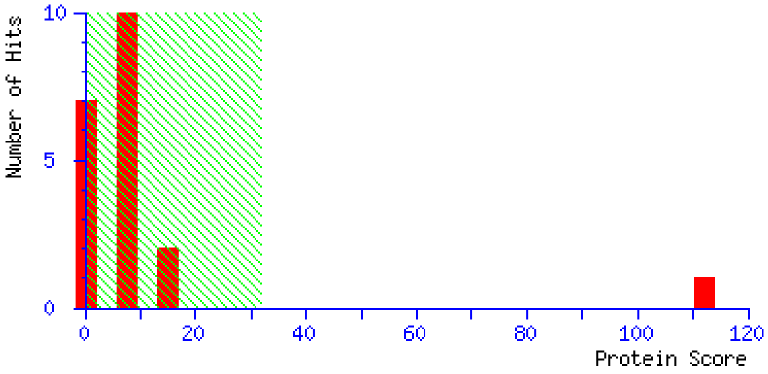

Spot No.: **62**

NCBI accession No.: **cassava4.1_017871m|PACid:17980723**

Plant species: ***Manihot esculenta***

Protein name： **class I heat shock protein**

PFF Mascot score: [95] **Sequence coverage %: [8]**

Matched peptides No.: [**2**] p value: **3.7e-008**

Calculated Mr: 18750 Calculated *p*I: **7.93**

Annotated PMF **spectra:**

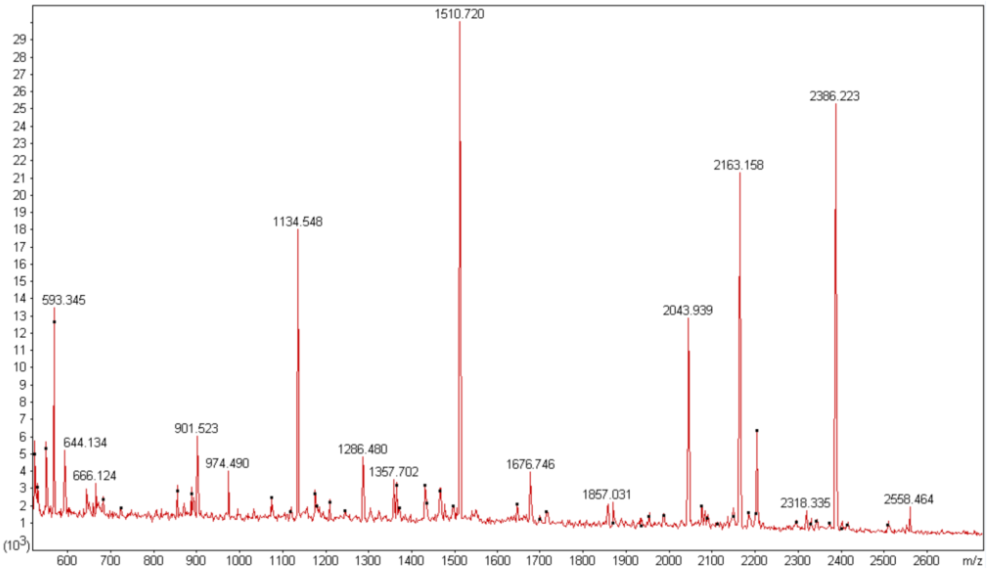

PFF Searched Score:

Ions score is -10*Log(P), where P is the probability that the observed match is a random event.
Individual ions scores > 33 indicate identity or extensive homology (p<0.05).
Protein scores are derived from ions scores as a non-probabilistic basis for ranking protein hits.

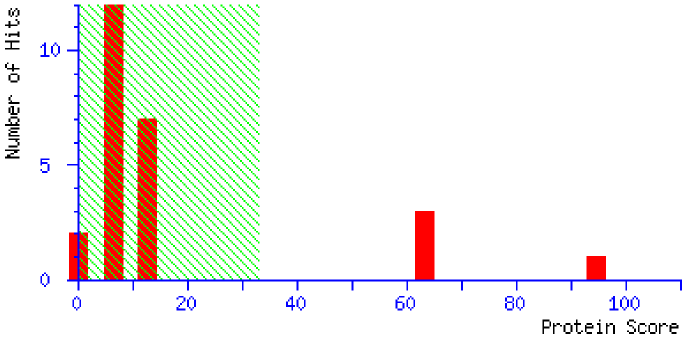

Spot No.: **63**

NCBI accession No.: **cassava4.1_018026m|PACid:17990511**

Plant species: ***Manihot esculenta***

Protein name： **Eukaryotic translation initiation factor 5A-2**

Mascot score: **82**

Sequence coverage %: **73**   p value: **0.00045**

Matched peptides No.: **11**   Total peptides No.: **76**

Calculated Mr: **17805**   Calculated *p*I: **5.60**

Annotated PMF **spectra:**

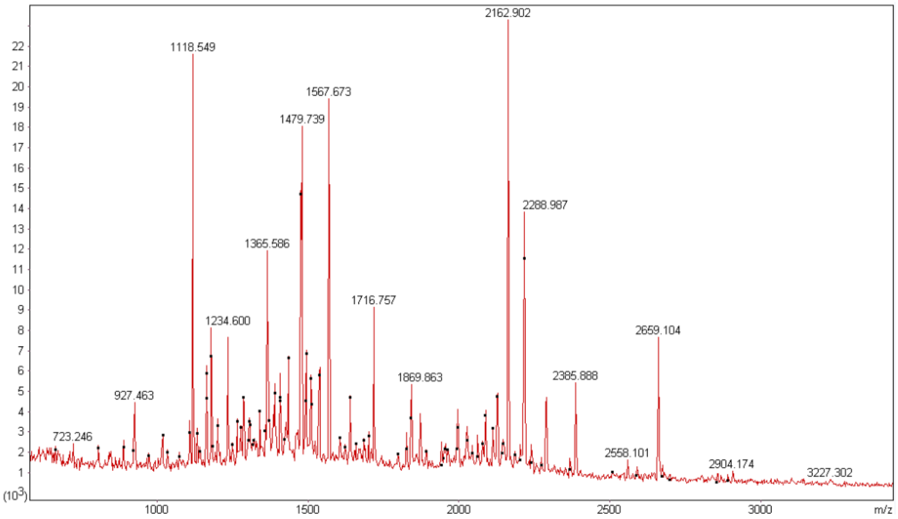

Probability Based Mowse Score:

Protein score is -10*Log(P), where P is the probability that the observed match is a random event. Protein scores greater than 61 are significant (p<0.05).

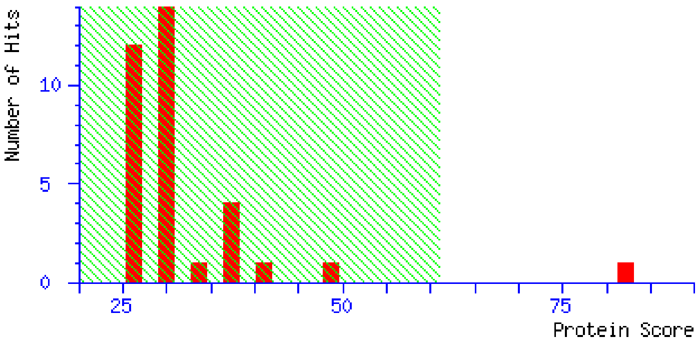

Spot No.: **64**

NCBI accession No.: **cassava4.1_017973m|PACid:17985926**

Plant species: ***Manihot esculenta***

Protein name：**Peroxiredoxin-2B**

PFF Mascot score: [**180**] Sequence coverage %: [**22**]

Matched peptides No.: [**10**] p value: 2e-013

Calculated Mr: **17433** Calculated *p*I: **5.70**

Annotated PMF **spectra:**

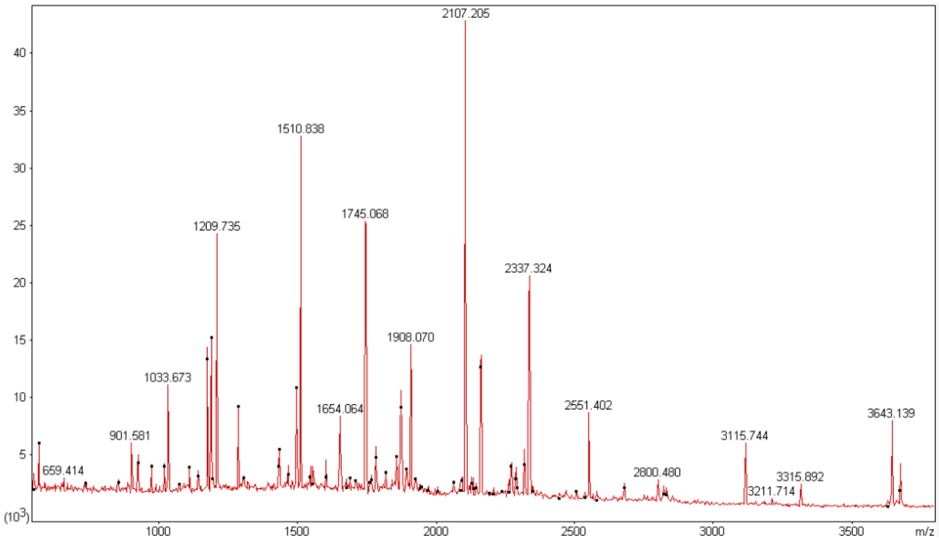

PFF Searched Score:

Ions score is -10*Log(P), where P is the probability that the observed match is a random event.
Individual ions scores > 32 indicate identity or extensive homology (p<0.05).
Protein scores are derived from ions scores as a non-probabilistic basis for ranking protein hits.

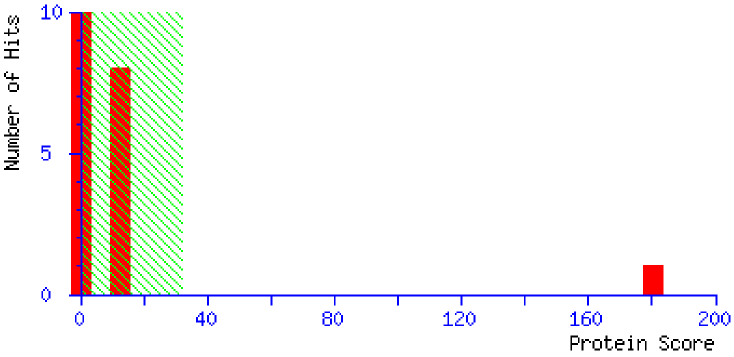

Spot No.: **65**

NCBI accession No.: **cassava4.1_017871m|PACid:17980723**

Plant species: ***Manihot esculenta***

Protein name：**18.1 kDa class I heat shock protein**

PFF Mascot score: **[164]** Sequence coverage %: **[25]**

Matched peptides No.: **[5]** p value: **0.00017**

Calculated Mr: **18750** Calculated *p*I: **7.93**

Annotated PMF **spectra:**

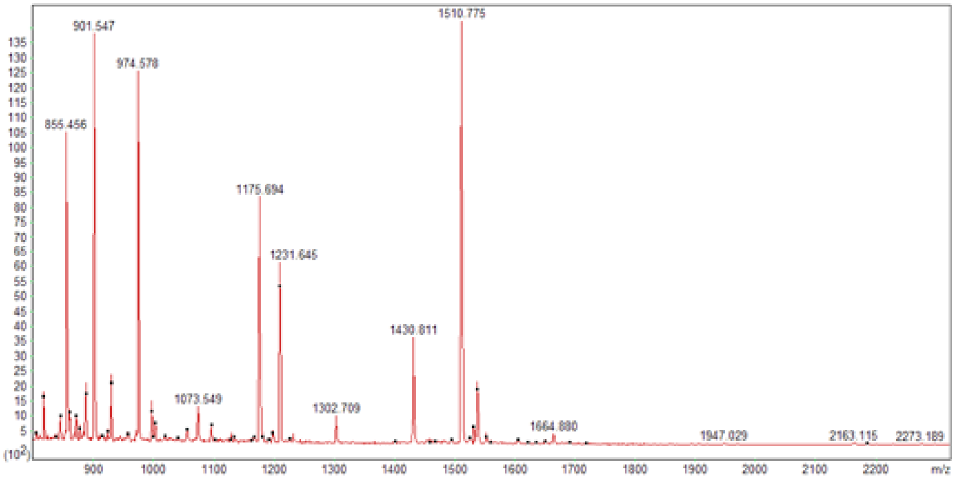

PFF Searched Score:

Ions score is -10*Log(P), where P is the probability that the observed match is a random event.
Individual ions scores > 34 indicate identity or extensive homology (p<0.05).
Protein scores are derived from ions scores as a non-probabilistic basis for ranking protein hits.

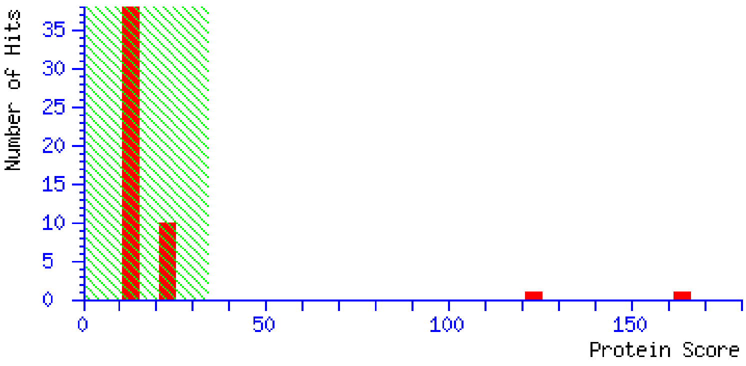

Spot No.: **66**

NCBI accession No.: **cassava4.1_018294m|PACid:17966054**

Plant species: ***Manihot esculenta***

Protein name： **Superoxide dismutase [Cu-Zn] 1**

PFF Mascot score: [80]   Sequence coverage %: [8]

Matched peptides No.: **[2]**   p value: **1.3e-006**

Calculated Mr: **15416**   Calculated *p*I: **5.85**

Annotated PMF **spectra:**

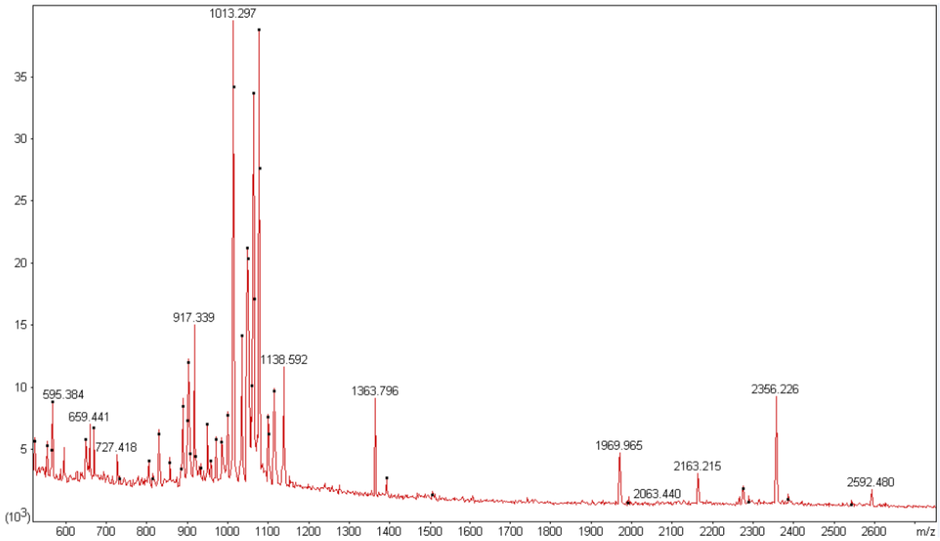

PFF Searched Score:

Ions score is -10*Log(P), where P is the probability that the observed match is a random event.
Individual ions scores > 33 indicate identity or extensive homology (p<0.05).
Protein scores are derived from ions scores as a non-probabilistic basis for ranking protein hits.

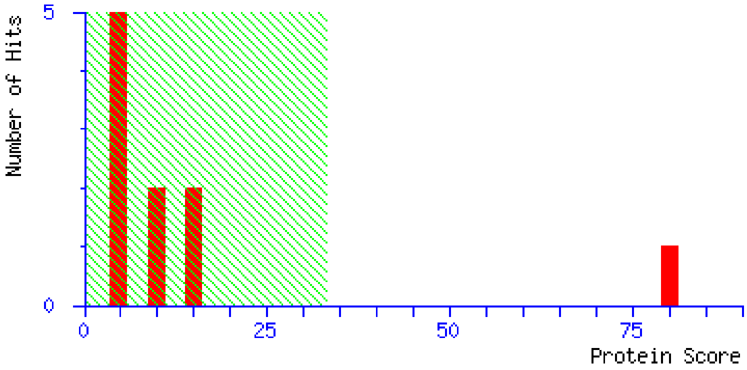

Spot No.: **67**

NCBI accession No.: **cassava4.1_018422m|PACid:17961678**

Plant species: ***Manihot esculenta***

Protein name: Actin-depolymerizing factor 3

PFF Mascot score: [147]

Sequence coverage %: [14]

Matched peptides No.: **[3]** p value: **1.5e-013**

Calculated Mr: **17512** Calculated *p*I: 8. 39

Annotated PMF **spectra:**

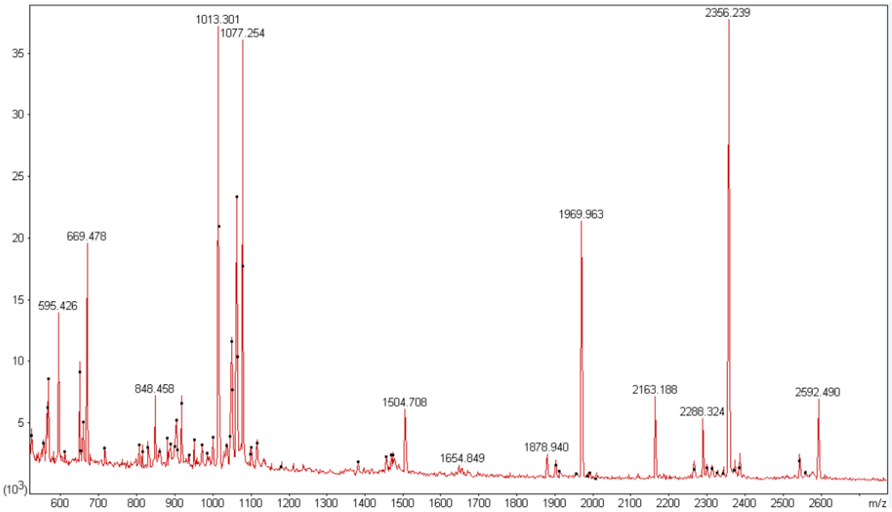

PFF Searched Score:

Ions score is -10*Log(P), where P is the probability that the observed match is a random event.
Individual ions scores > 33 indicate identity or extensive homology (p<0.05).
Protein scores are derived from ions scores as a non-probabilistic basis for ranking protein hits.

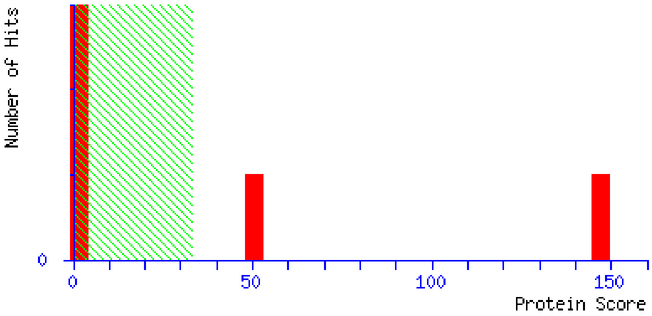

Spot No.: **68**

NCBI accession No.: **cassava4.1_018338m|PACid:17992346**

Plant species: ***Manihot esculenta***

Protein name: **Ubiquitin-conjugating enzyme E2 variant 1C**

Mascot score: **119**

Sequence coverage %: **72**

p value: **9.2e-008**

Matched peptides No.: **12**

Total peptides No.: **37**

Calculated Mr: **17236**

Calculated *p*I: **5.73**

Annotated PMF **spectra:**

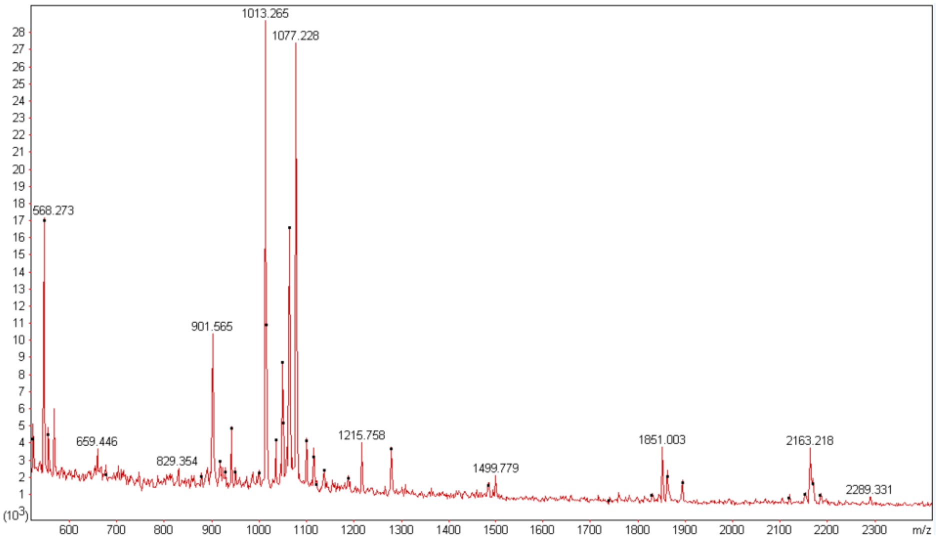

Probability Based Mowse Score:

Protein score is -10*Log(P), where P is the probability that the observed match is a random event.
Protein scores greater than 61 are significant (p<0.05).

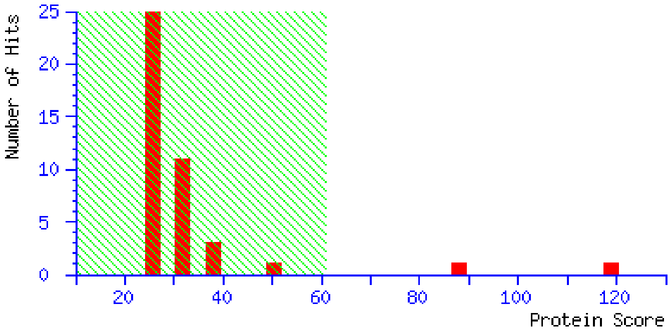

Spot No.: **69**

NCBI accession No.: **cassava4.1_018010m|PACid:17970081**

Plant species: ***Manihot esculenta***

Protein name： **AT3g53990/F5K20_290**

PFF Mascot score: [195] **Sequence coverage %: [21]**

Matched peptides No.: **[3]** p value: **5.2e-006**

Calculated Mr: **18044** Calculated *p*I: **6.14**

Annotated PMF **spectra:**

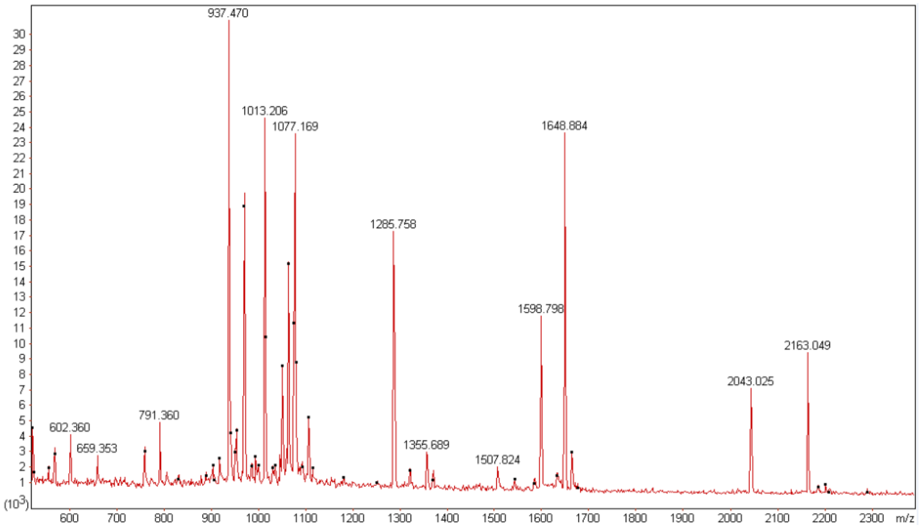

PFF Searched Score:

Ions score is -10*Log(P), where P is the probability that the observed match is a random event.
Individual ions scores > 33 indicate identity or extensive homology (p<0.05).
Protein scores are derived from ions scores as a non-probabilistic basis for ranking protein hits.

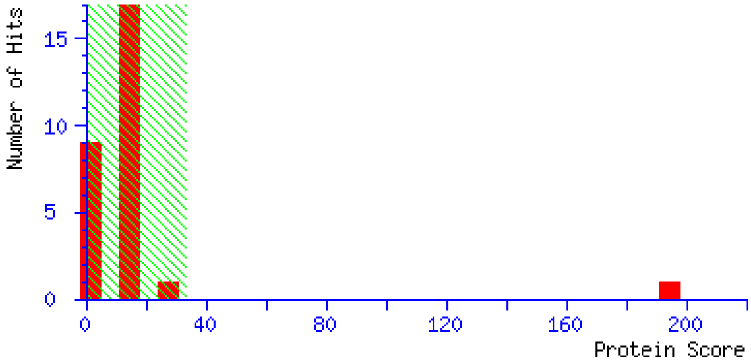

Spot No.: **70**

NCBI accession No.: **cassava4.1_015272m|PACid:17960181**

Plant species: ***Manihot esculenta***

Protein name: **Superoxide dismutase [Mn] 1, mitochondrial**

Mascot score: **139**

Sequence coverage %: **54** p value: **9.2e-010**

Matched peptides No.: **16** Total peptides No.:**67**

Calculated Mr: **25905** Calculated *p*I: **7.82**

Annotated PMF **spectra:**

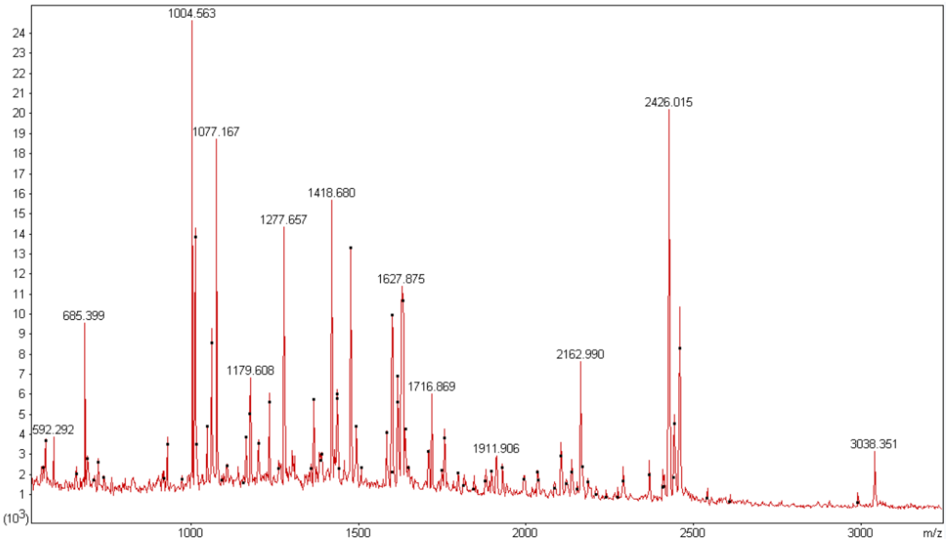

Probability Based Mowse Score:

Protein score is -10*Log(P), where P is the probability that the observed match is a random event. Protein scores greater than 61 are significant (p<0.05).

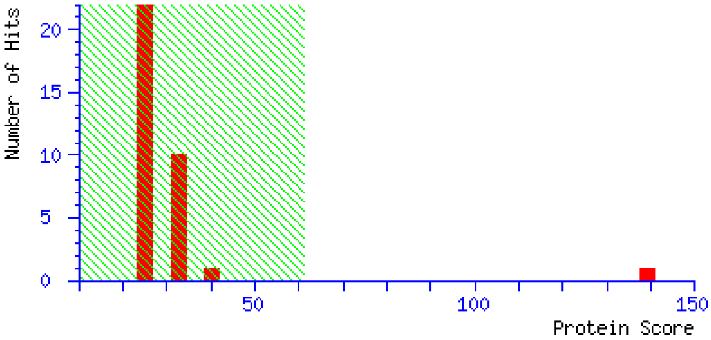

Spot No.: 71

NCBI accession No.: cassava4.1_013840m|PACid:17968262

Plant species: ***Manihot esculenta***

Protein name： **Proteasome subunit alpha type-2-A**

PFF Mascot score: [99] **Sequence coverage %: [7]**

Matched peptides No.: **[2]** p value: **0.0001**

Calculated Mr: **29845** Calculated *p*I: **6.13**

Annotated PMF **spectra:**

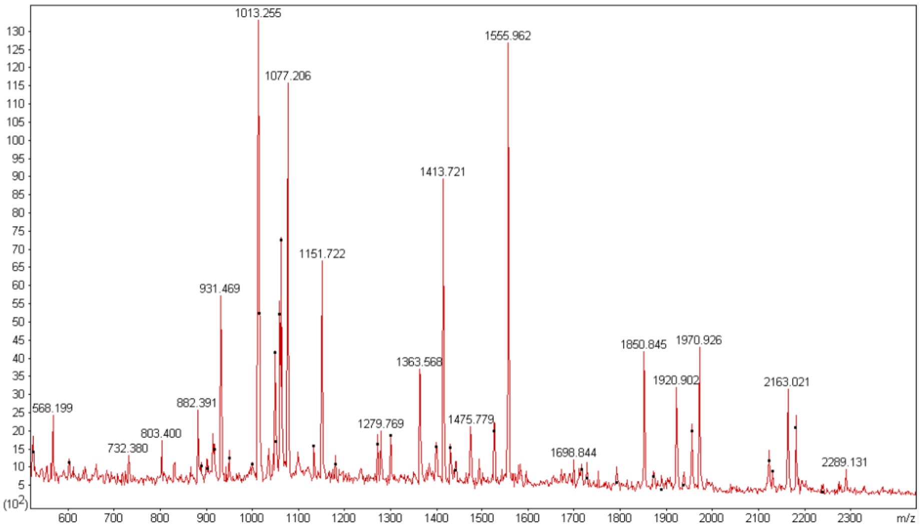

PFF Searched Score:

Ions score is -10*Log(P), where P is the probability that the observed match is a random event.
Individual ions scores > 33 indicate identity or extensive homology (p<0.05).
Protein scores are derived from ions scores as a non-probabilistic basis for ranking protein hits.

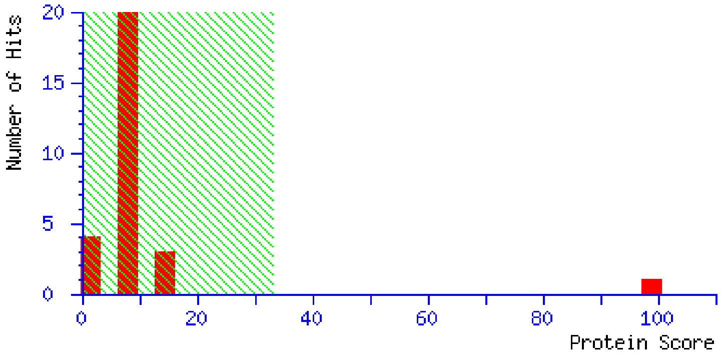

Spot No.: **72**

NCBI accession No.: **cassava4.1_013784m|PACid:17965032**

Plant species: ***Manihot esculenta***

Protein name：**Superoxide dismutase [Fe] 2, chloroplastic**

PFF Mascot score: [94] **Sequence coverage %: [11]**

Matched peptides No.: **[3]** p value: **0.0079**

Calculated Mr: **30888** Calculated *p*I: **6.67**

Annotated PMF **spectra:**

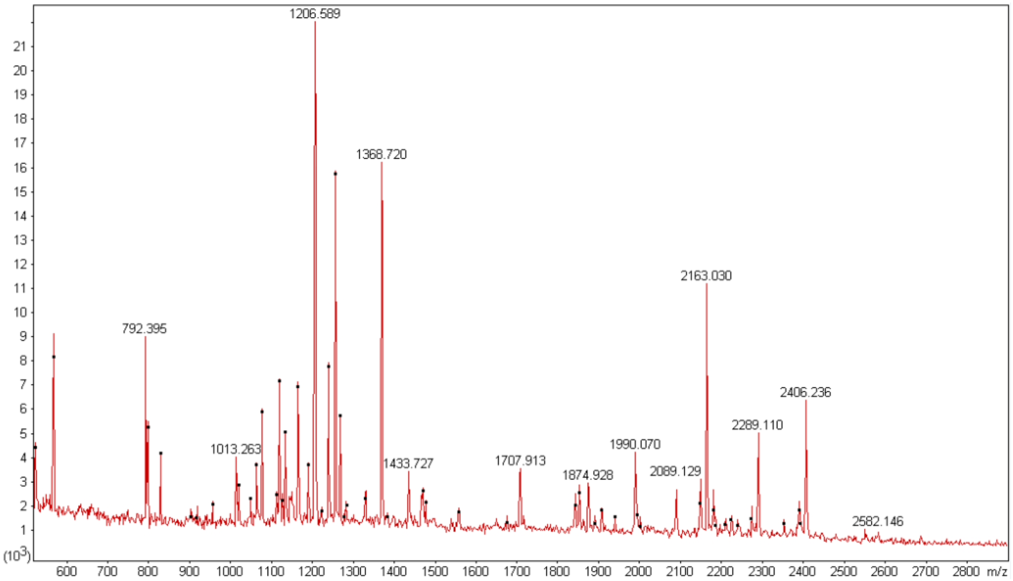

PFF Searched Score:

Ions score is -10*Log(P), where P is the probability that the observed match is a random event.
Individual ions scores > 34 indicate identity or extensive homology (p<0.05).
Protein scores are derived from ions scores as a non-probabilistic basis for ranking protein hits.

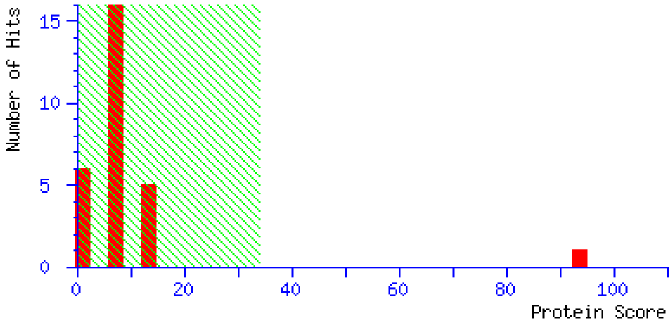

Spot No.: **73**

NCBI accession No.: **cassava4.1_014423m|PACid:17980868**

Plant species: ***Manihot esculenta***

Protein name： **L-ascorbate peroxidase 1, cytosolic**

PFF Mascot score: [234] **Sequence coverage %:** [**17]**

Matched peptides No.: **[3]** p value: **1.1e-010**

Calculated Mr: **28235** Calculated *p*I: **6.09**

Annotated PMF **spectra:**

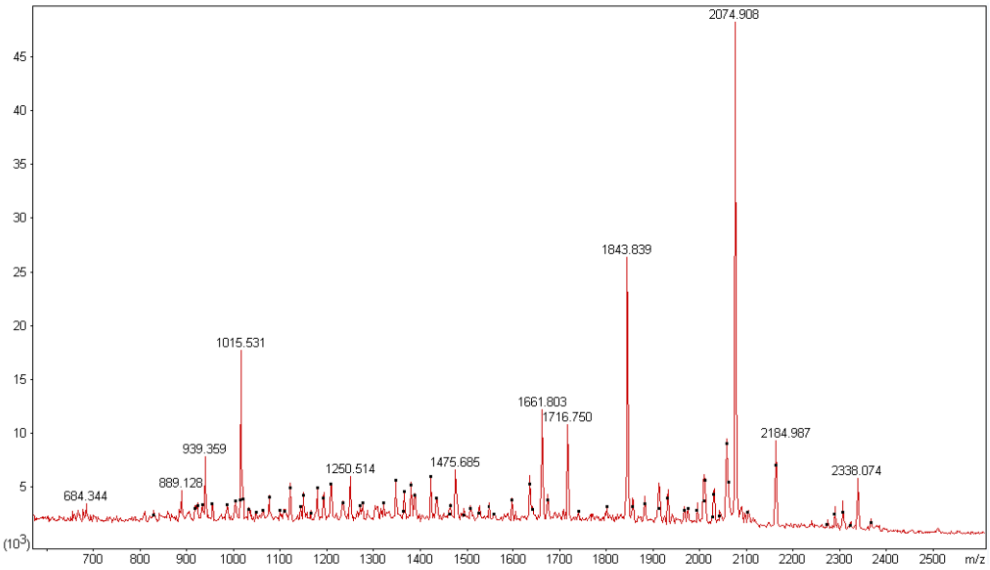

PFF Searched Score:

Ions score is -10*Log(P), where P is the probability that the observed match is a random event.
Individual ions scores > 33 indicate identity or extensive homology (p<0.05).
Protein scores are derived from ions scores as a non-probabilistic basis for ranking protein hits.

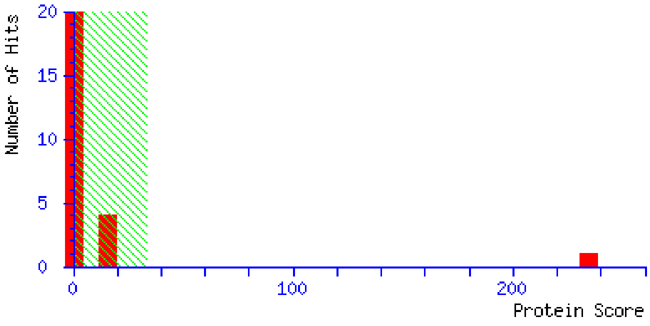

Spot No.: **74**

NCBI accession No.: **cassava4.1_014432m|PACid:17967798**

Plant species: ***Manihot esculenta***

Protein name: **Triosephosphate isomerase, cytosolic**

PFF Mascot score: [194] **Sequence coverage %: [16]**

Matched peptides No.: **[3]** p value: **9.9e-006**

Calculated Mr: **27729** Calculated *p*I: **5.77**

Annotated PMF **spectra:**

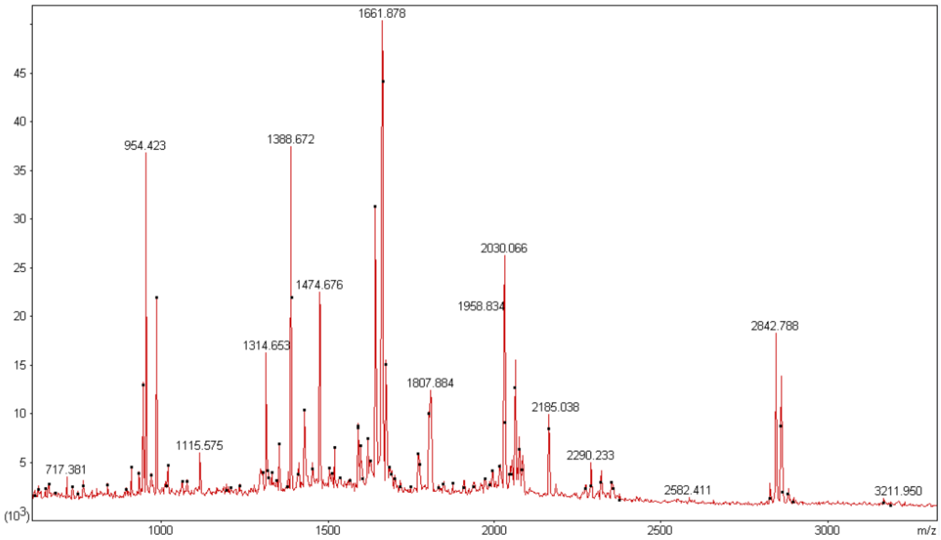

PFF Searched Score:

Ions score is -10*Log(P), where P is the probability that the observed match is a random event.
Individual ions scores > 33 indicate identity or extensive homology (p<0.05).
Protein scores are derived from ions scores as a non-probabilistic basis for ranking protein hits.

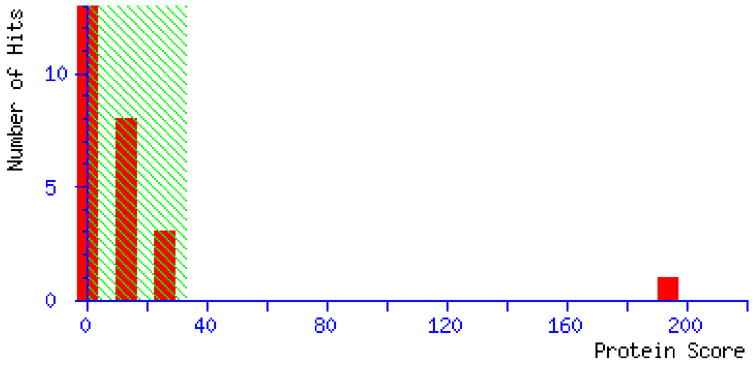

Spot No.: **75**

NCBI accession No.: **cassava4.1_014799m|PACid:17972994**

Plant species: ***Manihot esculenta***

Protein name：**Proteasome subunit alpha type-6-A**

Mascot score: **131**

Sequence coverage %: **52**   p value: **5.8e-009**

Matched peptides No.: **14**   Total peptides No.: **35**

Calculated Mr: **27642**   Calculated *p*I: **6.19**

Annotated PMF **spectra:**

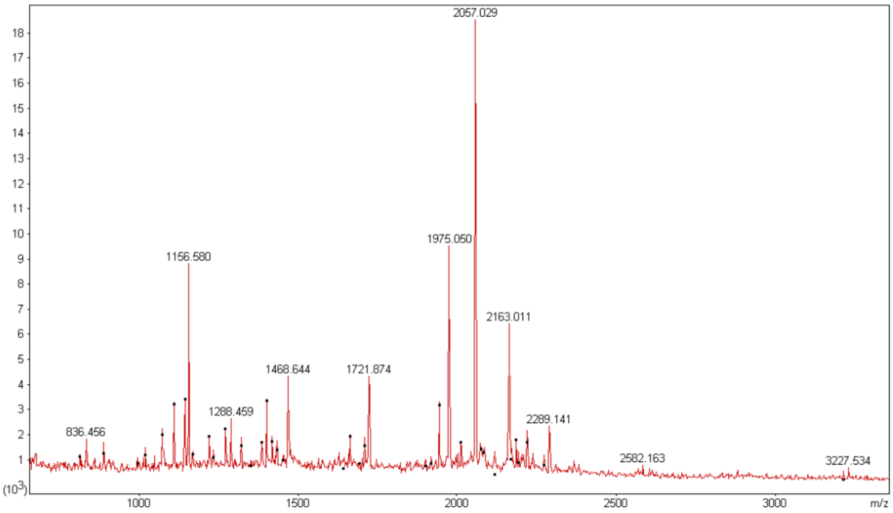

Probability Based Mowse Score:

Protein score is -10*Log(P), where P is the probability that the observed match is a random event.
Protein scores greater than 61 are significant (p<0.05).

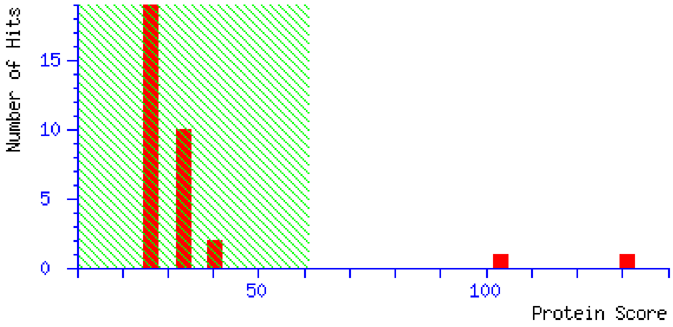

Spot No.: **76**

NCBI accession No.: **cassava4.1_010620m|PACid:17987783**

Plant species Manihot esculenta

Protein name：**Thiamine thiazole synthase, chloroplastic**

PFF Mascot score: [162] **Sequence coverage %:** [**8]**

Matched peptides No.: **[2]** p value: **1.8e-007**

Calculated Mr: 37625 Calculated *p*I: **6.07**

Annotated PMF **spectra:**

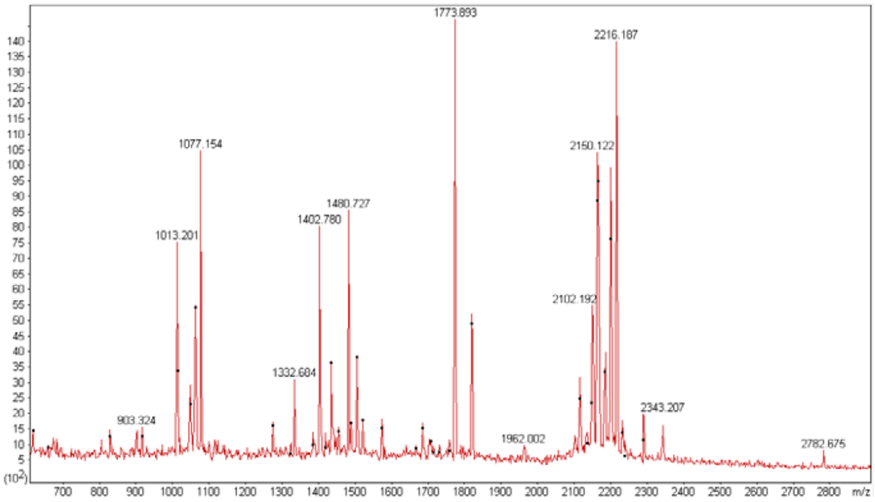

Probability Based Mowse Score:

Ions score is -10*Log(P), where P is the probability that the observed match is a random event.
Individual ions scores > 33 indicate identity or extensive homology (p<0.05).
Protein scores are derived from ions scores as a non-probabilistic basis for ranking protein hits.

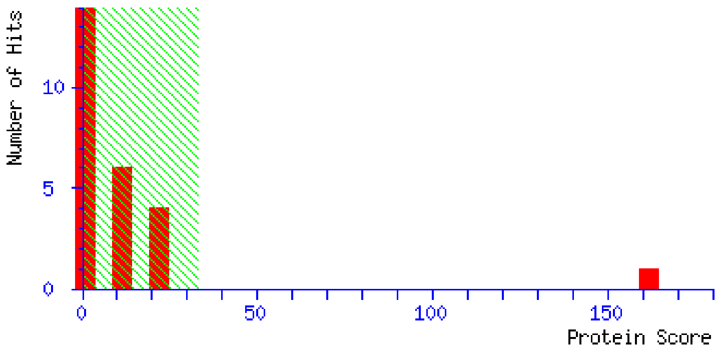

Spot No.: **77**

NCBI accession No.: **cassava4.1_010971m|PACid:17987784**

Plant species: ***Manihot esculenta***

Protein name：**Thiamine thiazole synthase, chloroplastic**

Mascot score: **88**

Sequence coverage %: **38**   p value: **0.00011**

Matched peptides No.: **17**   Total peptides No.: **70**

Calculated Mr: **36908**   Calculated *p*I: **7.10**

Annotated PMF **spectra:**

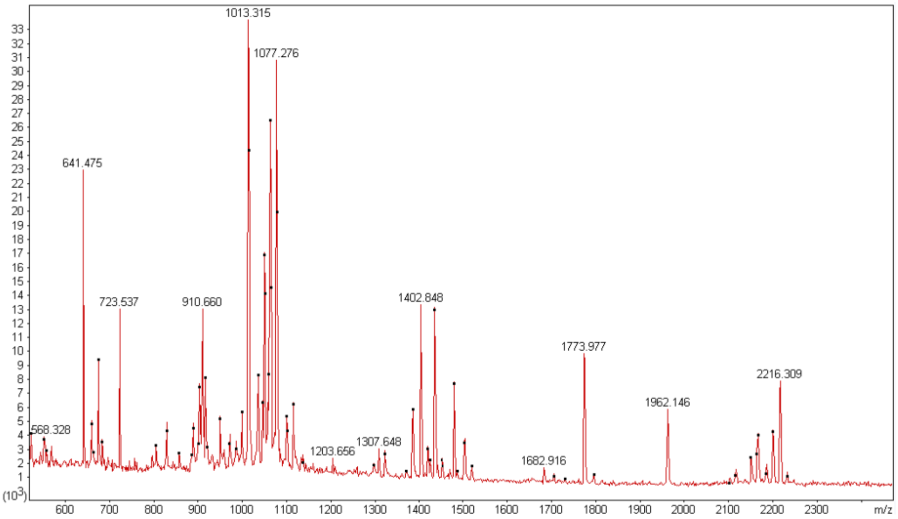

Probability Based Mowse Score:

Protein score is -10*Log(P), where P is the probability that the observed match is a random event. Protein scores greater than 61 are significant (p<0.05).

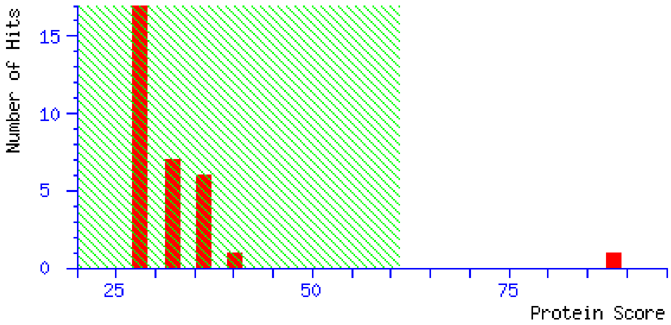

Spot No.: **78**

NCBI accession No.: **cassava4.1_009245m|PACid:17963775**

Plant species: ***Manihot esculenta***

Protein name： **S-adenosylmethionine synthase 1**

Mascot score: **119**

Sequence coverage %: **55** p value: **9.2e-008**

Matched peptides No.: **16** Total peptides No.: **54**

Calculated Mr: **43709** Calculated *p*I: **5.68**

Annotated PMF **spectra:**

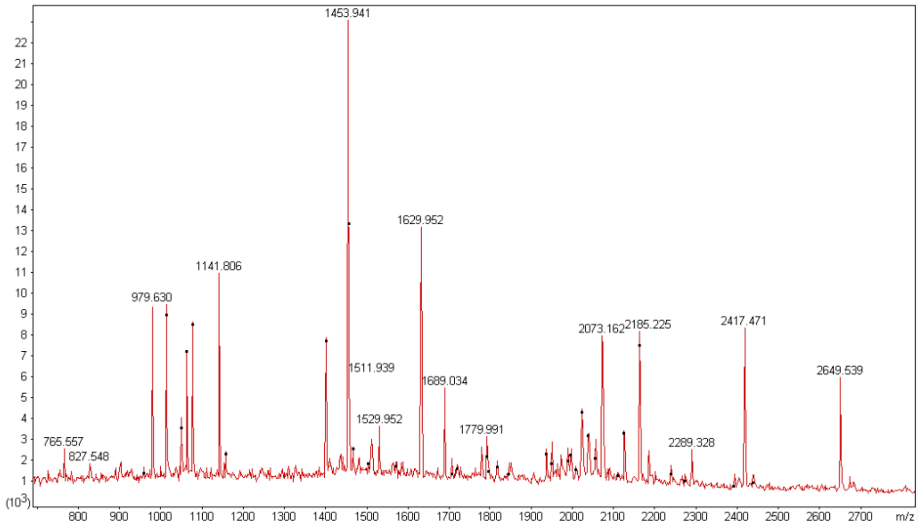

Probability Based Mowse Score:

Protein score is -10*Log(P), where P is the probability that the observed match is a random event. Protein scores greater than 61 are significant (p<0.05).

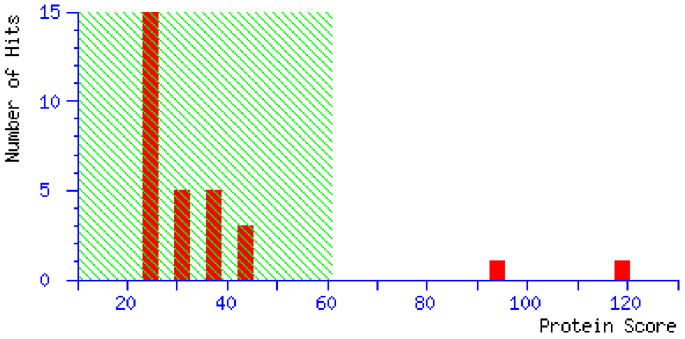

Spot No.: **79**

NCBI accession No.: **cassava4.1_011550m|PACid:17993466**

Plant species: ***Manihot esculenta***

Protein name: **predicted protein** Mascot score: **108**

Sequence coverage %: **50** p value: **1.2e-006**

Matched peptides No.: **17** Total peptides No.: **67**

Calculated Mr: **36178** Calculated *p*I: **6.09**

Annotated PMF **spectra:**

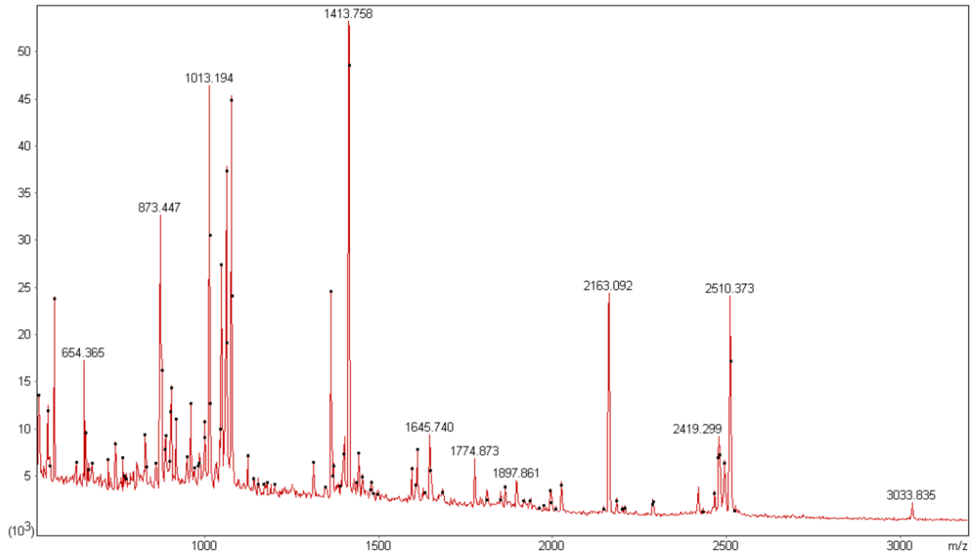

Probability Based Mowse Score:

Protein score is -10*Log(P), where P is the probability that the observed match is a random event. Protein scores greater than 61 are significant (p<0.05).

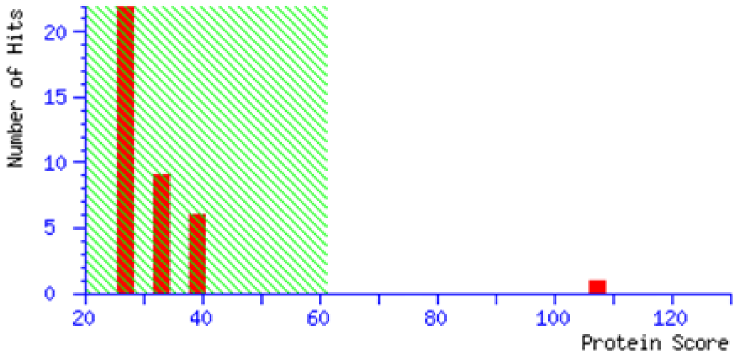

Spot No.: **80**

NCBI accession No.: **cassava4.1_009410m|PACid:17973470**

Plant species: ***Manihot esculenta***

Protein name： **UDP-D-apiose/UDP-D-xylose synthase 1**

Mascot score: **163**

Sequence coverage %: **51**   p value: **3.7e-012**

Matched peptides No.: **24**   Total peptides No.: **73**

Calculated Mr: **43967**   Calculated *p*I: **6.03**

Annotated PMF **spectra:**

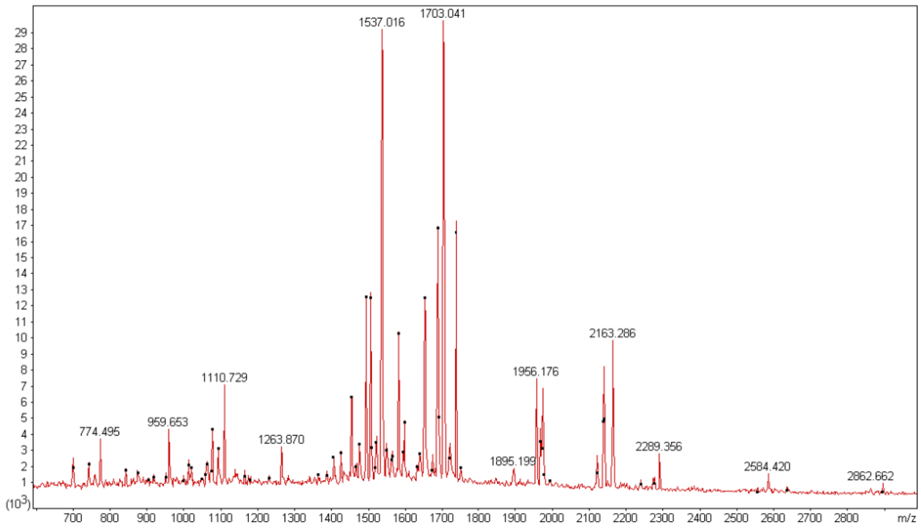

Probability Based Mowse Score:

Protein score is -10*Log(P), where P is the probability that the observed match is a random event.
Protein scores greater than 61 are significant (p<0.05).

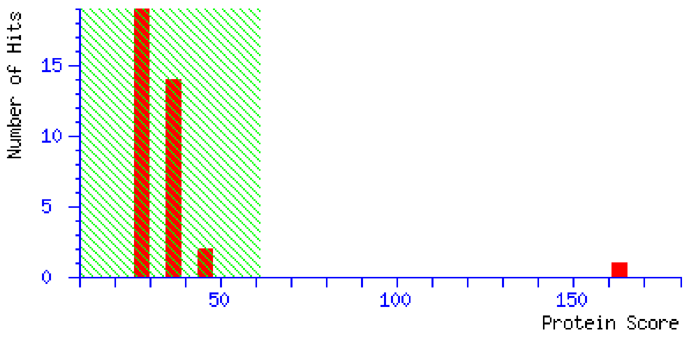

Spot No.: **81**

NCBI accession No.: **cassava4.1_008604m|PACid:17979400**

Plant species: ***Manihot esculenta***

Protein name： **Cytosolic isocitrate dehydrogenase [NADP]**

Mascot score: **115**

Sequence coverage %: **45** p value: **2.3e-007**

Matched peptides No.: **17** Total peptides No.: **49**

Calculated Mr: **46844** Calculated *p*I: **6.07**

Annotated PMF **spectra:**

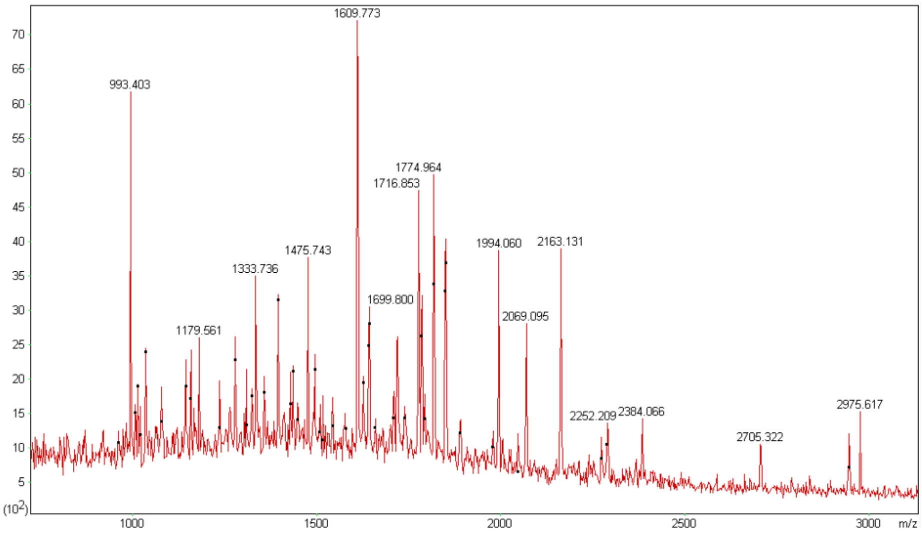

Probability Based Mowse Score:

Protein score is -10*Log(P), where P is the probability that the observed match is a random event. Protein scores greater than 61 are significant (p<0.05).

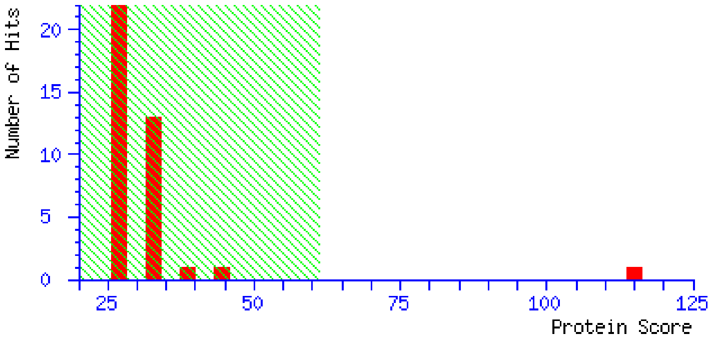

Spot No.: **82**

NCBI accession No.: **cassava4.1_009808m|PACid:17965663**

Plant species: ***Manihot esculenta***

Protein name: **GDP-mannose 3,5-epimerase** Mascot score: **76**

Sequence coverage %: **41** p value: **0.002**

Matched peptides No.: **14** Total peptides No.: **58**

Calculated Mr: **42843** Calculated *p*I: **6.05**

Annotated PMF **spectra:**

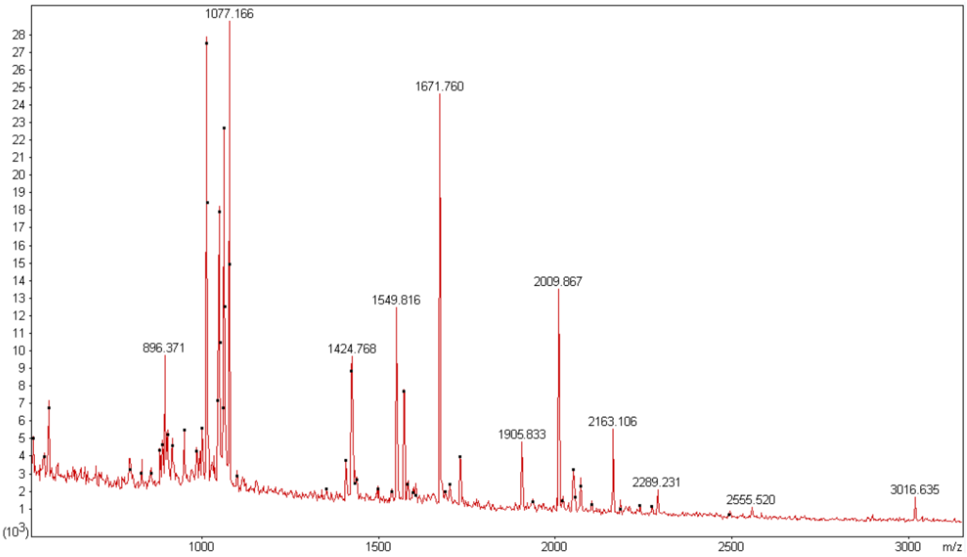

Probability Based Mowse Score:

Protein score is -10*Log(P), where P is the probability that the observed match is a random event. Protein scores greater than 61 are significant (p<0.05).

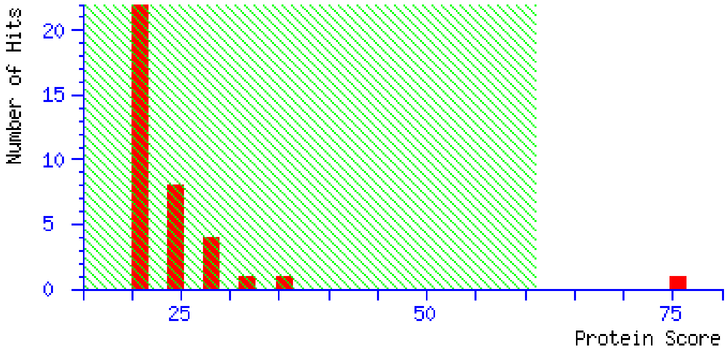

Spot No.: **83**

NCBI accession No.: **cassava4.1_007673m|PACid:17979089**

Plant species: ***Manihot esculenta***

Protein name： **Bifunctional enolase 2/transcriptional activator**

Mascot score: **124**

Sequence coverage %: **42** p value: **2.9e-008**

Matched peptides No.: **17** Total peptides No.: **57**

Calculated Mr: **48058** Calculated *p*I: **5.86**

Annotated PMF **spectra:**

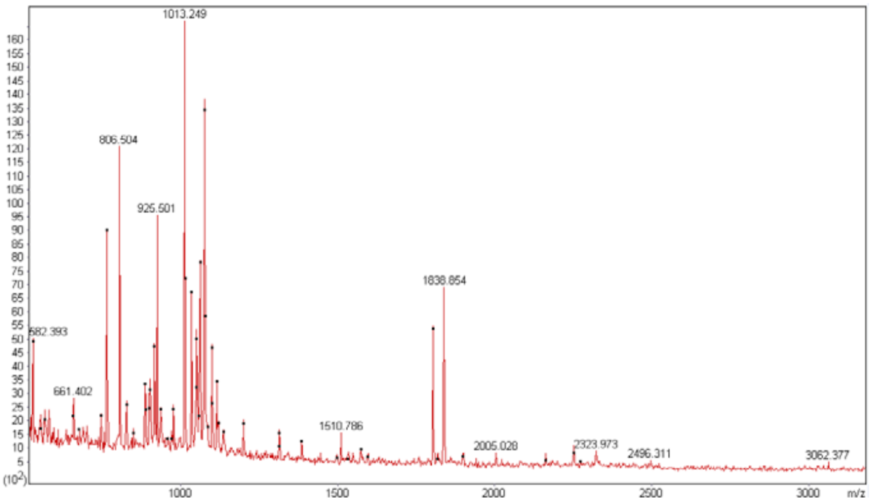

Probability Based Mowse Score:

Protein score is -10*Log(P), where P is the probability that the observed match is a random event. Protein scores greater than 61 are significant ($p<0.05$).

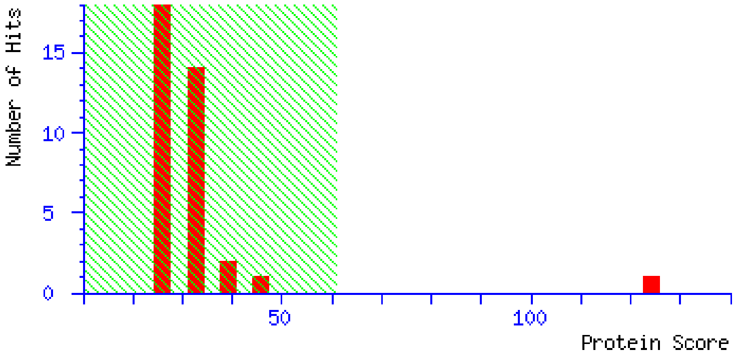

Spot No.: **84**

NCBI accession No.: **cassava4.1_007678m|PACid:17961094**

Plant species: ***Manihot esculenta***

Protein name: **Bifunctional enolase 2/transcriptional activator**

Mascot score: **100**

Sequence coverage %: **46** p value: **0.0000073**

Matched peptides No.: **14** Total peptides No.: **45**

Calculated Mr: **47854** Calculated *p*I: **5.86**

Annotated PMF **spectra:**

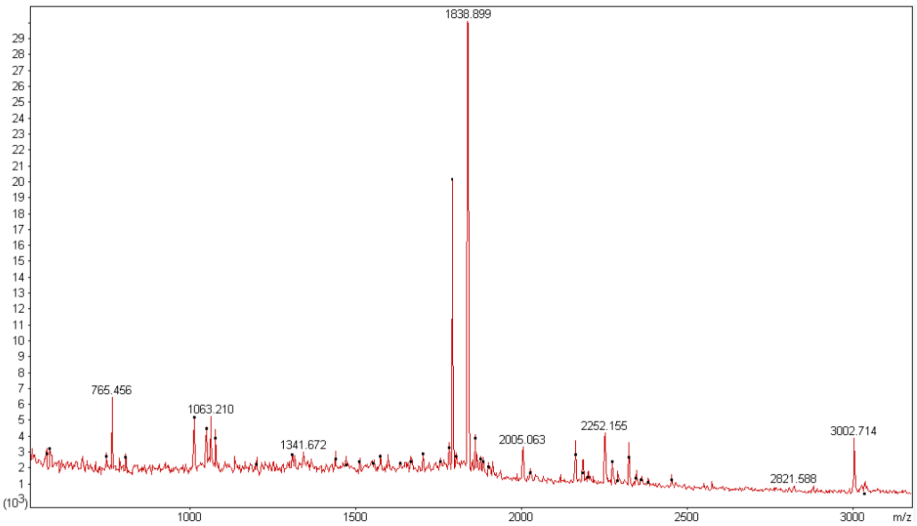

Probability Based Mowse Score:

Protein score is -10*Log(P), where P is the probability that the observed match is a random event.
Protein scores greater than 61 are significant (p<0.05).

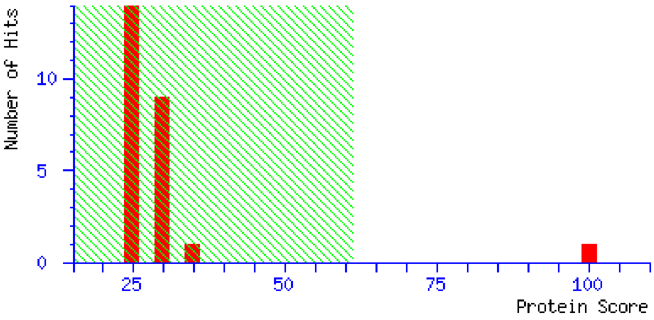

Spot No.: **85**

NCBI accession No.: **cassava4.1_033530m|PACid:17973804**

Plant species: ***Manihot esculenta***

Protein name： **ATP synthase F1 subunit 1**

Mascot score: **151** p value: **1.3e-09**

Sequence coverage %: **42**

Matched peptides No.: **20** Total peptides No.: **56**

Calculated Mr: **55536** Calculated *p*I: **6.02**

Annotated PMF **spectra:**

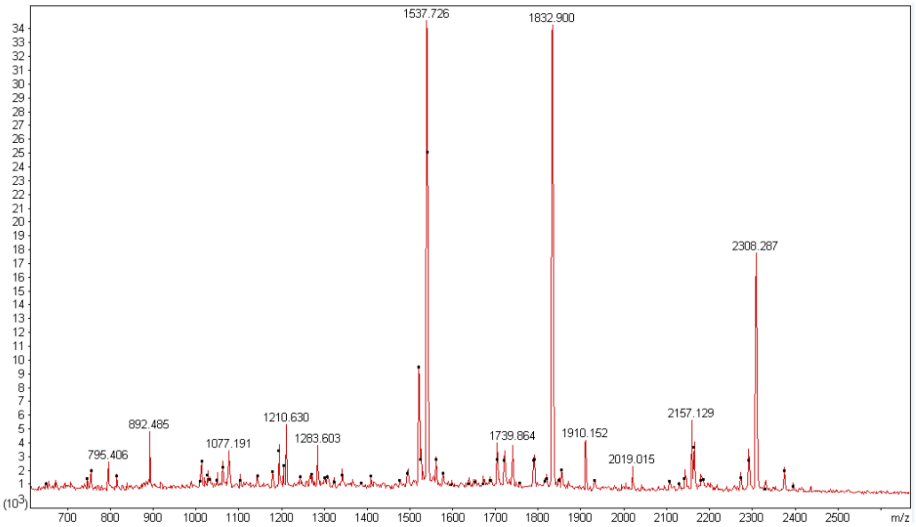

Probability Based Mowse Score:

Protein score is -10*Log(P), where P is the probability that the observed match is a random event. Protein scores greater than 75 are significant (p<0.05).

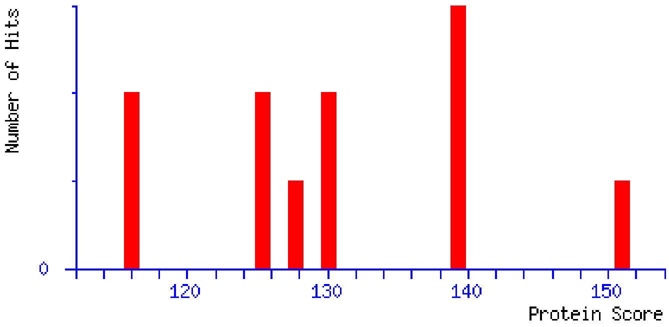

Spot No.: **86**

NCBI accession No.: **cassava4.1_004164m|PACid:17985770**

Plant species: ***Manihot esculenta***

Protein name： **malic enzyme, putative**

Mascot score: **177**

Sequence coverage %: **47** p value: **1.5e-013**

Matched peptides No.: **33** Total peptides No.: **90**

Calculated Mr: **65246** Calculated *p*I: **6.03**

Annotated PMF **spectra:**

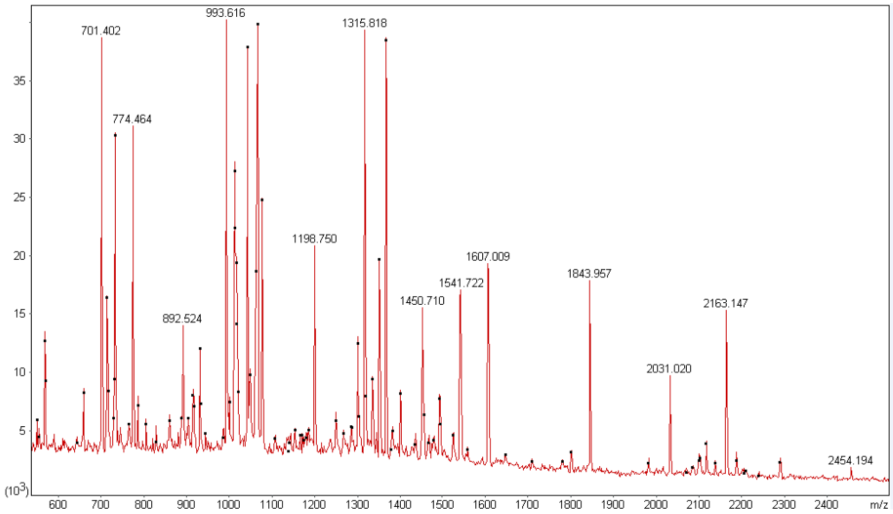

Probability Based Mowse Score:

Protein score is -10*Log(P), where P is the probability that the observed match is a random event. Protein scores greater than 61 are significant (p<0.05).

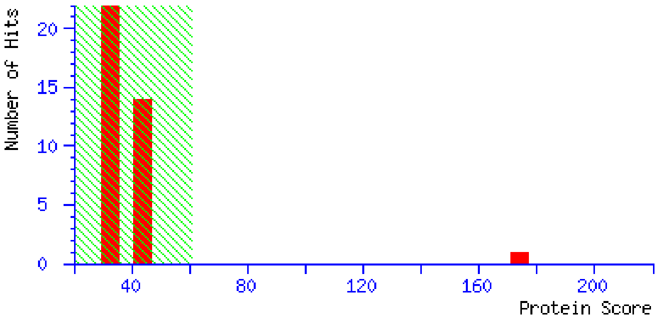

Spot No.: **87**

NCBI accession No.: **cassava4.1_007673m|PACid:17979089**

Plant species: ***Manihot esculenta***

Protein name：**Bifunctional enolase 2/transcriptional activator**

PFF Mascot score: **[150]**

Sequence coverage %: **[7]**

Matched peptides No.: **[2]**

p value: **1e-013**

Calculated Mr: **48058**

Calculated *p*I: **5.86**

Annotated PMF **spectra:**

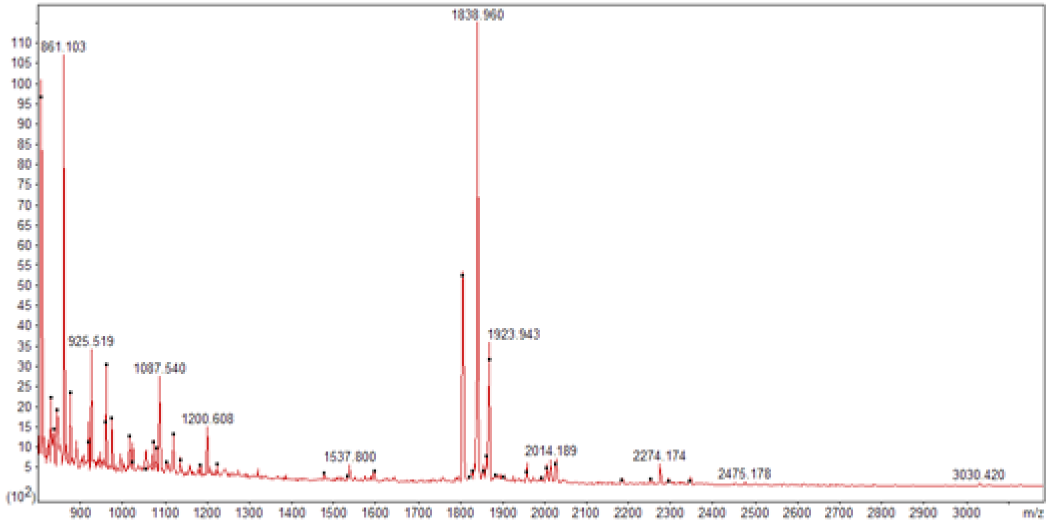

PFF Searched Score:

Ions score is -10*Log(P), where P is the probability that the observed match is a random event.
Individual ions scores > 33 indicate identity or extensive homology (p<0.05).
Protein scores are derived from ions scores as a non-probabilistic basis for ranking protein hits.

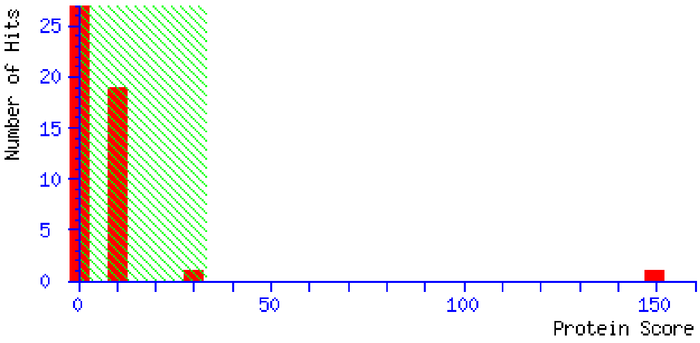

Spot No.: **88**

NCBI accession No.: **cassava4.1_007678m|PACid:17961094**

Plant species: ***Manihot esculenta***

Protein name: **Bifunctional enolase 2/transcriptional activator**

Mascot score: **160**

Sequence coverage %: **62** p value: **7.3e-012**

Matched peptides No.: **25** Total peptides No.: **78**

Calculated Mr: **47854** Calculated *p*I: **5.86**

Annotated PMF **spectra:**

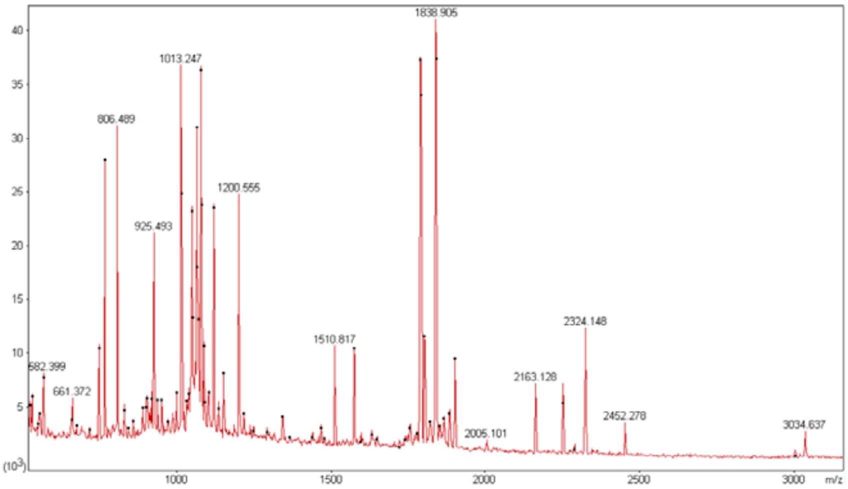

Probability Based Mowse Score:

Protein score is -10*Log(P), where P is the probability that the observed match is a random event. Protein scores greater than 61 are significant (p<0.05).

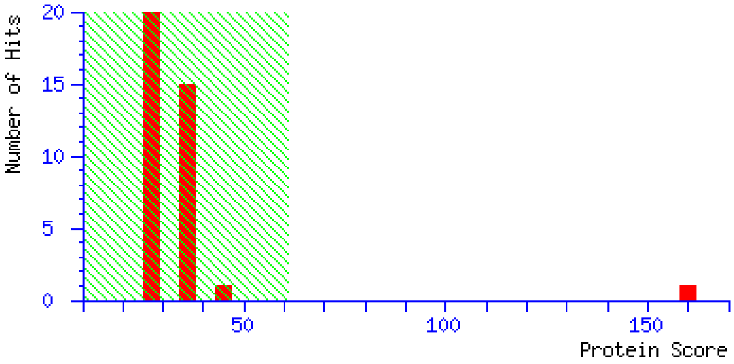

Spot No.: **89**

NCBI accession No.: **cassava4.1_003677m|PACid:17968299**

Plant species: ***Manihot esculenta***

Protein name：**V-type proton ATPase catalytic subunit A**

Mascot score: **234**

Sequence coverage %: **61** p value: **2.9e-019**

Matched peptides No.: **41** Total peptides No.: **95**

Calculated Mr: **68989** Calculated *p*I: **5.29**

Annotated PMF **spectra:**

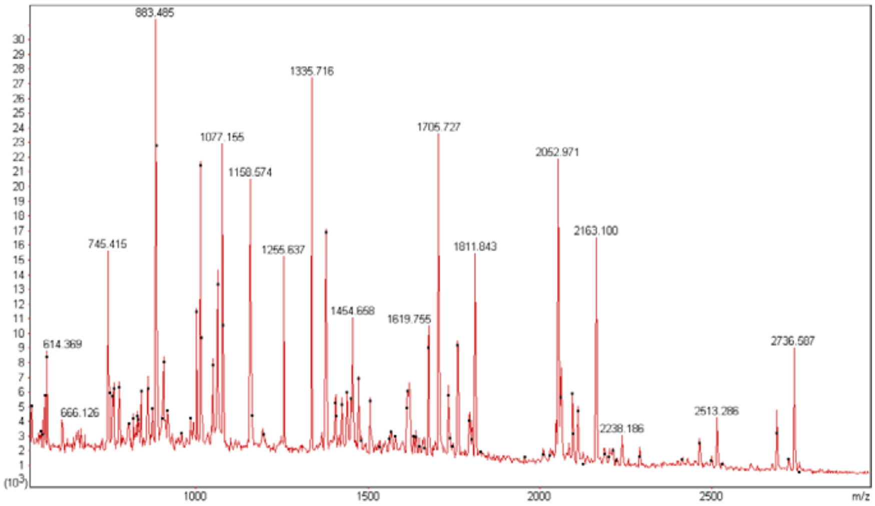

Probability Based Mowse Score:

Protein score is -10*Log(P), where P is the probability that the observed match is a random event.
Protein scores greater than 61 are significant (p<0.05).

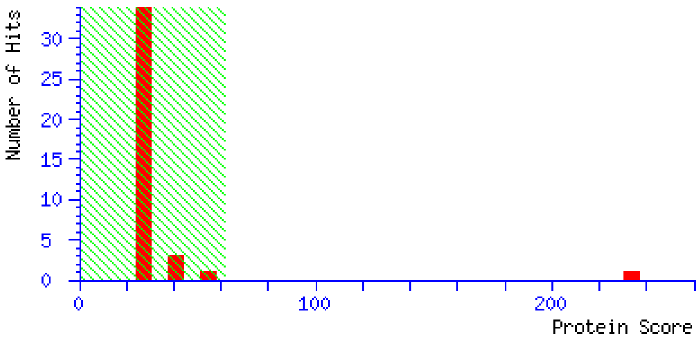

Spot No.: **90**

NCBI accession No.: **cassava4.1_003676m|PACid:17974360**

Plant species: ***Manihot esculenta***

Protein name：**V-type proton ATPase catalytic subunit A**

Mascot score: **194**

Sequence coverage %: **49**   p value: **2.9e-015**

Matched peptides No.: **30**   Total peptides No.: **68**

Calculated Mr: **68956**   Calculated *p*I: **5.34**

Annotated PMF **spectra:**

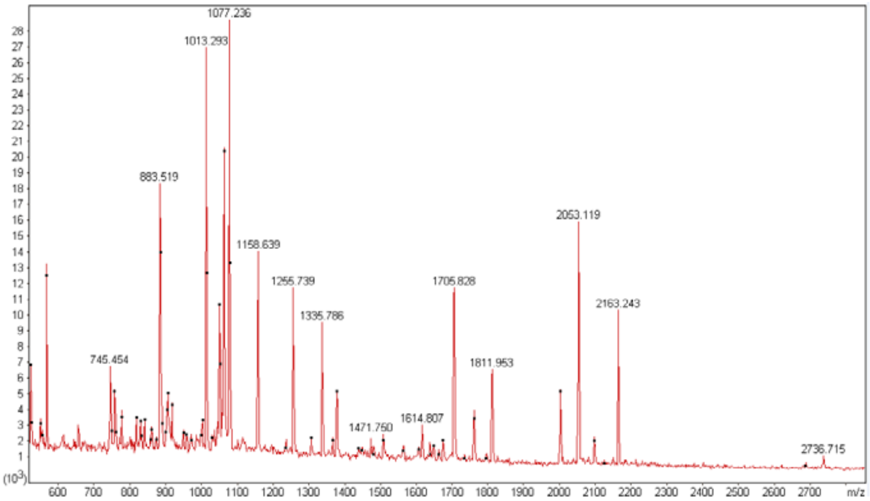

Probability Based Mowse Score:

Protein score is -10*Log(P), where P is the probability that the observed match is a random event.
Protein scores greater than 61 are significant (p<0.05).

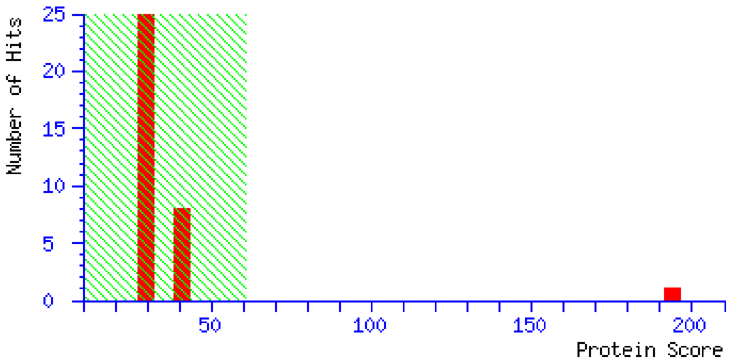

Spot No.: **91**

NCBI accession No.: **cassava4.1_005518m|PACid:17993865**

Plant species: ***Manihot esculenta***

Protein name：**Glucose-1-phosphate adenylyltransferase small subunit, chloroplastic** Mascot score: **81**

Sequence coverage %: **34** p value: **0.00053**

Matched peptides No.: **16** Total peptides No.: **63**

Calculated Mr: **57581** Calculated *p*I: **6.69**

Annotated PMF **spectra:**

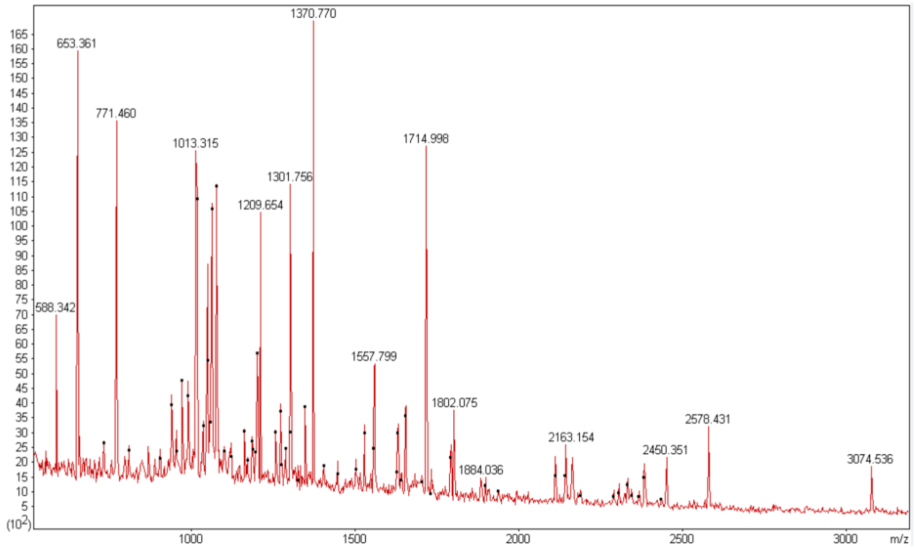

Probability Based Mowse Score:

Protein score is -10*Log(P), where P is the probability that the observed match is a random event.
Protein scores greater than 61 are significant (p<0.05).

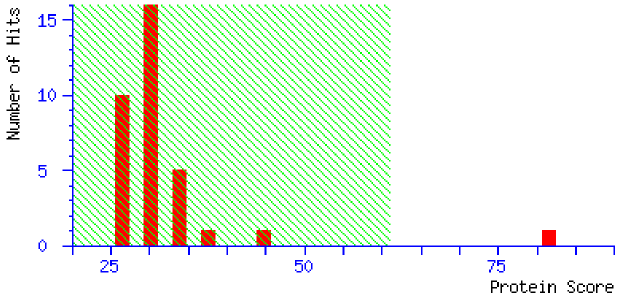

Spot No.: **92**

NCBI accession No.: **cassava4.1_004164m|PACid:17985770**

Plant species: ***Manihot esculenta***

Protein name：**malic enzyme, putative**

Mascot score: **154**

Sequence coverage %: **35** p value: **2.9e-11**

Matched peptides No.: **26** Total peptides No.: **66**

Calculated Mr: **65246** Calculated *p*I: **6.03**

Annotated PMF **spectra:**

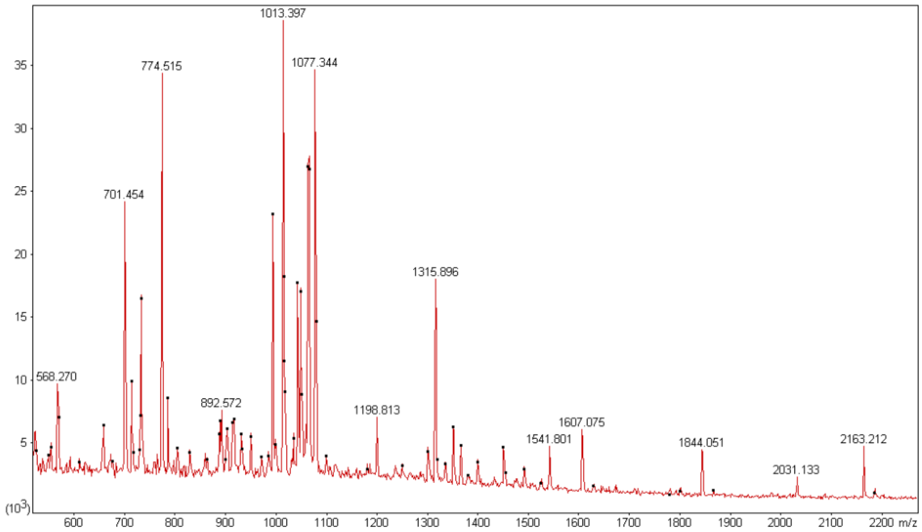

Probability Based Mowse Score:

Protein score is -10*Log(P), where P is the probability that the observed match is a random event.
Protein scores greater than 61 are significant (p<0.05).

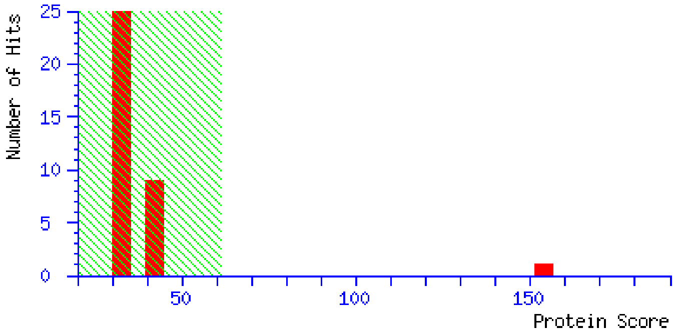

Spot No.: **93**

NCBI accession No.: **cassava4.1_014432m|PACid:17967798**

Plant species: ***Manihot esculenta***

Protein name: **Triosephosphate isomerase, cytosolic**

PFF Mascot score: **[114]** Sequence coverage %: **[23]**

Matched peptides No.: **[4]** p value: **2e-005**

Calculated Mr: 27729 Calculated *p*I: **5.77**

Annotated PMF spectra:

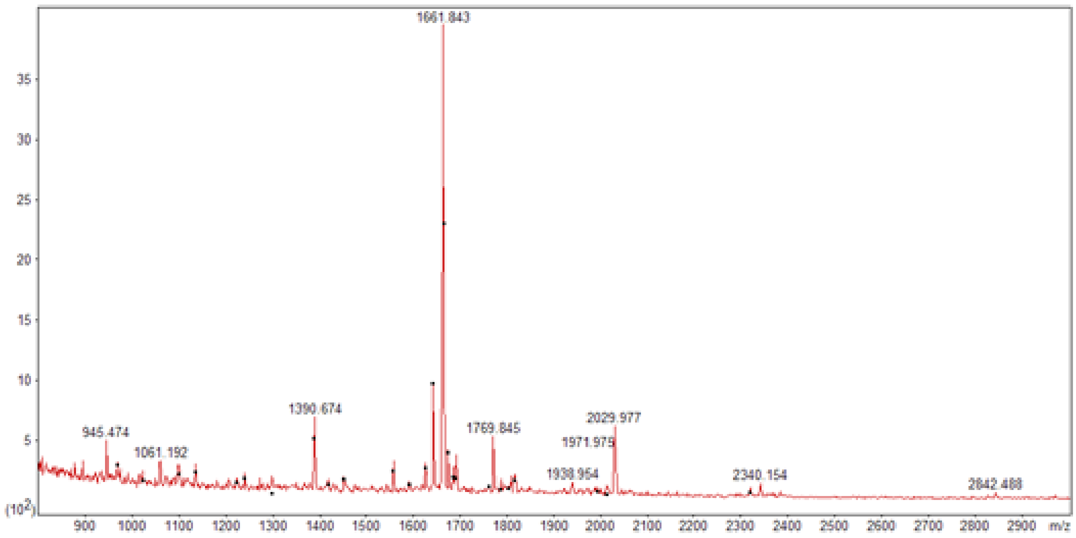

PFF Searched Score:

Ions score is -10*Log(P), where P is the probability that the observed match is a random event.
Individual ions scores > 33 indicate identity or extensive homology (p<0.05).
Protein scores are derived from ions scores as a non-probabilistic basis for ranking protein hits.

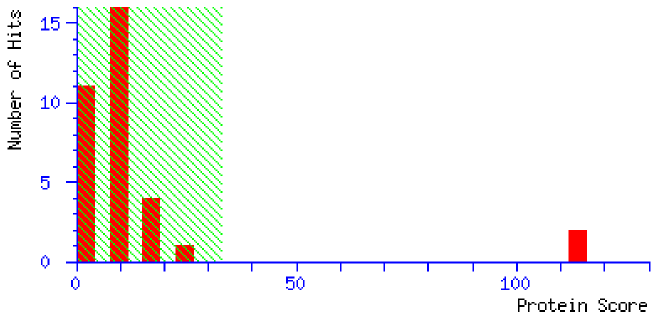

Spot No.: **94**

NCBI accession No.: **cassava4.1_002466m|PACid:17978799**

Plant species: ***Manihot esculenta***

Protein name: **Alpha-glucan phosphorylase 1**

PFF Mascot score: **[278]** Sequence coverage %: **[12]**

Matched peptides No.: **[7]** p value: **1.1e-007**

Calculated Mr: 81918 Calculated *p*I: **8.50**

Annotated PMF **spectra:**

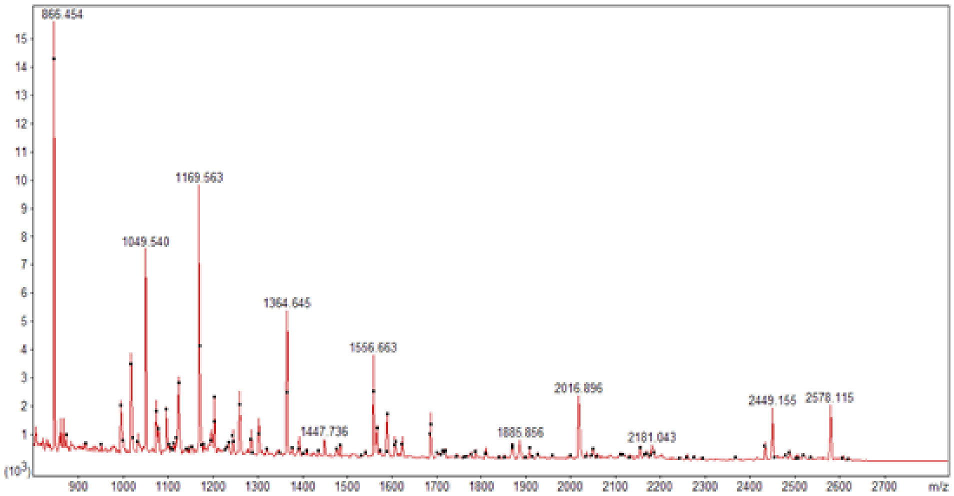

PFF Searched Score:

Ions score is -10*Log(P), where P is the probability that the observed match is a random event.
Individual ions scores > 34 indicate identity or extensive homology (p<0.05).
Protein scores are derived from ions scores as a non-probabilistic basis for ranking protein hits.

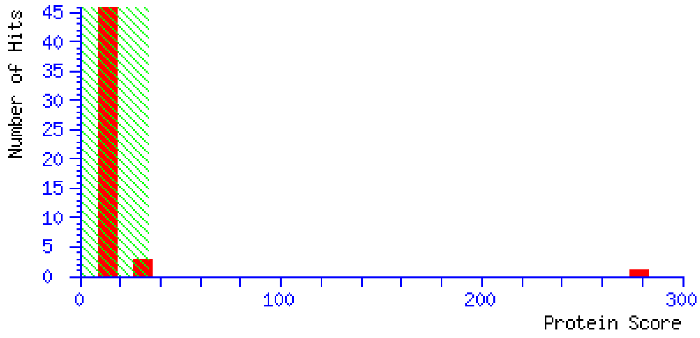

Spot No.: **95**

NCBI accession No.: **cassava4.1_009779m|PACid:17990964**

Plant species: ***Manihot esculenta***

Protein name：**Actin-7**

PFF Mascot score: **[108]** Sequence coverage %: **[9]**

Matched peptides No.: **[3]** p value: **9.7e-0086**

Calculated Mr: 41897 Calculated *p*I: **5.31**

Annotated PMF **spectra:**

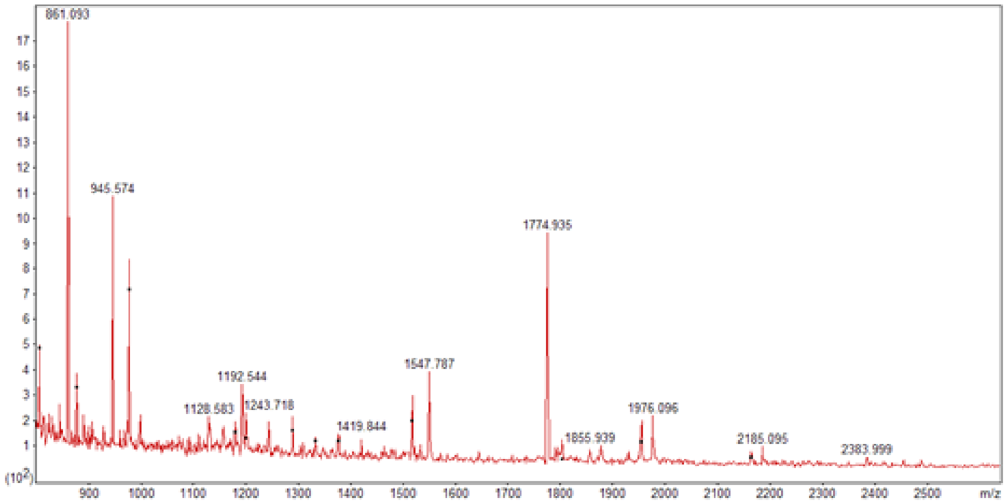

PFF Searched Score:

Ions score is -10*Log(P), where P is the probability that the observed match is a random event.
Individual ions scores > 34 indicate identity or extensive homology (p<0.05).
Protein scores are derived from ions scores as a non-probabilistic basis for ranking protein hits.

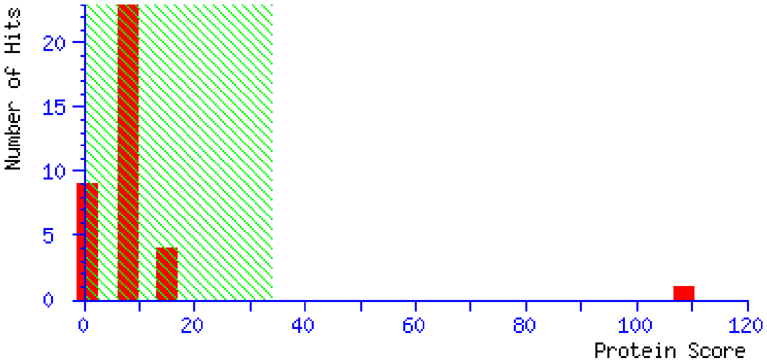

Spot No.: **96**

NCBI accession No.: **cassava4.1_009779m|PACid:17990964**

Plant species: ***Manihot esculenta***Protein name： **Actin-7**

PFF Mascot score: **[77]** Sequence coverage %: **[6]**

Matched peptides No.: **[2]** p value: **0.00032**

Calculated Mr: 41897 Calculated *p*I: **5.31**

Annotated PMF **spectra:**

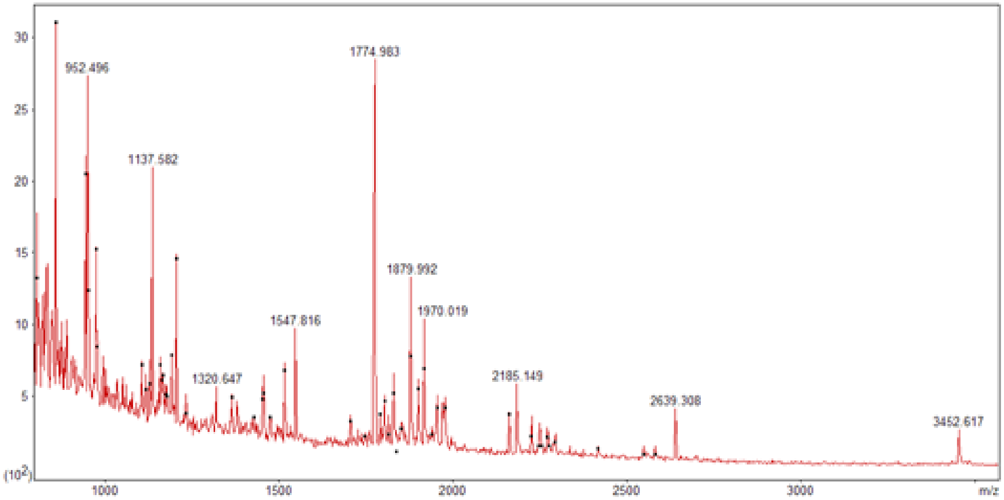

PFF Searched Score:

Ions score is -10*Log(P), where P is the probability that the observed match is a random event.
Individual ions scores > 34 indicate identity or extensive homology (p<0.05).
Protein scores are derived from ions scores as a non-probabilistic basis for ranking protein hits.

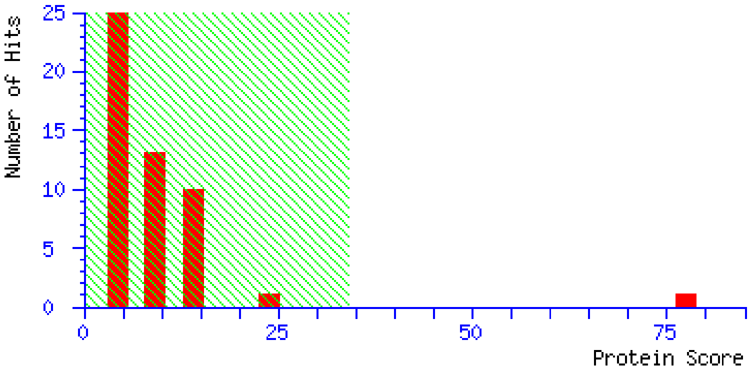

Spot No.: **97**

NCBI accession No.: **cassava4.1_011729m|PACid:17989246**

Plant species: ***Manihot esculenta***

Protein name: **nitrile-specifier protein 5-like**

PFF Mascot score: **[165]** Sequence coverage %: **[24]**

Matched peptides No.: **[7]** p value: **0.00081**

Calculated Mr: 35840 Calculated *p*I: **5.49**

Annotated PMF **spectra:**

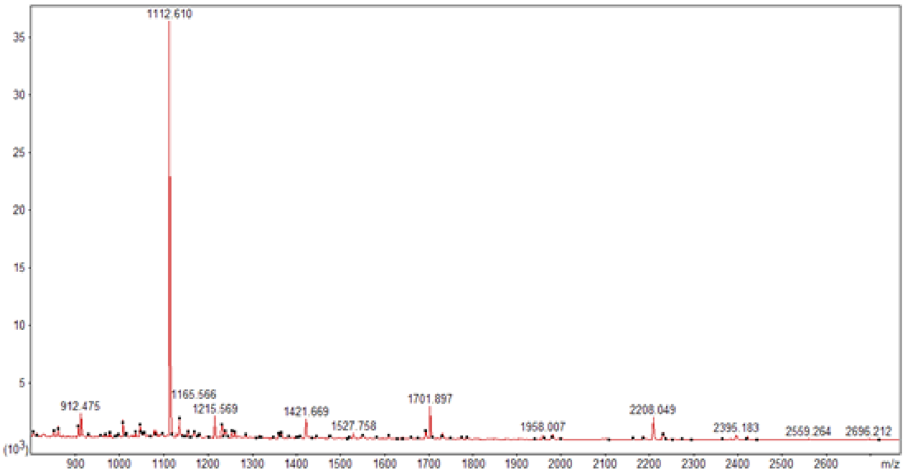

PFF Searched Score:

Ions score is -10*Log(P), where P is the probability that the observed match is a random event.
Individual ions scores > 33 indicate identity or extensive homology (p<0.05).
Protein scores are derived from ions scores as a non-probabilistic basis for ranking protein hits.

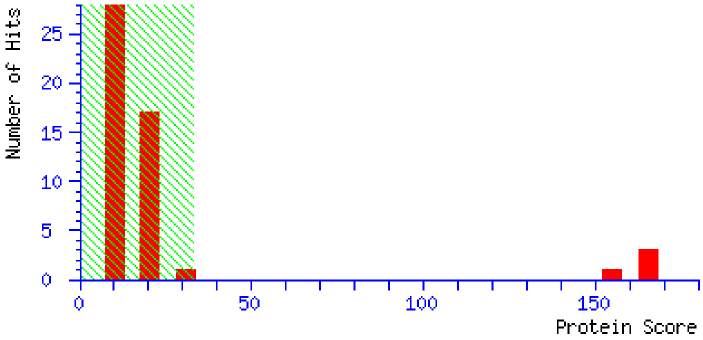

Spot No.: **98**

NCBI accession No.: **cassava4.1_016053m|PACid:17985673**

Plant species: ***Manihot esculenta***

Protein name：**Abscisic acid receptor PYR1**

PFF Mascot score: **[190]** Sequence coverage %: **[28]**

Matched peptides No.: **[4]** p value: **2.2e-006**

Calculated Mr: 23981 Calculated *p*I: **5.26**

Annotated PMF **spectra:**

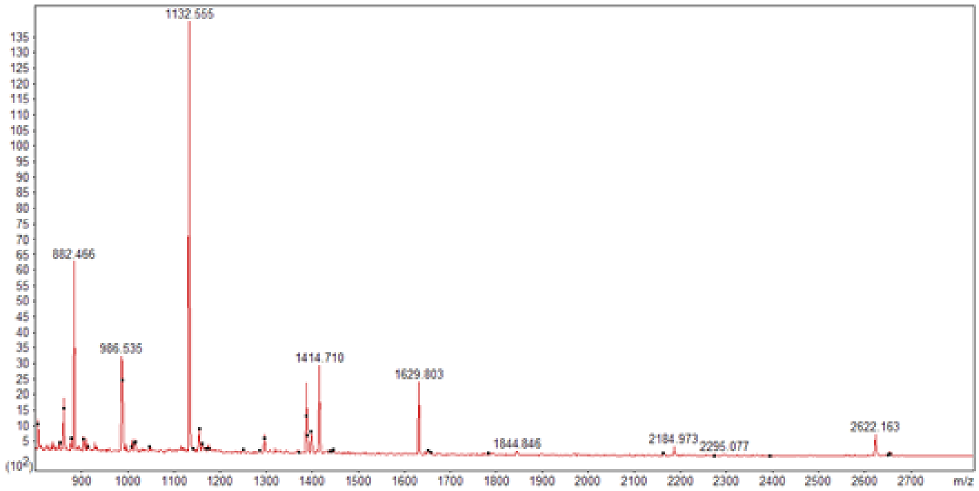

PFF Searched Score:

Ions score is -10*Log(P), where P is the probability that the observed match is a random event.
Individual ions scores > 34 indicate identity or extensive homology (p<0.05).
Protein scores are derived from ions scores as a non-probabilistic basis for ranking protein hits.

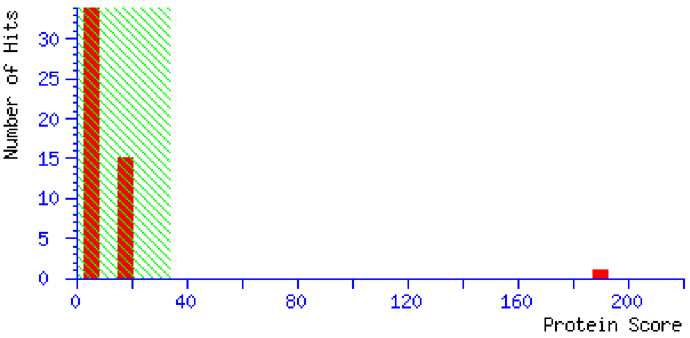

Spot No.: **99**

NCBI accession No.: **cassava4.1_012016m|PACid:17981009**

Plant species: ***Manihot esculenta***

Protein name：**Triosephosphate isomerase, chloroplastic**

PFF Mascot score: **[210]** Sequence coverage %: **[15]**

Matched peptides No.: **[4]** p value: **1.5e-007**

Calculated Mr: 34643 Calculated *p*I: **6.96**

Annotated PMF **spectra:**

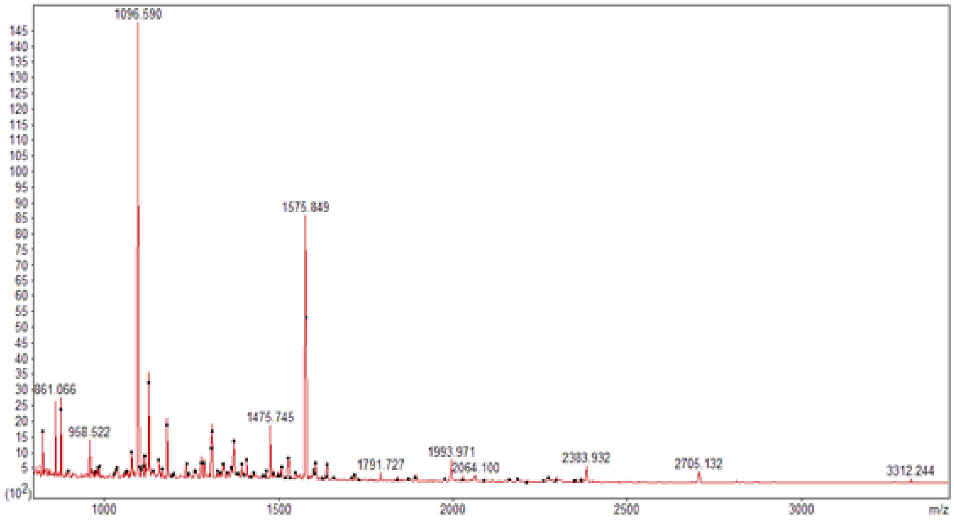

PFF Searched Score:

Ions score is -10*Log(P), where P is the probability that the observed match is a random event.
Individual ions scores > 33 indicate identity or extensive homology (p<0.05).
Protein scores are derived from ions scores as a non-probabilistic basis for ranking protein hits.

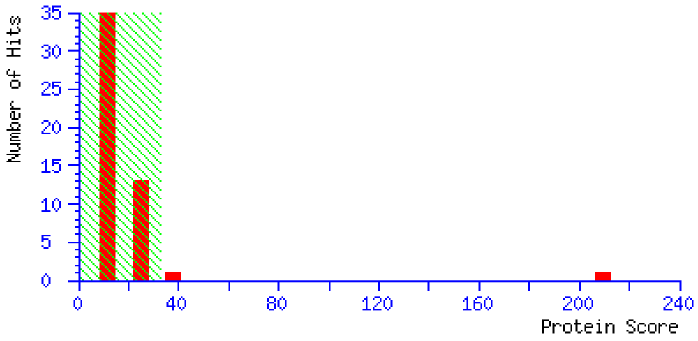

Spot No.: **100**

NCBI accession No.: **cassava4.1_013157m|PACid:17980169**

Plant species: ***Manihot esculenta***

Protein name：**Dienelactone hydrolase domain-containing protein**

PFF Mascot score: **[161]**

Sequence coverage %: **[17]**

Matched peptides No.: **[3]**

p value: **1.6e-009**

Calculated Mr: 31535

Calculated *p*I: **6.54**

Annotated PMF **spectra:**

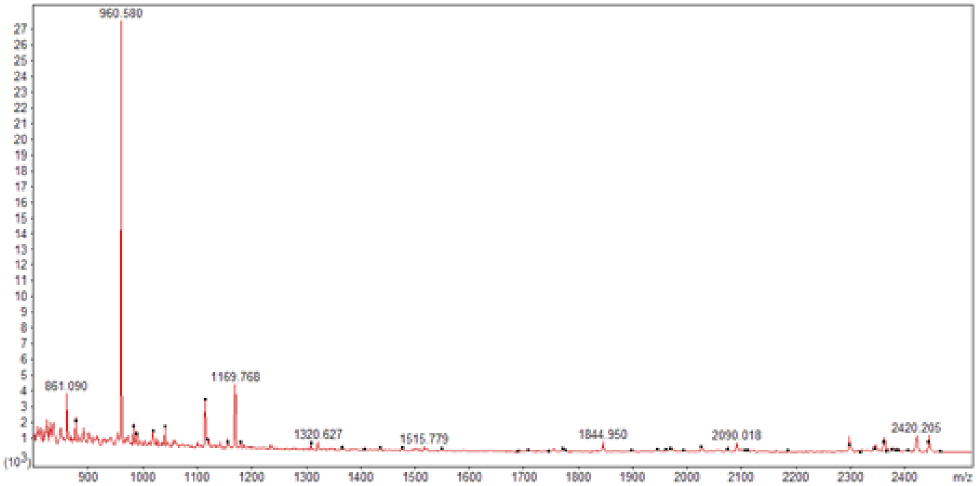

PFF Searched Score:

Ions score is -10*Log(P), where P is the probability that the observed match is a random event.
Individual ions scores > 33 indicate identity or extensive homology (p<0.05).
Protein scores are derived from ions scores as a non-probabilistic basis for ranking protein hits.

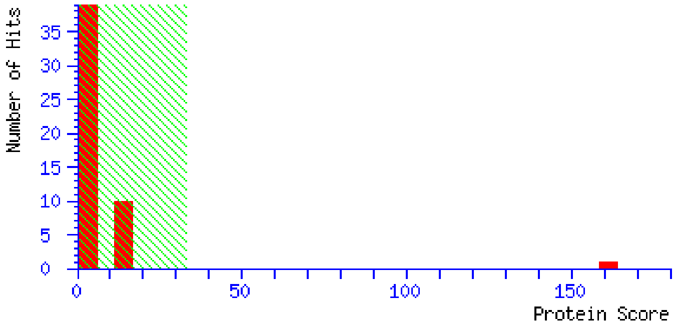

Spot No.: **101**

NCBI accession No.: **cassava4.1_016210m|PACid:17989119**

Plant species: ***Manihot esculenta***

Protein name： **UMP-CMP kinase 3**

PFF Mascot score: **[67]**　　　Sequence coverage %: **[8]**

Matched peptides No.: **[2]**　　　p value: **0.0035**

Calculated Mr: 23568　　　Calculated *p*I: **5.34**

Annotated PMF **spectra:**

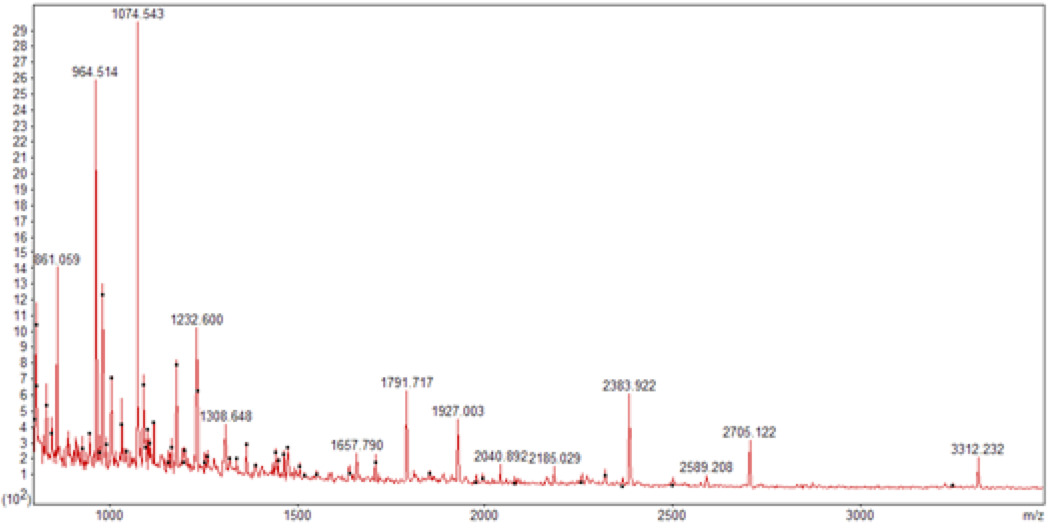

PFF Searched Score:

Ions score is -10*Log(P), where P is the probability that the observed match is a random event.
Individual ions scores > 34 indicate identity or extensive homology (p<0.05).
Protein scores are derived from ions scores as a non-probabilistic basis for ranking protein hits.

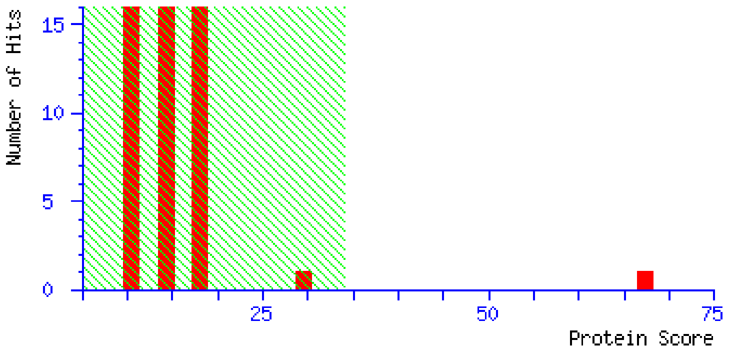

Spot No.: **102**

NCBI accession No.: **cassava4.1_009779m|PACid:17990964**

Plant species: ***Manihot esculenta***

Protein name： **Actin-7**

PFF Mascot score: **[189]** Sequence coverage %: **[24]**

Matched peptides No.: **[5]** p value: **1.6e-007**

Calculated Mr: 41897 Calculated *p*I: **5.31**

Annotated PMF **spectra:**

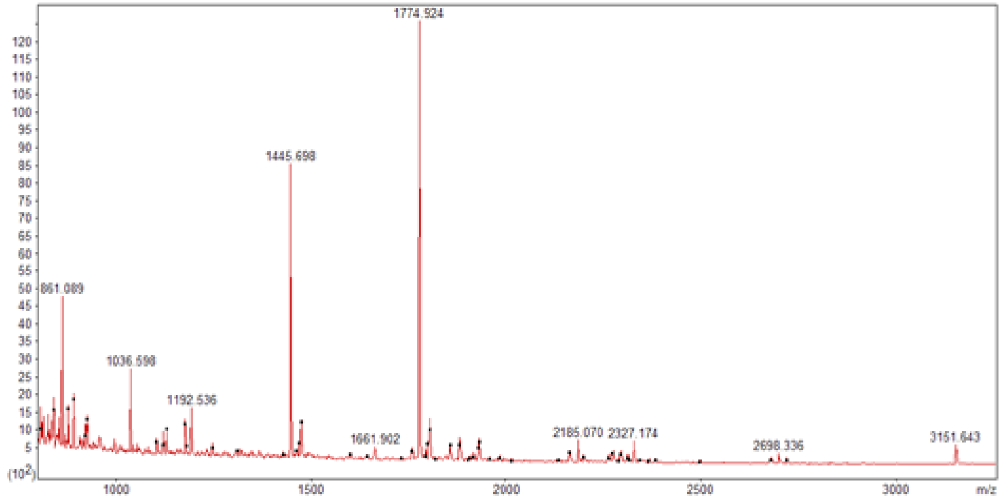

PFF Searched Score:

Ions score is -10*Log(P), where P is the probability that the observed match is a random event.
Individual ions scores > 33 indicate identity or extensive homology (p<0.05).
Protein scores are derived from ions scores as a non-probabilistic basis for ranking protein hits.

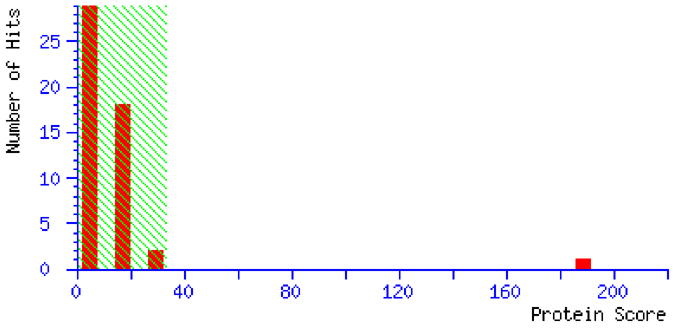

Spot No.: **103**

NCBI accession No.: **cassava4.1_016465m|PACid:17964780**

Plant species: ***Manihot esculenta***

Protein name：**Proteasome subunit beta type-3-A**

PFF Mascot score: **[158]** Sequence coverage %: **[11]**

Matched peptides No.: **[2]** p value: **1.1e-008**

Calculated Mr: 23143 Calculated *p*I: **5.33**

Annotated PMF **spectra:**

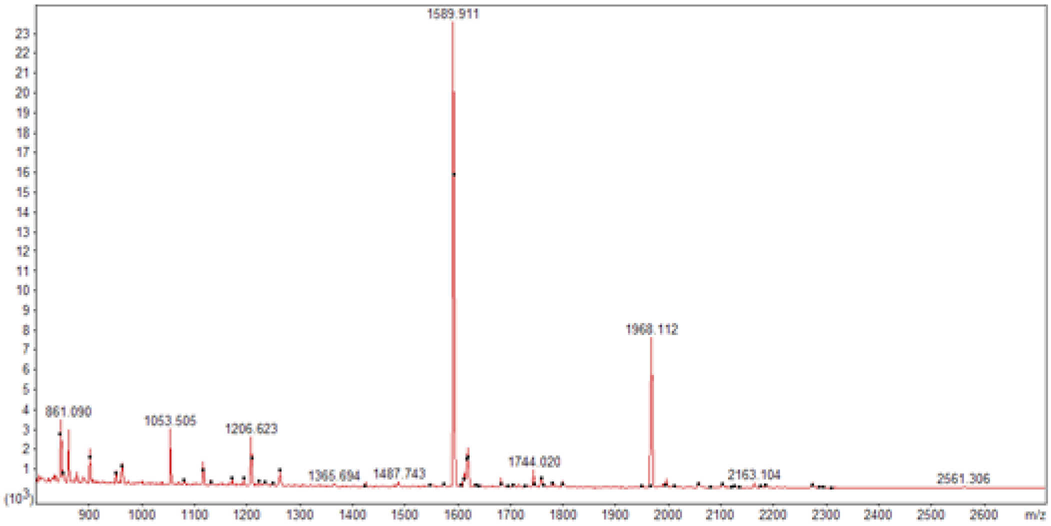

PFF Searched Score:

Ions score is -10*Log(P), where P is the probability that the observed match is a random event.
Individual ions scores > 34 indicate identity or extensive homology (p<0.05).
Protein scores are derived from ions scores as a non-probabilistic basis for ranking protein hits.

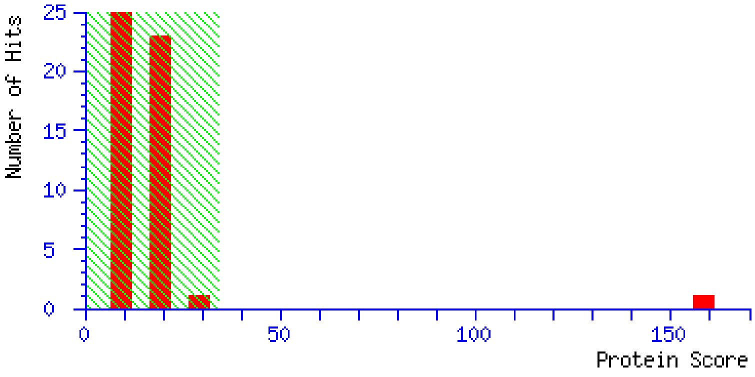

Spot No.: **104**

NCBI accession No.: **cassava4.1_018289m|PACid:17961715**

Plant species: ***Manihot esculenta***

Protein name: **Superoxide dismutase [Cu-Zn] 1**

PFF Mascot score: **[61]** Sequence coverage %: **[6]**

Matched peptides No.: **[2]** p value: **0.00011**

Calculated Mr: **15219** Calculated *p*I: **5.42**

Annotated PMF **spectra:**

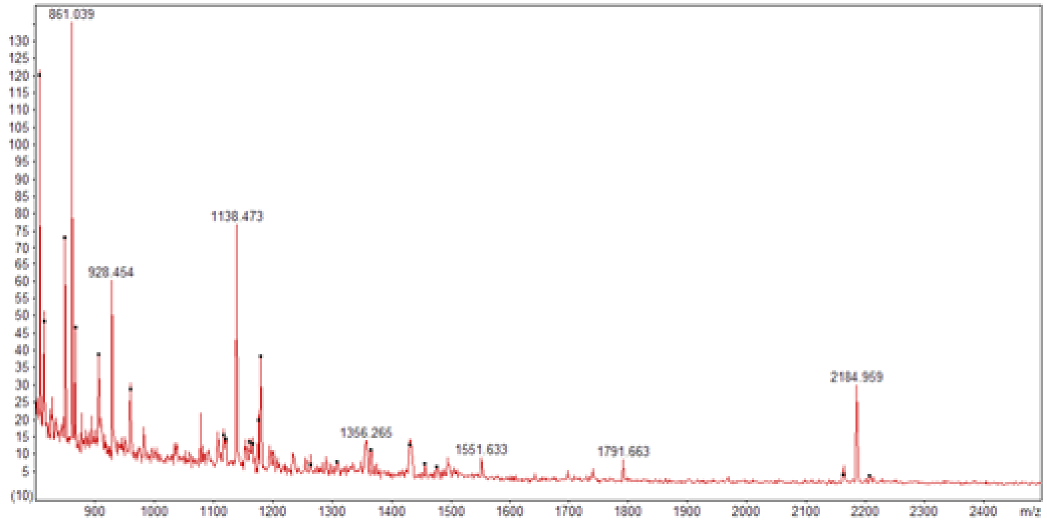

PFF Searched Score:

Ions score is -10*Log(P), where P is the probability that the observed match is a random event.
Individual ions scores > 34 indicate identity or extensive homology (p<0.05).
Protein scores are derived from ions scores as a non-probabilistic basis for ranking protein hits.

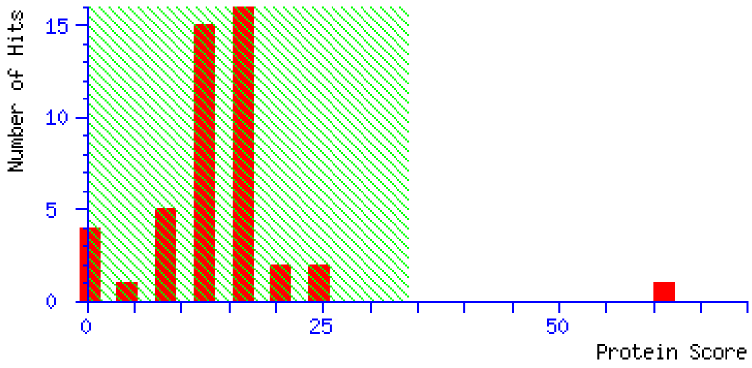

Spot No.: **105**

NCBI accession No.: **cassava4.1_017871m|PACid:17980723**

Plant species: ***Manihot esculenta***

Protein name: **18.1 kDa class I heat shock protein**

PFF Mascot score: **[142]** Sequence coverage %: **[23]**

Matched peptides No.: **[5]** p value: **0.00014**

Calculated Mr: **18750** Calculated *p*I: **7.93**

Annotated PMF **spectra:**

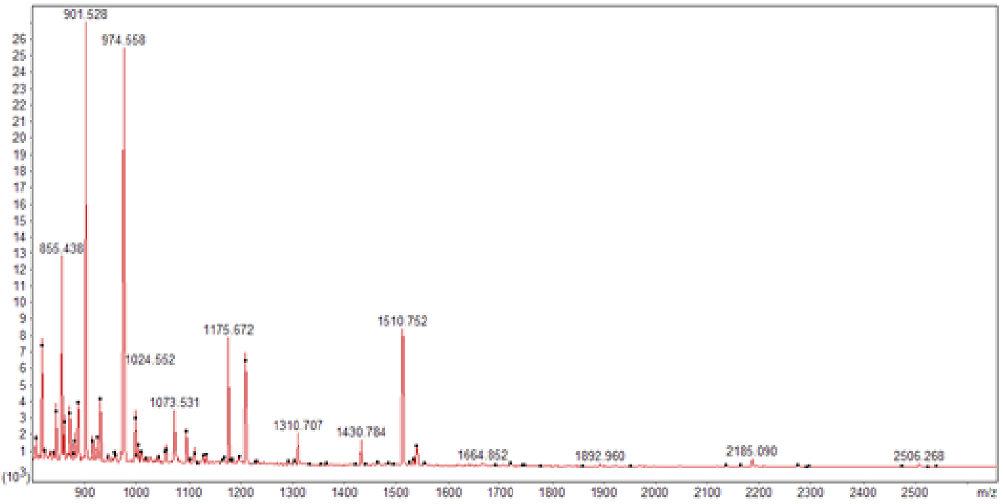

PFF Searched Score:

Ions score is -10*Log(P), where P is the probability that the observed match is a random event.
Individual ions scores > 34 indicate identity or extensive homology (p<0.05).
Protein scores are derived from ions scores as a non-probabilistic basis for ranking protein hits.

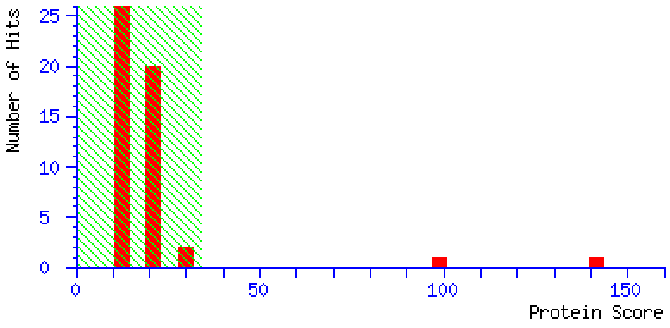

Spot No.: **106**

NCBI accession No.: **cassava4.1_018294m|PACid:17966054**

Plant species: ***Manihot esculenta***

Protein name：**Superoxide dismutase [Cu-Zn] 1**

PFF Mascot score: **[130]**   Sequence coverage %: **[23]**

Matched peptides No.: **[3]**   p value: **0.00011**

Calculated Mr: 15416   Calculated *p*I: **5.85**

Annotated PMF **spectra:**

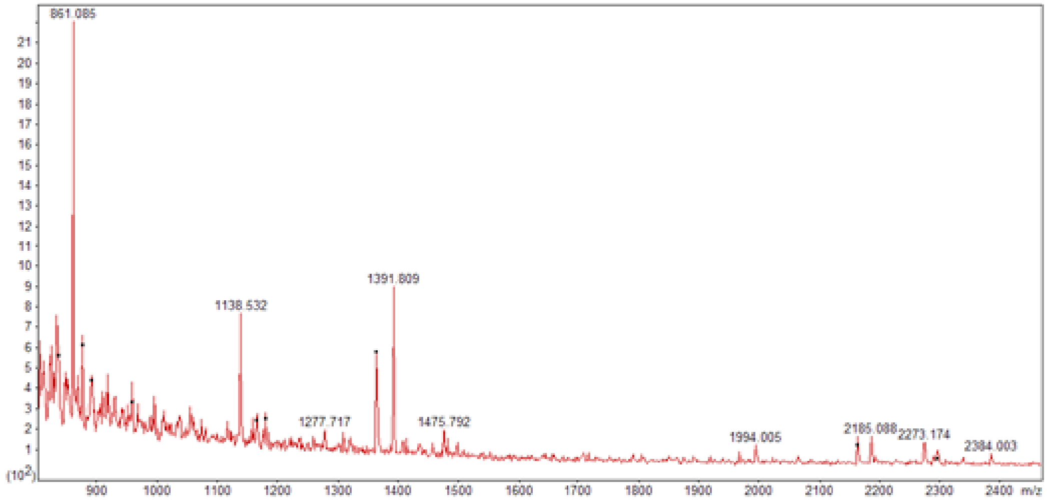

PFF Searched Score:

Ions score is -10*Log(P), where P is the probability that the observed match is a random event.
Individual ions scores > 34 indicate identity or extensive homology (p<0.05).
Protein scores are derived from ions scores as a non-probabilistic basis for ranking protein hits.

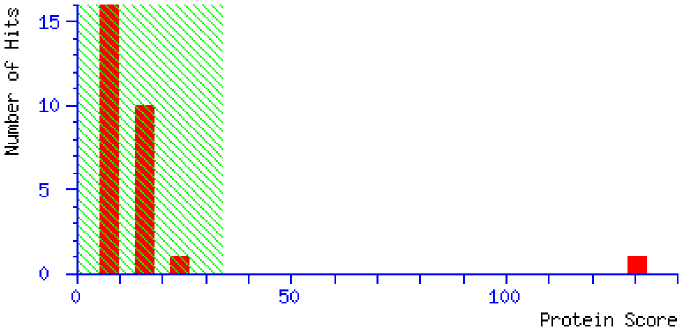

Spot No.: **107**

NCBI accession No.: **cassava4.1_016175m|PACid:17993085**

Plant species: ***Manihot esculenta***

Protein name： **glutathione S-transferase GST, putative**

PFF Mascot score: **[257]**　　Sequence coverage %: **[27]**

Matched peptides No.: **[5]**　　p value: **1.9e-009**

Calculated Mr: 24372　　Calculated *p*I: **6.25**

Annotated PMF **spectra:**

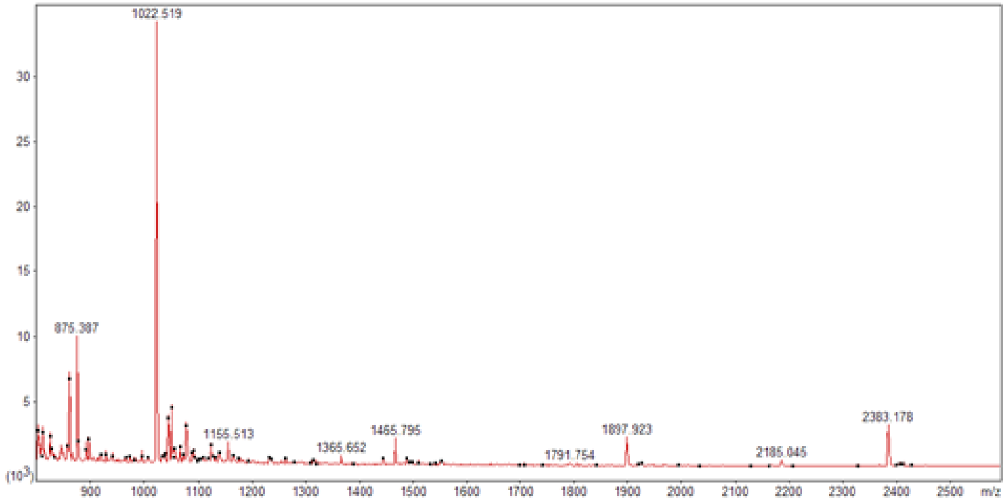

PFF Searched Score:

Ions score is -10*Log(P), where P is the probability that the observed match is a random event.
Individual ions scores > 34 indicate identity or extensive homology (p<0.05).
Protein scores are derived from ions scores as a non-probabilistic basis for ranking protein hits.

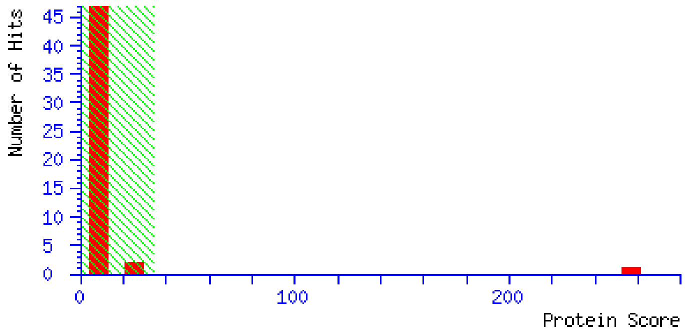

Spot No.: **108**

NCBI accession No.: **cassava4.1_014432m|PACid:17967798**

Plant species: ***Manihot esculenta***

Protein name：**Triosephosphate isomerase, cytosolic**

PFF Mascot score: **[166]** Sequence coverage %: **[16]**

Matched peptides No.: **[3]** p value: **0.00021**

Calculated Mr: **27729** Calculated *p*I: **5.77**

Annotated PMF **spectra:**

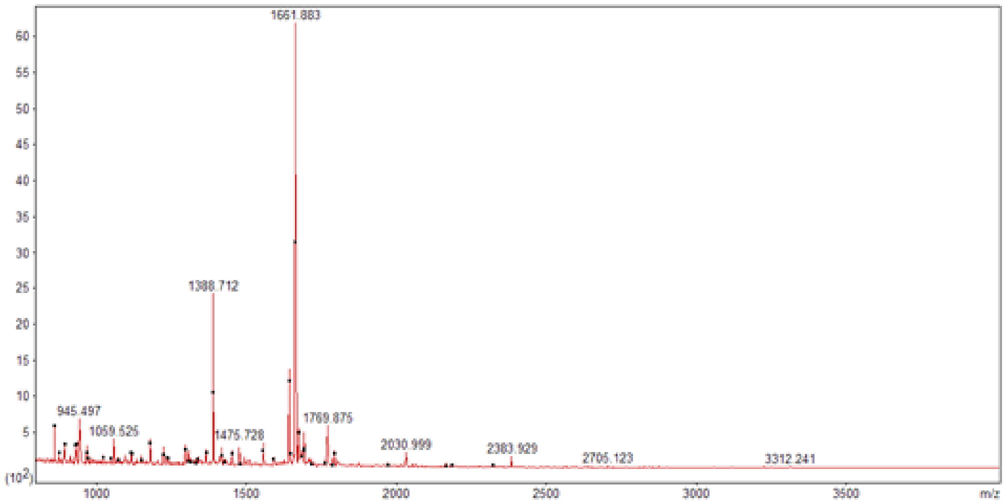

PFF Searched Score:

Ions score is -10*Log(P), where P is the probability that the observed match is a random event.
Individual ions scores > 34 indicate identity or extensive homology (p<0.05).
Protein scores are derived from ions scores as a non-probabilistic basis for ranking protein hits.

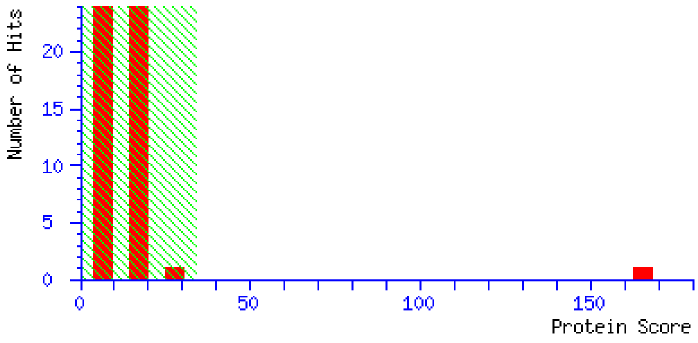

Spot No.: **109**

NCBI accession No.: **cassava4.1_011868m|PACid:17990035**

Plant species: ***Manihot esculenta***

Protein name：**6-phosphogluconolactonase**

PFF Mascot score: **[77]** Sequence coverage %: **[5]**

Matched peptides No.: **[2]** p value: **0.0018**

Calculated Mr: **35492** Calculated *p*I: **8.07**

Annotated PMF **spectra:**

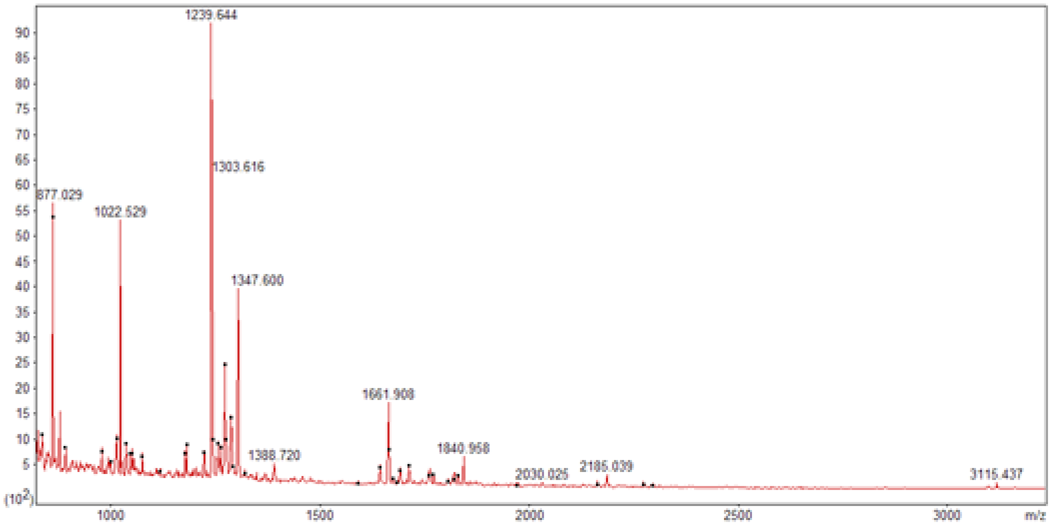

PFF Searched Score:

Ions score is -10*Log(P), where P is the probability that the observed match is a random event.
Individual ions scores > 33 indicate identity or extensive homology (p<0.05).
Protein scores are derived from ions scores as a non-probabilistic basis for ranking protein hits.

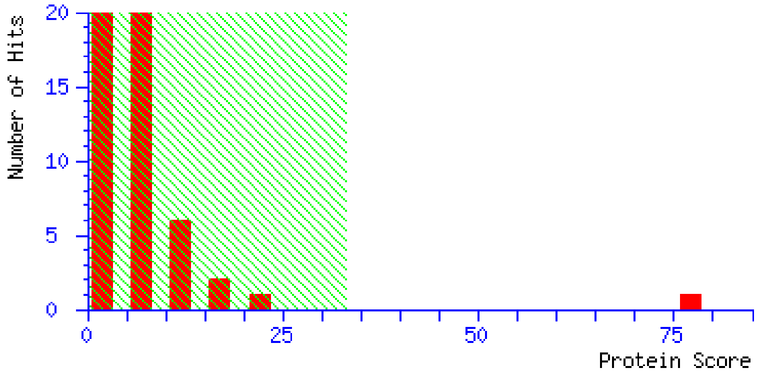

Spot No.: **110**

NCBI accession No.: **cassava4.1_014432m|PACid:17967798**

Plant species: ***Manihot esculenta***

Protein name：**Triosephosphate isomerase, cytosolic**

PFF Mascot score: **[298]** Sequence coverage %: **[31]**

Matched peptides No.: **[7]** p value: **2.3e-006**

Calculated Mr: 27729 Calculated *p*I: **5.77**

Annotated PMF **spectra:**

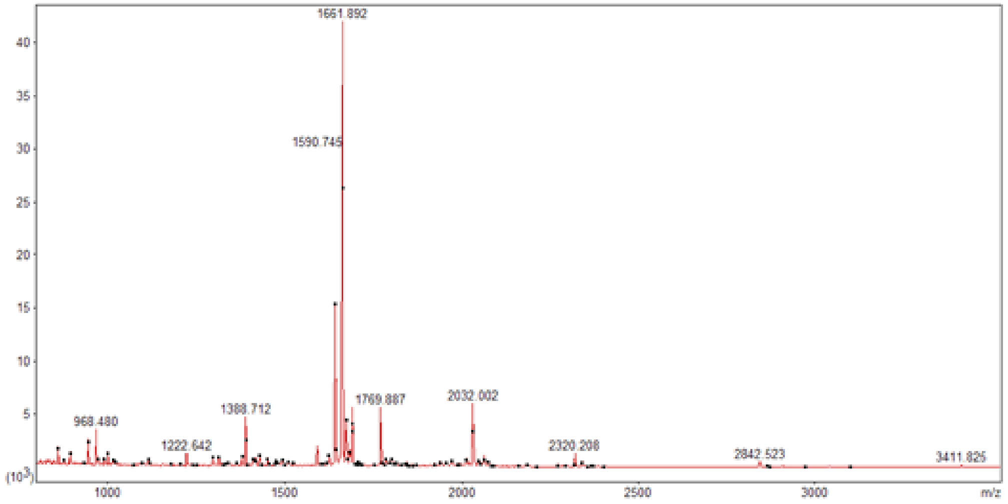

PFF Searched Score:

Ions score is -10*Log(P), where P is the probability that the observed match is a random event.
Individual ions scores > 33 indicate identity or extensive homology (p<0.05).
Protein scores are derived from ions scores as a non-probabilistic basis for ranking protein hits.

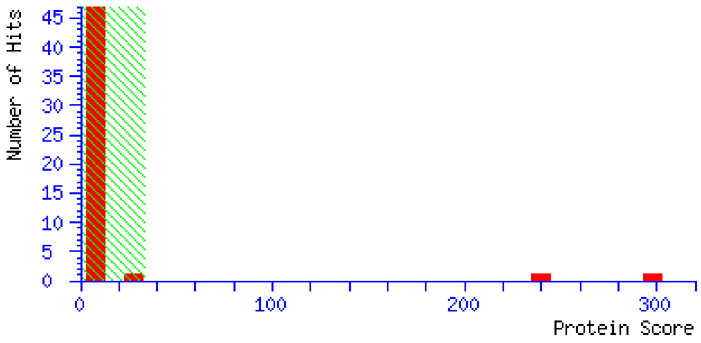

Spot No.: **111**

NCBI accession No.: **cassava4.1_021183m|PACid:17971835**

Plant species: ***Ricinus communis***

Protein name： **annexin**

PFF Mascot score: **[298]** Sequence coverage %: **[21]**

Matched peptides No.: **[6]** p value: **0.000000042**

Calculated Mr: 33276 Calculated *p*I: **6.04**

Annotated PMF **spectra:**

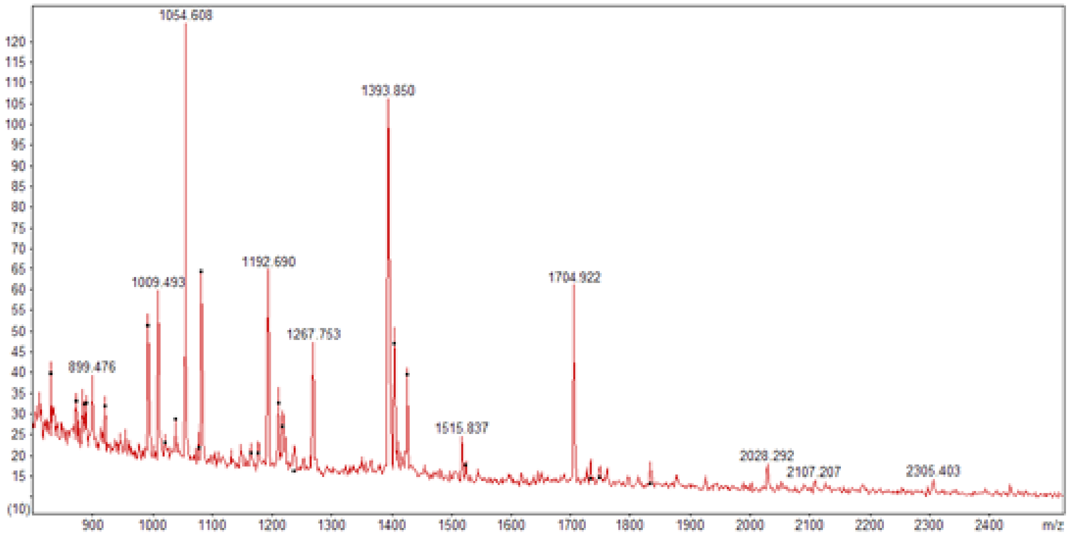

PFF Searched Score:

Ions score is -10*Log(P), where P is the probability that the observed match is a random event.
Individual ions scores > 34 indicate identity or extensive homology (p<0.05).
Protein scores are derived from ions scores as a non-probabilistic basis for ranking protein hits.

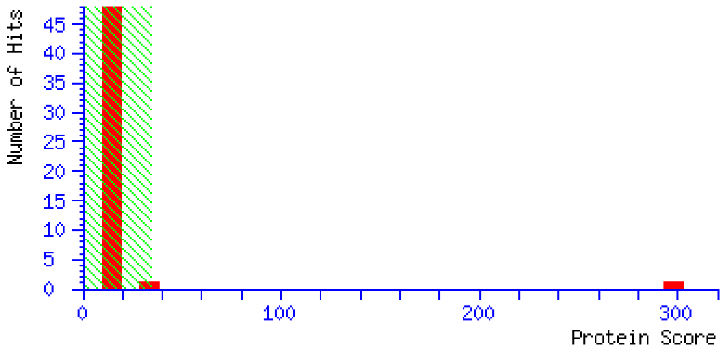

Spot No.: **112**

NCBI accession No.: **cassava4.1_010344m|PACid:17990491**

Plant species: ***Manihot esculenta***

Protein name：**alcohol dehydrogenase, putative**

PFF Mascot score: **[343]** Sequence coverage %: **[8]**

Matched peptides No.: **[2]** p value: **3.9e-011**

Calculated Mr: 39515 Calculated *p*I: **6.15**

Annotated PMF **spectra:**

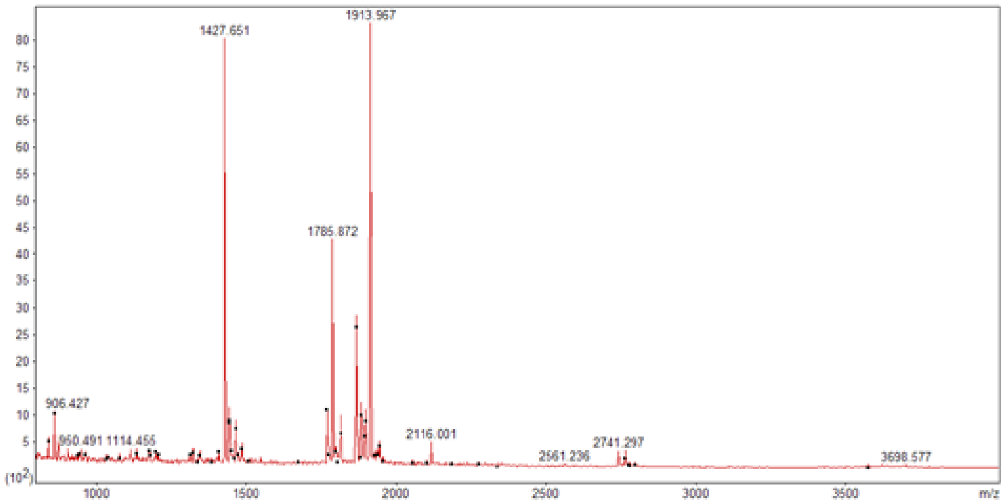

PFF Searched Score:

Ions score is -10*Log(P), where P is the probability that the observed match is a random event.
Individual ions scores > 32 indicate identity or extensive homology (p<0.05).
Protein scores are derived from ions scores as a non-probabilistic basis for ranking protein hits.

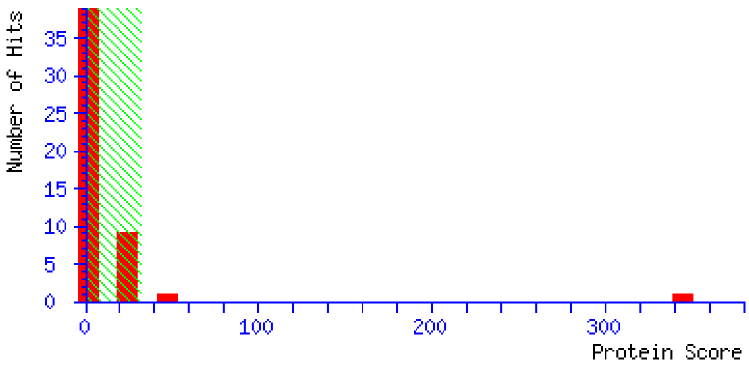

Spot No.: **113**

NCBI accession No.: **cassava4.1_009779m|PACid:17990964**

Plant species: ***Manihot esculenta***

Protein name: **Actin-7**

PFF Mascot score: **[440]** Sequence coverage %: **[36]**

Matched peptides No.: **[9]** p value: **2.9e-012**

Calculated Mr: 41897 Calculated *p*I: **5.31**

Annotated PMF **spectra:**

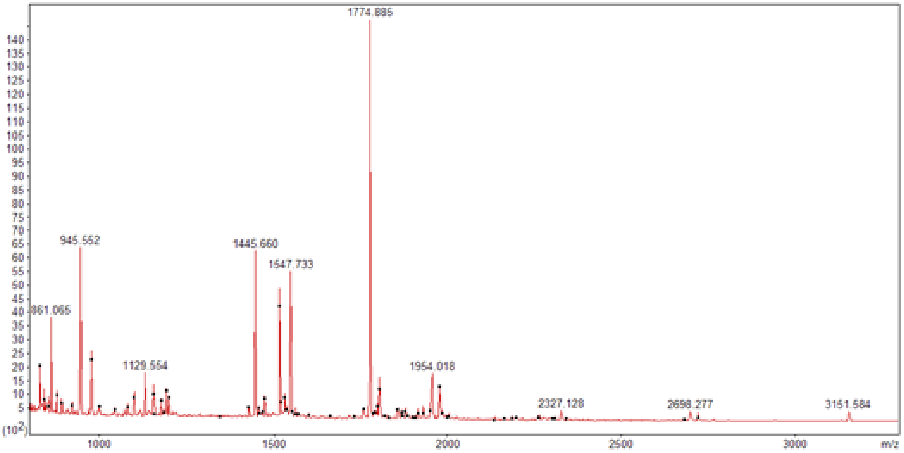

PFF Searched Score:

Ions score is -10*Log(P), where P is the probability that the observed match is a random event.
Individual ions scores > 33 indicate identity or extensive homology (p<0.05).
Protein scores are derived from ions scores as a non-probabilistic basis for ranking protein hits.

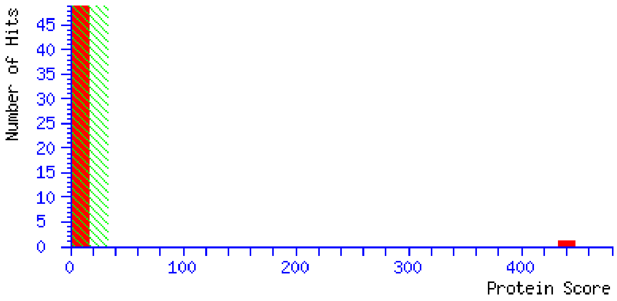

Spot No.: **114**

NCBI accession No.: **cassava4.1_010105m|PACid:17962148**

Plant species: ***Manihot esculenta***

Protein name： **Malate dehydrogenase, cytoplasmic 1**

PFF Mascot score: **[349]** Sequence coverage %: **[20]**

Matched peptides No.: **[6]** p value: **1.4e-006**

Calculated Mr: 40924 Calculated *p*I: **7.08**

Annotated PMF **spectra:**

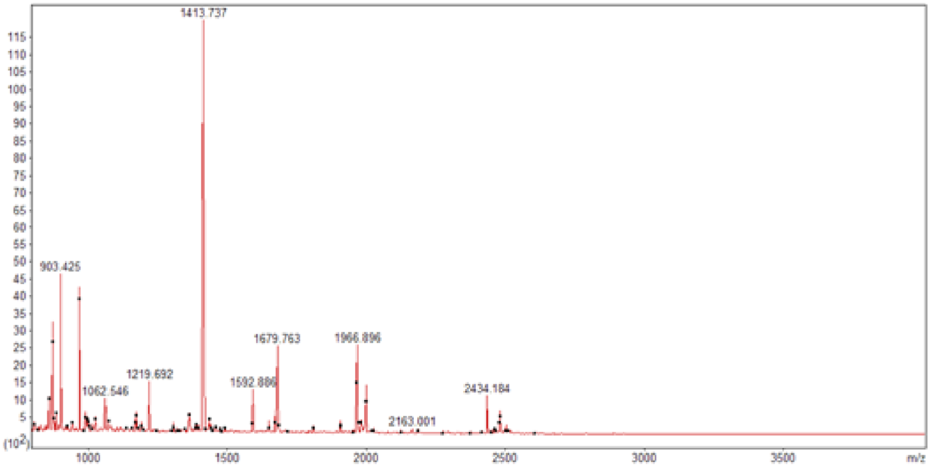

PFF Searched Score:

Ions score is -10*Log(P), where P is the probability that the observed match is a random event.
Individual ions scores > 34 indicate identity or extensive homology (p<0.05).
Protein scores are derived from ions scores as a non-probabilistic basis for ranking protein hits.

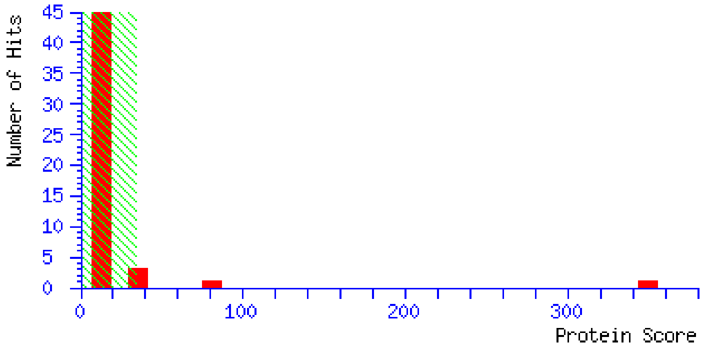

Spot No.: **115**

NCBI accession No.: **cassava4.1_004280m|PACid:17986008**

Plant species: ***Manihot esculenta***

Protein name： **Leucine aminopeptidase 3, chloroplastic**

PFF Mascot score: **[399]** Sequence coverage %: **[9]**

Matched peptides No.: **[4]** p value: **2.5e-010**

Calculated Mr: 61349 Calculated *p*I: **6.69**

Annotated PMF **spectra:**

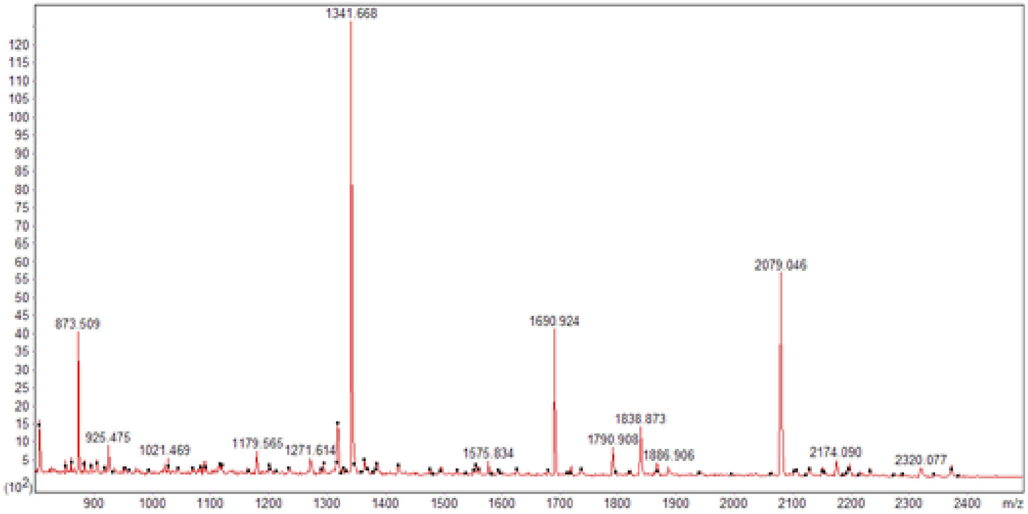

PFF Searched Score:

Ions score is -10*Log(P), where P is the probability that the observed match is a random event.
Individual ions scores > 33 indicate identity or extensive homology (p<0.05).
Protein scores are derived from ions scores as a non-probabilistic basis for ranking protein hits.

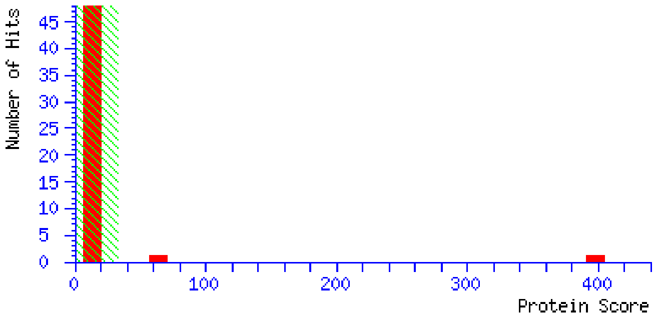

Spot No.: **116**

NCBI accession No.: **cassava4.1_007673m|PACid:17979089**

Plant species: ***Manihot esculenta***

Protein name: **Bifunctional enolase 2/transcriptional activator**

PFF Mascot score: **[173]** Sequence coverage %: **[9]**

Matched peptides No.: **[4]** p value: **1.3e-008**

Calculated Mr: **48058** Calculated *p*I: **5.86**

Annotated PMF **spectra:**

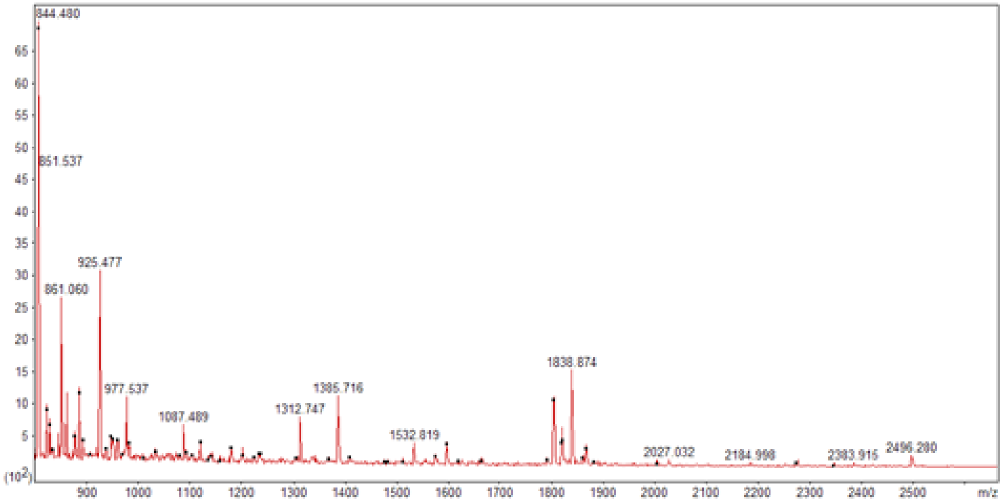

PFF Searched Score:

Ions score is -10*Log(P), where P is the probability that the observed match is a random event.
Individual ions scores > 34 indicate identity or extensive homology (p<0.05).
Protein scores are derived from ions scores as a non-probabilistic basis for ranking protein hits.

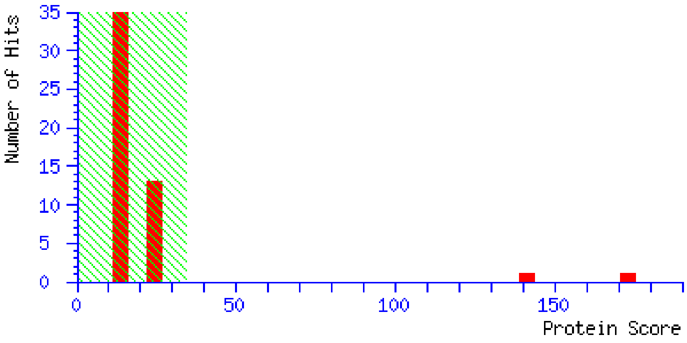

Spot No.: **117**

NCBI accession No.: **cassava4.1_006024m|PACid:17974163**

Plant species: ***Manihot esculenta***

Protein name: **Betaine aldehyde dehydrogenase 1, chloroplastic**

PFF Mascot score: **[345]** Sequence coverage %: **[18]**

Matched peptides No.: **[5]** p value: **6.9e-013**

Calculated Mr: 55678 Calculated *p*I: **5.48**

Annotated PMF **spectra:**

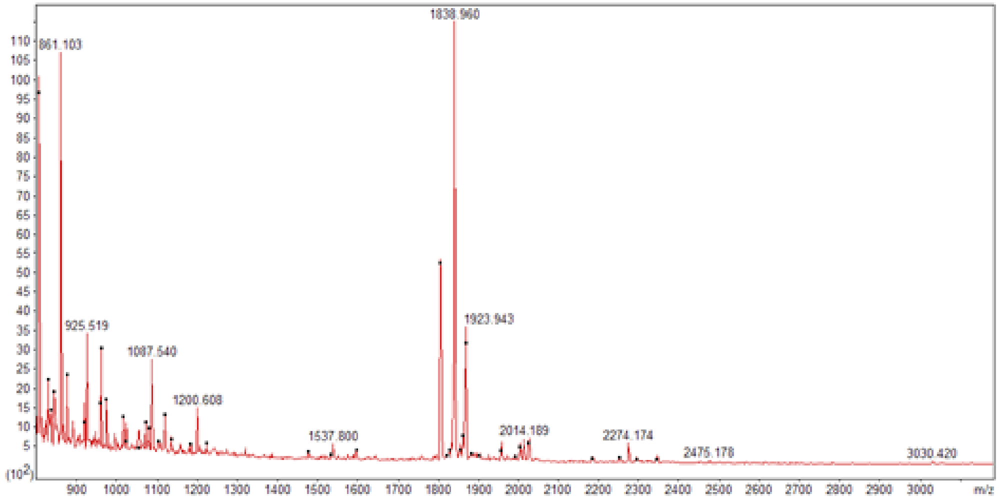

PFF Searched Score:

Ions score is -10*Log(P), where P is the probability that the observed match is a random event.
Individual ions scores > 33 indicate identity or extensive homology (p<0.05).
Protein scores are derived from ions scores as a non-probabilistic basis for ranking protein hits.

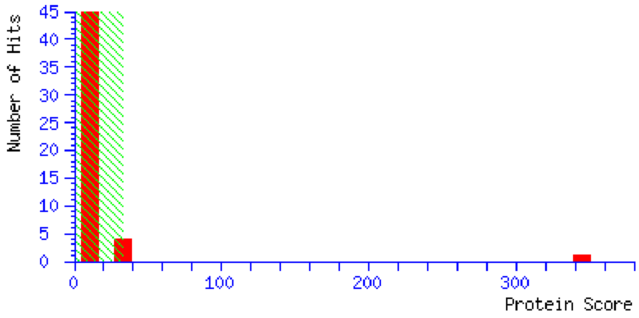

Spot No.: **118**

NCBI accession No.: **cassava4.1_005314m|PACid:17981128**

Plant species: ***Manihot esculenta***

Protein name: **Ketol-acid reductoisomerase, chloroplastic**

PFF Mascot score: **[214]** Sequence coverage %: **[9]**

Matched peptides No.: **[4]** p value: **9.6e-005**

Calculated Mr: **58515** Calculated *p*I: **5.50**

Annotated PMF **spectra:**

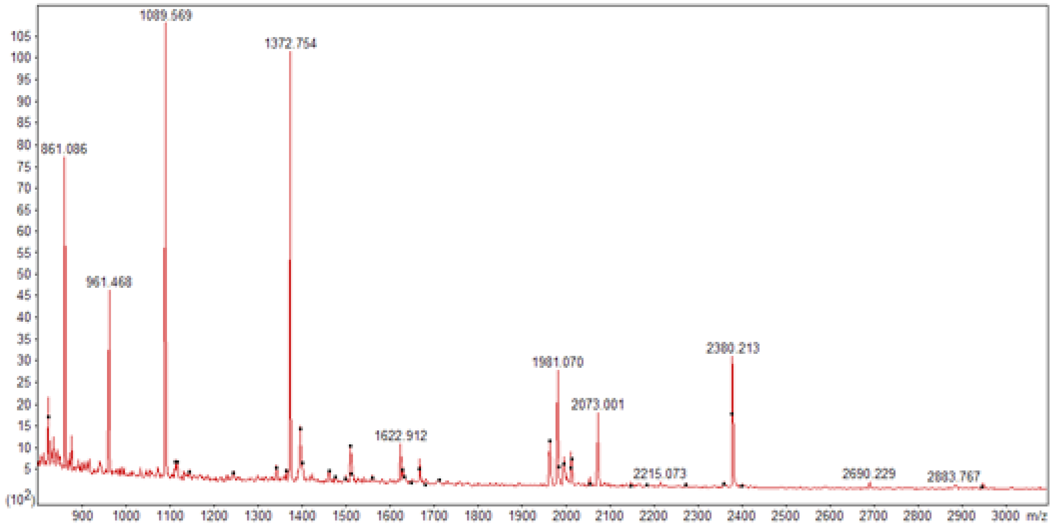

PFF Searched Score:

Ions score is -10*Log(P), where P is the probability that the observed match is a random event.
Individual ions scores > 33 indicate identity or extensive homology (p<0.05).
Protein scores are derived from ions scores as a non-probabilistic basis for ranking protein hits.

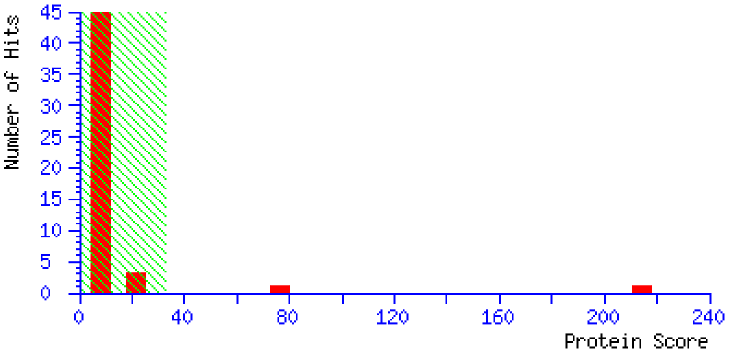

Supplemental Figure S3:

Supplemental spectra and MALDI TOF/TOF MS/MS identification information

Annotated spectra for the 80 proteins on DIGE gels identified by PMF and PFF.

CID: collision induced dissociation

MALDI TOF:

matrix assisted laser desorption/ionization time of flight

MS: mass spectrometry

PMF: peptide mass fingerprinting

PFF: peptide fragment fingerprinting

Spot No.: **2**

NCBI accession No.: **cassava4.1_004263m|PACid:17968763**

Protein name: **Chaperonin 60 subunit alpha 1, chloroplastic**

Plant species: ***Manihot esculenta***

Mascot score: **215**

Sequence coverage %: **47**

p value: **2.3e-017**

Matched peptides No.: **29**

Total peptides No.: **64**

Calculated Mr: **62302**

Calculated *p*I: **5.34**

Annotated PMF **spectra:**

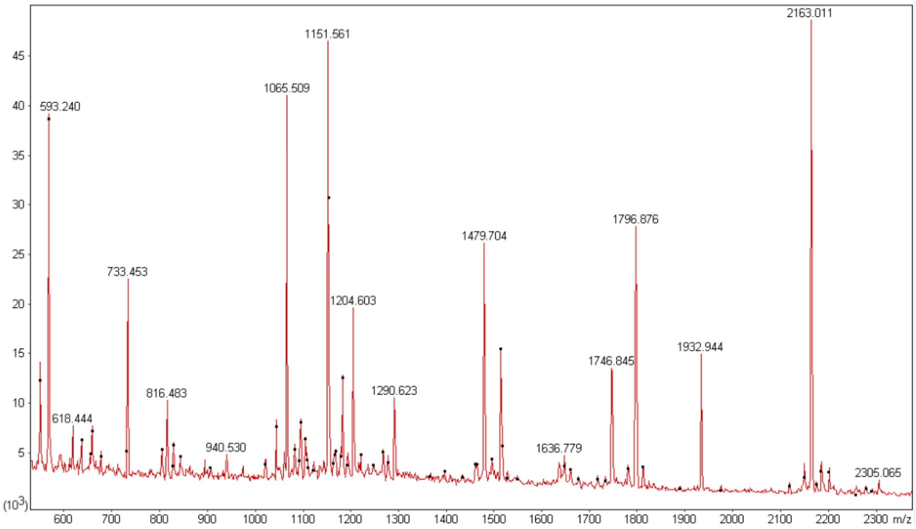

Probability Based Mowse Score:

Protein score is -10*Log(P), where P is the probability that the observed match is a random event.
Protein scores greater than 61 are significant (p<0.05).

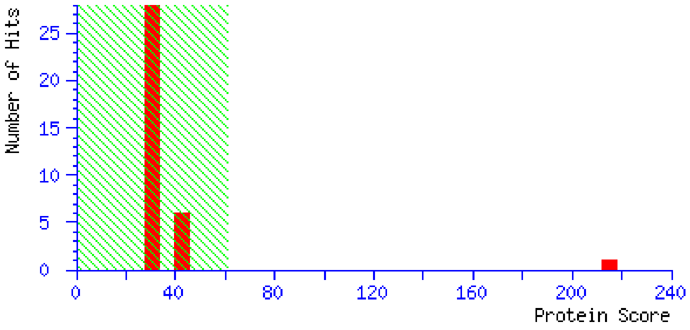

Spot No.: **5**

NCBI accession No.: **cassava4.1_007971m|PACid:17991617**

Protein name：**Tubulin alpha-6 chain**

Plant species: ***Manihot esculenta*** Mascot score: **101**

Sequence coverage %: **51** p value: **5.8e-006**

Matched peptides No.: **15** Total peptides No.: **65**

Calculated Mr: **48294** Calculated *p*I: **4.95**

Annotated PMF **spectra:**

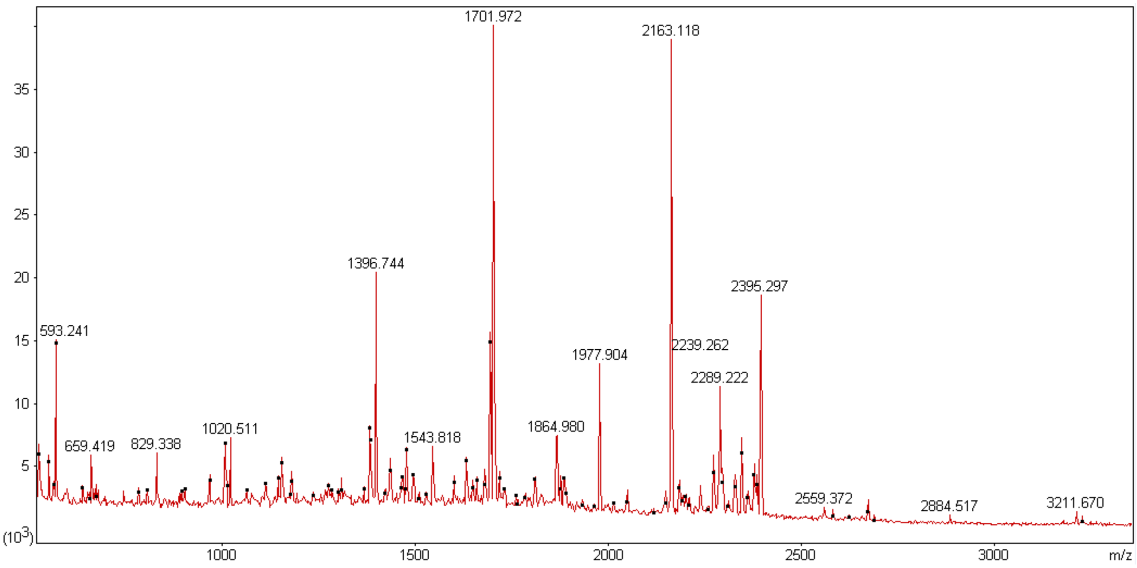

Probability Based Mowse Score:

Protein score is -10*Log(P), where P is the probability that the observed match is a random event. Protein scores greater than 61 are significant (p<0.05).

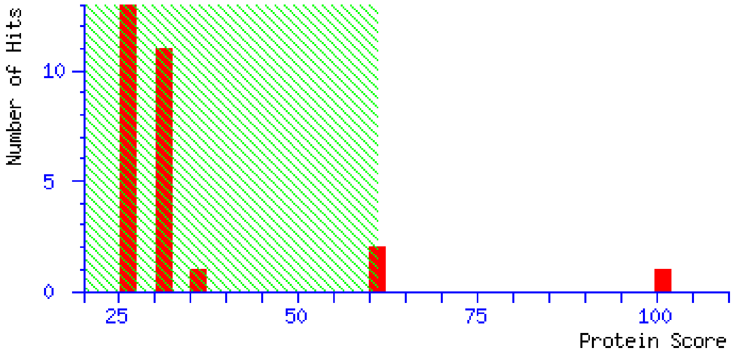

Spot No.: **6**

NCBI accession No.: **cassava4.1_008310m|PACid:17966609**

Protein name：**26S protease regulatory subunit 6A homolog A**

Plant species: ***Manihot esculenta*** Mascot score: **154**

Sequence coverage %: **59** p value: **2.9e-11**

Matched peptides No.: **29** Total peptides No.: **93**

Calculated Mr: **47700** Calculated *p*I: **4.99**

Annotated PMF **spectra:**

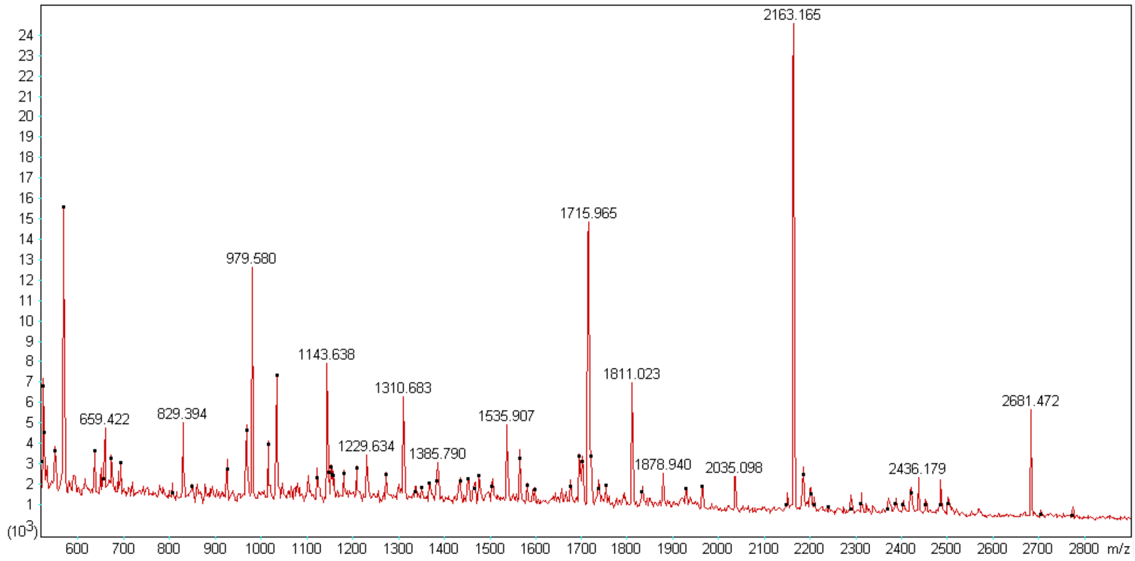

Probability Based Mowse Score:

Protein score is -10*Log(P), where P is the probability that the observed match is a random event. Protein scores greater than 61 are significant (p<0.05).

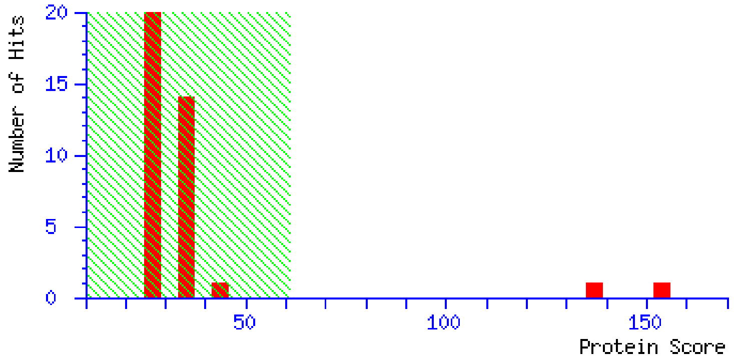

Spot No.: **15**

NCBI accession No.: **cassava4.1_007971m|PACid:17991617**

Protein name：**Tubulin alpha-6 chain**

Plant species: ***Manihot esculenta*** Mascot score: **103**

Sequence coverage %: **48** p value: 3. 7e−006

Matched peptides No.: **18** Total peptides No.: **79**

Calculated Mr: **48294** Calculated *p*I: **4.95**

Annotated PMF **spectra:**

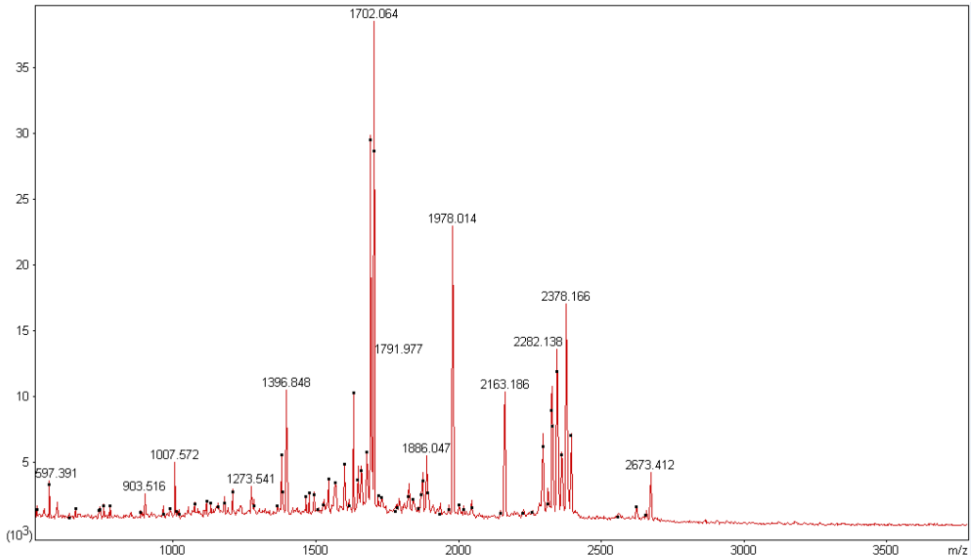

Probability Based Mowse Score:

Protein score is -10*Log(P), where P is the probability that the observed match is a random event. Protein scores greater than 61 are significant (p<0.05).

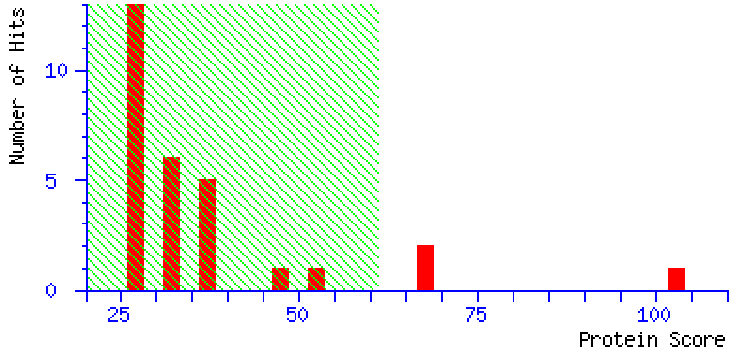

Spot No.: **21**

NCBI accession No.: **cassava4.1_008086m|PACid:17989319**

Plant species: ***Manihot esculenta***

Protein name: **Glutamine synthetase cytosolic isozyme 1-1**

Mascot score: **101**

Sequence coverage %: **33** p value: **5.8e-006**

Matched peptides No.: **14** Total peptides No.: **54**

Calculated Mr: **47502** Calculated *p*I: **6.27**

Annotated PMF **spectra:**

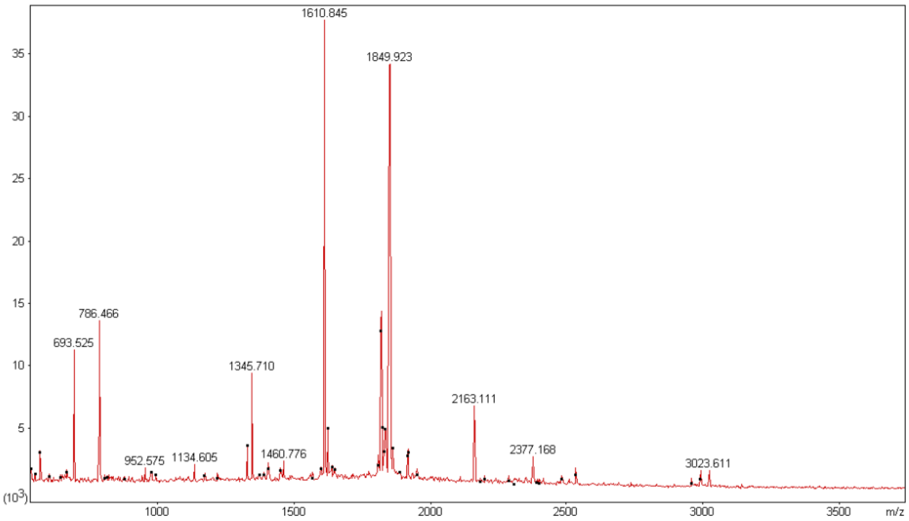

Probability Based Mowse Score:

Protein score is -10*Log(P), where P is the probability that the observed match is a random event. Protein scores greater than 61 are significant (p<0.05).

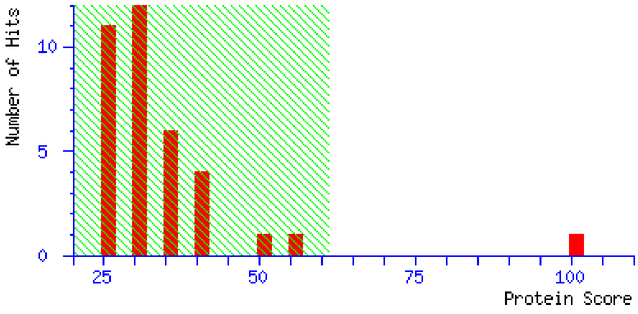

Spot No.: **23**

NCBI accession No.: **cassava4.1_033108m|PACid:17973616**

Plant species: ***Manihot esculenta***

Protein name： **Actin-7**

Mascot score: **93**

Sequence coverage %: **52** p value: **3.4e-005**

Matched peptides No.: **18** Total peptides No.: **87**

Calculated Mr: **41897** Calculated *p*I: **5.31**

Annotated PMF **spectra:**

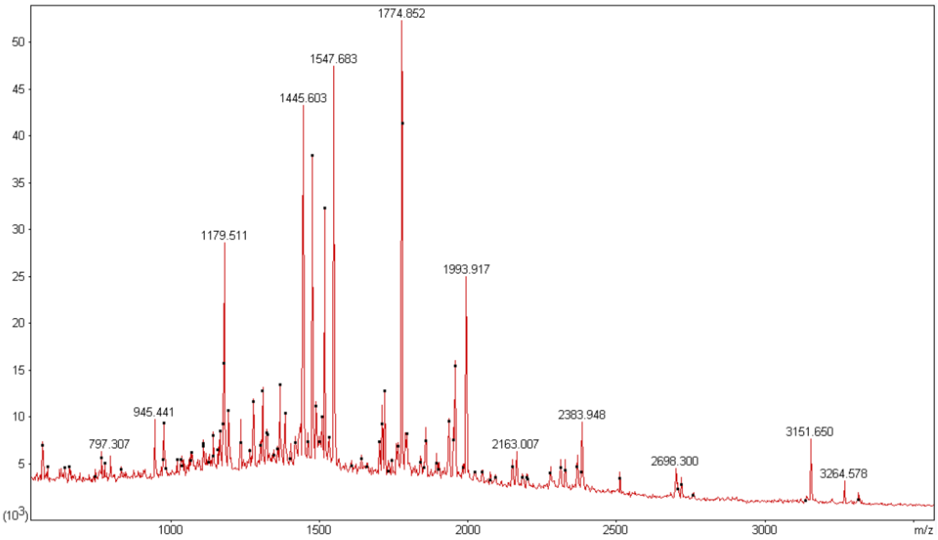

Probability Based Mowse Score:

Protein score is -10*Log(P), where P is the probability that the observed match is a random event.
Protein scores greater than 61 are significant (p<0.05).

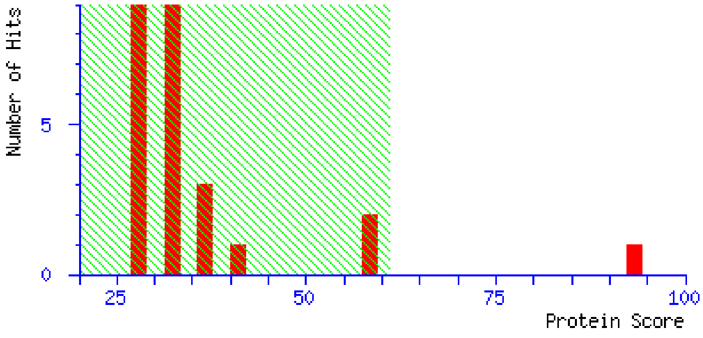

Spot No.: **27**

NCBI accession No.: **cassava4.1_011177m|PACid:17972447**

Protein name： **Adenosine kinase 1** Plant species: ***Manihot esculenta***

PFF Mascot score: [113] **Sequence coverage %:** [7]

Matched peptides No.: [**2**] p value: 3.1e-006

Calculated Mr: **38107** Calculated *p*I: **5.38**

Annotated PMF **spectra:**

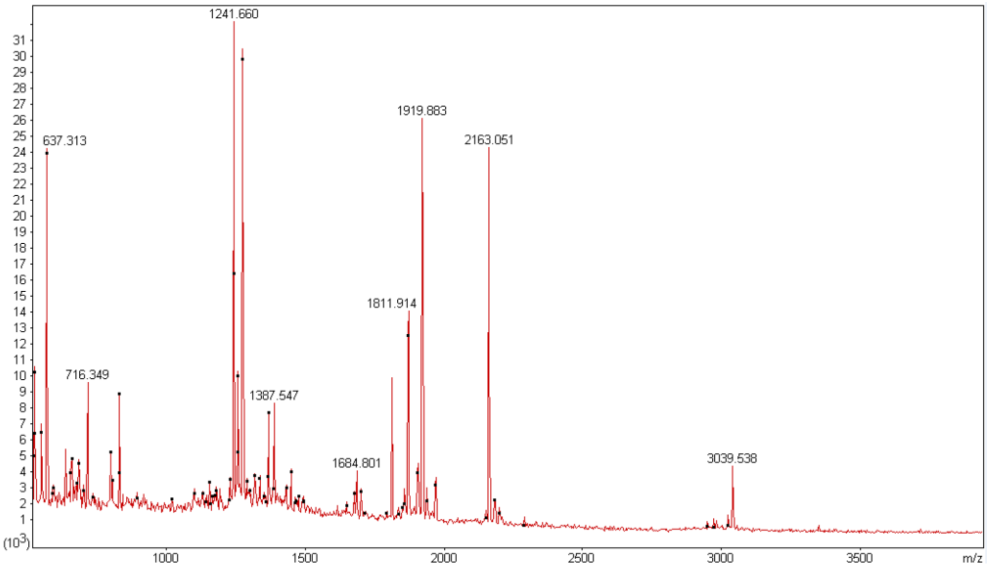

PFF Searched Score:

Ions score is -10*Log(P), where P is the probability that the observed match is a random event.
Individual ions scores > 33 indicate identity or extensive homology (p<0.05).
Protein scores are derived from ions scores as a non-probabilistic basis for ranking protein hits.

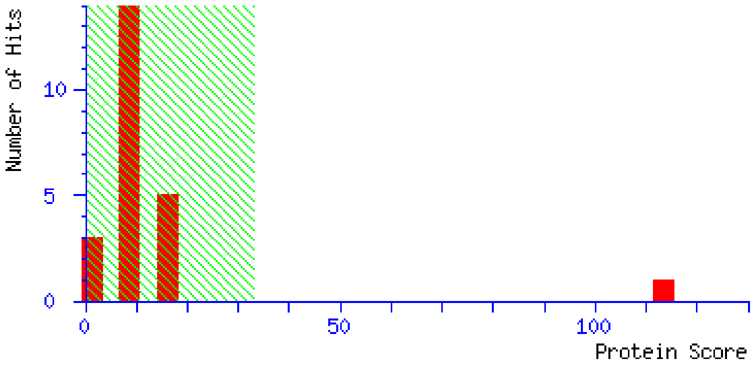

Spot No.: **28**

NCBI accession No.: **cassava4.1_002747m|PACid:17960141**

Plant species: ***Manihot esculenta***

Protein name： **Heat shock protein 90-3** Mascot score: **127**

Sequence coverage %: **37** p value: **1.5e-008**

Matched peptides No.: **33** Total peptides No.: **77**

Calculated Mr: **80250** Calculated *p*I: **4.99**

Annotated PMF **spectra:**

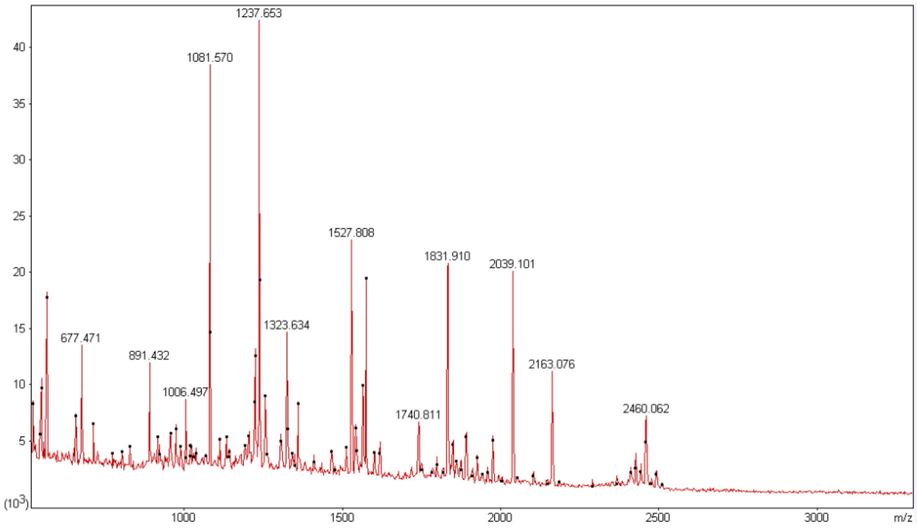

Probability Based Mowse Score:

Protein score is -10*Log(P), where P is the probability that the observed match is a random event.
Protein scores greater than 61 are significant (p<0.05).

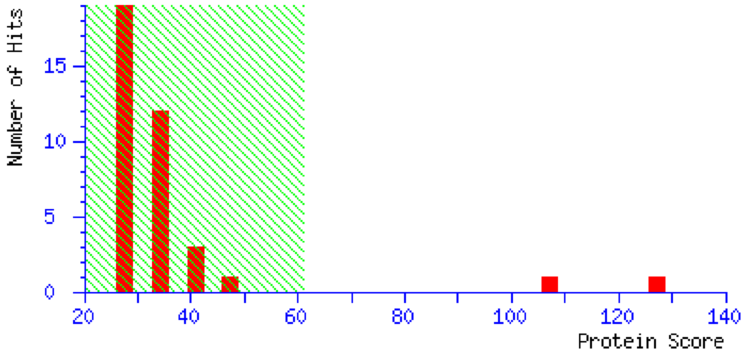

Spot No.: **29**

NCBI accession No.: **cassava4.1_002466m|PACid:17978799**

Plant species: ***Manihot esculenta***

Protein name: **Alpha-glucan phosphorylase 1**

Mascot score: **81**

Sequence coverage %: **24** p value: **0.00058**

Matched peptides No.: **24** Total peptides No.: **95**

Calculated Mr: **81918** Calculated *p*I: **8.50**

Annotated PMF **spectra:**

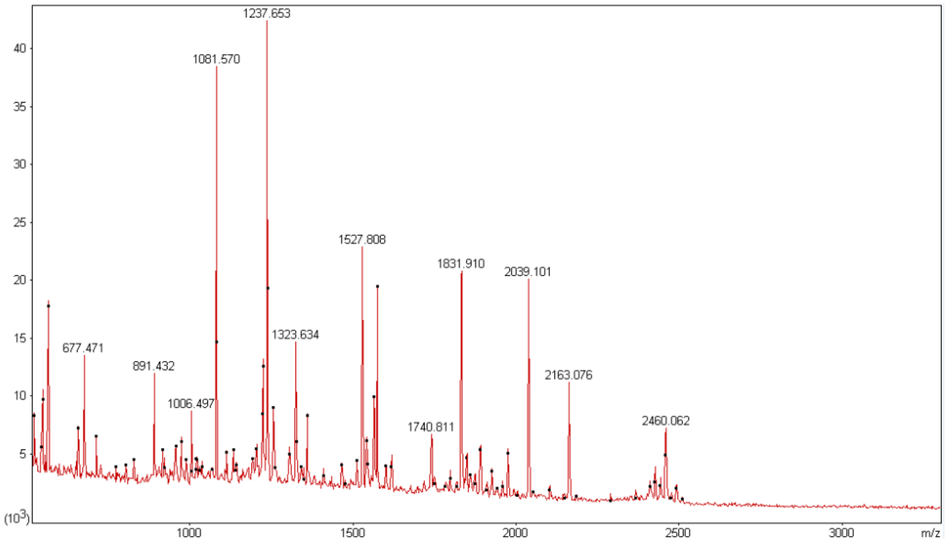

Probability Based Mowse Score:

Protein score is -10*Log(P), where P is the probability that the observed match is a random event.
Protein scores greater than 61 are significant (p<0.05).

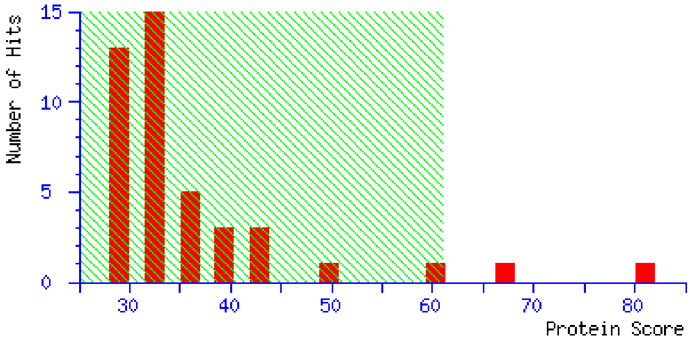

Spot No.: **30**

NCBI accession No.: **cassava4.1_002466m|PACid:17978799**

Plant species: **Manihot esculenta**

Protein name： **Alpha-glucan phosphorylase 1**

PFF **Mascot** score: **[387]** Sequence coverage %: **[12]**

Matched peptides No.: **[7]** p value: 3.5e-008

Calculated Mr: 81918 Calculated *p*I: **8.50**

Annotated PMF **spectra:**

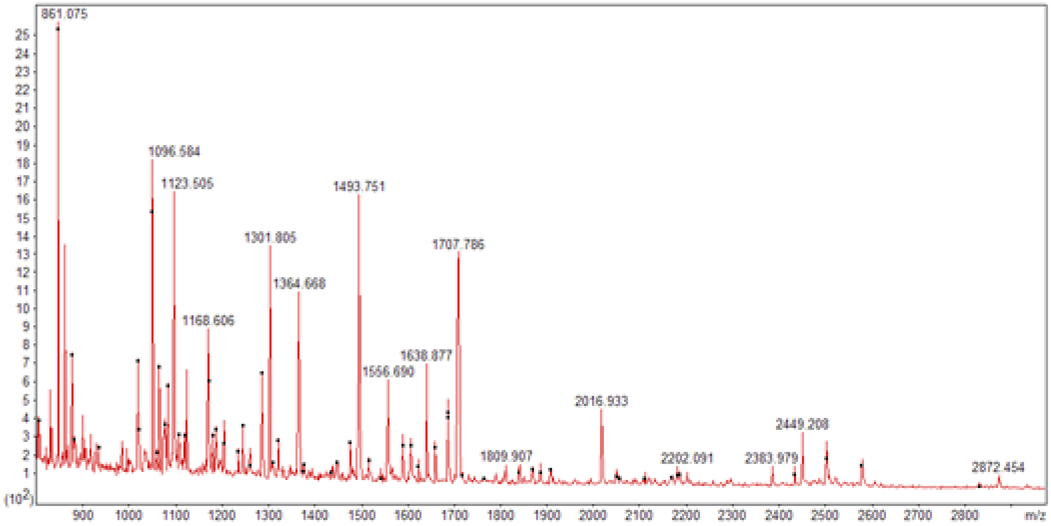

PFF Searched Score:

Ions score is -10*Log(P), where P is the probability that the observed match is a random event.
Individual ions scores > 33 indicate identity or extensive homology (p<0.05).
Protein scores are derived from ions scores as a non-probabilistic basis for ranking protein hits.

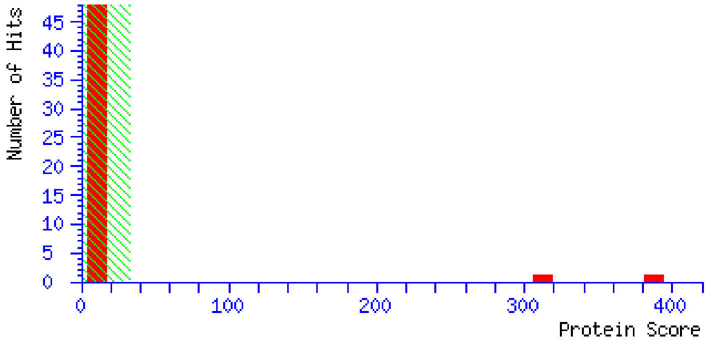

Spot No.: **31**

NCBI accession No.: **cassava4.1_002614m|PACid:17978880**

Plant species: ***Manihot esculenta***

Protein name： **Alpha-glucan phosphorylase 1**

Mascot score: **76** p value: **0.019**

Sequence coverage %: **32**

Matched peptides No.: **16** Total peptides No.: **58**

Calculated Mr: **80611** Calculated *p*I: **6.71**

Annotated PMF **spectra:**

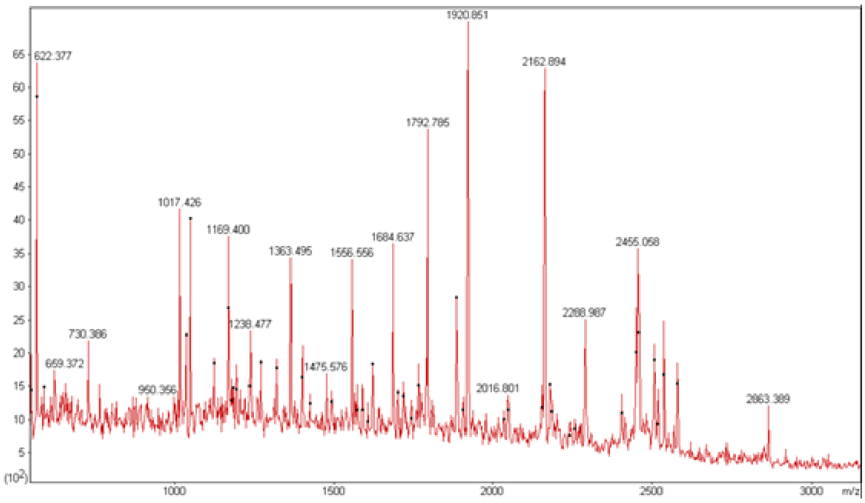

Probability Based Mowse Score:

Protein score is -10*Log(P), where P is the probability that the observed match is a random event.
Protein scores greater than 61 are significant (p<0.05).

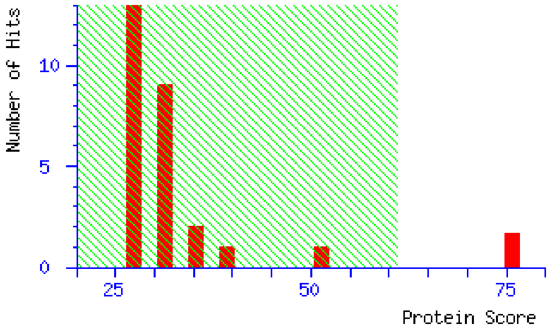

Spot No.: **34**

NCBI accession No.: **cassava4.1_014251m|PACid:17989805**

Plant species: ***Manihot esculenta***   Mascot score: **115**

Protein name: **14-3-3-like protein GF14 omega**

Sequence coverage %: **44**   p value: **2.3e-007**

Matched peptides No.: **16**   Total peptides No.: **46**

Calculated Mr: **29447**   Calculated *p*I: **4.77**

Annotated PMF **spectra:**

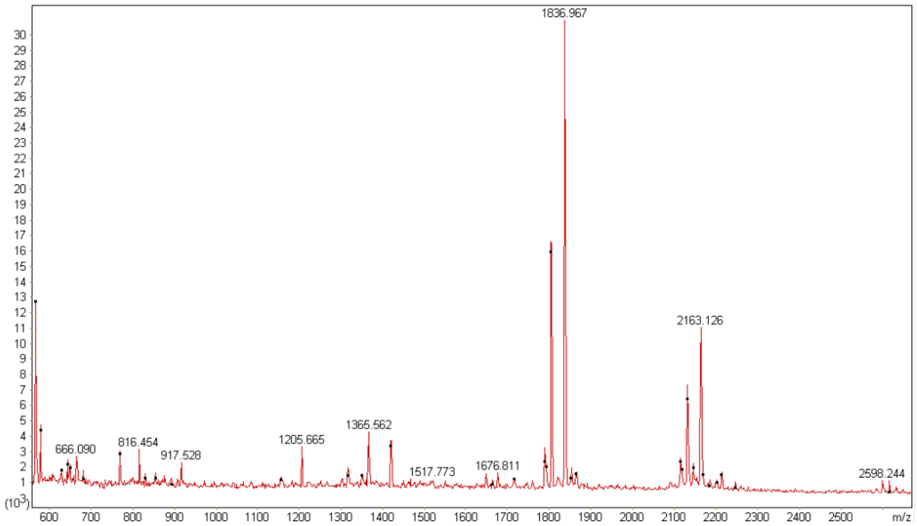

Probability Based Mowse Score:

Protein score is -10*Log(P), where P is the probability that the observed match is a random event.
Protein scores greater than 61 are significant (p<0.05).

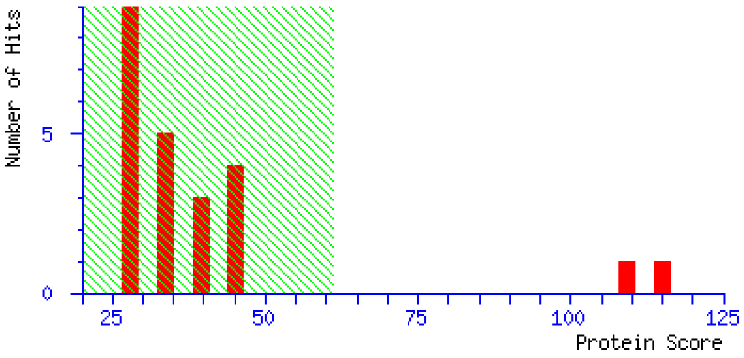

Spot No.: **35**

NCBI accession No.: **cassava4.1_014208m|PACid:17964823**

Plant species: *Manihot esculenta*

Protein name: **14-3-3-like protein GF14 omega**

Mascot score: **159** p value: **9.2e-012**

Sequence coverage %: **54**

Matched peptides No.: **21** Total peptides No.: **56**

Calculated Mr: **29548** Calculated *p*I: **4.76**

Annotated PMF **spectra:**

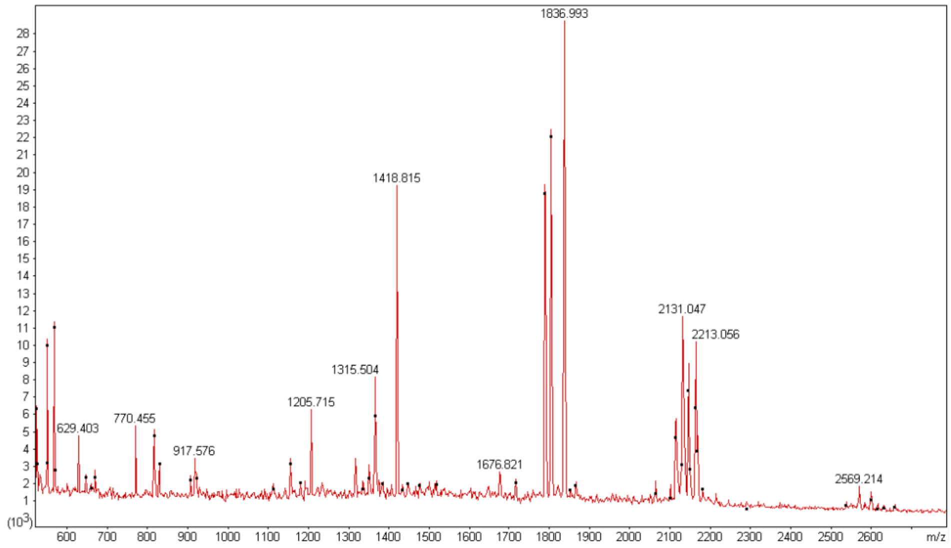

Probability Based Mowse Score:

Protein score is -10*Log(P), where P is the probability that the observed match is a random event.
Protein scores greater than 61 are significant (p<0.05).

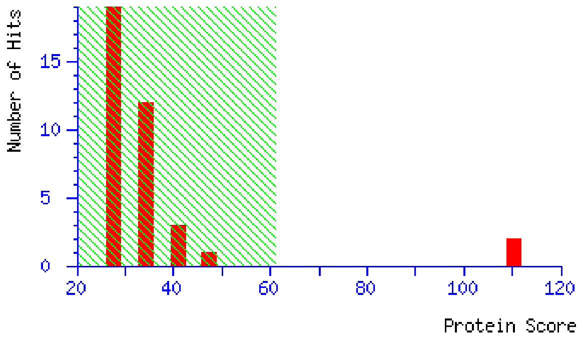

Spot No.: **36**

NCBI accession No.: **cassava4.1_014065m|PACid:17989625**

Plant species: *Manihot esculenta*

Protein name：**14-3-3-like protein GF14 psi**

Mascot score: **111** p value: **0.00000058**

Sequence coverage %: **60**

Matched peptides No.: **16** Total peptides No.: **67**

Calculated Mr: **29928** Calculated *p*I: **4.75**

Annotated PMF **spectra:**

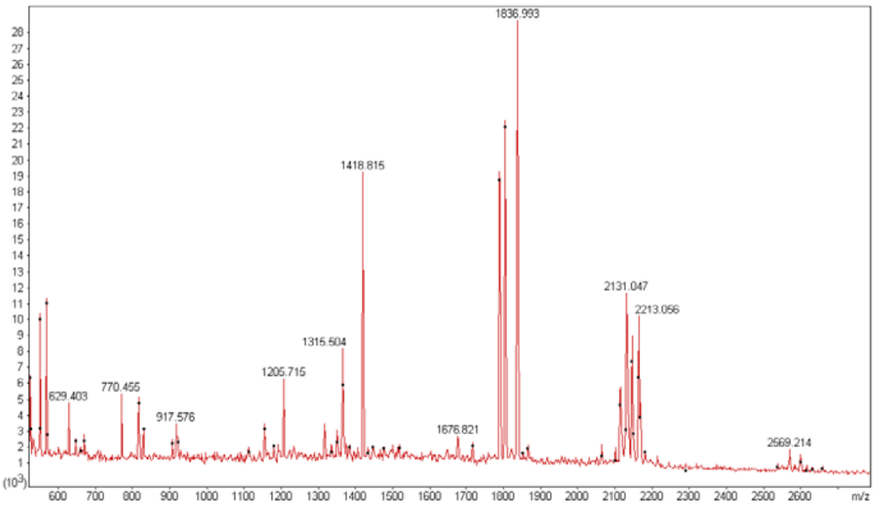

Probability Based Mowse Score:

Protein score is -10*Log(P), where P is the probability that the observed match is a random event.
Protein scores greater than 61 are significant (p<0.05).

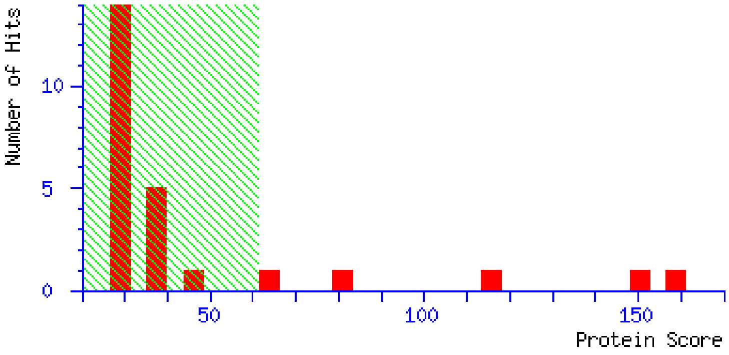

Spot No.: **39**

NCBI accession No.: **cassava4.1_013158m|PACid:17968853** p value: **0.0017**

Plant species: ***Manihot esculenta*** Mascot score: **151**

Protein name: **Probable lactoylglutathione lyase, chloroplast**

Sequence coverage %: **55** p value: **5.8e-011**

Matched peptides No.: **19** Total peptides No.: **77**

Calculated Mr: **32759** Calculated *p*I: **5.17**

Annotated PMF **spectra:**

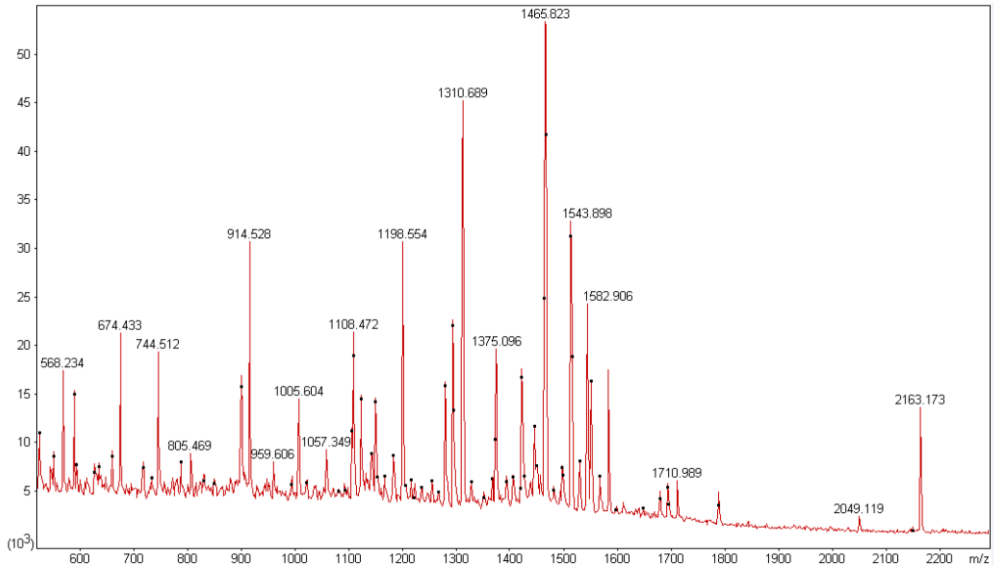

Probability Based Mowse Score:

Protein score is -10*Log(P), where P is the probability that the observed match is a random event.
Protein scores greater than 61 are significant (p<0.05).

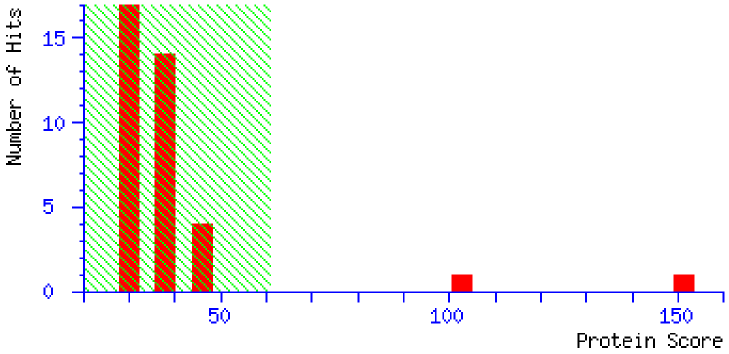

Spot No.: **40**

NCBI accession No.: **cassava4.1_013158m|PACid:17968853**

Plant species: ***Manihot esculenta***

Protein name：**Probable lactoylglutathione lyase, chloroplast**

PFF Mascot score: **[73]** Sequence coverage %: **[7]**

Matched peptides No.: **[2]** p value: 6.1e-006

Calculated Mr: **32759** Calculated *p*I: **5.17**

Annotated PMF **spectra:**

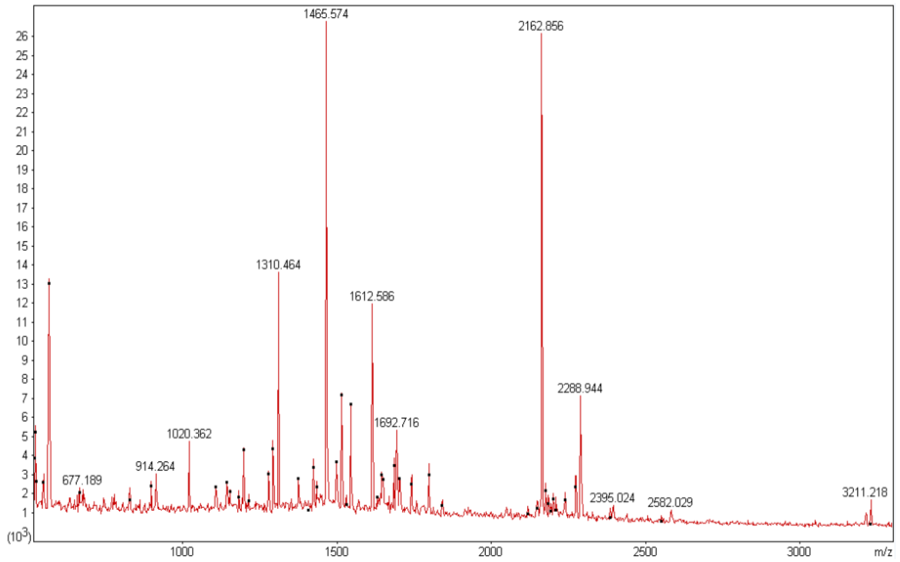

PFF Searched Score:

Ions score is -10*Log(P), where P is the probability that the observed match is a random event.
Individual ions scores > 33 indicate identity or extensive homology (p<0.05).
Protein scores are derived from ions scores as a non-probabilistic basis for ranking protein hits.

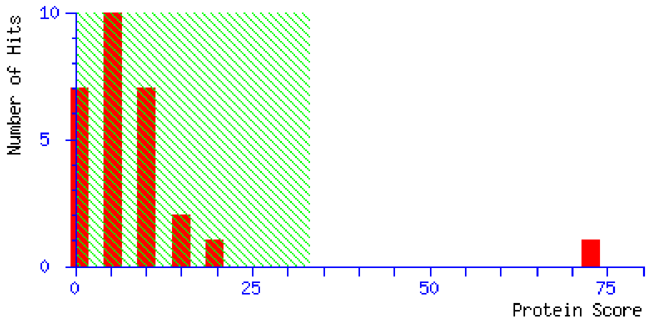

Spot No.: **42**

NCBI accession No.: **cassava4.1_011578m|PACid:17975802**

Plant species: ***Manihot esculenta***

Protein name： **Probable fructokinase-1**

PFF Mascot score: **[146]**　　　Sequence coverage %: **[11]**

Matched peptides No.: **[3]**　　　p value: **0.00019**

Calculated Mr: **35491**　　　Calculated *p*I: **5.29**

Annotated PMF **spectra:**

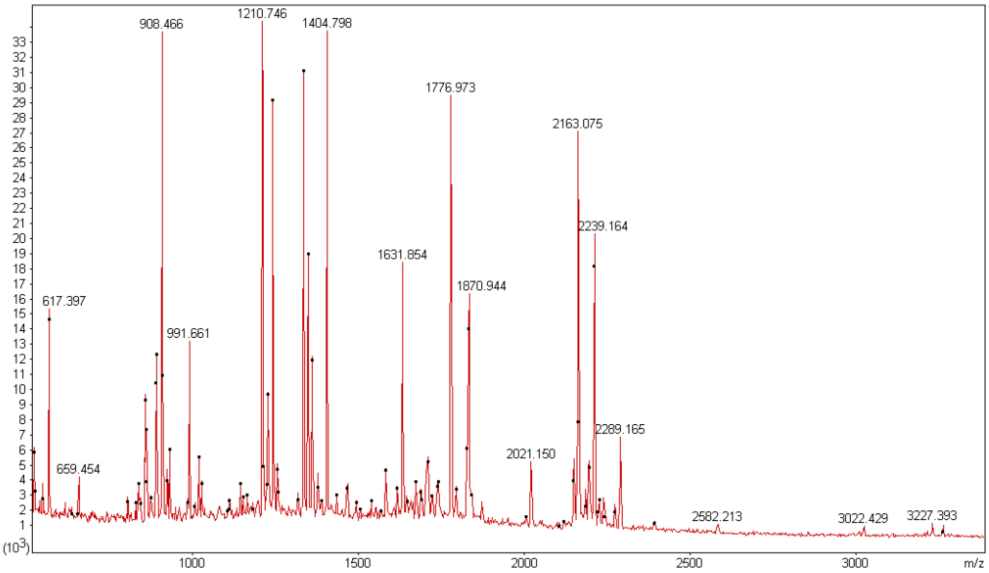

PFF Searched Score:

Ions score is -10*Log(P), where P is the probability that the observed match is a random event.
Individual ions scores > 33 indicate identity or extensive homology (p<0.05).
Protein scores are derived from ions scores as a non-probabilistic basis for ranking protein hits.

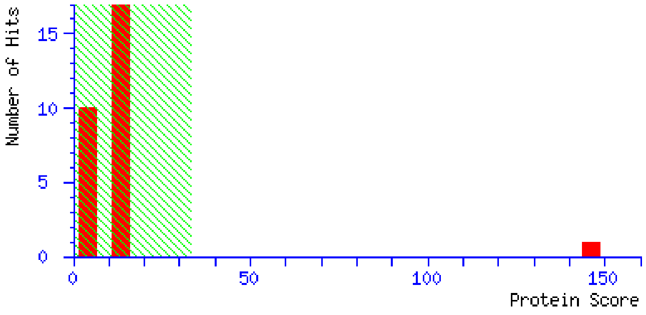

Spot No.: **44**

NCBI accession No.: **cassava4.1_010863m|PACid:17977552**

Plant species: ***Manihot esculenta***

Protein name：**NADP-dependent alkenal double bond reductase**

PFF Mascot score: **[99]**   Sequence coverage %: **[7]**

Matched peptides No.: **[2]**   p value: **7.3e-008**

Calculated Mr: **38818**   Calculated *p*I: **5.41**

Annotated PMF **spectra:**

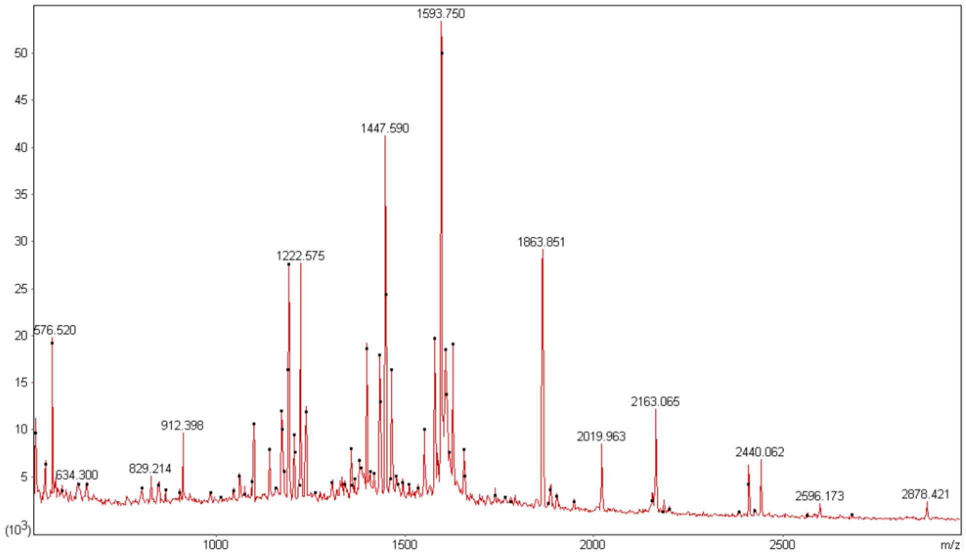

PFF Searched Score:

Ions score is -10*Log(P), where P is the probability that the observed match is a random event.
Individual ions scores > 33 indicate identity or extensive homology (p<0.05).
Protein scores are derived from ions scores as a non-probabilistic basis for ranking protein hits.

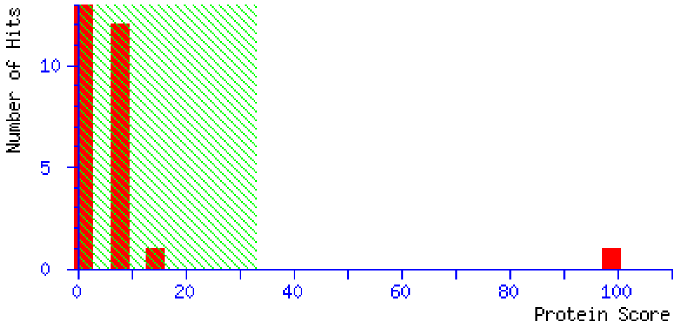

Spot No.: **46**

NCBI accession No.: **cassava4.1_012448m|PACid:17968446** Plant species: ***Manihot esculenta*** Protein name：**Putative NAD(P)H oxidoreductase, isoflavone reductase**

PFF Mascot score: **[254]** Sequence coverage %: **[15]**

Matched peptides No.: **[3]** p value: **4.7e-012**

Calculated Mr: 33873 Calculated *p*I: **5.47**

Annotated PMF **spectra:**

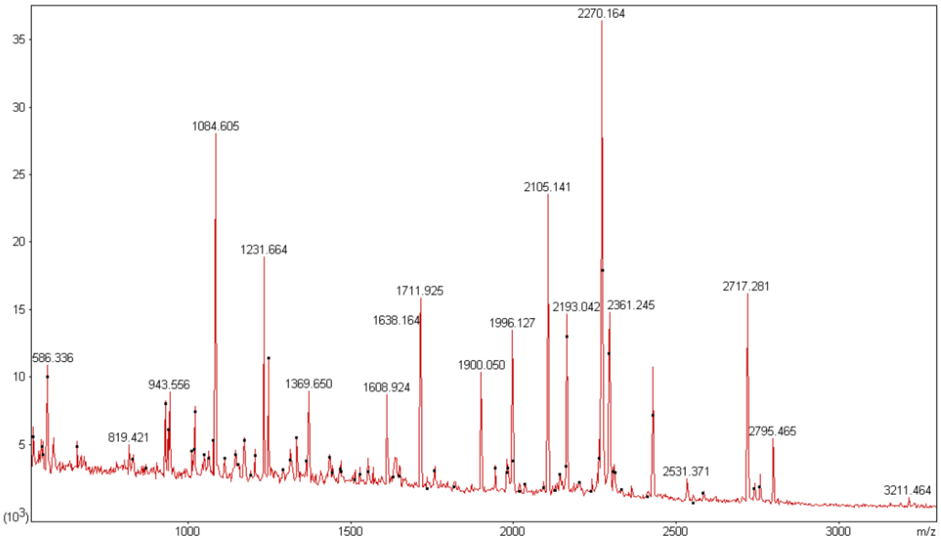

PFF Searched Score:

Ions score is -10*Log(P), where P is the probability that the observed match is a random event.
Individual ions scores > 32 indicate identity or extensive homology (p<0.05).
Protein scores are derived from ions scores as a non-probabilistic basis for ranking protein hits.

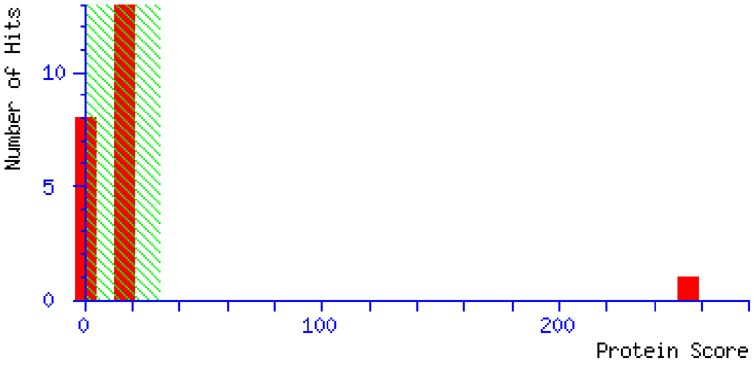

Spot No.: **47**

NCBI accession No.: **cassava4.1_010620m|PACid:17987783**

Plant species: ***Manihot esculenta***

Protein name: **Thiamine thiazole synthase, chloroplastic**

PFF Mascot score: [133] **Sequence coverage %: [8]**

Matched peptides No.: **[2]** p value: **6.7e-006**

Calculated Mr: **37625** Calculated *p*I: **6.07**

Annotated PMF **spectra:**

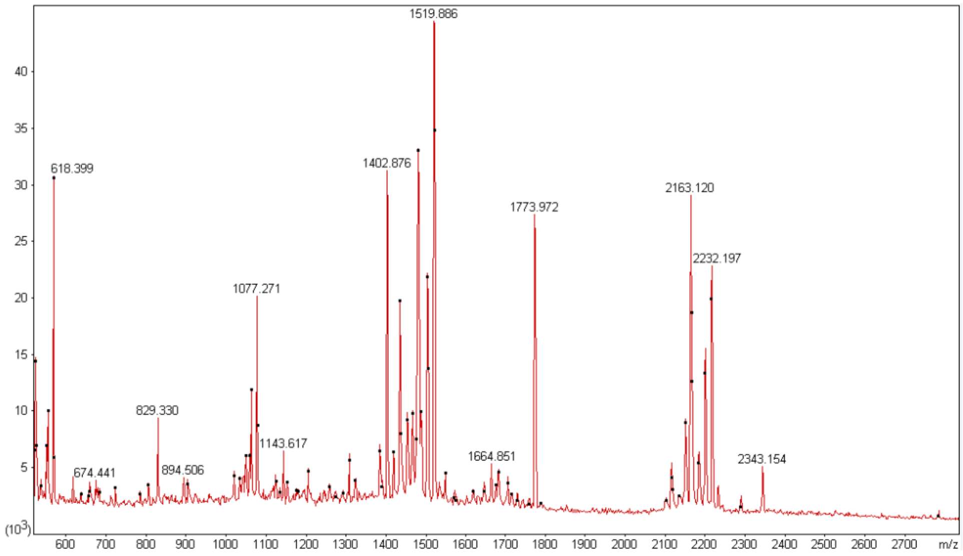

PFF Searched Score:

Ions score is -10*Log(P), where P is the probability that the observed match is a random event.
Individual ions scores > 33 indicate identity or extensive homology (p<0.05).
Protein scores are derived from ions scores as a non-probabilistic basis for ranking protein hits.

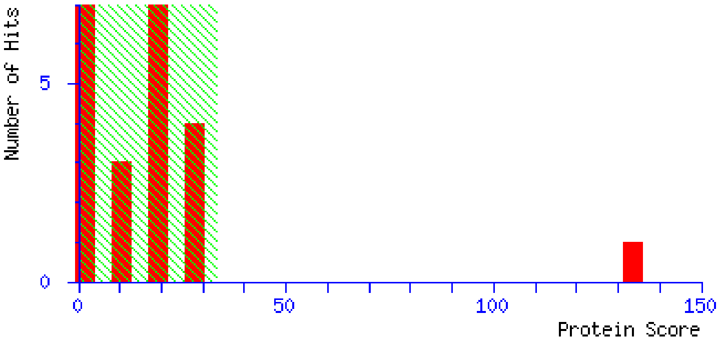

Spot No.: **49**

NCBI accession No.: **cassava4.1_014185m|PACid:17964106**

Plant species: ***Manihot esculenta***

Protein name: **Ferritin-4, chloroplastic**

Mascot score: **125**

Sequence coverage %: **61** p value: **2.3e-008**

Matched peptides No.: **18** Total peptides No.: **68**

Calculated Mr: **29470** Calculated *p*I: **5.30**

Annotated PMF **spectra:**

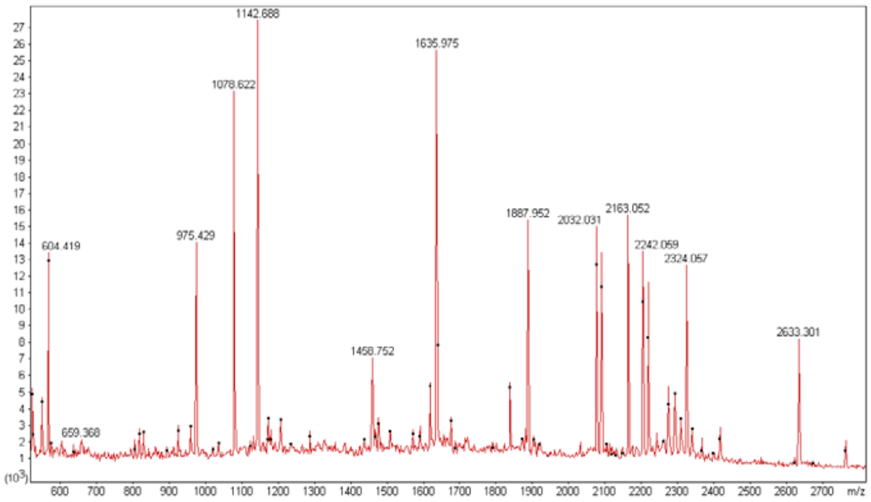

Probability Based Mowse Score:

Protein score is -10*Log(P), where P is the probability that the observed match is a random event. Protein scores greater than 61 are significant (p<0.05).

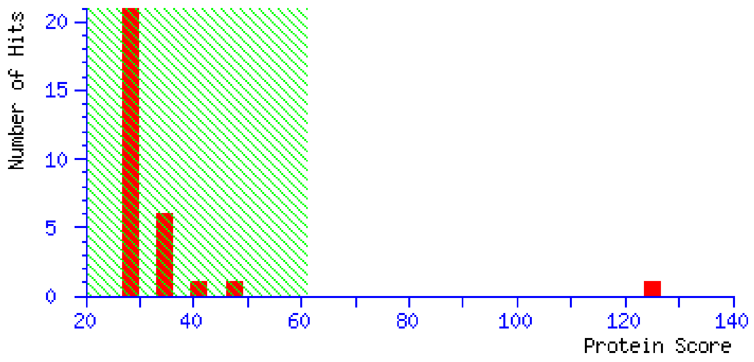

Spot No.: **50**

NCBI accession No.: **cassava4.1_014410m|PACid:17965277**

Plant species: ***Manihot esculenta***

Protein name: **20 kDa chaperonin, chloroplastic**

PFF Mascot score: **[172]** Sequence coverage %: **[21]**

Matched peptides No.: **[3]** p value: **5e-005**

Calculated Mr: **26695** Calculated *p*I: **7.79**

Annotated PMF **spectra:**

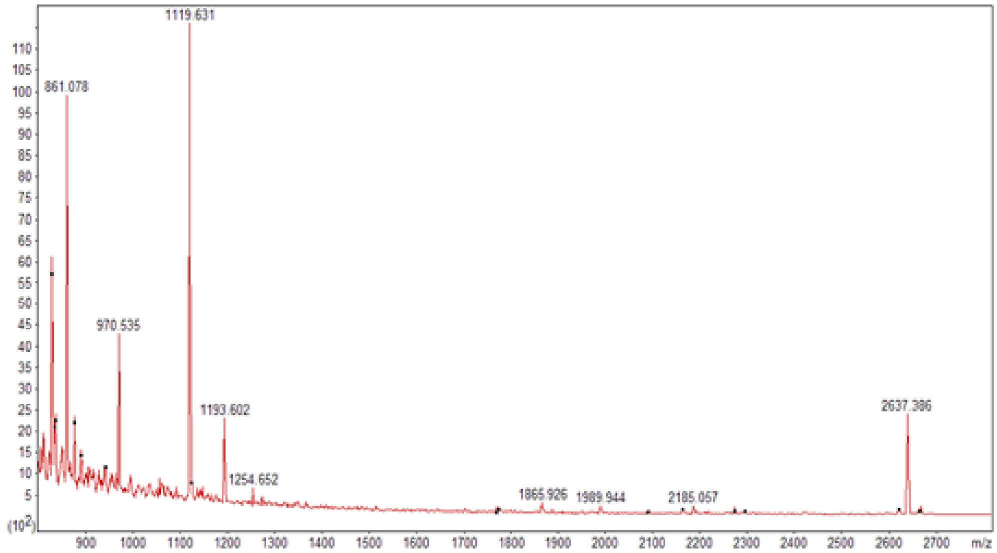

PFF Searched Score:

Ions score is -10*Log(P), where P is the probability that the observed match is a random event.
Individual ions scores > 33 indicate identity or extensive homology (p<0.05).
Protein scores are derived from ions scores as a non-probabilistic basis for ranking protein hits.

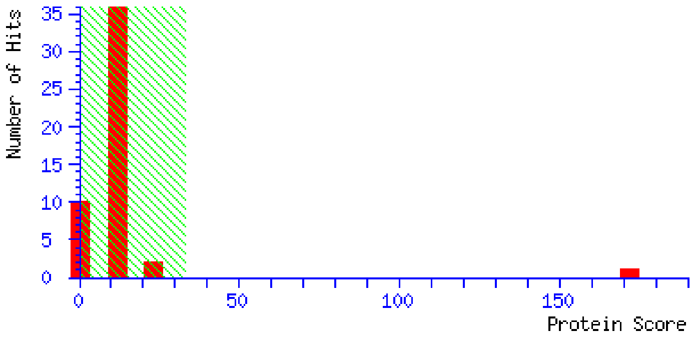

Spot No.: **51**

NCBI accession No.: **cassava4.1_014643m|PACid:17991662**

Plant species: ***Manihot esculenta***

Protein name: **L-ascorbate peroxidase 1, cytosolic**

Mascot score: **82** p value: **0.00046**

Sequence coverage %: **40** Matched peptides No.: **13**

Calculated Mr: **27766** Calculated *p*I: **5.31**

Annotated PMF **spectra:**

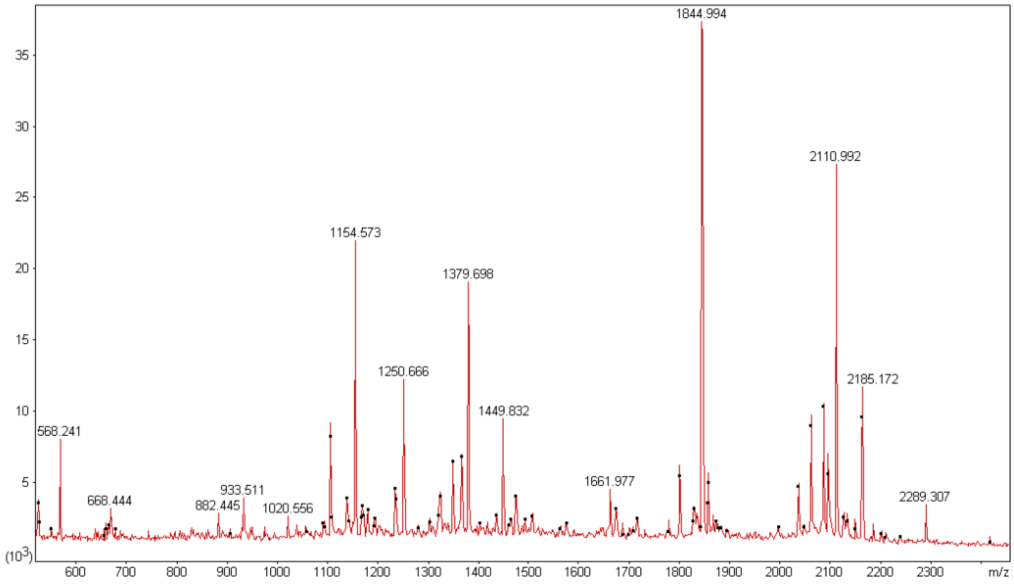

Probability Based Mowse Score:

Protein score is -10*Log(P), where P is the probability that the observed match is a random event. Protein scores greater than 61 are significant (p<0.05).

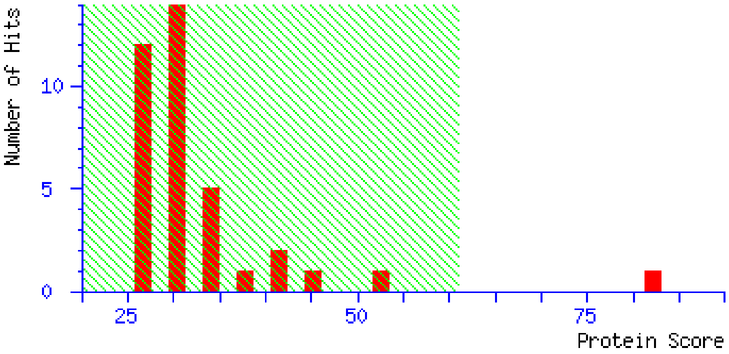

Spot No.: **53**

NCBI accession No.: **cassava4.1_009779m|PACid:17990964**

Plant species: ***Manihot esculenta***

Protein name： **Actin-7**

PFF Mascot score: [96] **Sequence coverage %:** [7]

Matched peptides No.: [**2]** p value: **0.00065**

Calculated Mr: 41897 Calculated p*I*: **5.31**

Annotated PMF **spectra:**

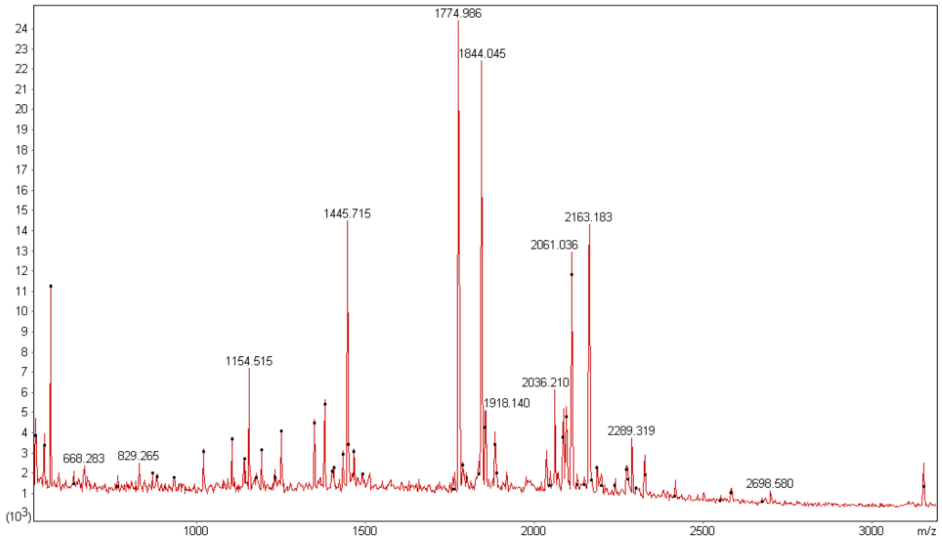

PFF Searched Score:

Ions score is -10*Log(P), where P is the probability that the observed match is a random event.
Individual ions scores > 33 indicate identity or extensive homology (p<0.05).
Protein scores are derived from ions scores as a non-probabilistic basis for ranking protein hits.

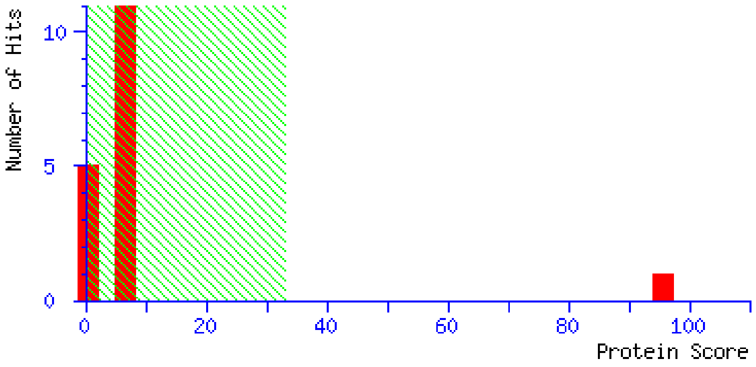

Spot No.: **57**

NCBI accession No.: **cassava4.1_017832m|PACid:17961591**

Plant species: ***Manihot esculenta***

Protein name： **regulator of ribonuclease activity a**

PFF Mascot score: [133] **Sequence coverage %: [16]**

Matched peptides No.: [**2**] p value: **8.1e-007**

Calculated Mr: 18015 Calculated *p*I: **5.39**

Annotated PMF **spectra:**

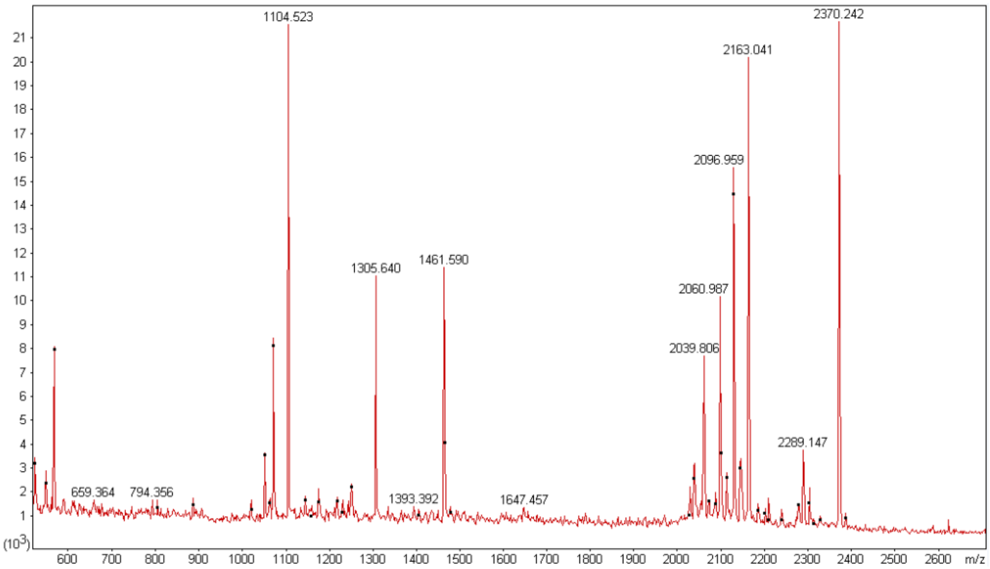

PFF Searched Score:

Ions score is -10*Log(P), where P is the probability that the observed match is a random event.
Individual ions scores > 33 indicate identity or extensive homology (p<0.05).
Protein scores are derived from ions scores as a non-probabilistic basis for ranking protein hits.

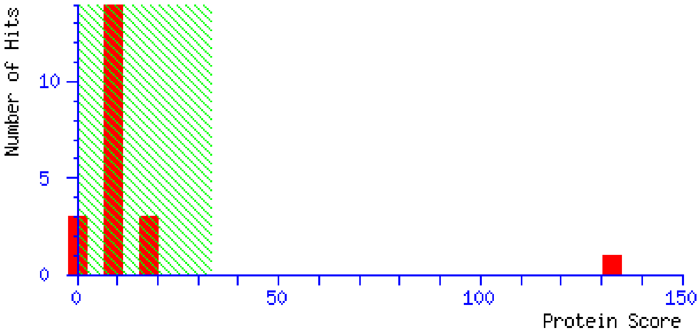

Spot No.: **58**

NCBI accession No.: **cassava4.1_018059m|PACid:17963195**

Plant species: ***Manihot esculenta***

Protein name: **Eukaryotic translation initiation factor 5A-2**

PFF Mascot score: [66] **Sequence coverage %: [18]**

Matched peptides No.: **[2]** p value: **0.00026**

Calculated Mr: **17706** Calculated *p*I: **5.60**

Annotated PMF **spectra:**

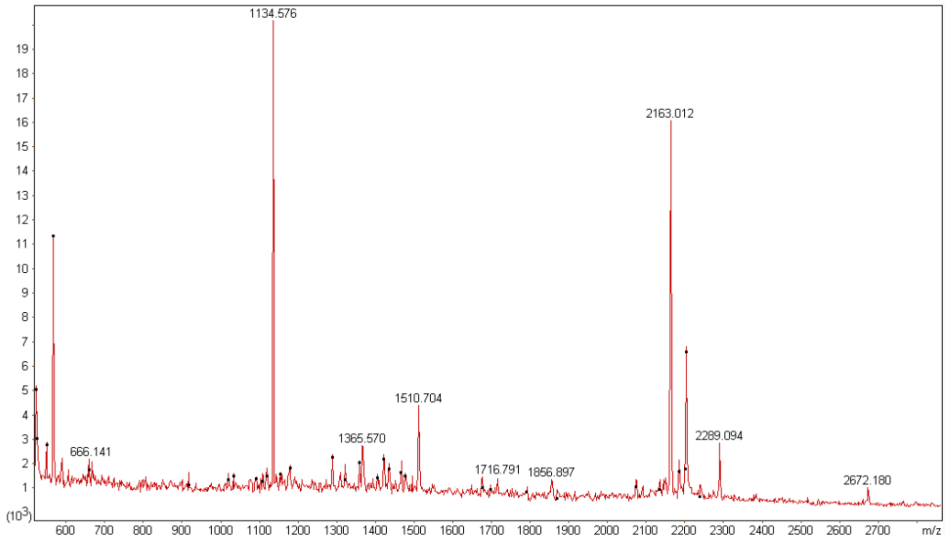

PFF Searched Score:

Ions score is -10*Log(P), where P is the probability that the observed match is a random event.
Individual ions scores > 33 indicate identity or extensive homology (p<0.05).
Protein scores are derived from ions scores as a non-probabilistic basis for ranking protein hits.

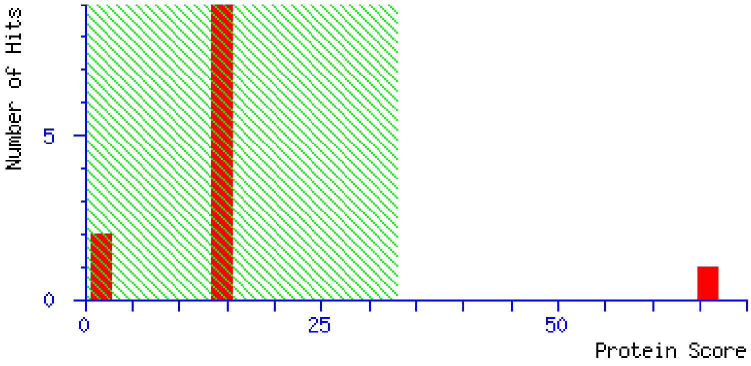

Spot No.: **61**

NCBI accession No.: **cassava4.1_018737m|PACid:17986123**

Plant species: ***Manihot esculenta***

Protein name: **Actin-depolymerizing factor 3**

PFF Mascot score: [112] Sequence coverage %: [11]

Matched peptides No.: **[2]** p value: **5.3e-010**

Calculated Mr: **16253** Calculated *p*I: **6.60**

Annotated PMF **spectra:**

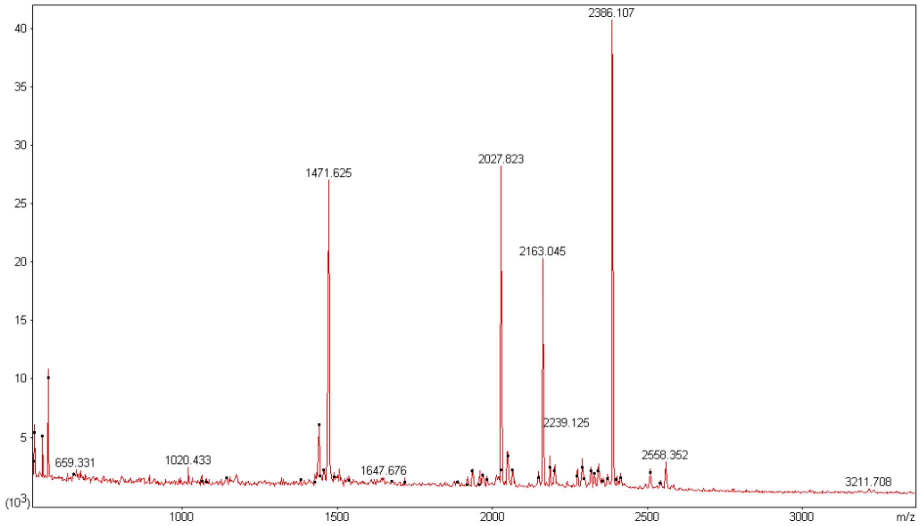

PFF Searched Score:

Ions score is -10*Log(P), where P is the probability that the observed match is a random event.
Individual ions scores > 32 indicate identity or extensive homology (p<0.05).
Protein scores are derived from ions scores as a non-probabilistic basis for ranking protein hits.

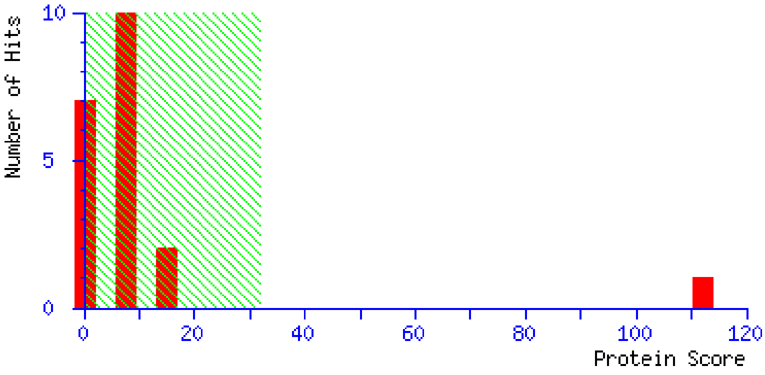

Spot No.: **64**

NCBI accession No.: **cassava4.1_017973m|PACid:17985926**

Plant species: ***Manihot esculenta***

Protein name：**Peroxiredoxin-2B**

PFF Mascot score: [**180]**　　　Sequence coverage %: **[22]**

Matched peptides No.: [**10]**　　　p value: 2e-013

Calculated Mr: **17433**　　　Calculated *p*I: **5.70**

Annotated PMF **spectra:**

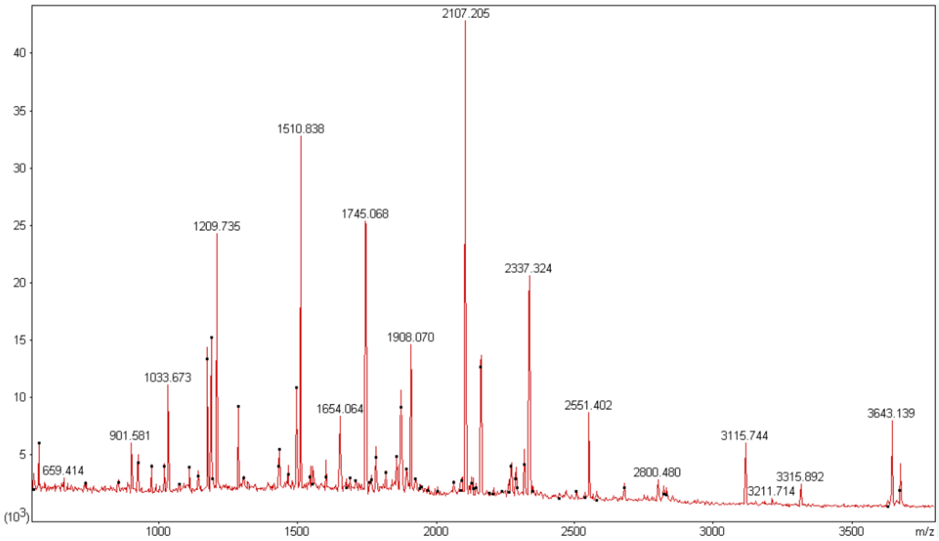

PFF Searched Score:

Ions score is -10*Log(P), where P is the probability that the observed match is a random event.
Individual ions scores > 32 indicate identity or extensive homology (p<0.05).
Protein scores are derived from ions scores as a non-probabilistic basis for ranking protein hits.

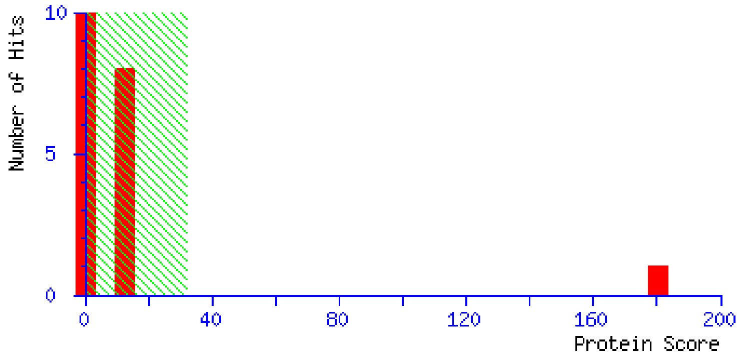

Spot No.: **65**

NCBI accession No.: **cassava4.1_017871m|PACid:17980723**

Plant species: ***Manihot esculenta***

Protein name: **18.1 kDa class I heat shock protein**

PFF Mascot score: **[164]** Sequence coverage %: **[25]**

Matched peptides No.: **[5]** p value: **0.00017**

Calculated Mr: **18750** Calculated *p*I: **7.93**

Annotated PMF **spectra:**

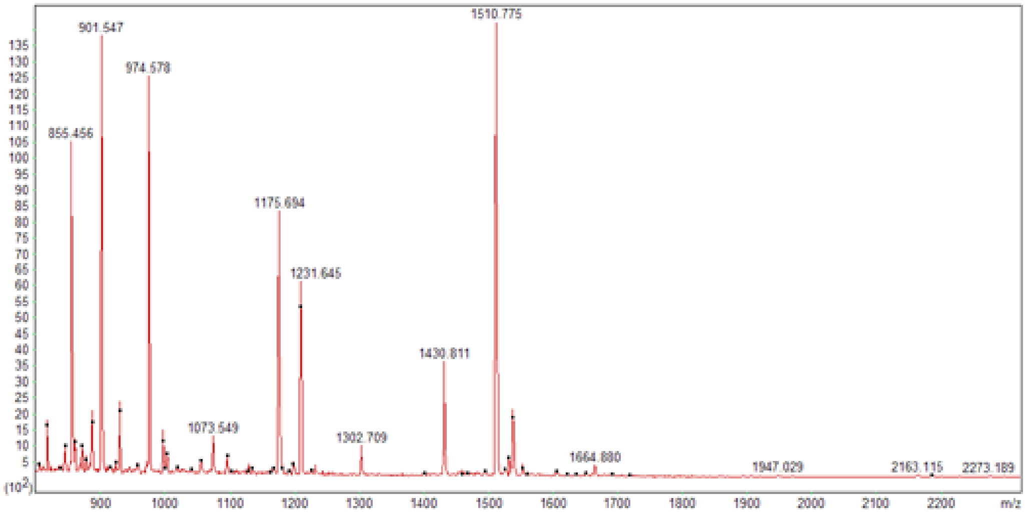

PFF Searched Score:

Ions score is -10*Log(P), where P is the probability that the observed match is a random event.
Individual ions scores > 34 indicate identity or extensive homology (p<0.05).
Protein scores are derived from ions scores as a non-probabilistic basis for ranking protein hits.

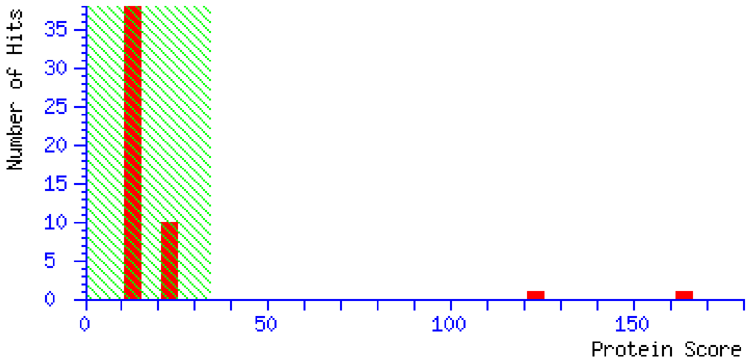

Spot No.: **68**

NCBI accession No.: **cassava4.1_018338m|PACid:17992346**

Plant species: ***Manihot esculenta***

Protein name： **Ubiquitin-conjugating enzyme E2 variant 1C**

Mascot score: **119**

Sequence coverage %: **72**　　p value: **9.2e-008**

Matched peptides No.: **12**　　Total peptides No.: **37**

Calculated Mr: **17236**　　Calculated *p*I: **5.73**

Annotated PMF **spectra:**

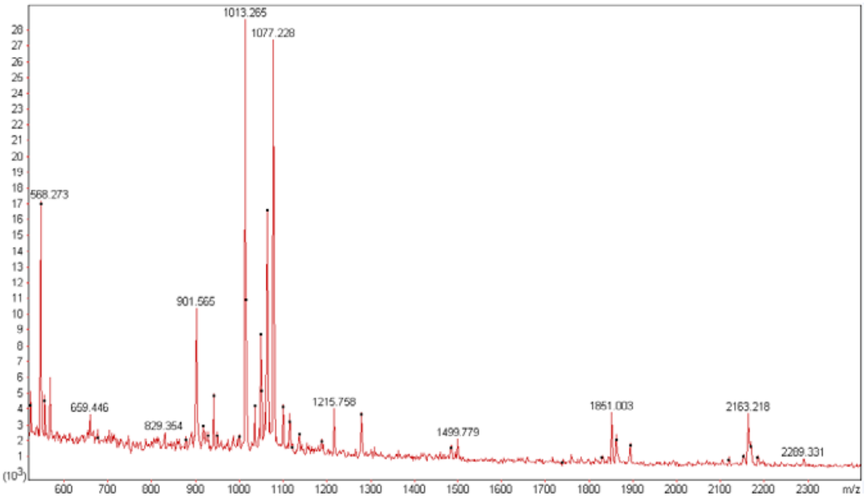

Probability Based Mowse Score:

Protein score is -10*Log(P), where P is the probability that the observed match is a random event.
Protein scores greater than 61 are significant ($p<0.05$).

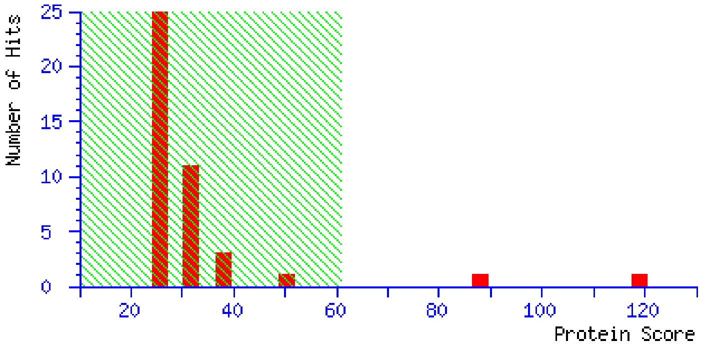

Spot No.: **69**

NCBI accession No.: **cassava4.1_018010m|PACid:17970081**

Plant species: ***Manihot esculenta***

Protein name： **AT3g53990/F5K20_290**

PFF Mascot score: [195] **Sequence coverage %: [21]**

Matched peptides No.: **[3]** p value: **5.2e-006**

Calculated Mr: **18044** Calculated *p*I: **6.14**

Annotated PMF **spectra:**

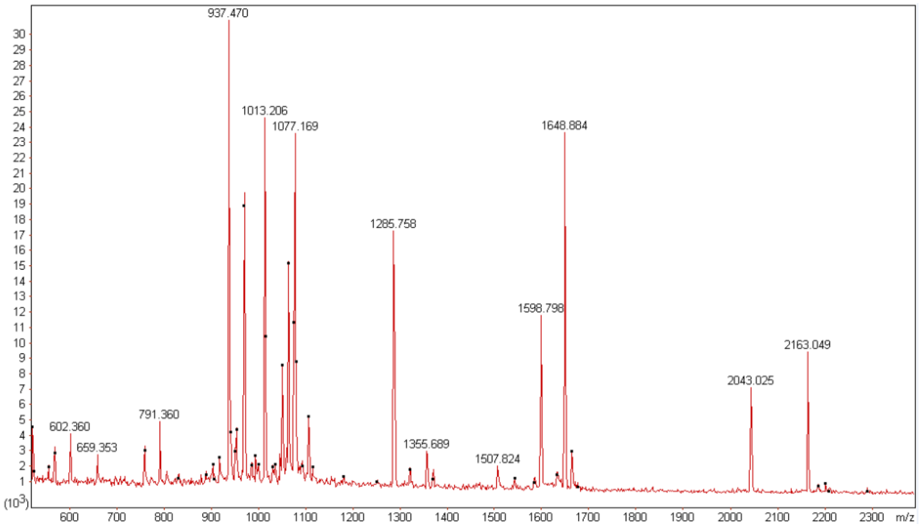

PFF Searched Score:

Ions score is -10*Log(P), where P is the probability that the observed match is a random event.
Individual ions scores > 33 indicate identity or extensive homology (p<0.05).
Protein scores are derived from ions scores as a non-probabilistic basis for ranking protein hits.

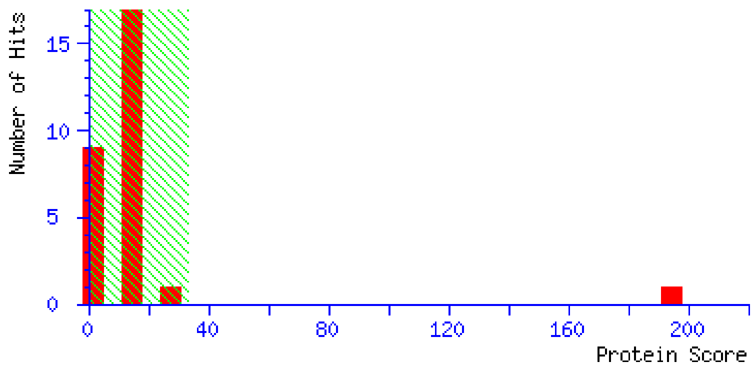

Spot No.: 71

NCBI accession No.: cassava4.1_013840m|PACid:17968262

Plant species: ***Manihot esculenta***

Protein name：**Proteasome subunit alpha type-2-A**

PFF Mascot score: [99]

Sequence coverage %: [8]

Matched peptides No.: **[2]**

p value: **0.0001**

Calculated Mr: **29845**

Calculated *p*I: **6.13**

Annotated PMF **spectra:**

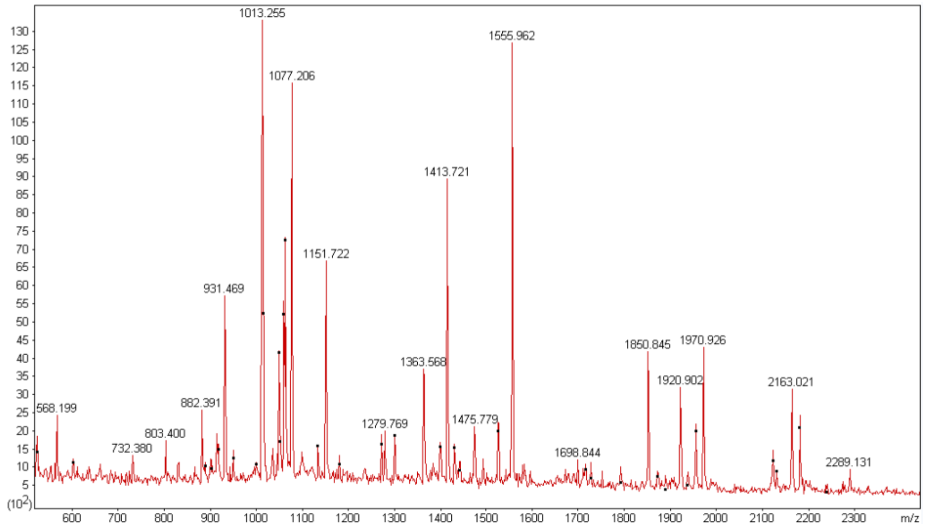

PFF Searched Score:

Ions score is -10*Log(P), where P is the probability that the observed match is a random event.
Individual ions scores > 33 indicate identity or extensive homology (p<0.05).
Protein scores are derived from ions scores as a non-probabilistic basis for ranking protein hits.

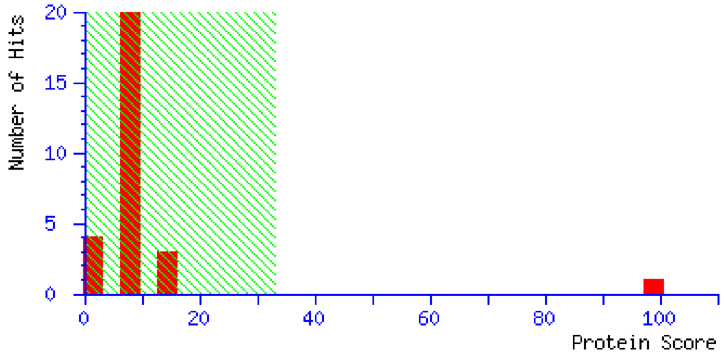

Spot No.: **77**

NCBI accession No.: **cassava4.1_010971m|PACid:17987784**

Plant species: ***Manihot esculenta***

Protein name: **Thiamine thiazole synthase, chloroplastic**

Mascot score: **88**

Sequence coverage %: **38** p value: **0.00011**

Matched peptides No.: **17** Total peptides No.: **70**

Calculated Mr: **36908** Calculated *p*I: **7.10**

Annotated PMF **spectra:**

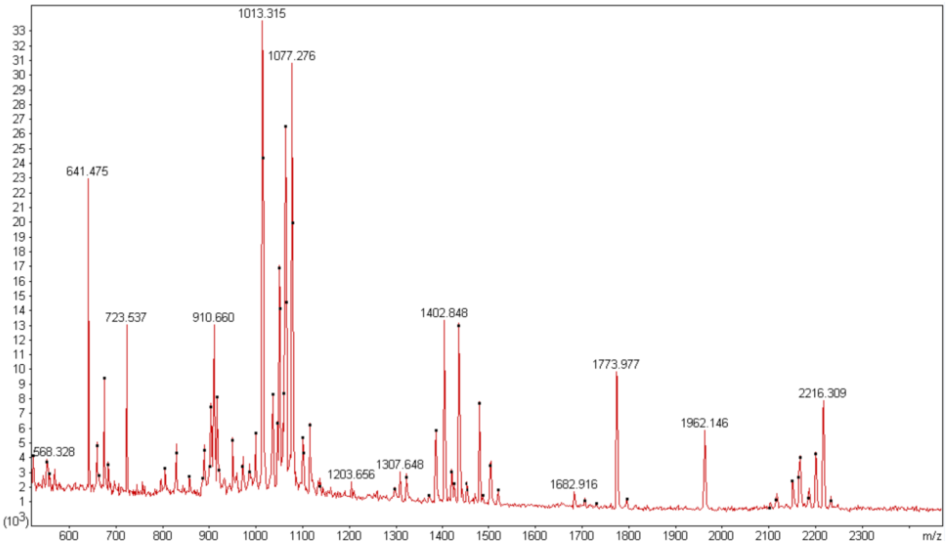

Probability Based Mowse Score:

Protein score is -10*Log(P), where P is the probability that the observed match is a random event.
Protein scores greater than 61 are significant (p<0.05).

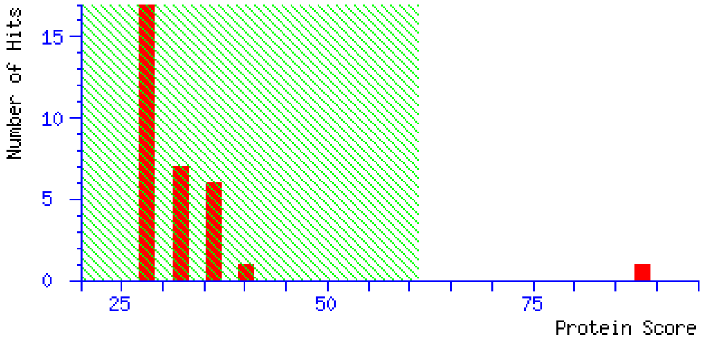

Spot No.: **78**

NCBI accession No.: **cassava4.1_009245m|PACid:17963775**

Plant species: ***Manihot esculenta***

Protein name: **S-adenosylmethionine synthase 1**

Mascot score: **119**

Sequence coverage %: **55** p value: **9.2e-008**

Matched peptides No.: **16** Total peptides No.: **54**

Calculated Mr: **43709** Calculated *p*I: **5.68**

Annotated PMF **spectra:**

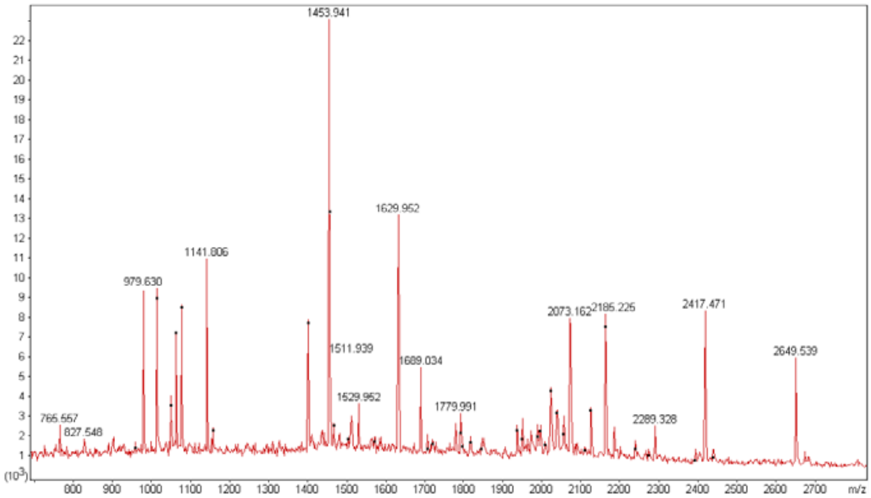

Probability Based Mowse Score:

Protein score is -10*Log(P), where P is the probability that the observed match is a random event. Protein scores greater than 61 are significant (p<0.05).

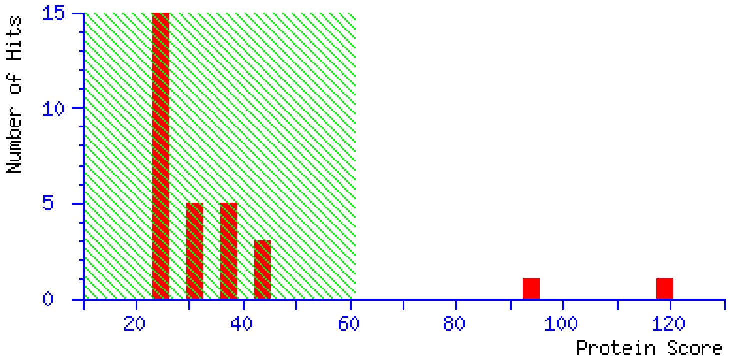

Spot No.: **79**

NCBI accession No.: **cassava4.1_011550m|PACid:17993466**

Plant species: ***Manihot esculenta***

Protein name：**predicted protein** Mascot score: **108**

Sequence coverage %: **50**　　p value: **1.2e-006**

Matched peptides No.: **17**　　Total peptides No.: **67**

Calculated Mr: **36178**　　Calculated *p*I: **6.09**

Annotated PMF **spectra:**

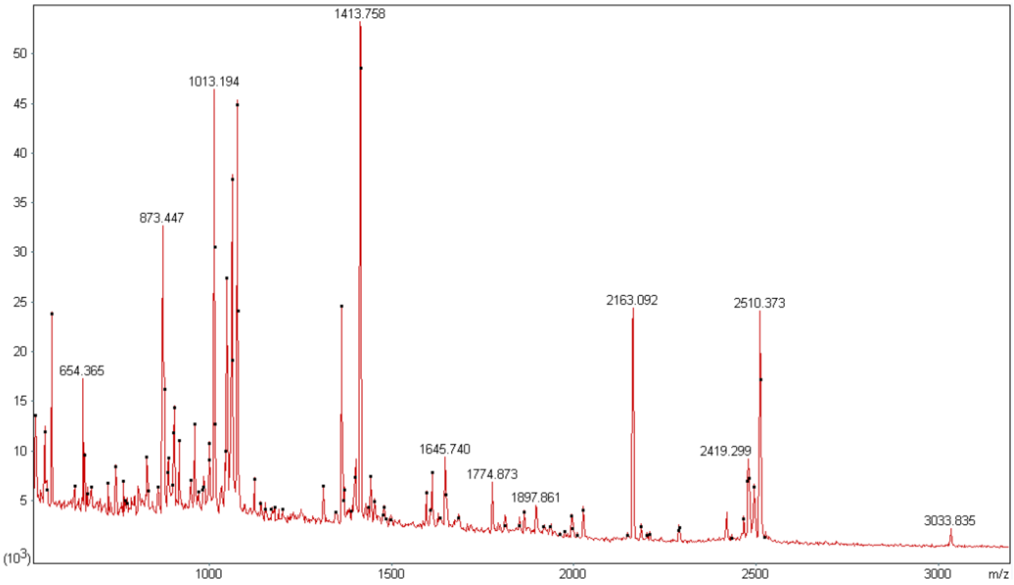

Probability Based Mowse Score:

Protein score is -10*Log(P), where P is the probability that the observed match is a random event. Protein scores greater than 61 are significant (p<0.05).

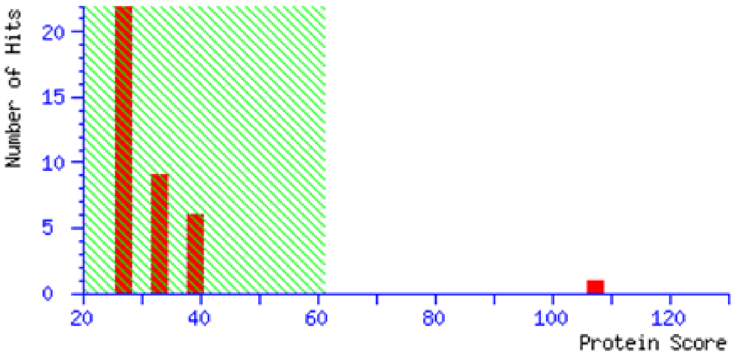

Spot No.: **86**

NCBI accession No.: **cassava4.1_004164m|PACid:17985770**

Plant species: ***Manihot esculenta***

Protein name: **malic enzyme, putative** Mascot score: **177**

Sequence coverage %: **47** p value: **1.5e-013**

Matched peptides No.: **33** Total peptides No.: **90**

Calculated Mr: **65246** Calculated *p*I: **6.03**

Annotated PMF **spectra:**

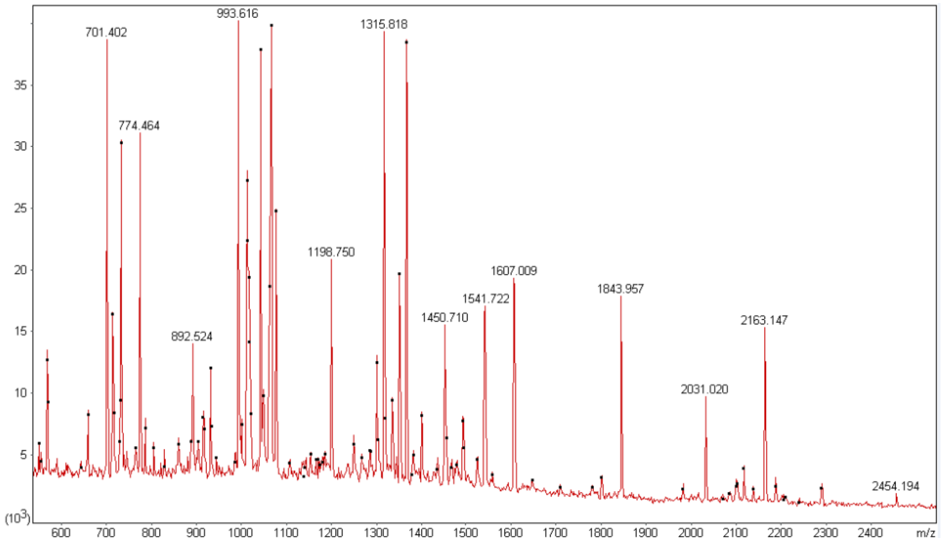

Probability Based Mowse Score:

Protein score is -10*Log(P), where P is the probability that the observed match is a random event. Protein scores greater than 61 are significant (p<0.05).

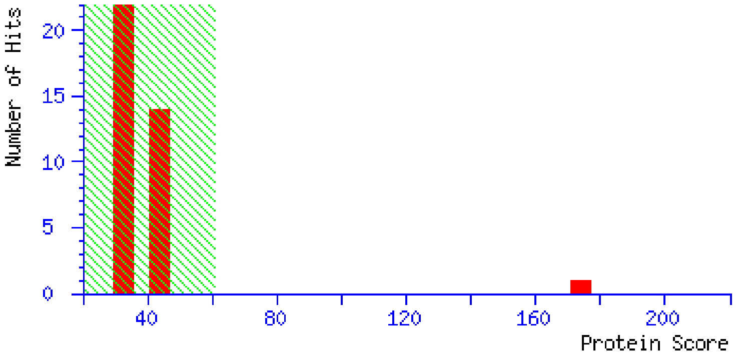

Spot No.: **88**

NCBI accession No.: **cassava4.1_007678m|PACid:17961094**

Plant species: ***Manihot esculenta*** Mascot score: **160**

Protein name： **Bifunctional enolase 2/transcriptional activator**

Sequence coverage %: **62** p value: **7.3e-012**

Matched peptides No.: **25** Total peptides No.: **78**

Calculated Mr: **47854** Calculated *p*I: **5.86**

Annotated PMF **spectra:**

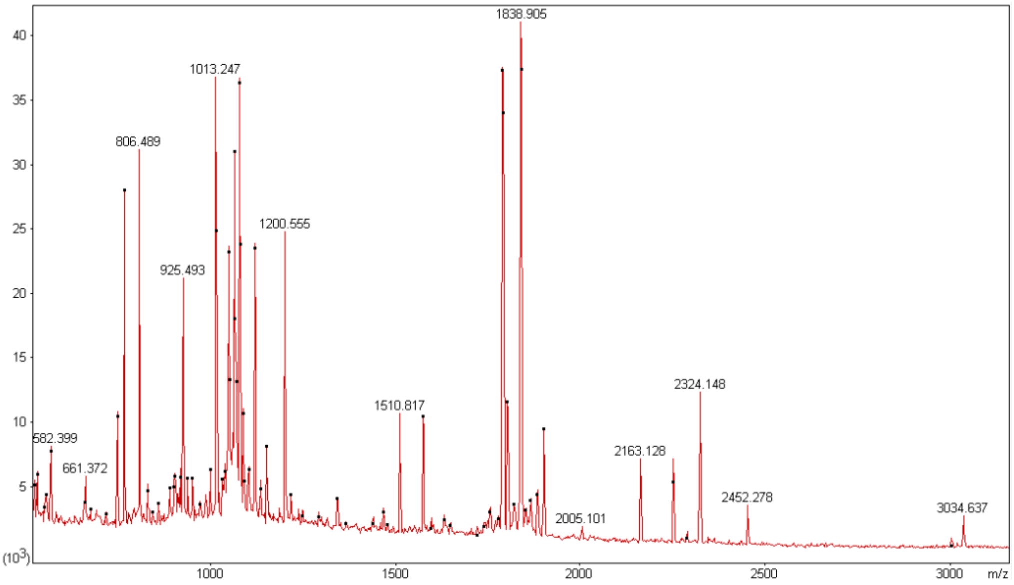

Probability Based Mowse Score:

Protein score is -10*Log(P), where P is the probability that the observed match is a random event. Protein scores greater than 61 are significant (p<0.05).

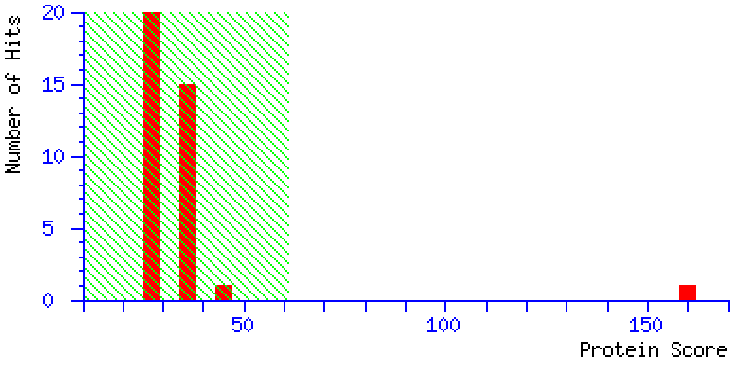

Spot No.: **89**

NCBI accession No.: **cassava4.1_003677m|PACid:17968299**

Plant species: ***Manihot esculenta*** Mascot score: **234**

Protein name： **V-type proton ATPase catalytic subunit A**

Sequence coverage %: **61** p value: **2.9e-019**

Matched peptides No.: **41** Total peptides No.: **95**

Calculated Mr: **68989** Calculated *p*I: **5.29**

Annotated PMF **spectra:**

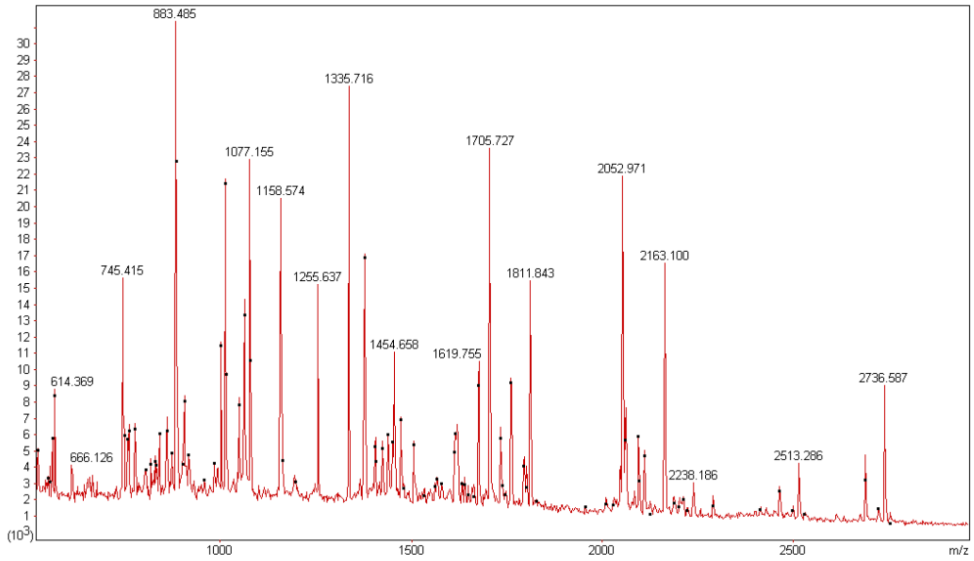

Probability Based Mowse Score:

Protein score is -10*Log(P), where P is the probability that the observed match is a random event. Protein scores greater than 61 are significant (p<0.05).

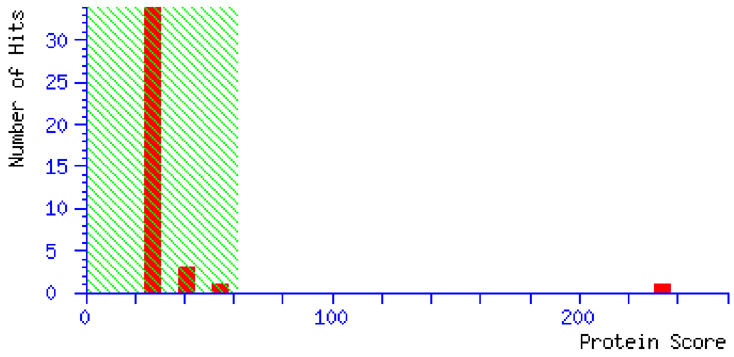

Spot No.: **92**

NCBI accession No.: **cassava4.1_004164m|PACid:17985770**

Plant species: ***Manihot esculenta***

Protein name: **malic enzyme, putative** Mascot score: **154**

Sequence coverage %: **35** p value: **2.9e-11**

Matched peptides No.: **26** Total peptides No.: **66**

Calculated Mr: **65246** Calculated *p*I: **6.03**

Annotated PMF **spectra:**

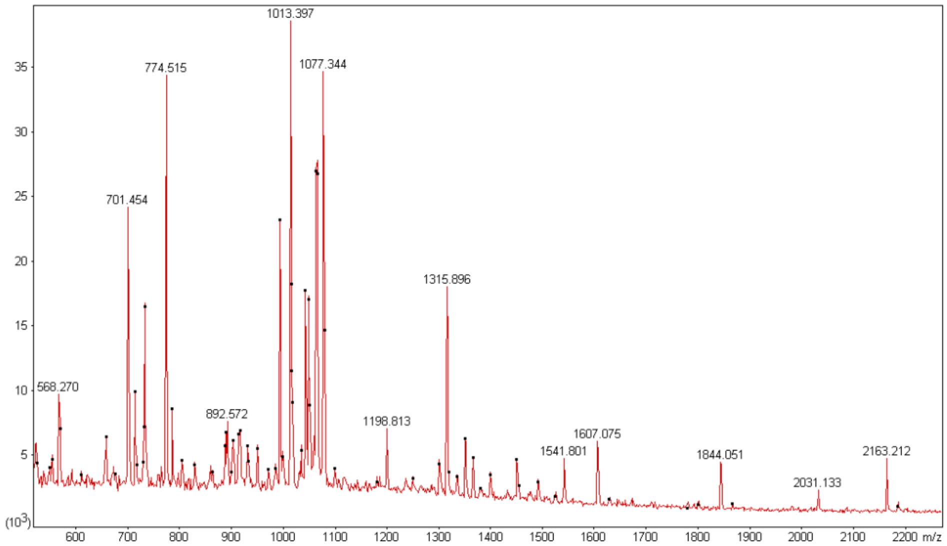

Probability Based Mowse Score:

Protein score is -10*Log(P), where P is the probability that the observed match is a random event. Protein scores greater than 61 are significant (p<0.05).

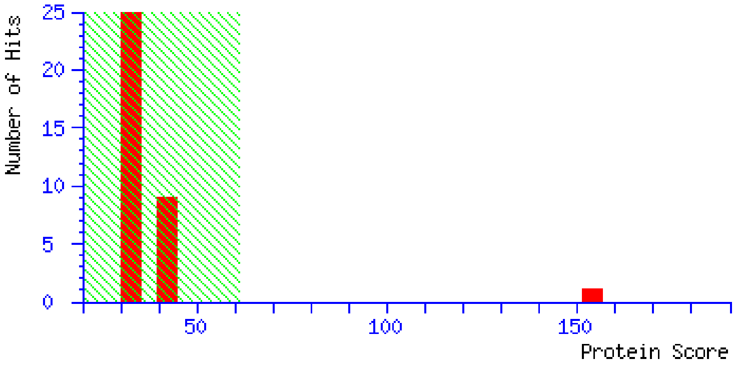

Spot No.: **94**

NCBI accession No.: **cassava4.1_002466m|PACid:17978799**

Plant species: ***Manihot esculenta***

Protein name: **Alpha-glucan phosphorylase 1**

PFF Mascot score: **[278]** Sequence coverage %: **[12]**

Matched peptides No.: **[7]** p value: **1.1e-007**

Calculated Mr: 81918 Calculated *p*I: **8.50**

Annotated PMF **spectra:**

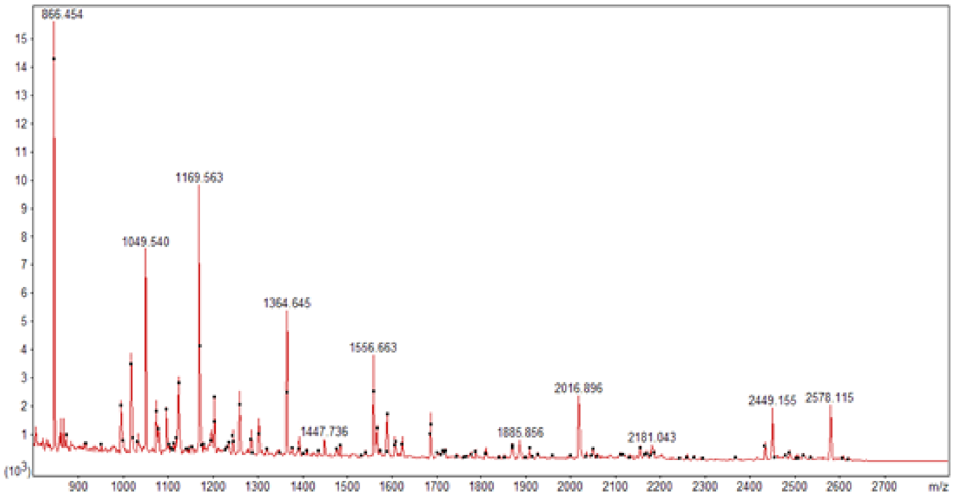

PFF Searched Score:

Ions score is -10*Log(P), where P is the probability that the observed match is a random event.
Individual ions scores > 34 indicate identity or extensive homology (p<0.05).
Protein scores are derived from ions scores as a non-probabilistic basis for ranking protein hits.

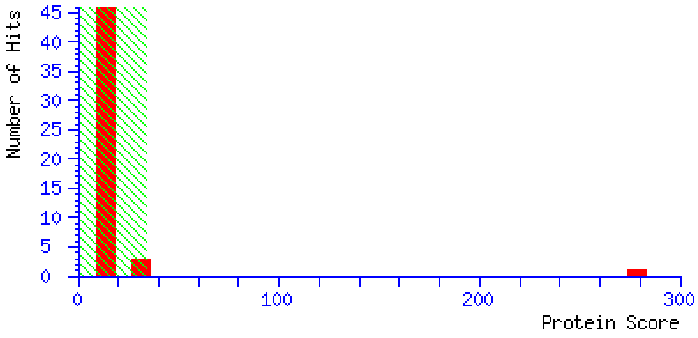

Spot No.: **97**

NCBI accession No.: **cassava4.1_011729m|PACid:17989246**

Plant species: ***Manihot esculenta***

Protein name: **nitrile-specifier protein 5-like**

PFF Mascot score: **[165]** Sequence coverage %: **[24]**

Matched peptides No.: **[7]** p value: **0.00081**

Calculated Mr: 35840 Calculated *p*I: **5.49**

Annotated PMF **spectra:**

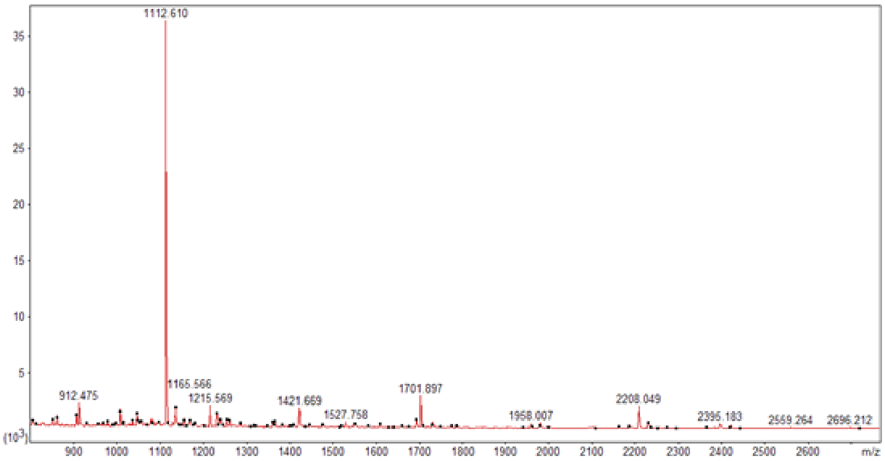

PFF Searched Score:

Ions score is -10*Log(P), where P is the probability that the observed match is a random event.
Individual ions scores > 33 indicate identity or extensive homology (p<0.05).
Protein scores are derived from ions scores as a non-probabilistic basis for ranking protein hits.

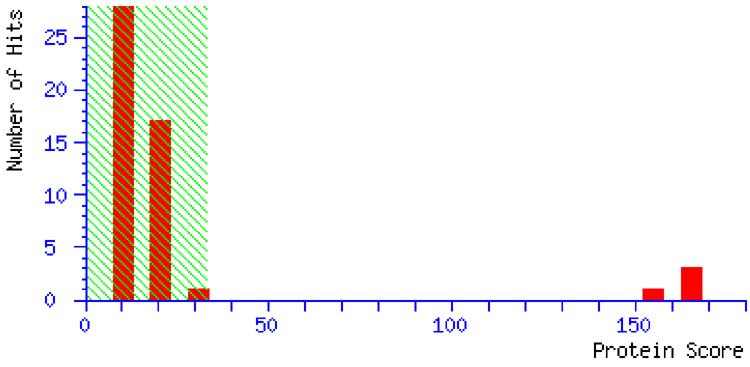

Spot No.: **105**

NCBI accession No.: **cassava4.1_017871m|PACid:17980723**

Plant species: ***Manihot esculenta***

Protein name： **18.1 kDa class I heat shock protein**

PFF Mascot score: **[142]** Sequence coverage %: **[23]**

Matched peptides No.: **[5]** p value: **0.00014**

Calculated Mr: 18750 Calculated *p*I: **7.93**

Annotated PMF **spectra:**

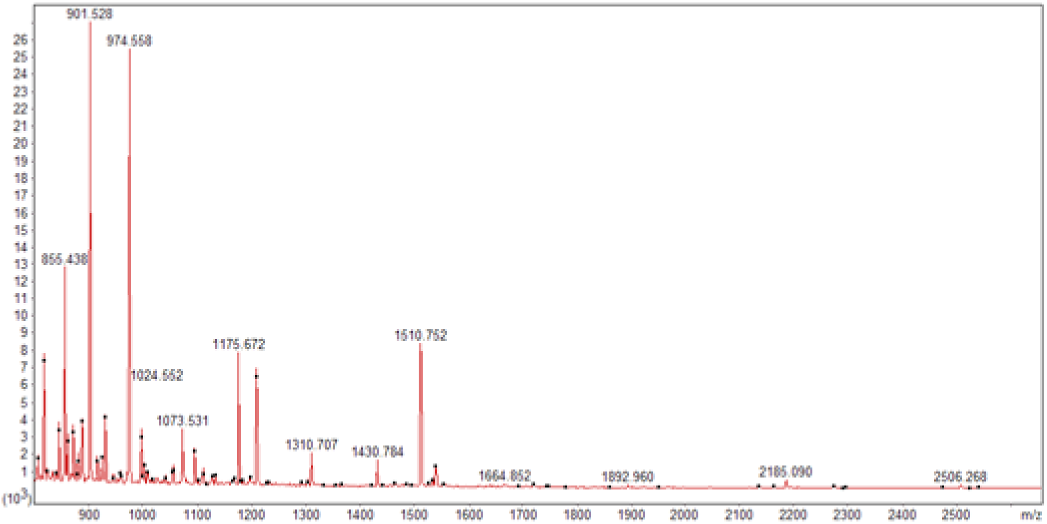

PFF Searched Score:

Ions score is -10*Log(P), where P is the probability that the observed match is a random event.
Individual ions scores > 34 indicate identity or extensive homology (p<0.05).
Protein scores are derived from ions scores as a non-probabilistic basis for ranking protein hits.

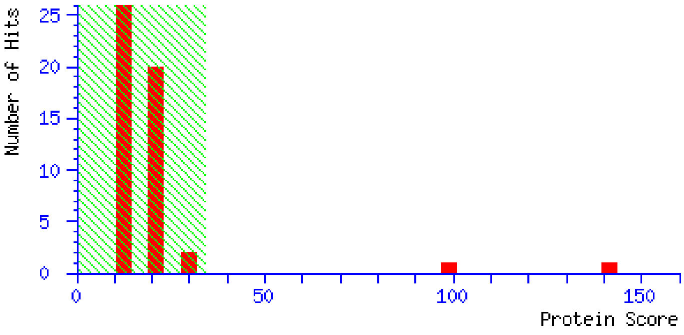

Spot No.: **113**

NCBI accession No.: **cassava4.1_009779m|PACid:17990964**

Plant species: ***Manihot esculenta***

Protein name：**Actin-7**

PFF Mascot score: **[440]** Sequence coverage %: **[36]**

Matched peptides No.: **[9]** p value: **2.9e-012**

Calculated Mr: 41897 Calculated *p*I: **5.31**

Annotated PMF spectra:

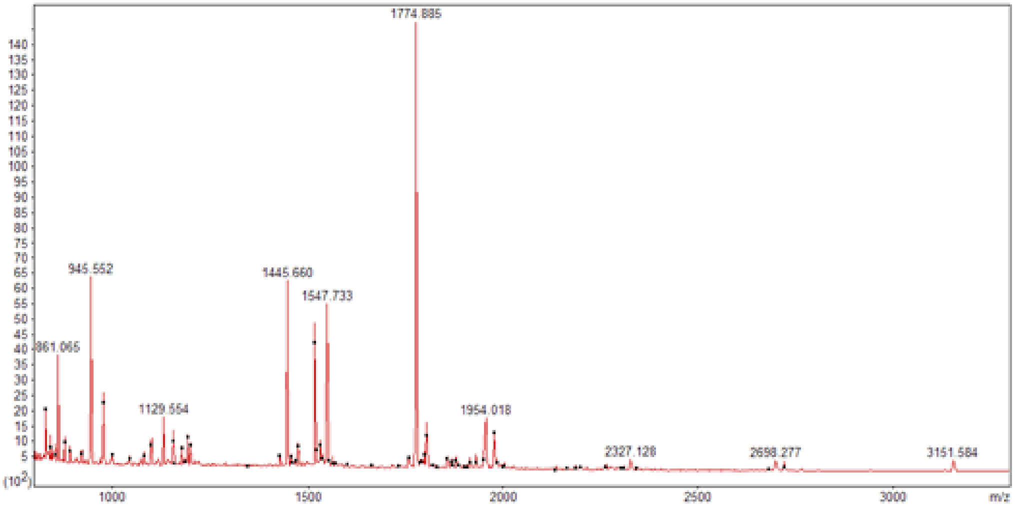

PFF Searched Score:

Ions score is -10*Log(P), where P is the probability that the observed match is a random event.
Individual ions scores > 33 indicate identity or extensive homology (p<0.05).
Protein scores are derived from ions scores as a non-probabilistic basis for ranking protein hits.

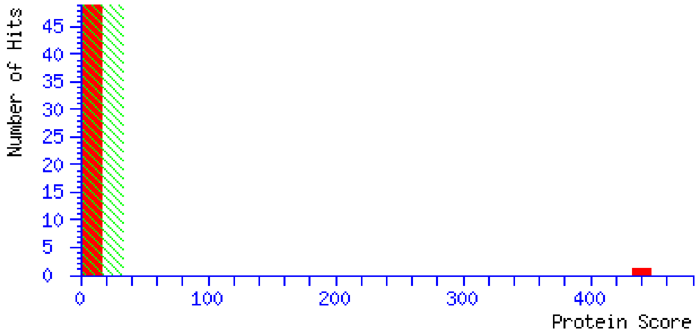

Spot No.: **118**

NCBI accession No.: **cassava4.1_005314m|PACid:17981128**

Plant species: ***Manihot esculenta***

Protein name：**Ketol-acid reductoisomerase, chloroplastic**

PFF Mascot score: **[214]** Sequence coverage %: **[9]**

Matched peptides No.: **[4]** p value: **9.6e-005**

Calculated Mr: **58515** Calculated *p*I: **5.50**

Annotated PMF **spectra:**

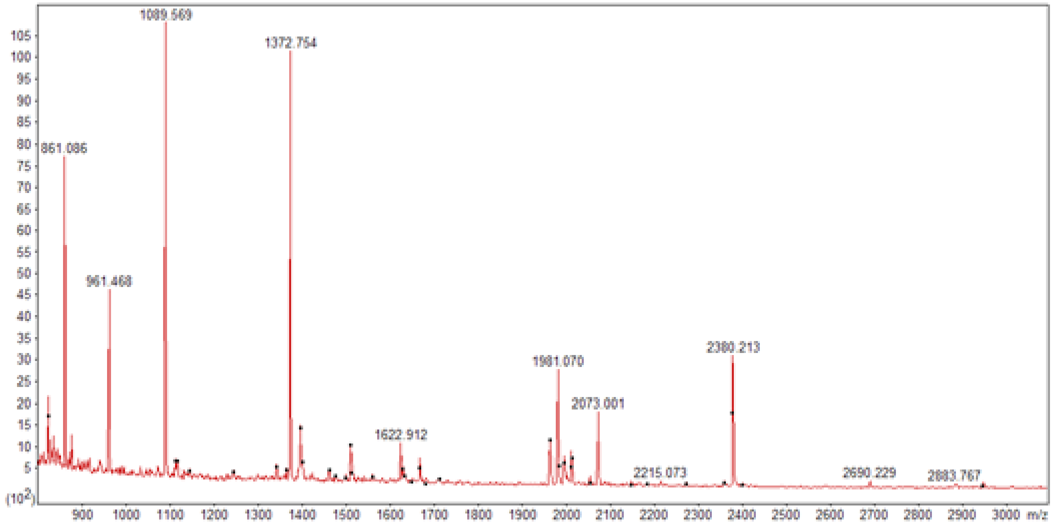

PFF Searched Score:

Ions score is -10*Log(P), where P is the probability that the observed match is a random event.
Individual ions scores > 33 indicate identity or extensive homology (p<0.05).
Protein scores are derived from ions scores as a non-probabilistic basis for ranking protein hits.

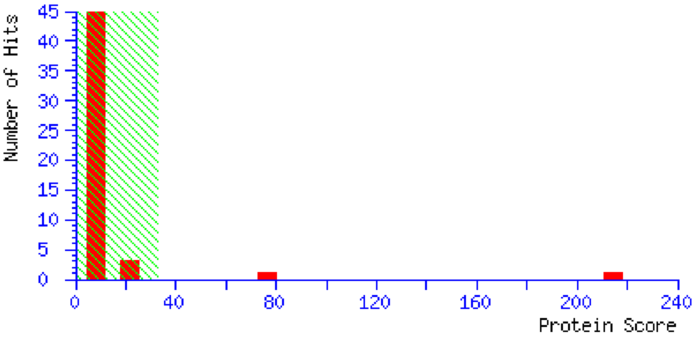

Spot No.: **130**

NCBI accession No.: **cassava4.1_001644m|PACid:17971276**

Plant species: ***Manihot esculenta***

Protein name： **At1g56070/T6H22_13**

PFF Mascot score: **[184]** Sequence coverage %: **[10]**

Matched peptides No.: **[7]** p value: **2.5e-005**

Calculated Mr: **95045** Calculated *p*I: **5.86**

Annotated PMF **spectra:**

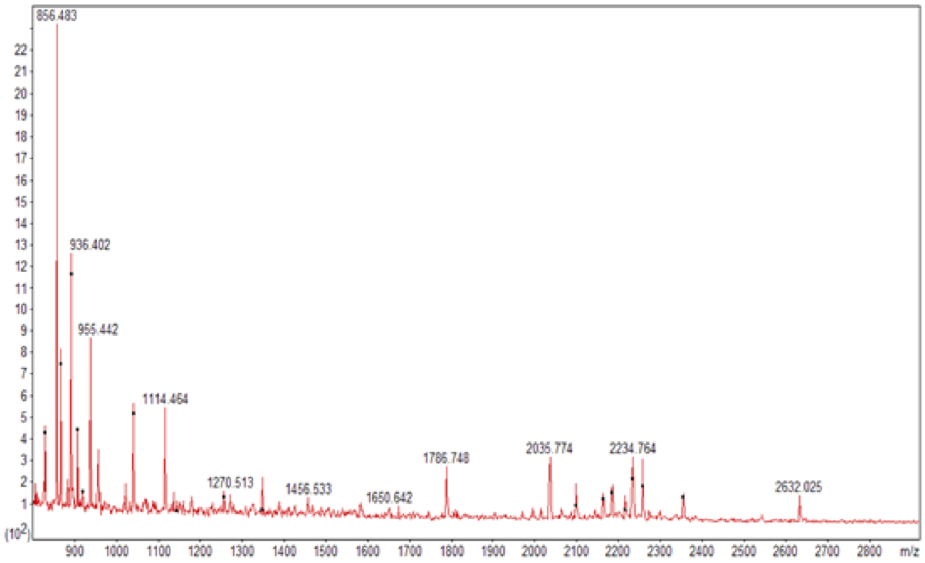

PFF Searched Score:

Ions score is -10*Log(P), where P is the probability that the observed match is a random event.
Individual ions scores > 33 indicate identity or extensive homology (p<0.05).
Protein scores are derived from ions scores as a non-probabilistic basis for ranking protein hits.

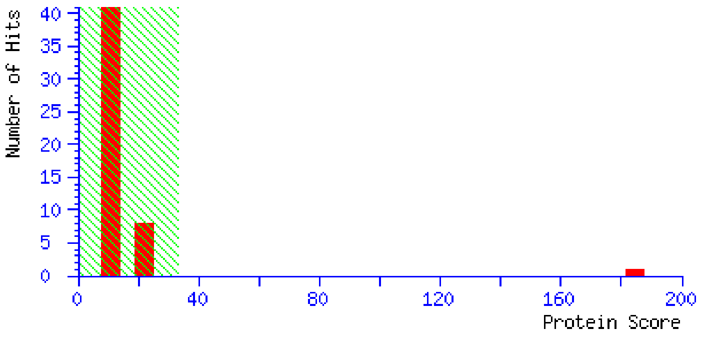

Spot No.: **131**

NCBI accession No.: **cassava4.1_002747m|PACid:17960141**

Plant species: ***Manihot esculenta***

Protein name: **Heat shock protein 90-3**

PFF Mascot score: **[181]** Sequence coverage %: **[7]**

Matched peptides No.: **[4]** p value: **0.00052**

Calculated Mr: **80250** Calculated *p*I: **4.99**

Annotated PMF **spectra:**

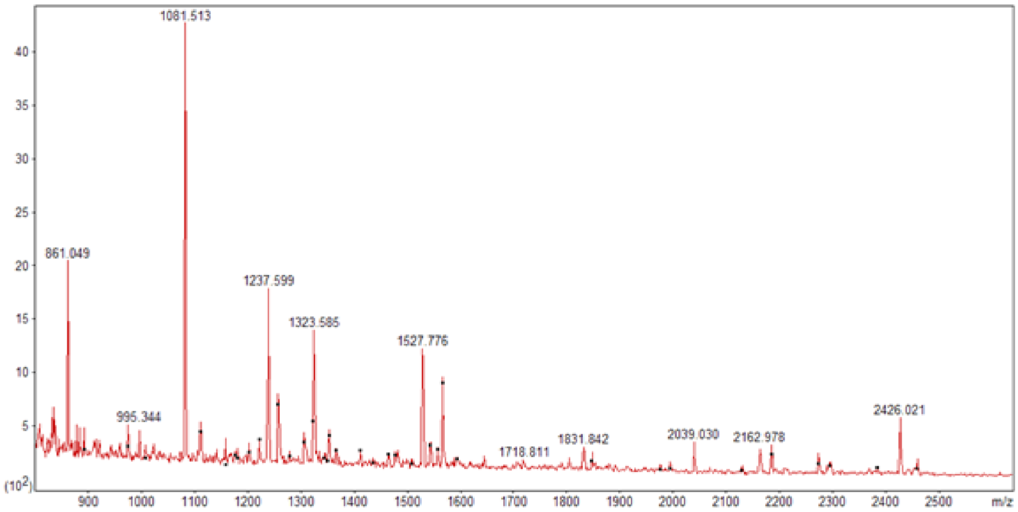

PFF Searched Score:

Ions score is -10*Log(P), where P is the probability that the observed match is a random event.
Individual ions scores > 33 indicate identity or extensive homology (p<0.05).
Protein scores are derived from ions scores as a non-probabilistic basis for ranking protein hits.

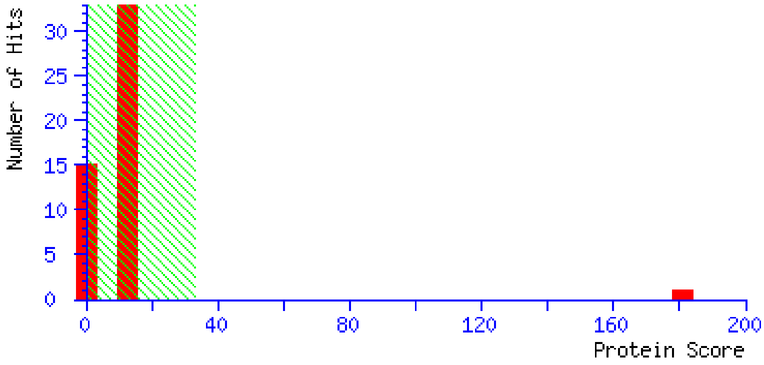

Spot No.: **132**

NCBI accession No.: **cassava4.1_002716m|PACid:17962449**

Plant species: ***Manihot esculenta***

Protein name： **Heat shock 70 kDa protein 7, chloroplastic**

PFF Mascot score: **[516]** Sequence coverage %: **[16]**

Matched peptides No.: **[9]** p value: **1.7e-012**

Calculated Mr: **75341** Calculated *p*I: **5.20**

Annotated PMF **spectra:**

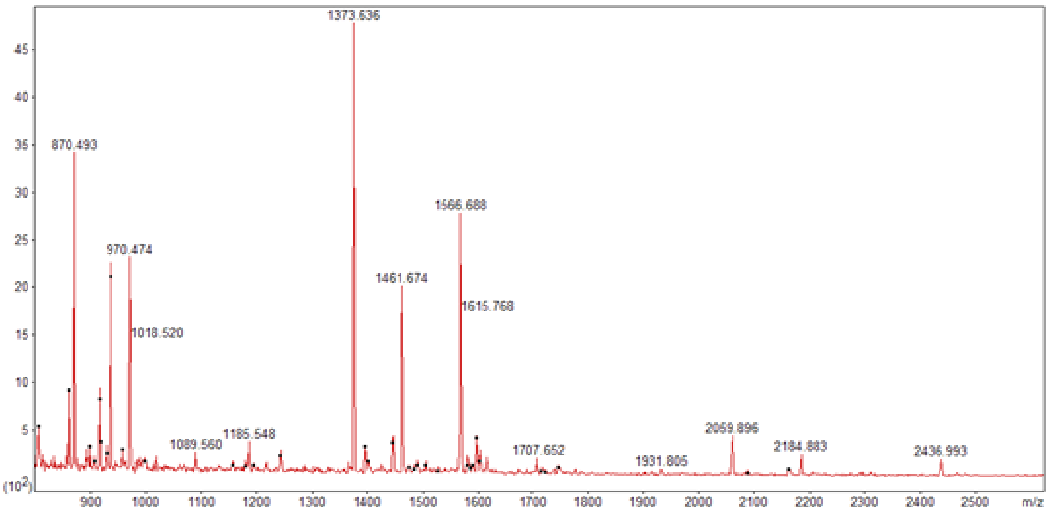

PFF Searched Score:

Ions score is -10*Log(P), where P is the probability that the observed match is a random event.
Individual ions scores > 34 indicate identity or extensive homology (p<0.05).
Protein scores are derived from ions scores as a non-probabilistic basis for ranking protein hits.

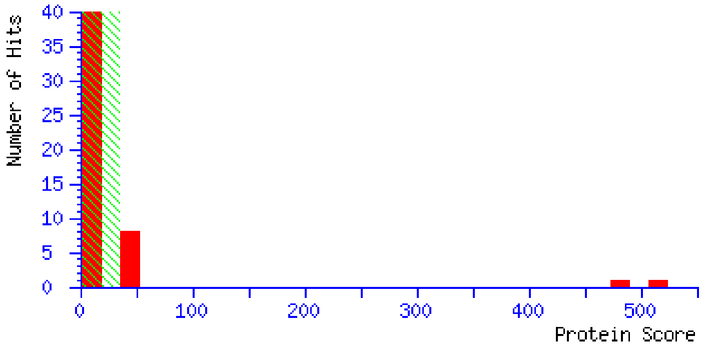

Spot No.: **133**

NCBI accession No.: **cassava4.1_002706m|PACid:17974818**

Plant species: ***Manihot esculenta***

Protein name: **Heat shock 70 kDa protein 7, chloroplastic**

PFF Mascot score: **[386]** Sequence coverage %: **[12]**

Matched peptides No.: **[7]** p value: **6.6e-011**

Calculated Mr: **75452** Calculated *p*I: **5.24**

Annotated PMF **spectra:**

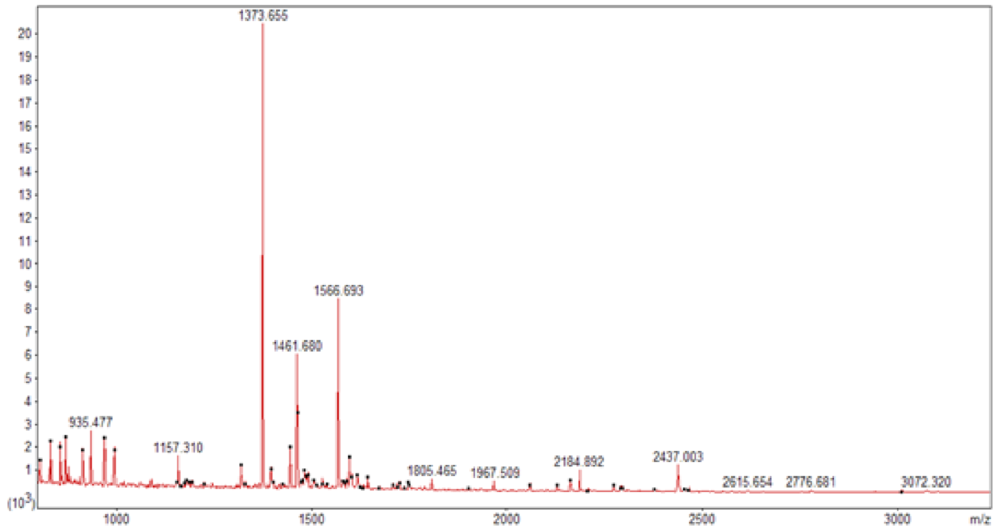

PFF Searched Score:

Ions score is -10*Log(P), where P is the probability that the observed match is a random event.
Individual ions scores > 34 indicate identity or extensive homology (p<0.05).
Protein scores are derived from ions scores as a non-probabilistic basis for ranking protein hits.

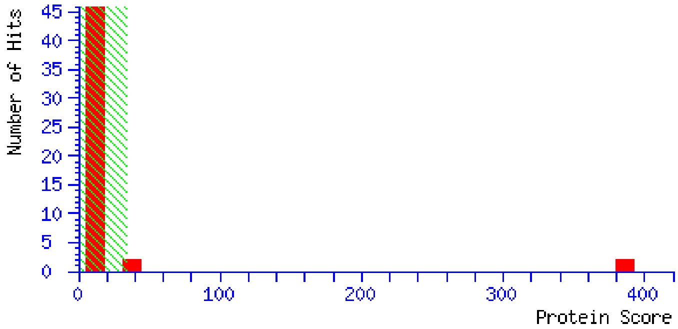

Spot No.: **134**

NCBI accession No.: **cassava4.1_005468m|PACid:17987918**

Plant species: ***Manihot esculenta***

Protein name：**Putative S9 Tyrosyl aminopeptidase**

PFF Mascot score: **[305]** Sequence coverage %: **[14]**

Matched peptides No.: **[6]** p value: **1.6e-007**

Calculated Mr: **59341** Calculated *p*I: **4.99**

Annotated PMF **spectra:**

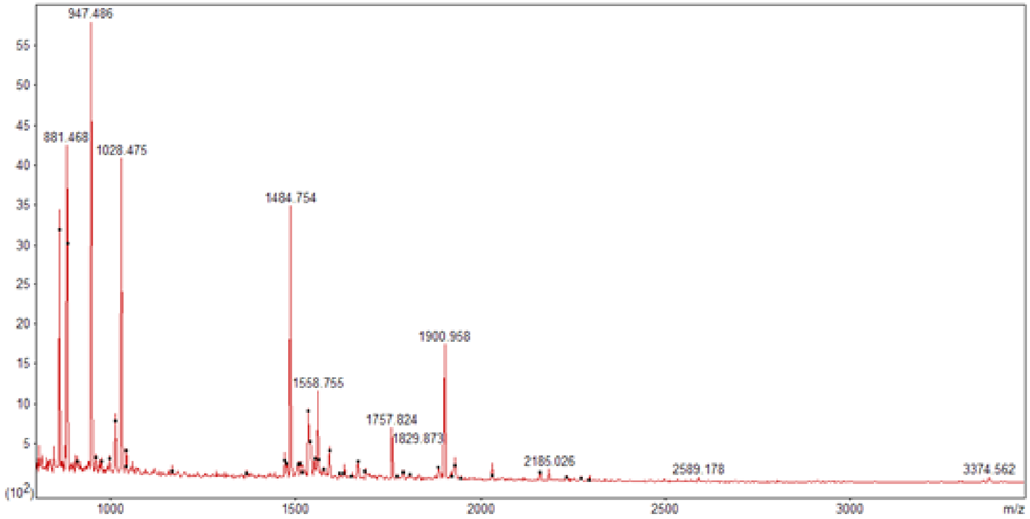

PFF Searched Score:

Ions score is -10*Log(P), where P is the probability that the observed match is a random event.
Individual ions scores > 34 indicate identity or extensive homology (p<0.05).
Protein scores are derived from ions scores as a non-probabilistic basis for ranking protein hits.

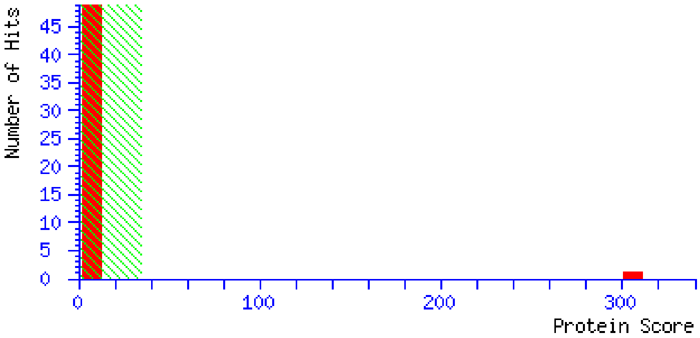

Spot No.: **135**

NCBI accession No.: **cassava4.1_002307m|PACid:17988755**

Plant species: ***Manihot esculenta***

Protein name: **Transketolase-2, chloroplastic**

PFF score: **78** Sequence coverage %: [**7**]

Matched peptides No.: [**2**] p value: **0.0001**

Calculated Mr: **80925** Calculated *p*I: **6.31**

Annotated PMF **spectra:**

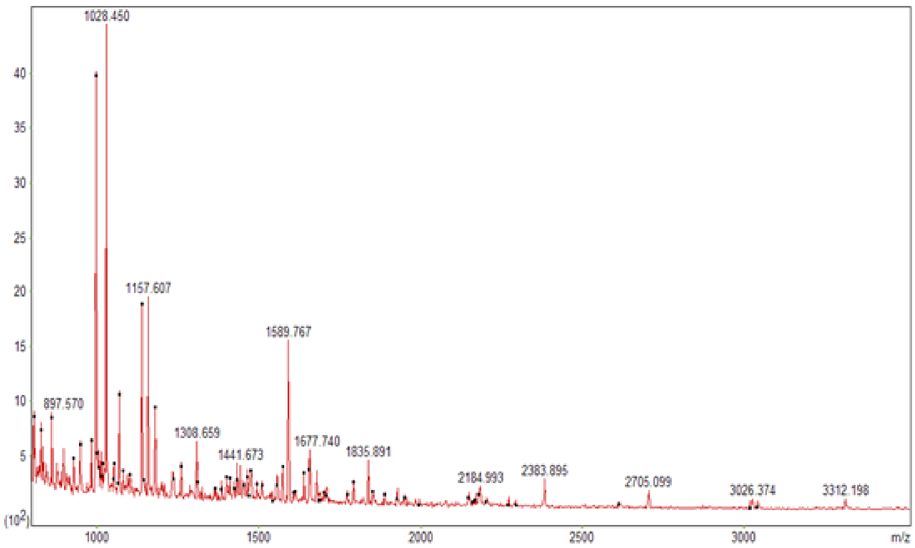

PFF Searched Score:

Ions score is -10*Log(P), where P is the probability that the observed match is a random event.
Individual ions scores > 32 indicate identity or extensive homology (p<0.05).
Protein scores are derived from ions scores as a non-probabilistic basis for ranking protein hits.

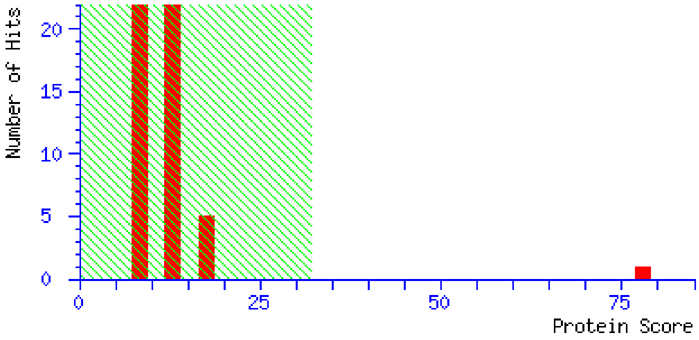

Spot No.: **136**

NCBI accession No.: **cassava4.1_006409m|PACid:17968834**

Plant species: ***Manihot esculenta***

Protein name：**V-type proton ATPase subunit B3**

PFF Mascot score: **[78]** Sequence coverage %: **[8]**

Matched peptides No.: **[2]** p value: **0.0009**

Calculated Mr: **54514** Calculated *p*I: **4.91**

Annotated PMF **spectra:**

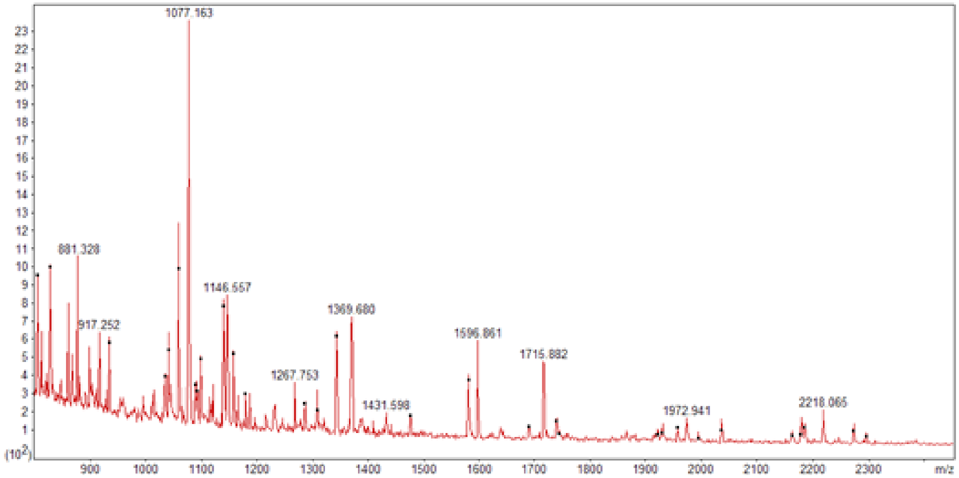

PFF Searched Score:

Ions score is -10*Log(P), where P is the probability that the observed match is a random event.
Individual ions scores > 32 indicate identity or extensive homology (p<0.05).
Protein scores are derived from ions scores as a non-probabilistic basis for ranking protein hits.

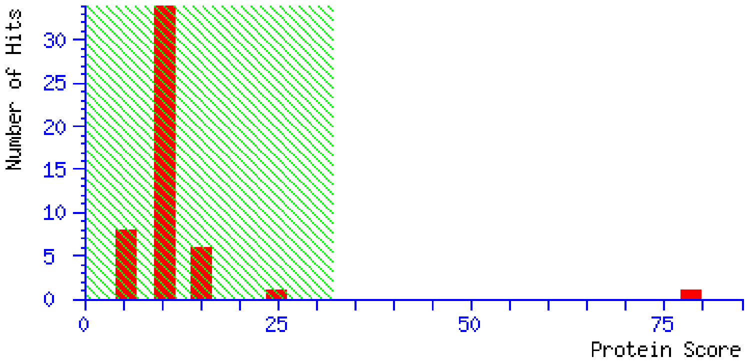

Spot No.: **137**

NCBI accession No.: **cassava4.1_007650m|PACid:17967223**

Plant species: ***Manihot esculenta***

Protein name：**Tubulin beta-6 chain**

PFF Mascot score: **[193]** Sequence coverage %: **[11]**

Matched peptides No.: **[5]** p value: **0.00015**

Calculated Mr: **50741** Calculated *p*I: **4.77**

Annotated PMF **spectra:**

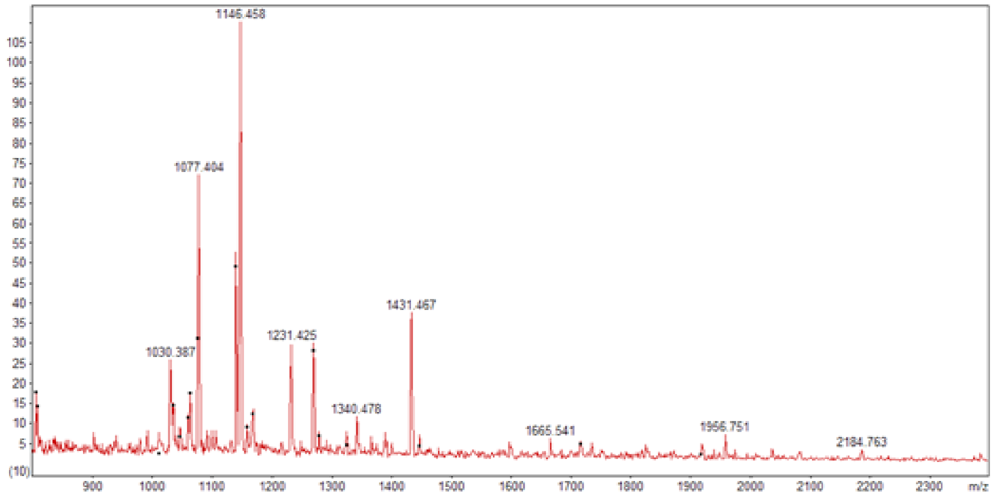

PFF Searched Score:

Ions score is -10*Log(P), where P is the probability that the observed match is a random event.
Individual ions scores > 34 indicate identity or extensive homology (p<0.05).
Protein scores are derived from ions scores as a non-probabilistic basis for ranking protein hits.

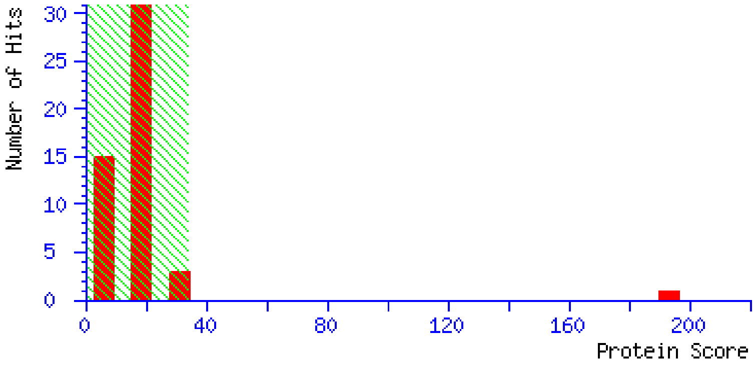

Spot No.: **138**

NCBI accession No.: **cassava4.1_005250m|PACid:17973850**

Plant species: ***Manihot esculenta***

Protein name: **Importin subunit alpha-1**

PFF Mascot score: **[195]** Sequence coverage %: **[13]**

Matched peptides No.: **[4]** p value: **1.6e-009**

Calculated Mr: **59677** Calculated *p*I: **5.30**

Annotated PMF **spectra:**

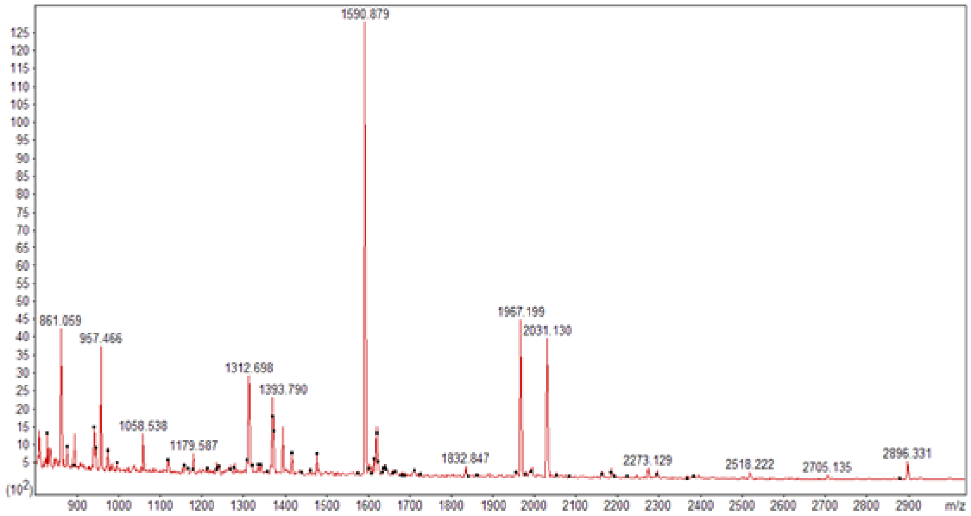

PFF Searched Score:

Ions score is -10*Log(P), where P is the probability that the observed match is a random event.
Individual ions scores > 34 indicate identity or extensive homology (p<0.05).
Protein scores are derived from ions scores as a non-probabilistic basis for ranking protein hits.

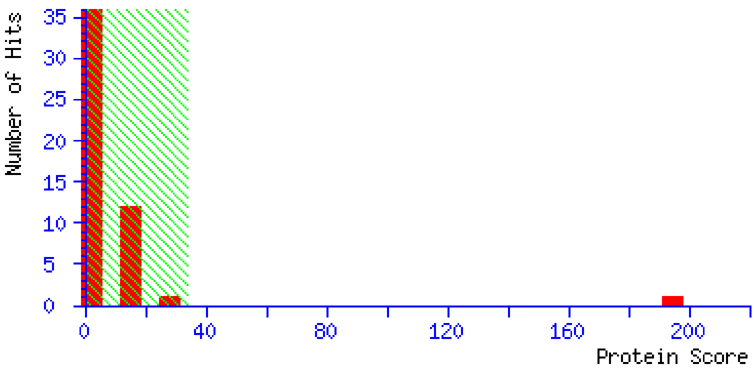

Spot No.: **139**

NCBI accession No.: **cassava4.1_007640m|PACid:17979054**

Plant species: ***Manihot esculenta***

Protein name: **Tubulin beta-6 chain**

PFF Mascot score: **[170]** Sequence coverage %: **[10]**

Matched peptides No.: **[4]** p value: **1.1e-008**

Calculated Mr: **50722** Calculated *p*I: **4.68**

Annotated PMF **spectra:**

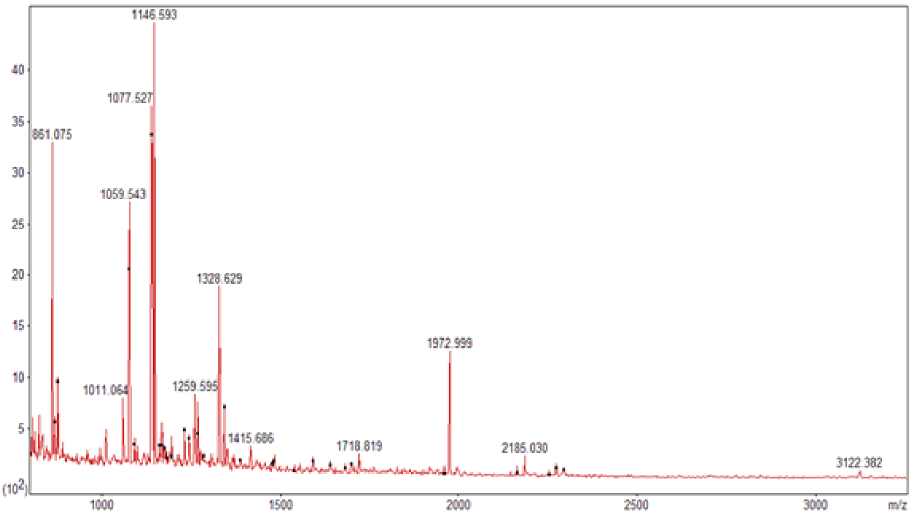

PFF Searched Score:

Ions score is -10*Log(P), where P is the probability that the observed match is a random event.
Individual ions scores > 33 indicate identity or extensive homology (p<0.05).
Protein scores are derived from ions scores as a non-probabilistic basis for ranking protein hits.

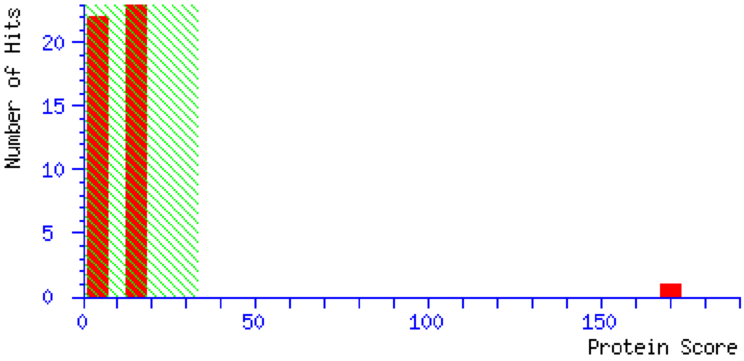

Spot No.: **140**

NCBI accession No.: **cassava4.1_006508m|PACid:17967572**

Plant species：Manihot esculenta

Protein name：**Adenosylhomocysteinase 1**

PFF Mascot score: **[65]** Sequence coverage %: **[10]**

Matched peptides No.: **[4]** p value: **0.0011**

Calculated Mr: **53894** Calculated *p*I: **5.86**

Annotated PMF **spectra:**

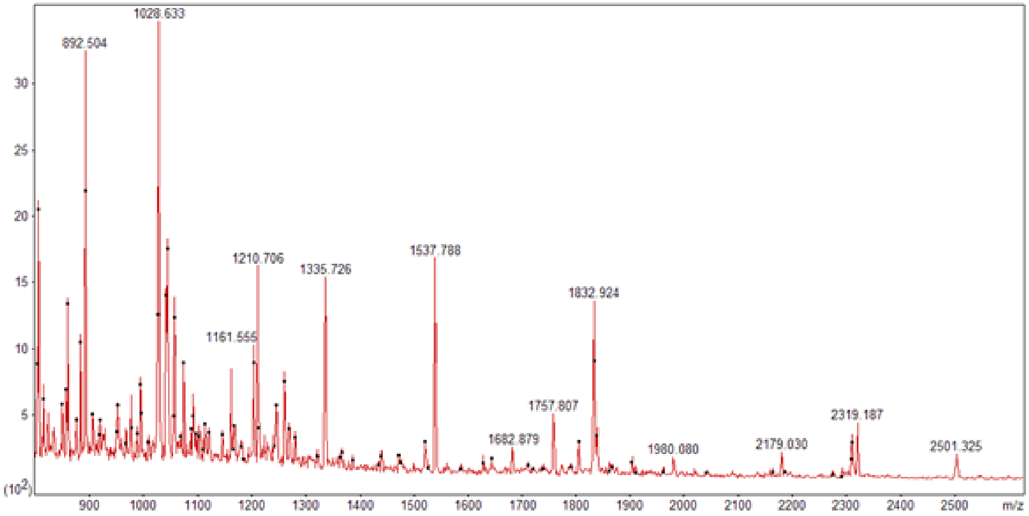

PFF Searched Score:

Ions score is -10*Log(P), where P is the probability that the observed match is a random event.
Individual ions scores > 34 indicate identity or extensive homology (p<0.05).
Protein scores are derived from ions scores as a non-probabilistic basis for ranking protein hits.

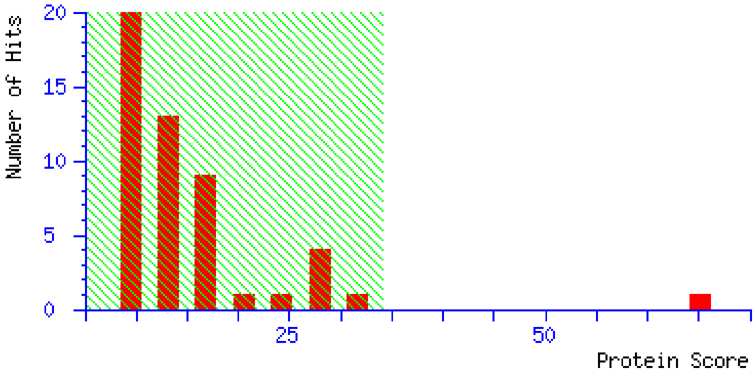

Spot No.: **141**

NCBI accession No.: cassava4.1_020254m|PACid:17972349

Plant species: ***Manihot esculenta***

Protein name： **ribulose 1,5 bisphosphate carboxylase-oxygenase**

PFF Mascot score: **[153]** Sequence coverage %: **[7]**

Matched peptides No.: **[3]** p value: **0.00069**

Calculated Mr: **51876** Calculated *p*I: **5.70**

Annotated PMF **spectra:**

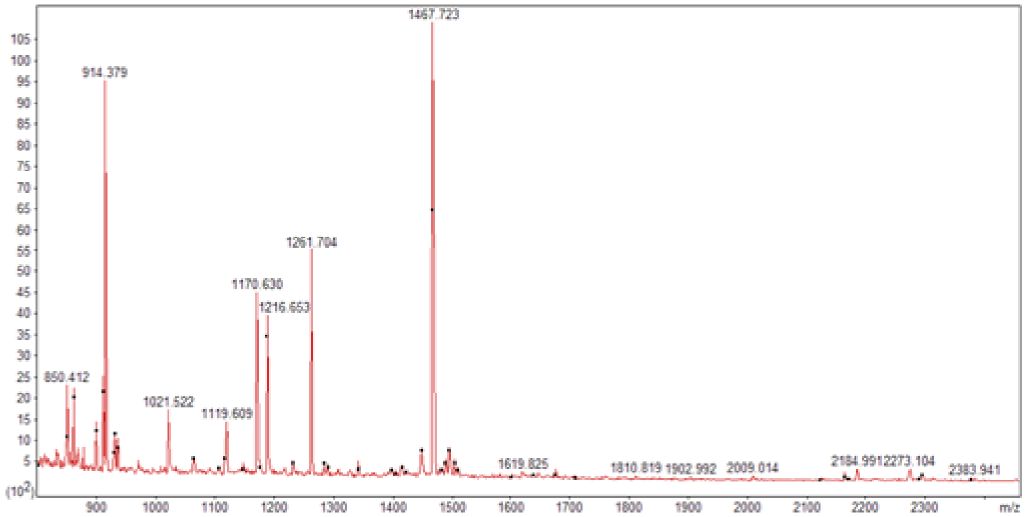

PFF Searched Score:

Ions score is -10*Log(P), where P is the probability that the observed match is a random event.
Individual ions scores > 49 indicate identity or extensive homology (p<0.05).
Protein scores are derived from ions scores as a non-probabilistic basis for ranking protein hits.

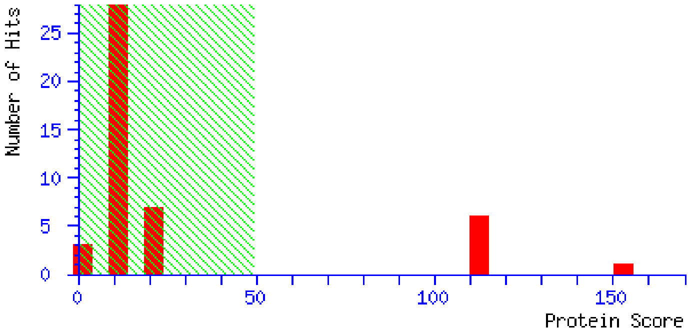

Spot No.: **142**

NCBI accession No.: **cassava4.1_007756m|PACid:17993326**

Plant species: ***Manihot esculenta***

Protein name：**Aldolase-type TIM barrel family protein**

PFF Mascot score: **[94]** Sequence coverage %: **[10]**

Matched peptides No.: **[4]** p value: **0.00026**

Calculated Mr: **48525** Calculated *p*I: **6.19**

Annotated PMF **spectra:**

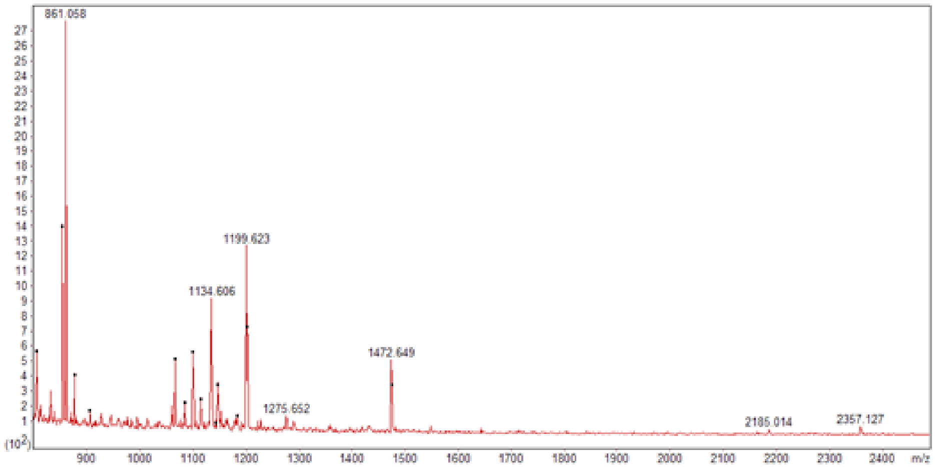

PFF Searched Score:

Ions score is -10*Log(P), where P is the probability that the observed match is a random event.
Individual ions scores > 33 indicate identity or extensive homology (p<0.05).
Protein scores are derived from ions scores as a non-probabilistic basis for ranking protein hits.

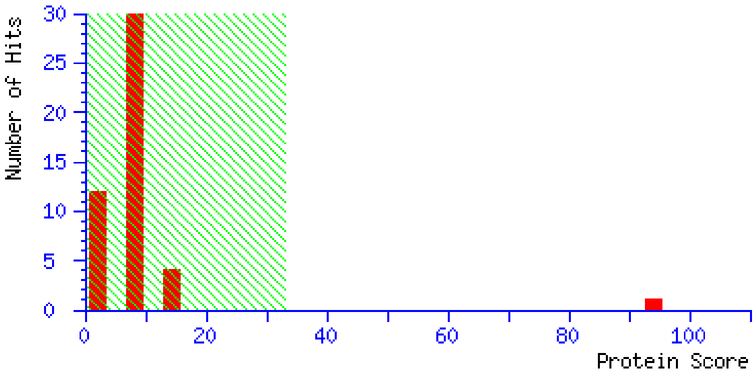

Spot No.: **143**

NCBI accession No.: **cassava4.1_026035m|PACid:17968666**

Plant species: ***Manihot esculenta***

Protein name: **Alcohol dehydrogenase class-P**

PFF Mascot score: **[152]** Sequence coverage %: **[20]**

Matched peptides No.: **[5]** p value: **0.00014**

Calculated Mr: **38296** Calculated *p*I: **6.02**

Annotated PMF **spectra:**

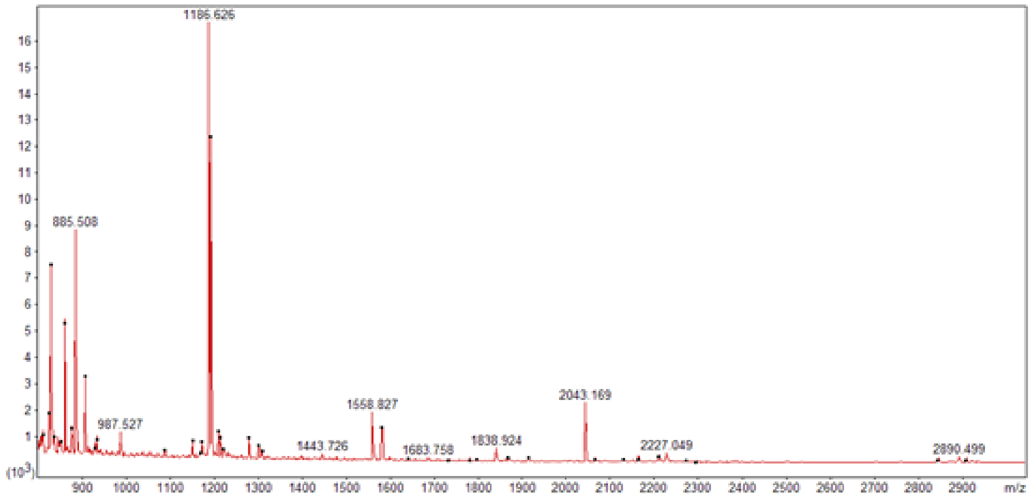

PFF Searched Score:

Ions score is -10*Log(P), where P is the probability that the observed match is a random event.
Individual ions scores > 33 indicate identity or extensive homology (p<0.05).
Protein scores are derived from ions scores as a non-probabilistic basis for ranking protein hits.

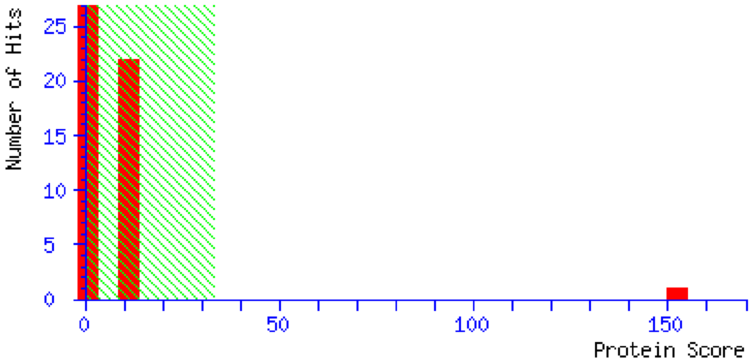

Spot No.: **144**

NCBI accession No.: **cassava4.1_014881m|PACid:17982856**

Plant species: ***Manihot esculenta***

Protein name： **acetylornithine deacetylase-like**

PFF Mascot score: **[61]** Sequence coverage %: **[6]**

Matched peptides No.: **[2]** p value: **7.1e-005**

Calculated Mr: **26425** Calculated *p*I: **5.50**

Annotated PMF **spectra:**

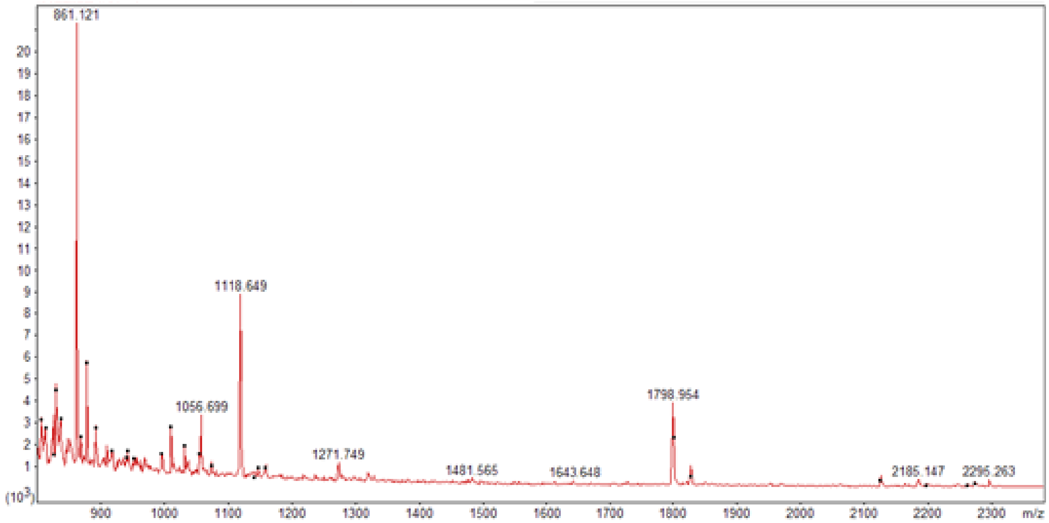

PFF Searched Score:

Ions score is -10*Log(P), where P is the probability that the observed match is a random event.
Individual ions scores > 34 indicate identity or extensive homology (p<0.05).
Protein scores are derived from ions scores as a non-probabilistic basis for ranking protein hits.

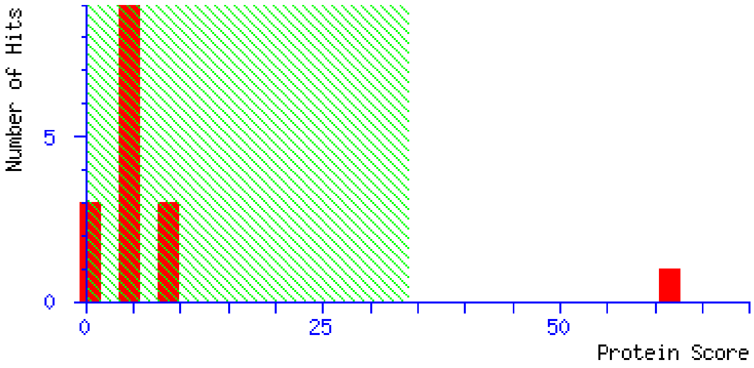

Spot No.: **145**

NCBI accession No.: **cassava4.1_009780m|PACid:17993116**

Plant species: ***Manihot esculenta***

Protein name： **Actin-7**

PFF Mascot score: **[115]** Sequence coverage %: **[6]**

Matched peptides No.: **[2]** p value: **2.6e-06**

Calculated Mr: **42246** Calculated *p*I: **5.64**

Annotated PMF **spectra:**

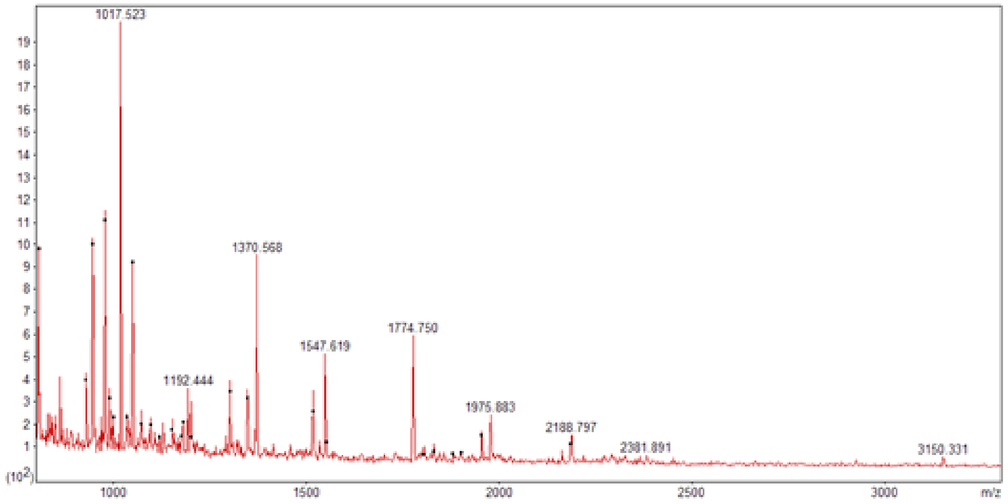

PFF Searched Score:

Ions score is -10*Log(P), where P is the probability that the observed match is a random event.
Individual ions scores > 33 indicate identity or extensive homology (p<0.05).
Protein scores are derived from ions scores as a non-probabilistic basis for ranking protein hits.

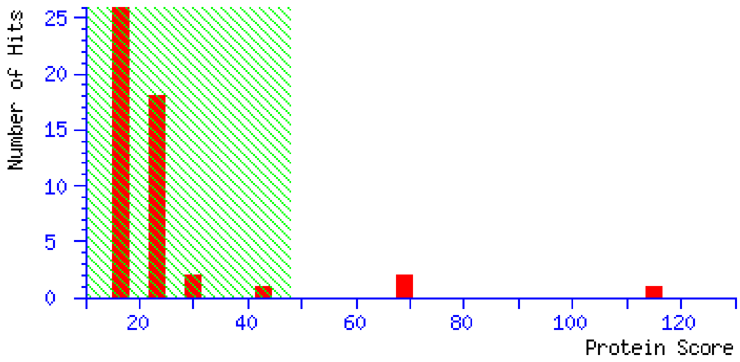

Spot No.: **146**

NCBI accession No.: **cassava4.1_010334m|PACid:17962764**

Plant species: ***Manihot esculenta***

Protein name： **UDP-arabinopyranose mutase 3**

PFF Mascot score: **[73]** Sequence coverage %: **[8]**

Matched peptides No.: **[2]** p value: **4.4e-006**

Calculated Mr: **41616** Calculated *p*I: **5.82**

Annotated PMF **spectra:**

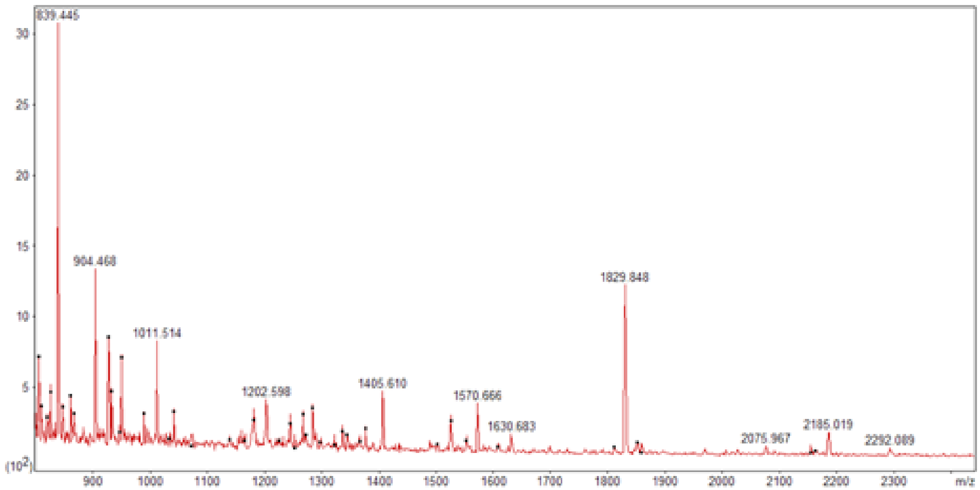

PFF Searched Score:

Ions score is -10*Log(P), where P is the probability that the observed match is a random event.
Individual ions scores > 31 indicate identity or extensive homology (p<0.05).
Protein scores are derived from ions scores as a non-probabilistic basis for ranking protein hits.

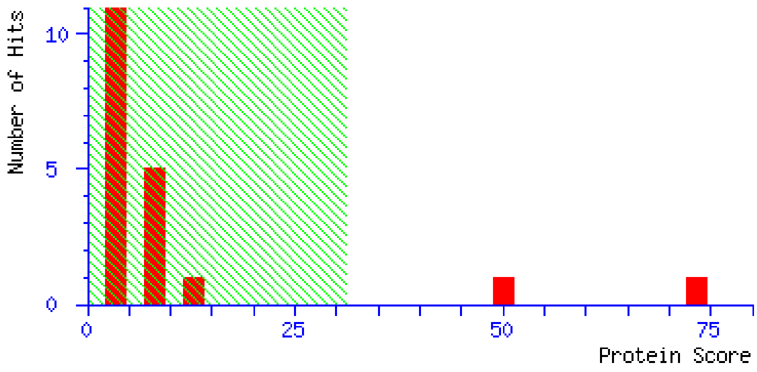

Spot No.: **147**

NCBI accession No.: **cassava4.1_006605m|PACid:17968275**

Plant species: ***Arabidopsis thaliana***

Protein name：**Phosphoglycerate kinase 1, chloroplastic**

PFF Mascot score: **[103]** Sequence coverage %: **[9]**

Matched peptides No.: **[4]** p value: **0.00031**

Calculated Mr: **50372** Calculated *p*I: **8.26**

Annotated PMF **spectra:**

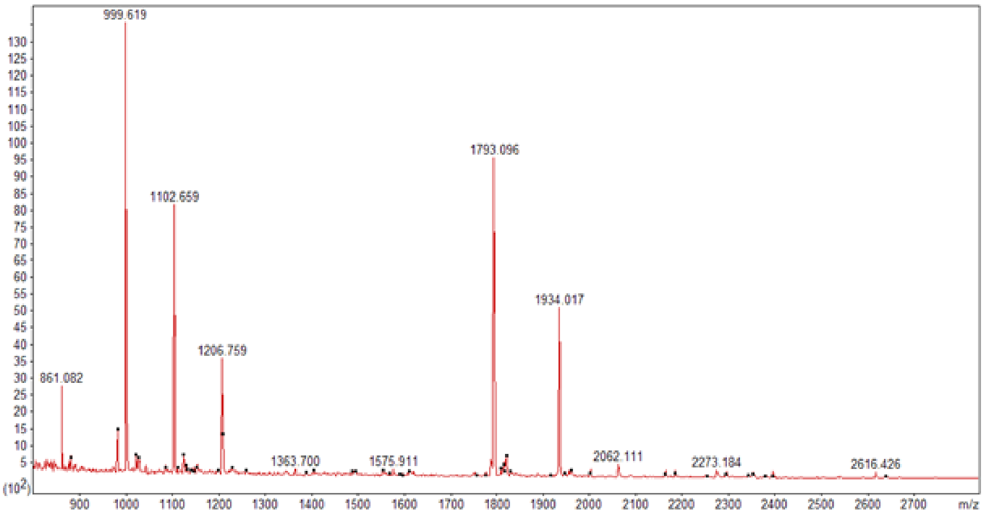

PFF Searched Score:

Ions score is -10*Log(P), where P is the probability that the observed match is a random event.
Individual ions scores > 33 indicate identity or extensive homology (p<0.05).
Protein scores are derived from ions scores as a non-probabilistic basis for ranking protein hits.

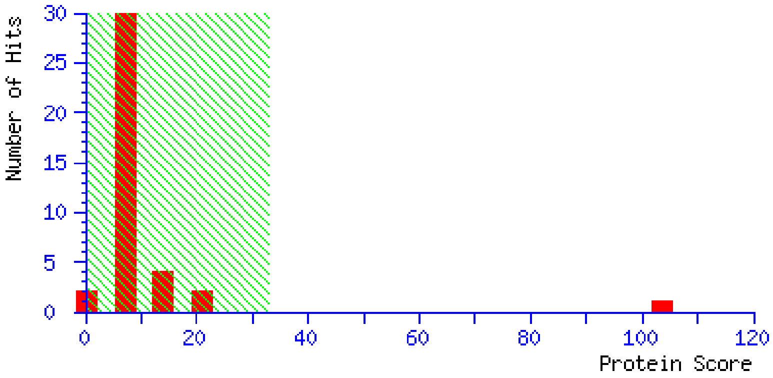

Spot No.: **148**

NCBI accession No.: **cassava4.1_009233m|PACid:17991143**

Plant species: ***Manihot esculenta***

Protein name: **Probable fructose-bisphosphate aldolase 3**

PFF Mascot score: **[380]** Sequence coverage %: **[20]**

Matched peptides No.: **[7]** p value: **1.5e-010**

Calculated Mr: **43050** Calculated *p*I: **8.63**

Annotated PMF **spectra:**

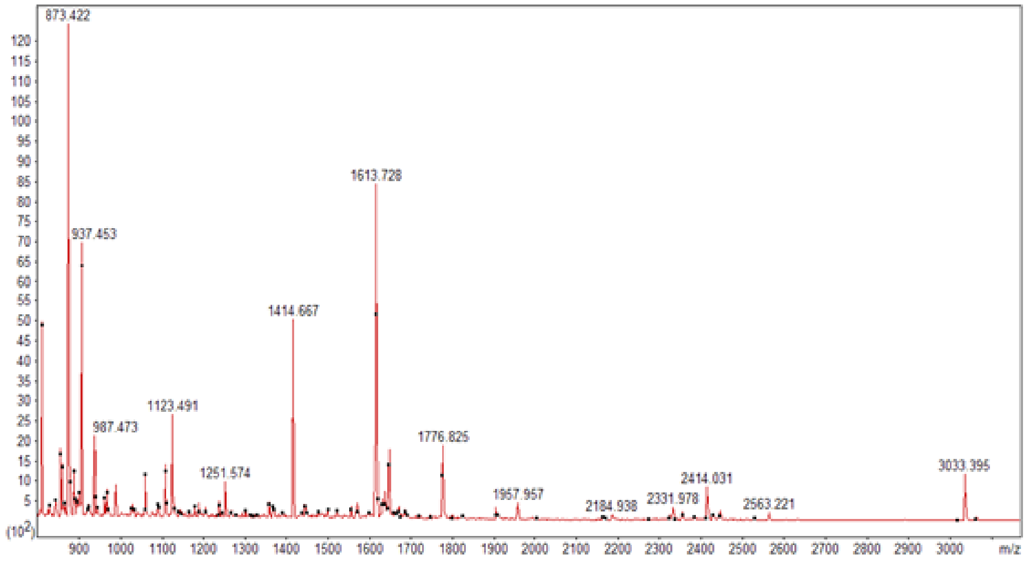

PFF Searched Score:

Ions score is -10*Log(P), where P is the probability that the observed match is a random event.
Individual ions scores > 33 indicate identity or extensive homology (p<0.05).
Protein scores are derived from ions scores as a non-probabilistic basis for ranking protein hits.

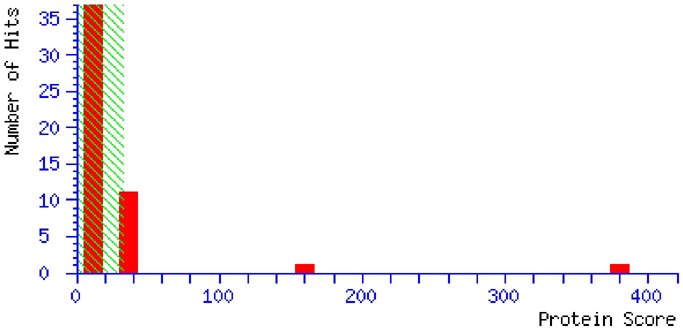

Spot No.: **149**

NCBI accession No.: **cassava4.1_009217m|PACid:17976496**

Plant species: ***Manihot esculenta***

Protein name：**fructose-bisphosphate aldolase, putative**

PFF Mascot score: **[118]** Sequence coverage %: **[15]**

Matched peptides No.: **[4]** p value: **0.00029**

Calculated Mr: **24559** Calculated *p*I: **8.93**

Annotated PMF **spectra:**

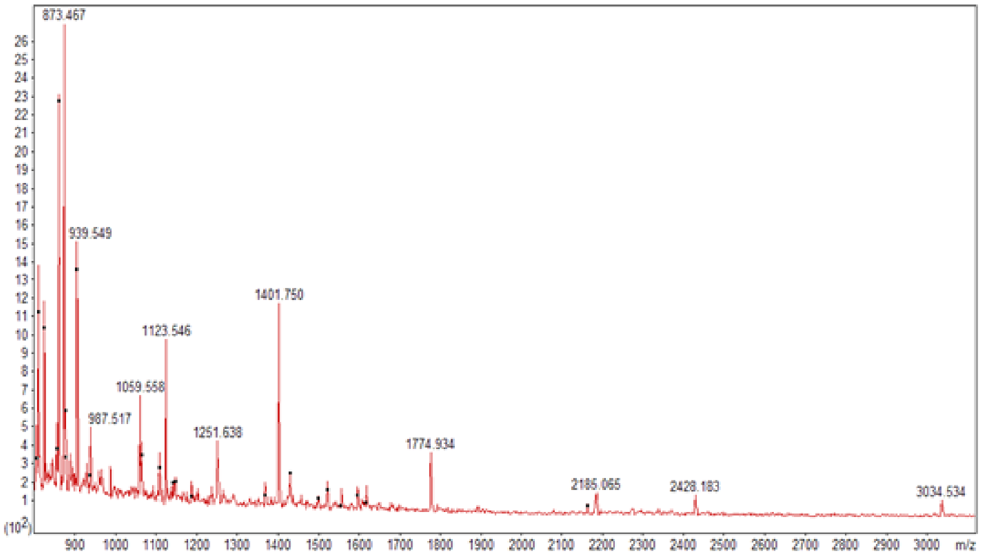

PFF Searched Score:

Ions score is -10*Log(P), where P is the probability that the observed match is a random event.
Individual ions scores > 34 indicate identity or extensive homology (p<0.05).
Protein scores are derived from ions scores as a non-probabilistic basis for ranking protein hits.

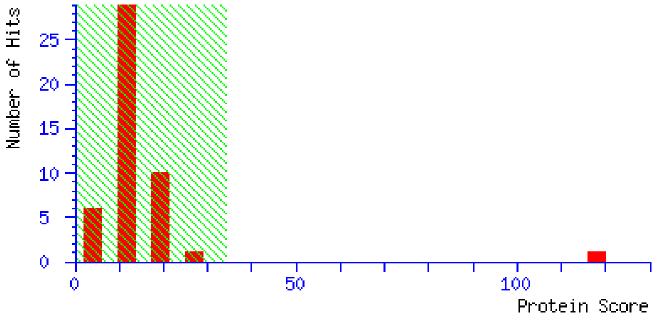

Spot No.: **150**

NCBI accession No.: **cassava4.1_022763m|PACid:17989911**

Plant species: ***Manihot esculenta***

Protein name： **aldo/keto reductase AKR**

PFF Mascot score: **[69]** Sequence coverage %: **[10]**

Matched peptides No.: **[2]** p value: **9.7e-005**

Calculated Mr: **15199** Calculated *p*I: **7.72**

Annotated PMF **spectra:**

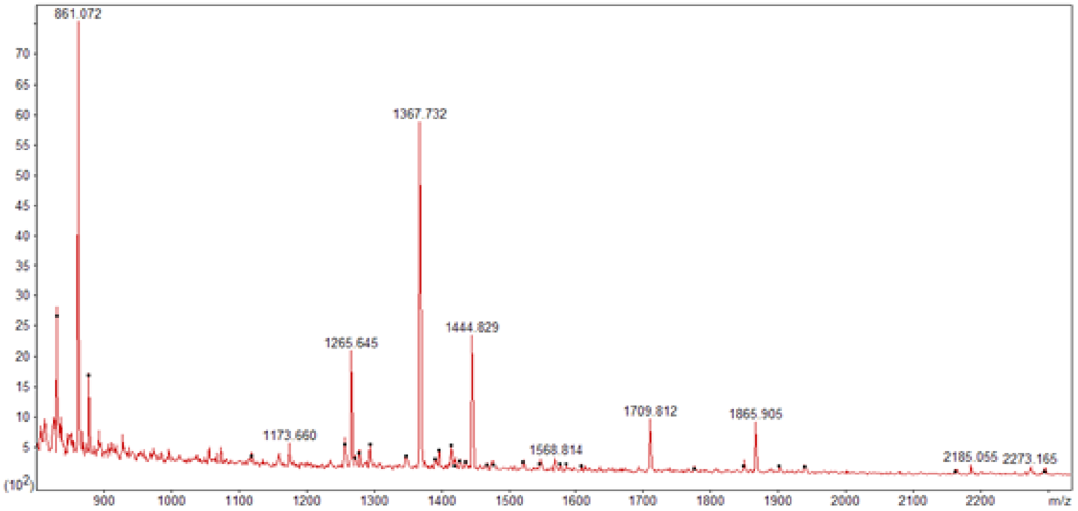

PFF Searched Score:

Ions score is -10*Log(P), where P is the probability that the observed match is a random event.
Individual ions scores > 33 indicate identity or extensive homology (p<0.05).
Protein scores are derived from ions scores as a non-probabilistic basis for ranking protein hits.

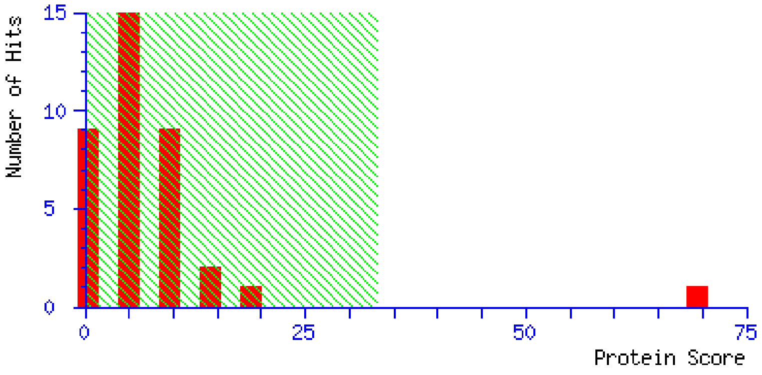

Spot No.: **151**

NCBI accession No.: **cassava4.1_011584m|PACid:17982732**

Plant species: ***Manihot esculenta***

Protein name： **Probable fructokinase-1**

PFF Mascot score: **[110]** Sequence coverage %: **[16]**

Matched peptides No.: **[4]** p value: **0.00064**

Calculated Mr: **35621** Calculated *p*I: **5.12**

Annotated PMF **spectra:**

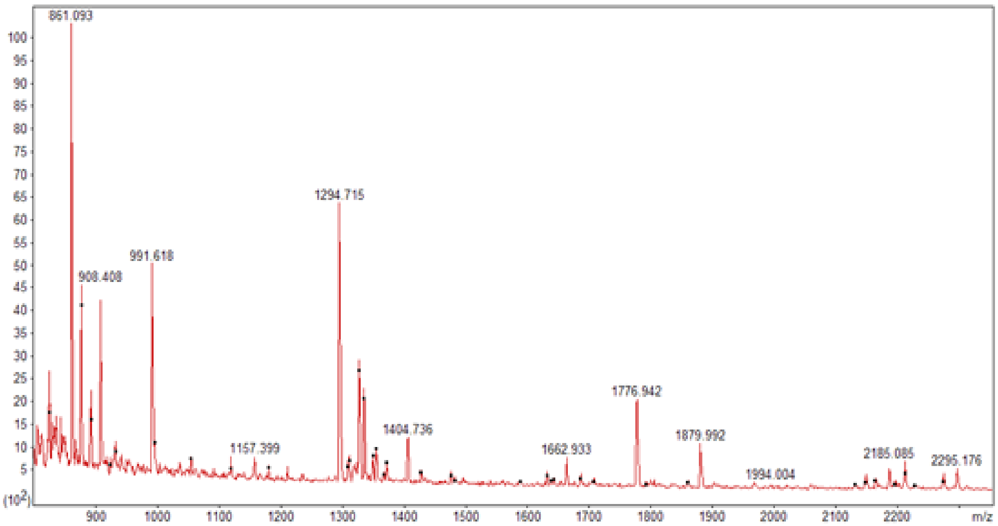

PFF Searched Score:

Ions score is -10*Log(P), where P is the probability that the observed match is a random event.
Individual ions scores > 33 indicate identity or extensive homology (p<0.05).
Protein scores are derived from ions scores as a non-probabilistic basis for ranking protein hits.

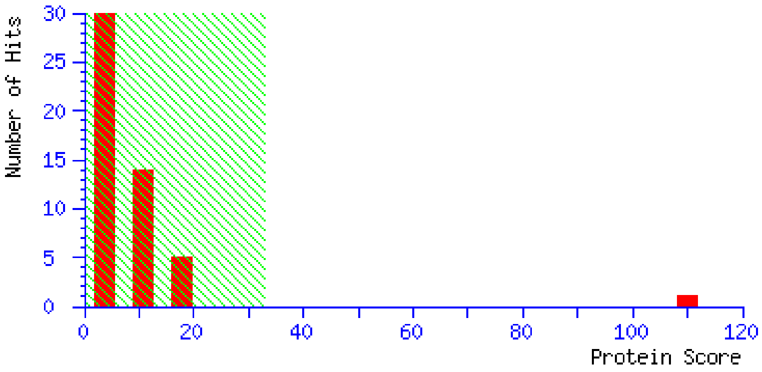

Spot No.: **152**

NCBI accession No.: **cassava4.1_025676m|PACid:17984832**

Plant species: ***Manihot esculenta***

Protein name：**Similar to late embryogenesis abundant proteins**

PFF Mascot score: **[125]** Sequence coverage %: **[14]**

Matched peptides No.: **[3]** p value: **7.1e-006**

Calculated Mr: **34906** Calculated *p*I: **4.75**

Annotated PMF **spectra:**

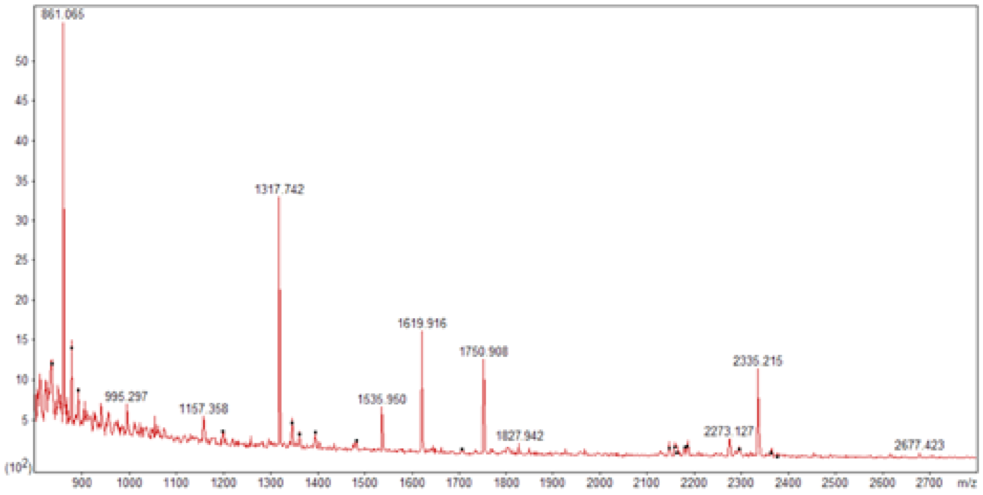

PFF Searched Score:

Ions score is -10*Log(P), where P is the probability that the observed match is a random event
Individual ions scores > 33 indicate identity or extensive homology (p<0.05).
Protein scores are derived from ions scores as a non-probabilistic basis for ranking protein hits.

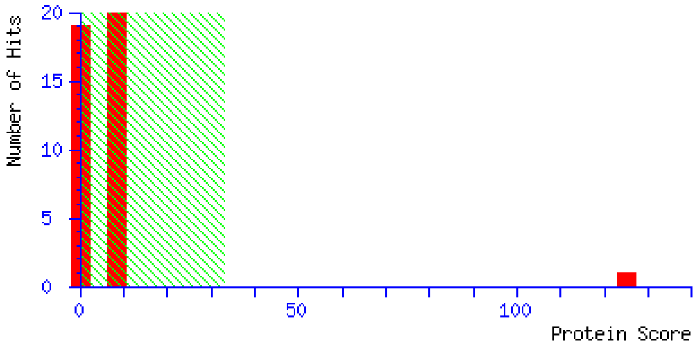

Spot No.: **153**

NCBI accession No.: **cassava4.1_009779m|PACid:17990964**

Plant species: ***Manihot esculenta***

Protein name： **Actin-7**

PFF Mascot score: **[62]**　　Sequence coverage %: **[7]**

Matched peptides No.: **[3]**　　p value: **0.0079**

Calculated Mr: **41897**　　Calculated *p*I: **5.31**

Annotated PMF **spectra:**

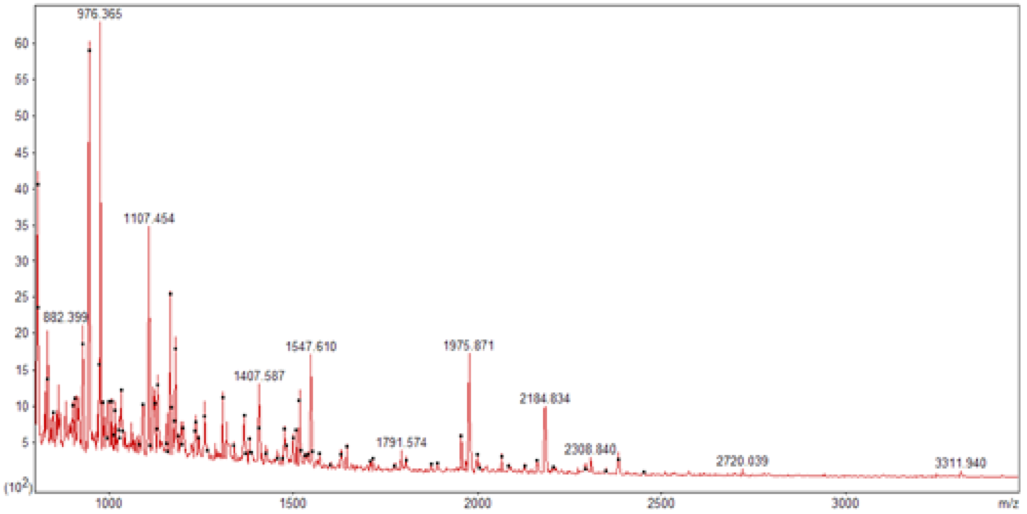

PFF Searched Score:

Ions score is -10*Log(P), where P is the probability that the observed match is a random event.
Individual ions scores > 34 indicate identity or extensive homology (p<0.05).
Protein scores are derived from ions scores as a non-probabilistic basis for ranking protein hits.

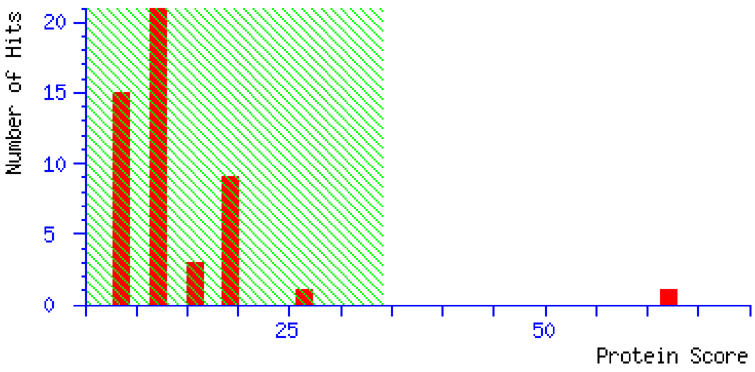

Spot No.: **154**

NCBI accession No.: **cassava4.1_013984m|PACid:17980530**

Plant species: ***Manihot esculenta***

Protein name: **26S proteasome non-ATPase regulatory subunit 8 homolog A**

PFF Mascot score: **[343]** Sequence coverage %: **[37]**

Matched peptides No.: **[7]** p value: **0.00000000028**

Calculated Mr: 30915 Calculated *p*I: 4.93

Annotated PMF **spectra:**

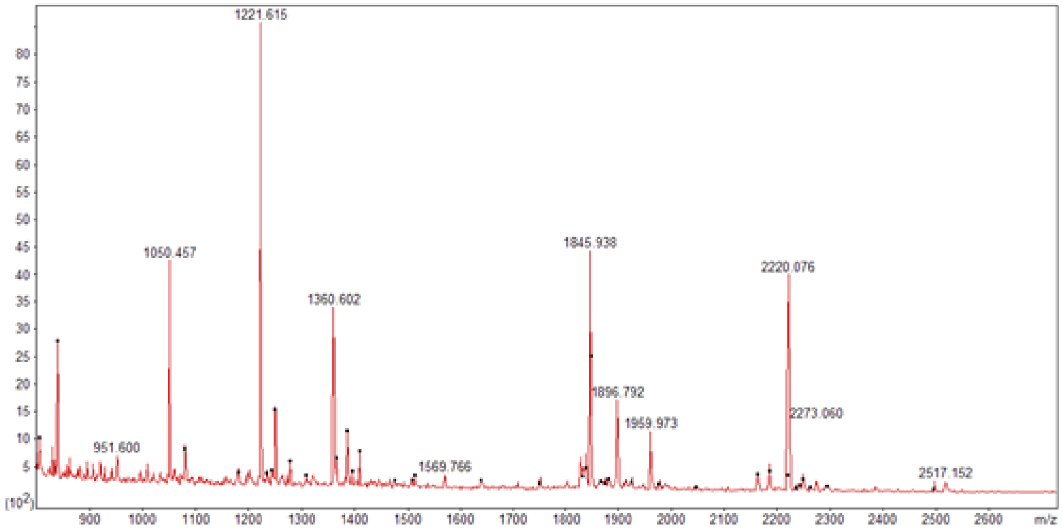

PFF Searched Score:

Ions score is -10*Log(P), where P is the probability that the observed match is a random event.
Individual ions scores > 33 indicate identity or extensive homology (p<0.05).
Protein scores are derived from ions scores as a non-probabilistic basis for ranking protein hits.

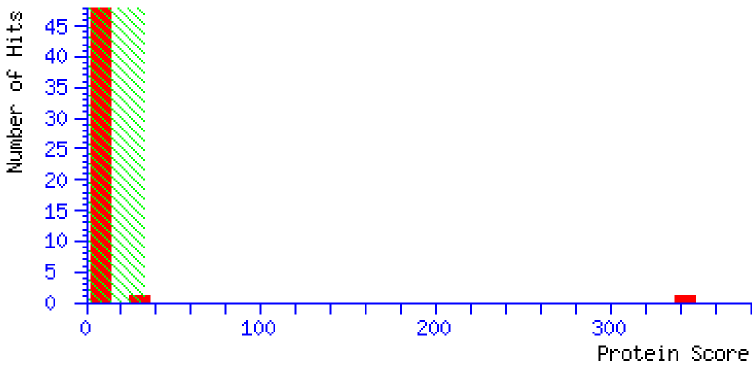

Spot No.: **155**

NCBI accession No.: **cassava4.1_014432m|PACid:17967798**

Plant species: ***Manihot esculenta***

Protein name: **Triosephosphate isomerase, cytosolic**

PFF Mascot score: **[107]** Sequence coverage %: **[10]**

Matched peptides No.: **[2]** p value: **3.9e-007**

Calculated Mr: **27729** Calculated *p*I: **5.77**

Annotated PMF **spectra:**

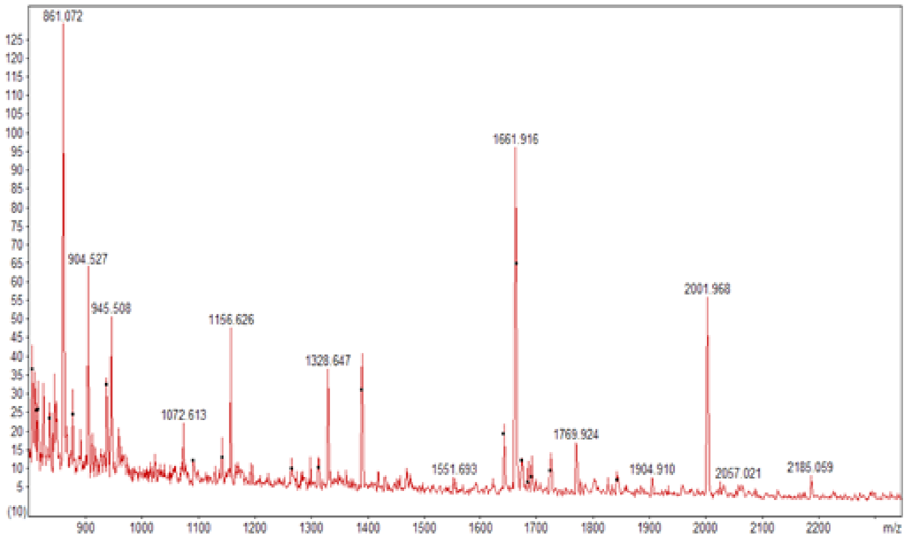

PFF Searched Score:

Ions score is -10*Log(P), where P is the probability that the observed match is a random event.
Individual ions scores > 34 indicate identity or extensive homology (p<0.05).
Protein scores are derived from ions scores as a non-probabilistic basis for ranking protein hits.

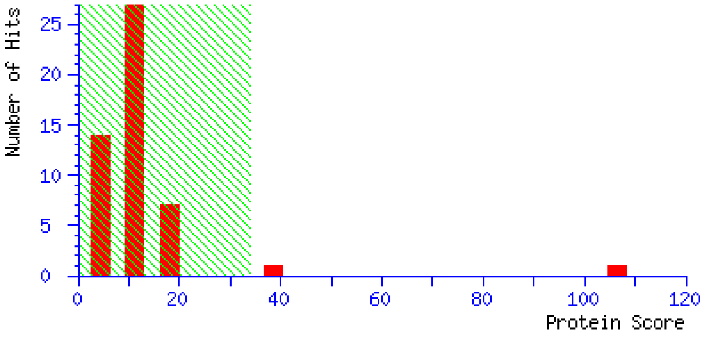

Spot No.: **156**

NCBI accession No.: **cassava4.1_015127m|PACid:17970169**

Plant species: ***Manihot esculenta***

Protein name：**Proteasome subunit alpha type-5-A**

PFF Mascot score: **[123]**

Sequence coverage %: **[10]**

Matched peptides No.: **[3]**

p value: **9.4e-005**

Calculated Mr: **26165**

Calculated *p*I: **4.70**

Annotated PMF **spectra:**

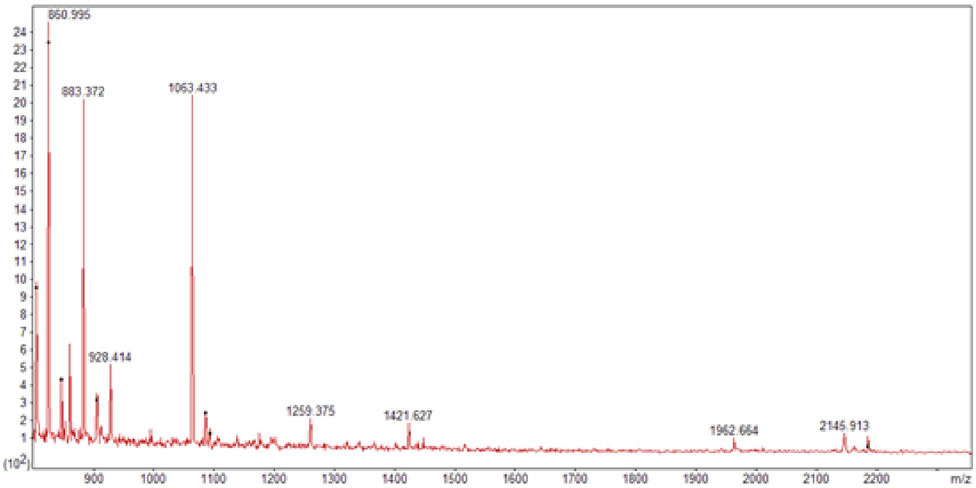

PFF Searched Score:

Ions score is -10*Log(P), where P is the probability that the observed match is a random event.
Individual ions scores > 34 indicate identity or extensive homology (p<0.05).
Protein scores are derived from ions scores as a non-probabilistic basis for ranking protein hits.

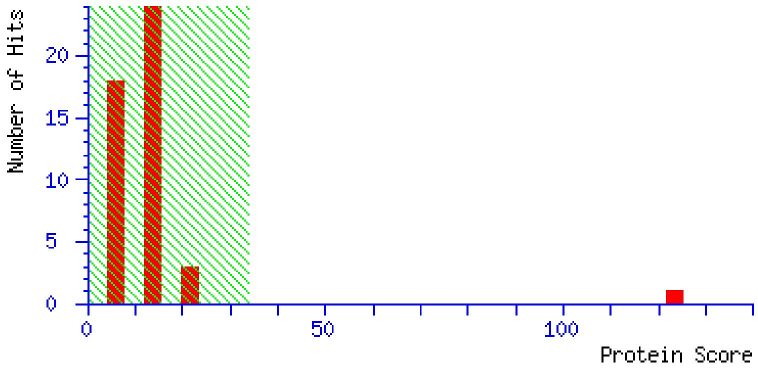

Spot No.: **157**

NCBI accession No.: **cassava4.1_015667m|PACid:17963200**

Plant species: ***Manihot esculenta***

Protein name： **Proteasome subunit beta type-1**

PFF Mascot score: **[61]** Sequence coverage %: **[8]**

Matched peptides No.: **[2]** p value: **7.2e-005**

Calculated Mr: **24884** Calculated *p*I: **6.42**

Annotated PMF **spectra:**

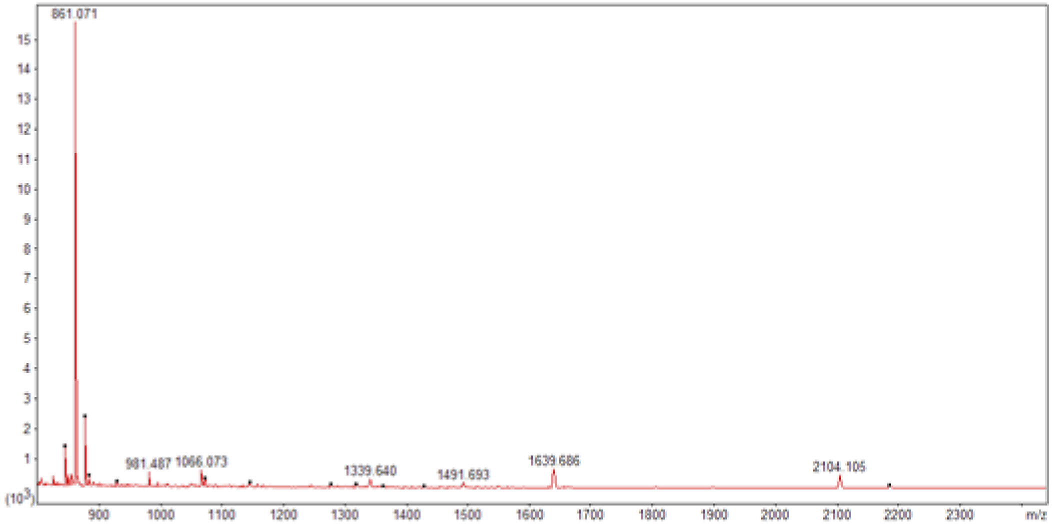

PFF Searched Score:

Ions score is -10*Log(P), where P is the probability that the observed match is a random event.
Individual ions scores > 34 indicate identity or extensive homology (p<0.05).
Protein scores are derived from ions scores as a non-probabilistic basis for ranking protein hits.

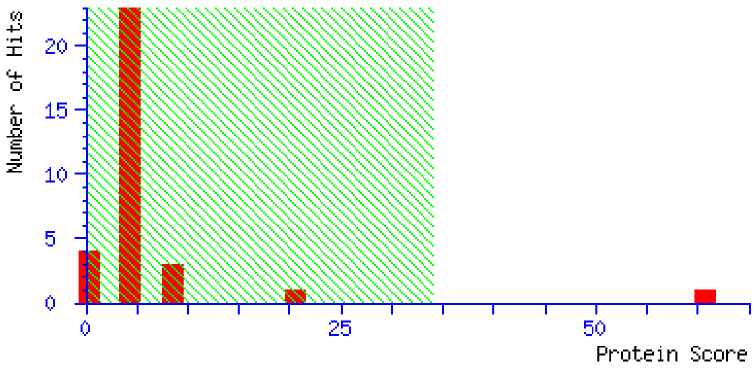

Spot No.: **158**

NCBI accession No.: **cassava4.1_014674m|PACid:17976440**

Plant species: ***Manihot esculenta***

Protein name: **3-isopropylmalate dehydratase small subunit 3**

PFF Mascot score: **[292]** Sequence coverage %: **[26]**

Matched peptides No.: **[4]** p value: **9.3e-007**

Calculated Mr: **26948** Calculated *p*I: **6.52**

Annotated PMF **spectra:**

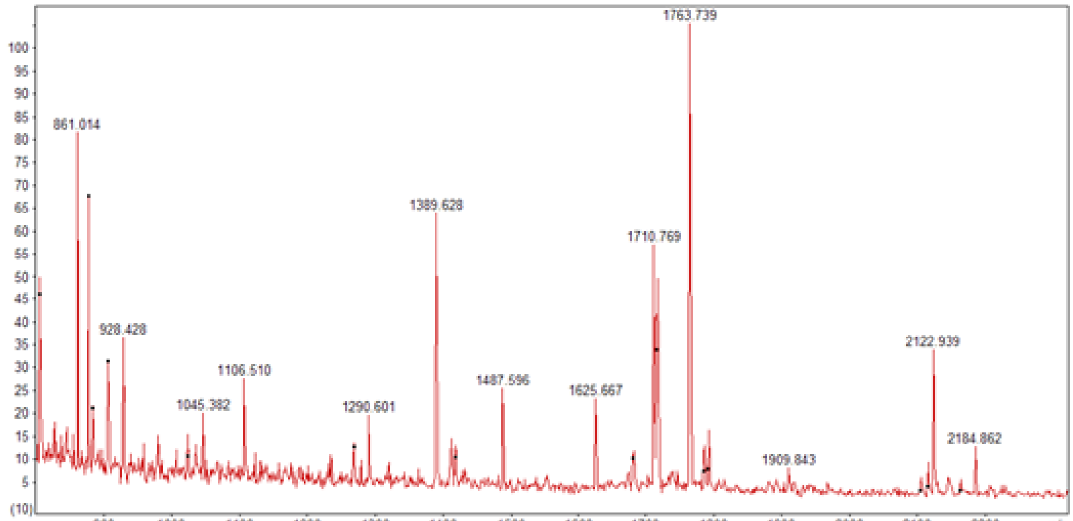

PFF Searched Score:

Ions score is -10*Log(P), where P is the probability that the observed match is a random event.
Individual ions scores > 33 indicate identity or extensive homology (p<0.05).
Protein scores are derived from ions scores as a non-probabilistic basis for ranking protein hits.

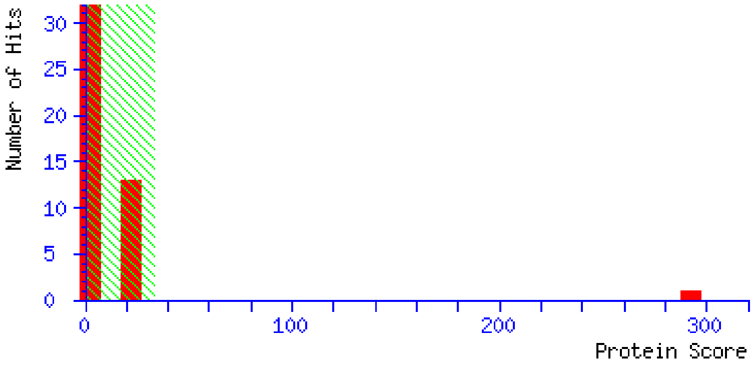

Spot No.: **159**

NCBI accession No.: **cassava4.1_016068m|PACid:17988757**

Plant species: ***Manihot esculenta***

Protein name：**glutathione-s-transferase theta, gst,**

PFF Mascot score: **[192]** Sequence coverage %: **[24]**

Matched peptides No.: **[4]** p value: **1.7e-005**

Calculated Mr: **23848** Calculated *p*I: **6.17**

Annotated PMF **spectra:**

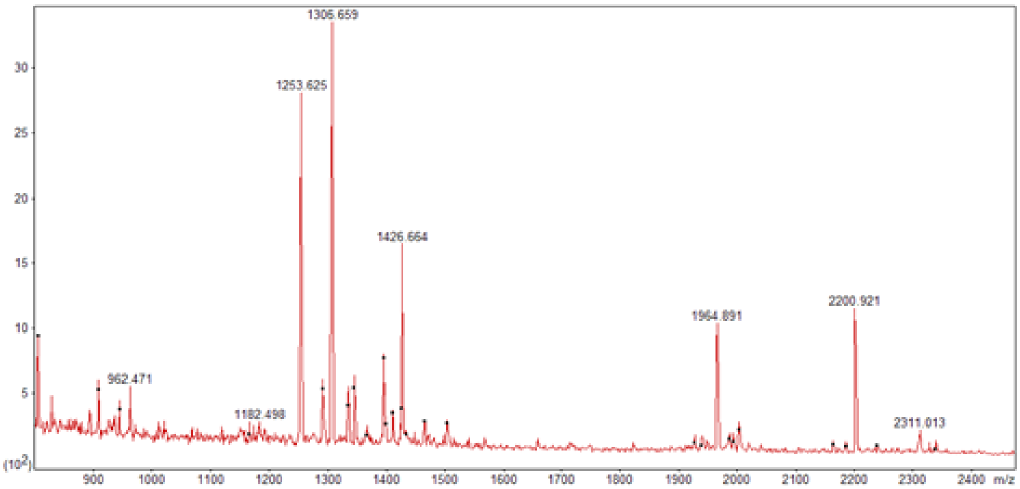

PFF Searched Score:

Ions score is -10*Log(P), where P is the probability that the observed match is a random event.
Individual ions scores > 34 indicate identity or extensive homology (p<0.05).
Protein scores are derived from ions scores as a non-probabilistic basis for ranking protein hits.

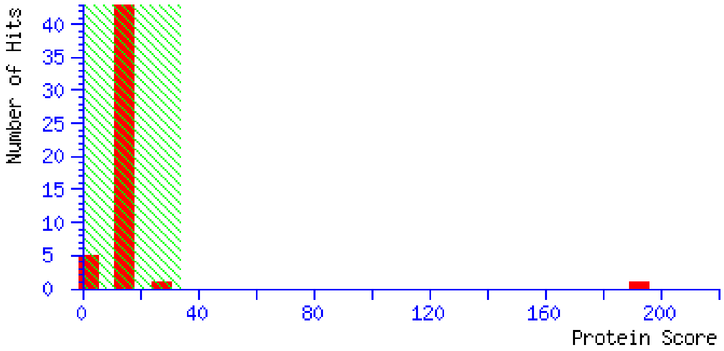

Spot No.: **160**

NCBI accession No.: **cassava4.1_013921m|PACid:17992320**

Plant species: ***Manihot esculenta***

Protein name：**peroxiredoxin**

PFF Mascot score: **[79]** Sequence coverage %: **[9]**

Matched peptides No.: **[2]** p value: **1.4e-006**

Calculated Mr: **29562** Calculated *p*I: **7.66**

Annotated PMF **spectra:**

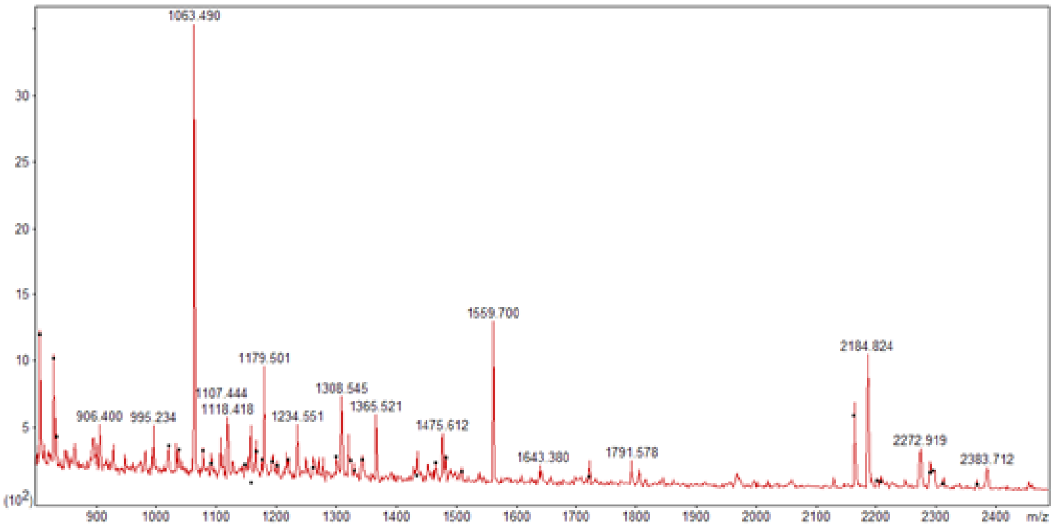

PFF Searched Score:

Ions score is -10*Log(P), where P is the probability that the observed match is a random event.
Individual ions scores > 33 indicate identity or extensive homology (p<0.05).
Protein scores are derived from ions scores as a non-probabilistic basis for ranking protein hits.

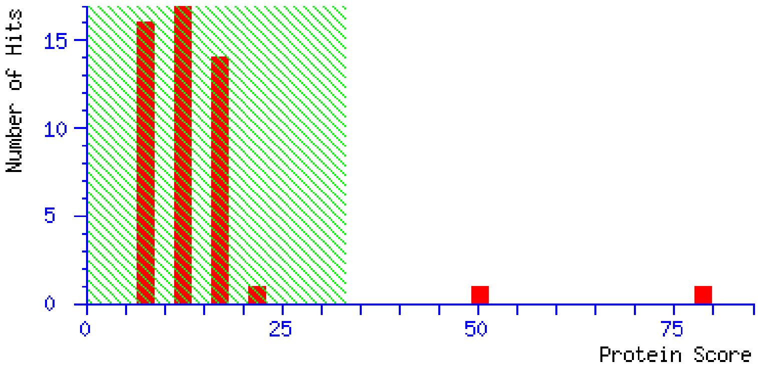

Spot No.: **161**

NCBI accession No.: **cassava4.1_015116m|PACid:17987913**

Plant species: ***Manihot esculenta***

Protein name: **conserved hypothetical protein**

PFF Mascot score: **[173]** Sequence coverage %: **[13]**

Matched peptides No.: **[2]** p value: **2.7e-008**

Calculated Mr: **26233** Calculated *p*I: **5.98**

Annotated PMF **spectra:**

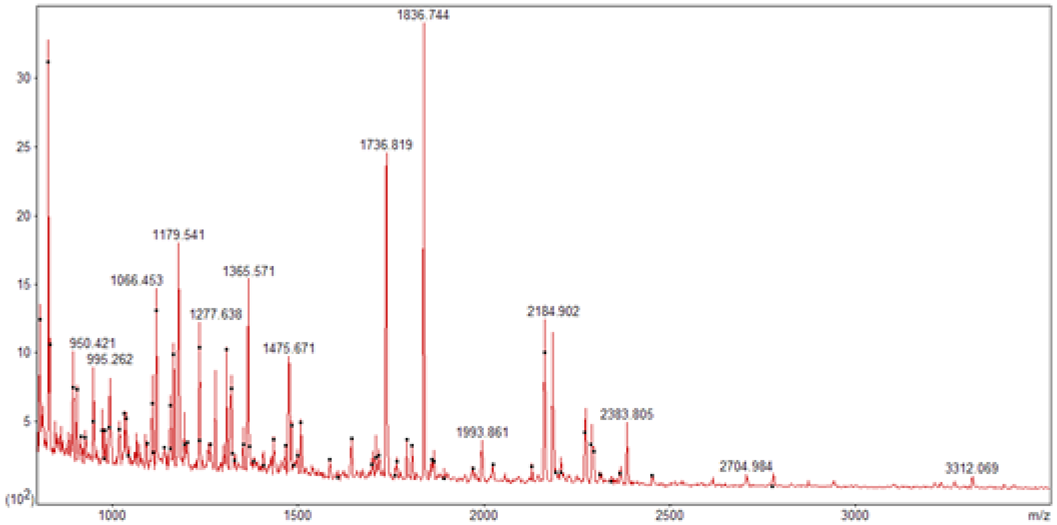

PFF Searched Score:

Ions score is -10*Log(P), where P is the probability that the observed match is a random event.
Individual ions scores > 34 indicate identity or extensive homology (p<0.05).
Protein scores are derived from ions scores as a non-probabilistic basis for ranking protein hits.

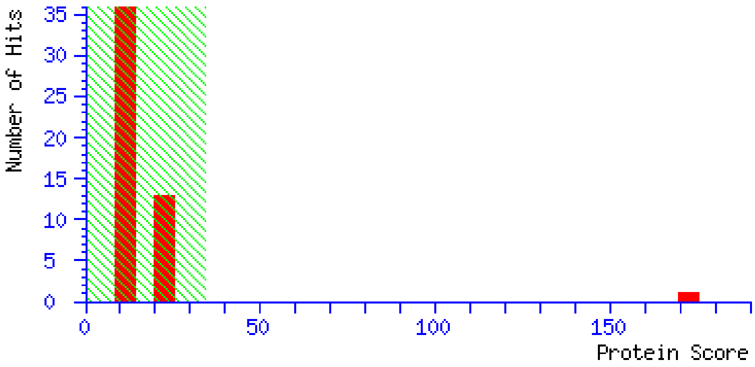

Spot No.: **162**

NCBI accession No.: **cassava4.1_019468m|PACid:17983825**

Plant species: ***Manihot esculenta***

Protein name：**Small ubiquitin-related modifier**

PFF Mascot score: **[88]** Sequence coverage %: **[22]**

Matched peptides No.: **[2]** p value: **0.00045**

Calculated Mr: **12426** Calculated *p*I: **4.95**

Annotated PMF **spectra:**

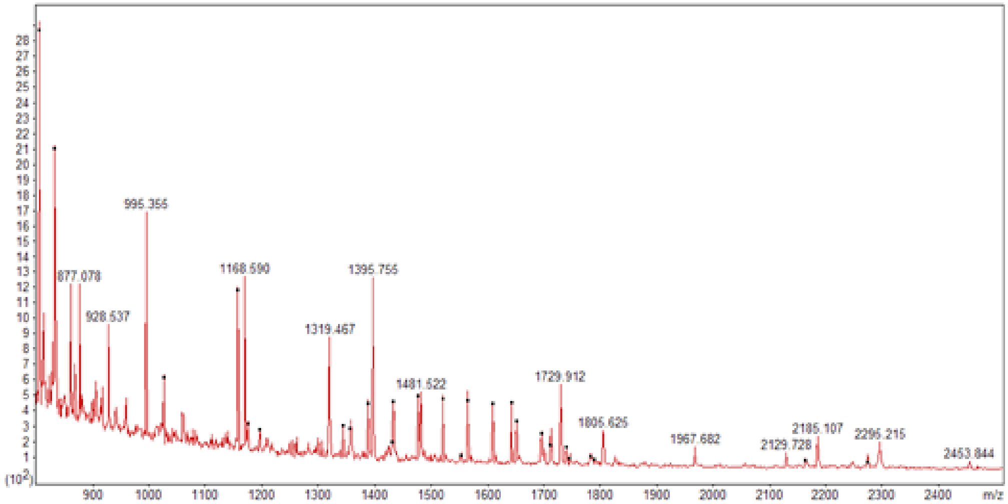

PFF Searched Score:

Ions score is -10*Log(P), where P is the probability that the observed match is a random event.
Individual ions scores > 33 indicate identity or extensive homology (p<0.05).
Protein scores are derived from ions scores as a non-probabilistic basis for ranking protein hits.

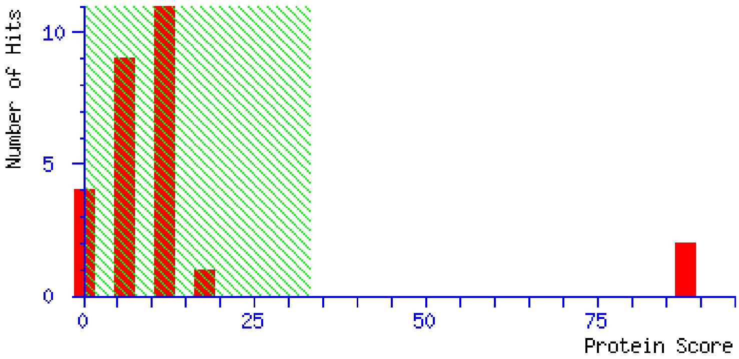

Spot No.: **163**

NCBI accession No.: **cassava4.1_009782m|PACid:17988600**

Plant species: ***Manihot esculenta***

Protein name： **actin**

PFF Mascot score: **78**   Sequence coverage %: **31**

Matched peptides No.: **8**   p value: **0.0026**

Calculated Mr: **32104**   Calculated *p*I: **4.92**

Annotated PMF **spectra:**

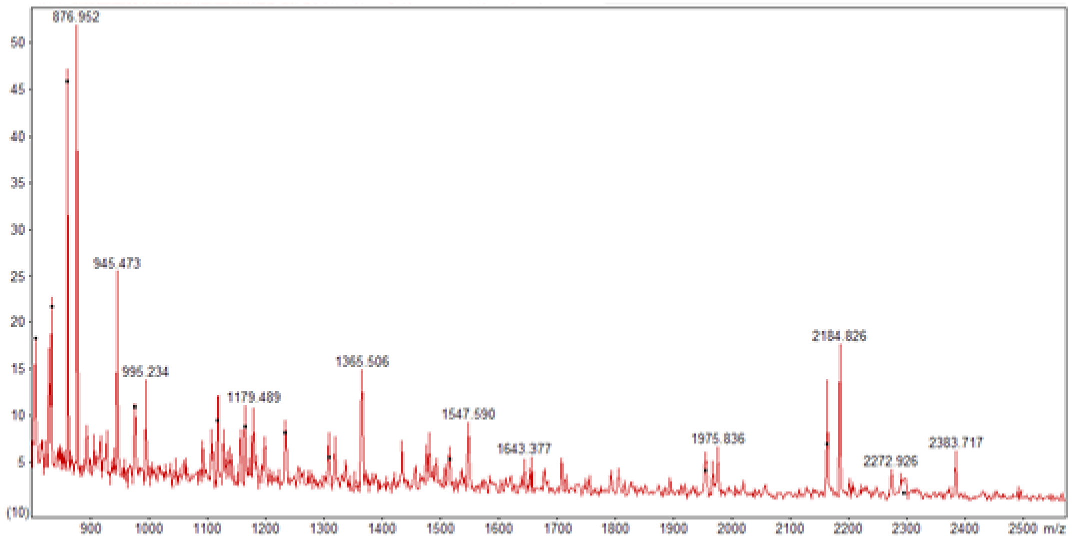

PFF Searched Score:

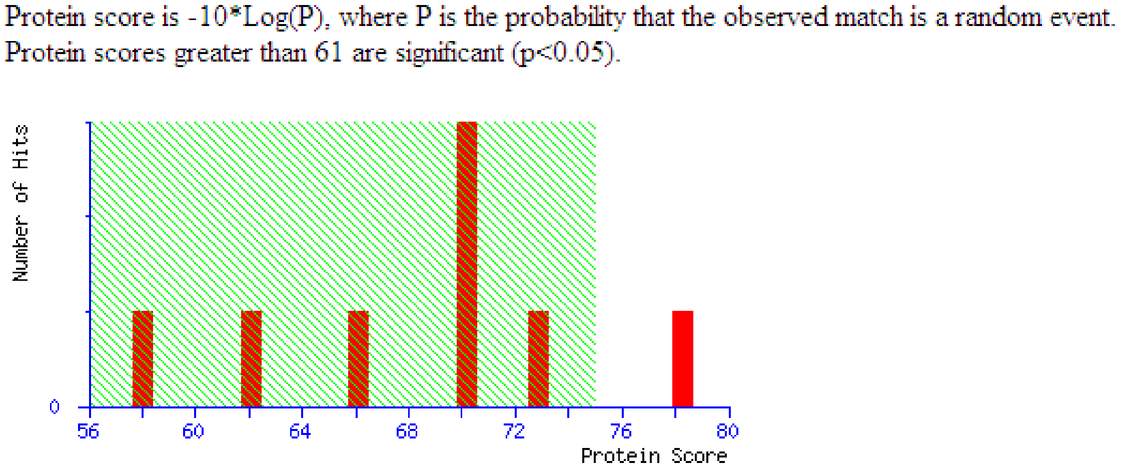

Spot No.: **164**

NCBI accession No.: **cassava4.1_018266m|PACid:17987264**

Plant species: ***Manihot esculenta***

Protein name：**Ubiquitin-conjugating enzyme E2 35**

PFF Mascot score: **[88]** Sequence coverage %: **[26]**

Matched peptides No.: **[5]** p value: **0.00011**

Calculated Mr: **17266** Calculated *p*I: **6.74**

Annotated PMF **spectra:**

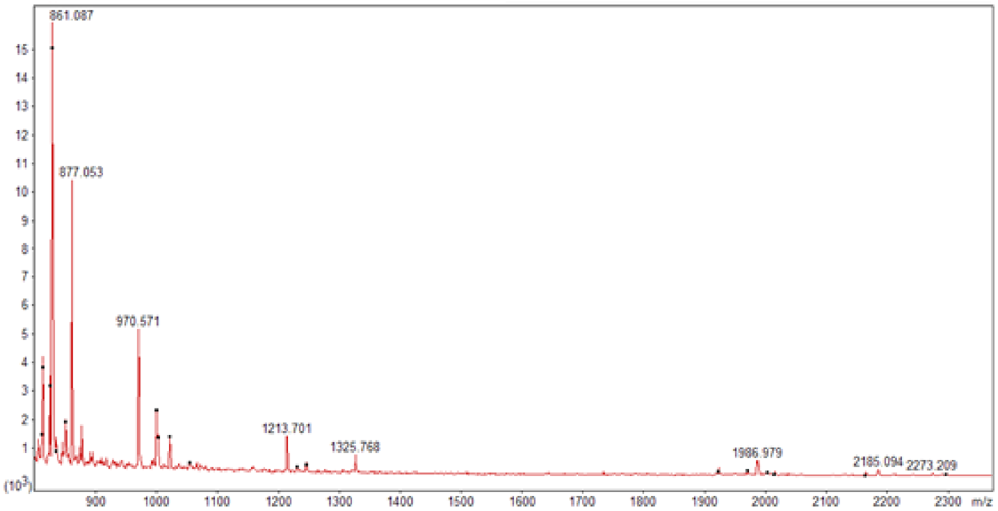

PFF Searched Score:

Ions score is -10*Log(P), where P is the probability that the observed match is a random event.
Individual ions scores > 33 indicate identity or extensive homology (p<0.05).
Protein scores are derived from ions scores as a non-probabilistic basis for ranking protein hits.

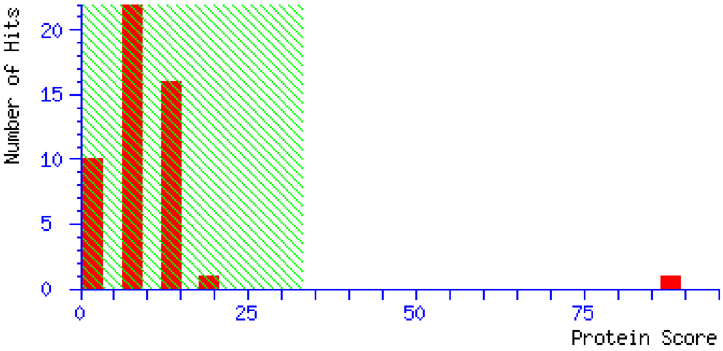

Spot No.: **165**

NCBI accession No.: **cassava4.1_018546m|PACid:17976766**

Plant species: ***Manihot esculenta***

Protein name：**Putative uncharacterized protein At3g56490**

PFF Mascot score: **[153]** Sequence coverage %: **[17]**

Matched peptides No.: **[2]** p value: **1.3e-007**

Calculated Mr: **15944** Calculated *p*I: **6.36**

Annotated PMF **spectra:**

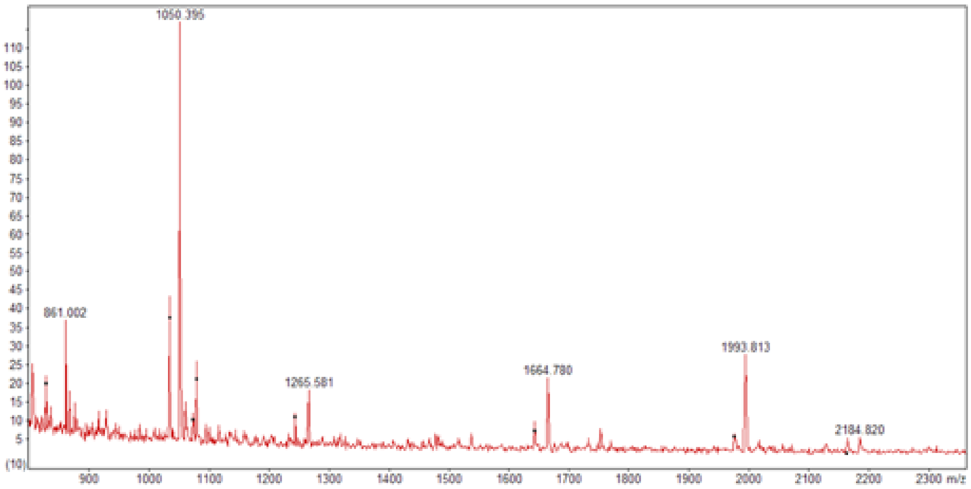

PFF Searched Score:

Ions score is -10*Log(P), where P is the probability that the observed match is a random event.
Individual ions scores > 34 indicate identity or extensive homology (p<0.05).
Protein scores are derived from ions scores as a non-probabilistic basis for ranking protein hits.

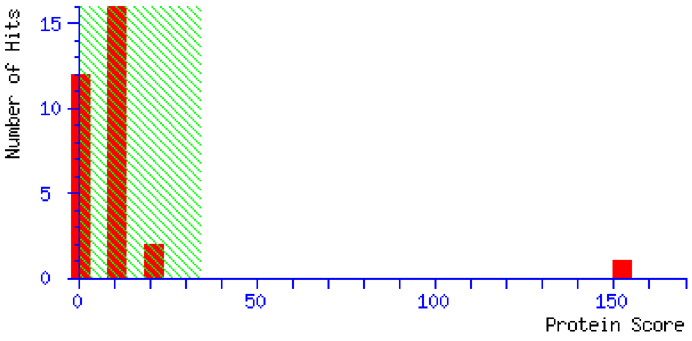

Supplemental Figure S4: Main pathway ways overexpressed in all the differential proteins.

Enzymes exhibiting differential expression during tubernization are marked in green and red color. Marked in light green are enzymes that changed to some extent exhibit. Marked in red are the dramatically up-regulated enzymes. Marked in white are the not detected enzymes or general enzyme categories. Enzyme annotation was obtained from the protein sequence annotation and GO classification data.

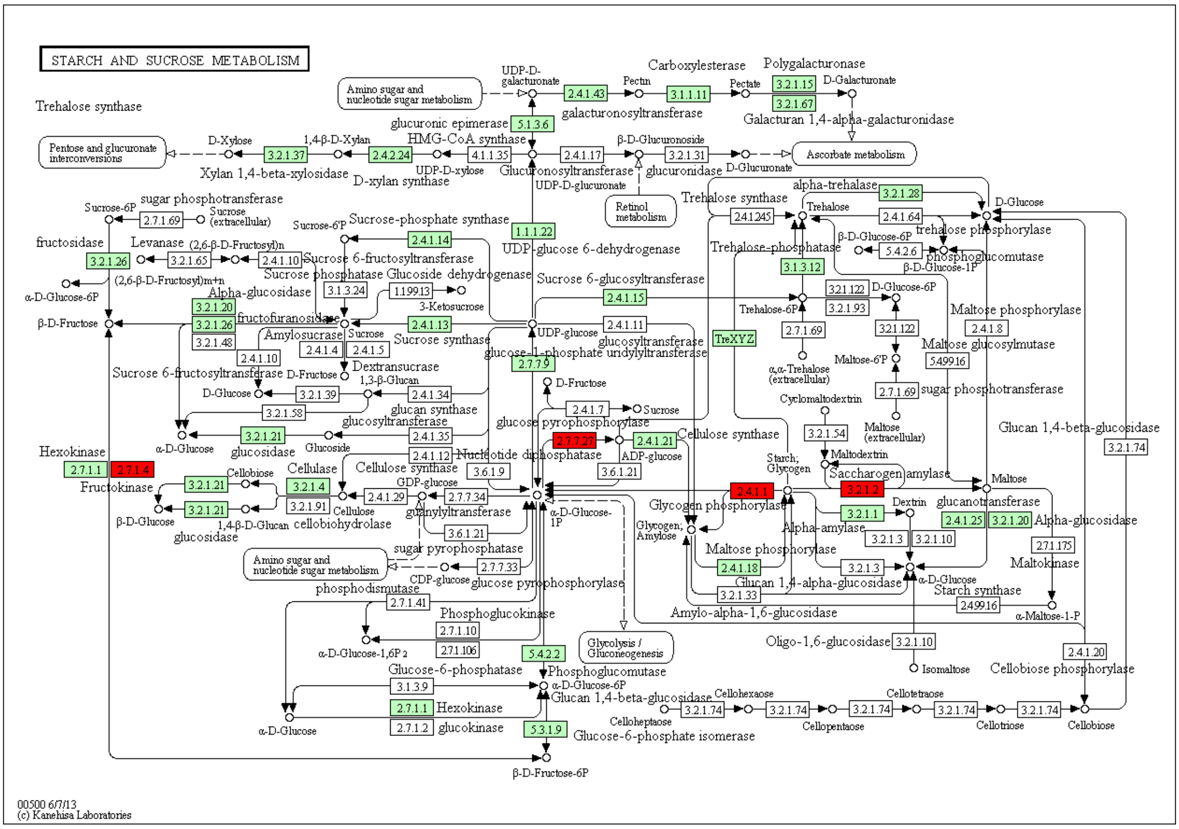

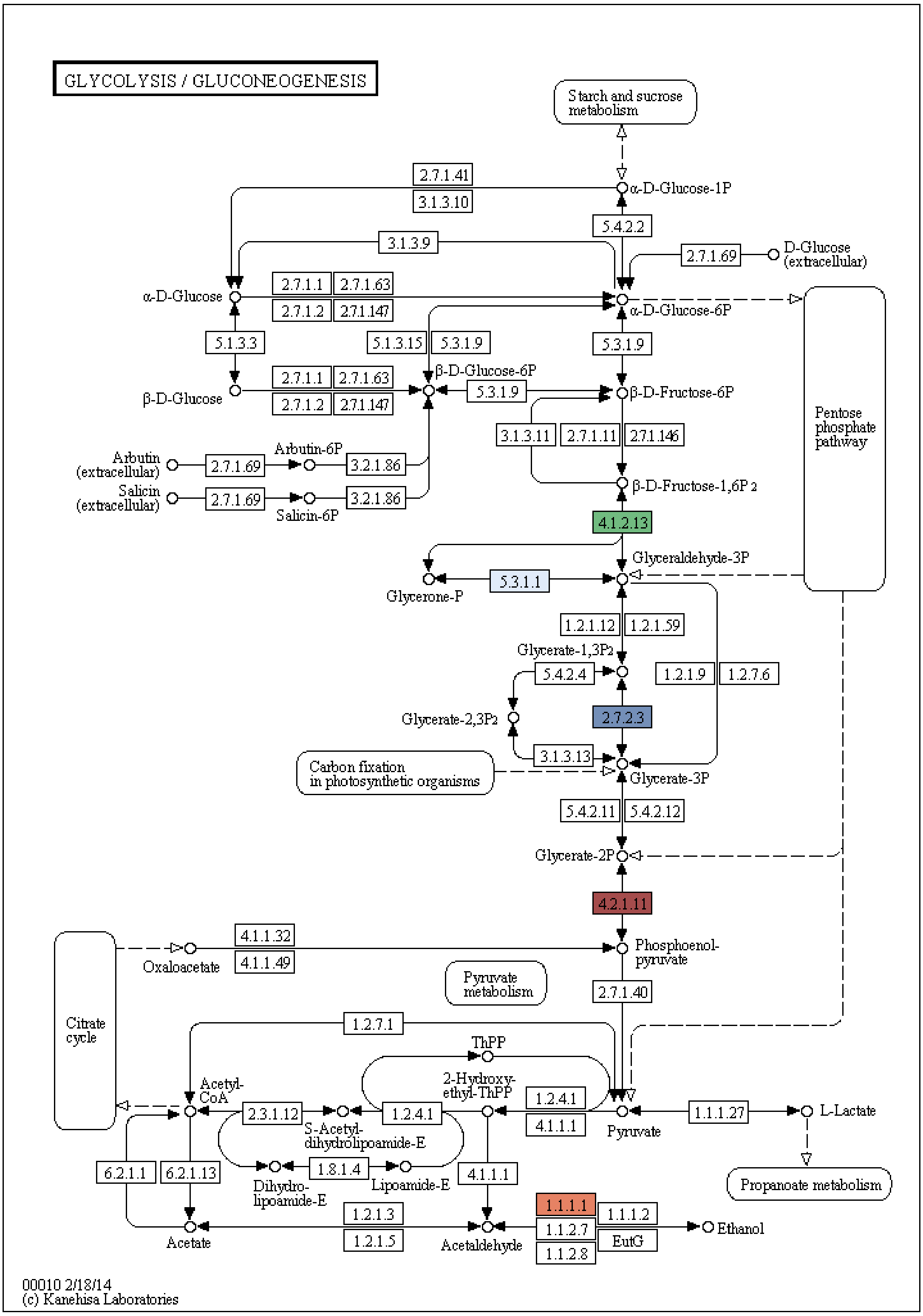
GLYCOLYSIS / GLUCONEOGENESIS
Starch and sucrose metabolism
α-D-Glucose-1P
2.7.1.41
3.1.3.10
5.4.2.2
3.1.3.9
2.7.1.69
D-Glucose (extracellular)
α-D-Glucose
2.7.1.1
2.7.1.63
2.7.1.2
2.7.1.147
α-D-Glucose-6P
5.1.3.3
5.1.3.15
5.3.1.9
β-D-Glucose-6P
β-D-Glucose
β-D-Fructose-6P
Pentose phosphate pathway
3.1.3.11
2.7.1.11
2.7.1.146
Arbutin (extracellular)
Arbutin-6P
3.2.1.86
Salicin (extracellular)
Salicin-6P
β-D-Fructose-1,6P2
4.1.2.13
Glyceraldehyde-3P
Glycerone-P
5.3.1.1
1.2.1.12
1.2.1.59
Glycerate-1,3P2
5.4.2.4
1.2.1.9
1.2.7.6
Glycerate-2,3P2
2.7.2.3
3.1.3.13
Carbon fixation in photosynthetic organisms
Glycerate-3P
5.4.2.11
5.4.2.12
Glycerate-2P
4.2.1.11
Citrate cycle
Oxaloacetate
4.1.1.32
4.1.1.49
Phosphoenol-pyruvate
Pyruvate metabolism
2.7.1.40
1.2.7.1
ThPP
Acetyl-CoA
2-Hydroxy-ethyl-ThPP
1.2.4.1
4.1.1.1
2.3.1.12
S-Acetyl-dihydrolipoamide-E
Pyruvate
1.1.1.27
L-Lactate
6.2.1.1
6.2.1.13
1.8.1.4
Dihydro-lipoamide-E
Lipoamide-E
Propanoate metabolism
1.2.1.3
1.2.1.5
1.1.1.1
1.1.1.2
1.1.2.7
EutG
1.1.2.8
Acetate
Acetaldehyde
Ethanol
00010 2/18/14
(c) Kanehisa Laboratories

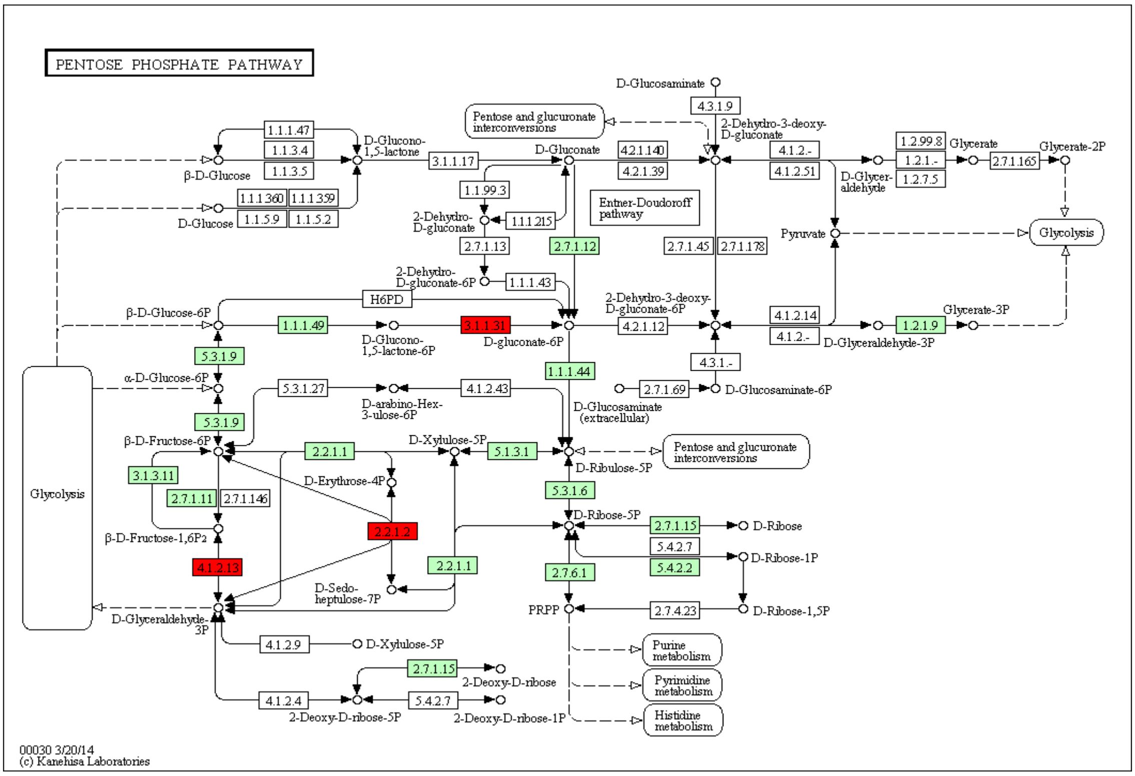
PENTOSE PHOSPHATE PATHWAY
D-Glucosaminate
4.3.1.9
Pentose and glucuronate interconversions
2-Dehydro-3-deoxy-D-gluconate
1.1.1.47
1.1.3.4
1.1.3.5
D-Glucono-1,5-lactone
β-D-Glucose
3.1.1.17
D-Gluconate
4.2.1.140
4.2.1.39
4.1.2.-
4.1.2.51
1.2.99.8
1.2.1.-
1.2.7.5
Glycerate
Glycerate-2P
2.7.1.165
D-Glyceraldehyde
1.1.1.360
1.1.1.359
1.1.5.9
1.1.5.2
D-Glucose
1.1.99.3
2-Dehydro-D-gluconate
1.1.1.215
Entner-Doudoroff pathway
Pyruvate
Glycolysis
2.7.1.13
2.7.1.12
2.7.1.45
2.7.1.178
2-Dehydro-D-gluconate-6P
1.1.1.43
H6PD
β-D-Glucose-6P
1.1.1.49
3.1.1.31
2-Dehydro-3-deoxy-D-gluconate-6P
4.1.2.14
4.1.2.-
Glycerate-3P
1.2.1.9
D-Glucono-1,5-lactone-6P
D-gluconate-6P
4.2.1.12
D-Glyceraldehyde-3P
5.3.1.9
1.1.1.44
4.3.1.-
α-D-Glucose-6P
5.3.1.27
4.1.2.43
2.7.1.69
D-Glucosamine-6P
D-arabino-Hex-3-ulose-6P
D-Glucosamine (extracellular)
5.3.1.9
β-D-Fructose-6P
2.2.1.1
D-Xylulose-5P
5.1.3.1
D-Ribulose-5P
Pentose and glucuronate interconversions
3.1.3.11
D-Erythrose-4P
2.7.1.11
2.7.1.146
5.3.1.6
Glycolysis
β-D-Fructose-1,6P2
2.2.1.2
D-Ribose-5P
2.7.1.15
D-Ribose
5.4.2.7
2.2.1.1
5.4.2.2
D-Ribose-1P
4.1.2.13
2.7.6.1
D-Sedo-heptulose-7P
PRPP
2.7.4.23
D-Ribose-1,5P
D-Glyceraldehyde-3P
Purine metabolism
4.1.2.9
D-Xylulose-5P
2.7.1.15
2-Deoxy-D-ribose
Pyrimidine metabolism
4.1.2.4
5.4.2.7
2-Deoxy-D-ribose-5P
2-Deoxy-D-ribose-1P
Histidine metabolism
00030 3/20/14
(c) Kanehisa Laboratories

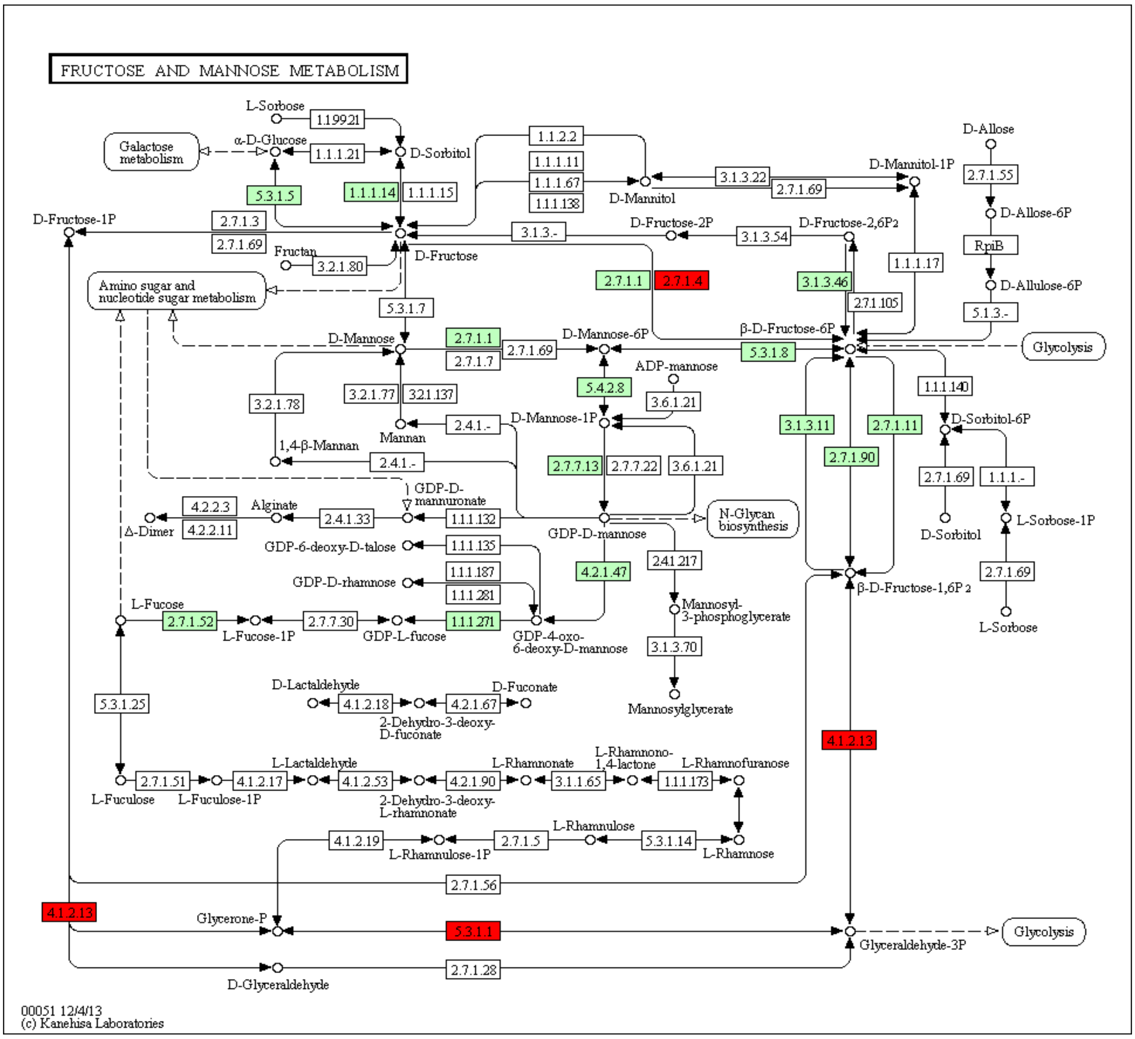
FRUCTOSE AND MANNOSE METABOLISM
L-Sorbose
1.1.99.21
α-D-Glucose
Galactose metabolism
1.1.1.21
D-Sorbitol
1.1.2.2
D-Allose
1.1.1.11
1.1.1.67
1.1.1.138
D-Mannitol
3.1.3.22
2.7.1.69
D-Mannitol-1P
2.7.1.55
D-Allose-6P
5.3.1.5
1.1.1.14
1.1.1.15
D-Fructose-1P
2.7.1.3
2.7.1.69
3.1.3.-
D-Fructose-2P
3.1.3.54
D-Fructose-2,6P2
RpiB
Fructan
3.2.1.80
D-Fructose
1.1.1.17
D-Allulose-6P
Amino sugar and nucleotide sugar metabolism
2.7.1.1
2.7.1.4
3.1.3.46
2.7.1.105
5.1.3.-
5.3.1.7
D-Mannose
2.7.1.1
2.7.1.7
2.7.1.69
D-Mannose-6P
5.3.1.8
β-D-Fructose-6P
Glycolysis
ADP-mannose
5.4.2.8
1.1.1.140
3.2.1.78
3.2.1.77
3.2.1.137
3.6.1.21
Mannan
2.4.1.-
D-Mannose-1P
3.1.3.11
2.7.1.11
D-Sorbitol-6P
1,4-β-Mannan
2.4.1.-
2.7.7.13
2.7.7.22
3.6.1.21
2.7.1.90
2.7.1.69
1.1.1.-
GDP-D-mannuronate
4.2.2.3
Alginate
2.4.1.33
1.1.1.132
N-Glycan biosynthesis
L-Sorbose-1P
Δ-Dimer
4.2.2.11
GDP-D-mannose
D-Sorbitol
GDP-6-deoxy-D-talose
1.1.1.135
2.4.1.217
1.1.1.187
4.2.1.47
β-D-Fructose-1,6P2
2.7.1.69
GDP-D-rhamnose
1.1.1.281
Mannosyl-3-phosphoglycerate
L-Sorbose
L-Fucose
2.7.1.52
2.7.7.30
1.1.1.271
L-Fucose-1P
GDP-L-fucose
GDP-4-oxo-6-deoxy-D-mannose
3.1.3.70
D-Lactaldehyde
D-Fuconate
5.3.1.25
4.1.2.18
4.2.1.67
Mannosylglycerate
2-Dehydro-3-deoxy-D-fuconate
4.1.2.13
L-Lactaldehyde
L-Rhamnonate
L-Rhamnono-1,4-lactone
L-Rhamnofuranose
2.7.1.51
4.1.2.17
4.1.2.53
4.2.1.90
3.1.1.65
1.1.1.173
L-Fuculose
L-Fuculose-1P
2-Dehydro-3-deoxy-L-rhamnonate
L-Rhamnulose
4.1.2.19
2.7.1.5
5.3.1.14
L-Rhamnulose-1P
L-Rhamnose
2.7.1.56
4.1.2.13
Glycerone-P
5.3.1.1
Glycolysis
Glyceraldehyde-3P
D-Glyceraldehyde
2.7.1.28
00051 12/4/13
(c) Kanehisa Laboratories

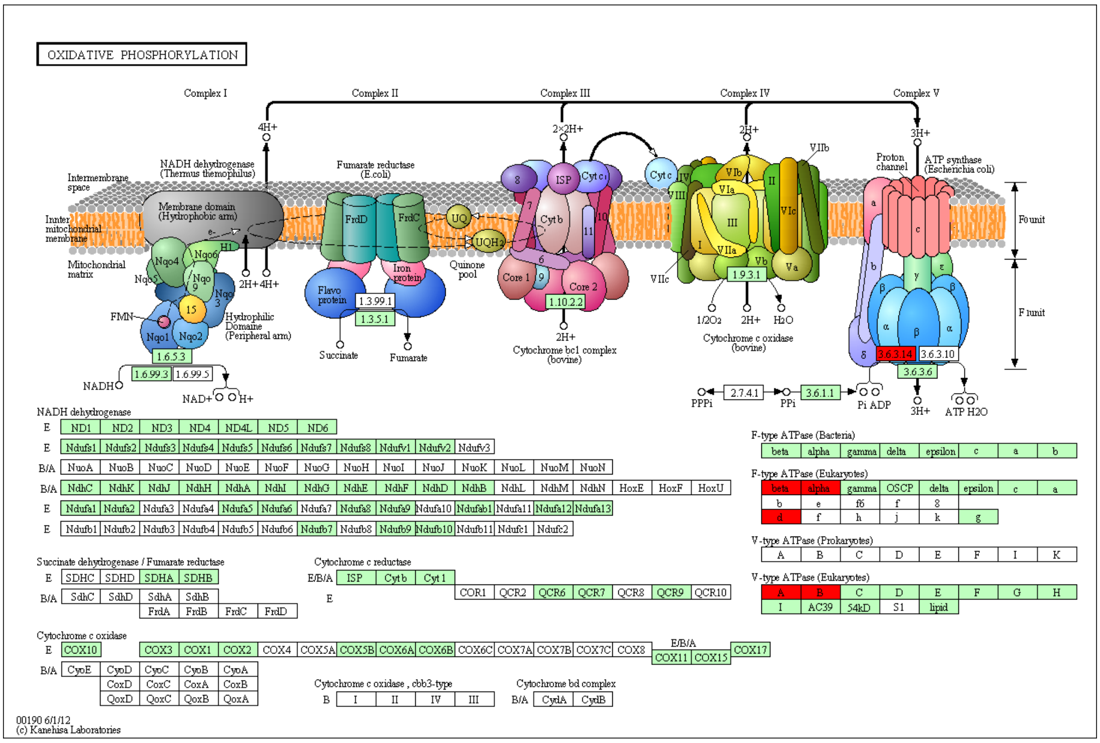
OXIDATIVE PHOSPHORYLATION
Complex I
Complex II
Complex III
Complex IV
Complex V
4H+
2×2H+
2H+
3H+
NADH dehydrogenase (Thermus thermophilus)
Fumarate reductase (E.coli)
ATP synthase (Escherichia coli)
Proton channel
Intermembrane space
Inner mitochondrial membrane
Mitochondrial matrix
Membrane domain (Hydrophobic arm)
Hydrophilic Domain (Peripheral arm)
FMN
NADH
NAD+
H+
Flavo protein
Iron protein
Quinone pool
Succinate
Fumarate
UQ
UQH2
ISP
Cyt c
Cyt b
Core 1
Core 2
Cytochrome bc1 complex (bovine)
Cytochrome c oxidase (bovine)
1/2O2
H2O
F0 unit
F1 unit
1.6.5.3
1.6.99.3
1.6.99.5
1.3.99.1
1.3.5.1
1.10.2.2
1.9.3.1
3.6.3.14
3.6.3.10
3.6.3.6
2.7.4.1
3.6.1.1
PPPi
PPi
Pi ADP
ATP H2O
NADH dehydrogenase
E ND1 ND2 ND3 ND4 ND4L ND5 ND6
E Ndufs1 Ndufs2 Ndufs3 Ndufs4 Ndufs5 Ndufs6 Ndufs7 Ndufs8 Ndufv1 Ndufv2 Ndufv3
B/A NuoA NuoB NuoC NuoD NuoE NuoF NuoG NuoH NuoI NuoJ NuoK NuoL NuoM NuoN
B/A NdhC NdhK NdhJ NdhH NdhA NdhI NdhG NdhE NdhF NdhD NdhB NdhL NdhM NdhN HoxE HoxF HoxU
E Ndufa1 Ndufa2 Ndufa3 Ndufa4 Ndufa5 Ndufa6 Ndufa7 Ndufa8 Ndufa9 Ndufa10 Ndufab1 Ndufa11 Ndufa12 Ndufa13
E Ndufb1 Ndufb2 Ndufb3 Ndufb4 Ndufb5 Ndufb6 Ndufb7 Ndufb8 Ndufb9 Ndufb10 Ndufb11 Ndufc1 Ndufc2
Succinate dehydrogenase / Fumarate reductase
E SDHC SDHD SDHA SDHB
B/A SdhC SdhD SdhA SdhB
FrdA FrdB FrdC FrdD
Cytochrome c reductase
E/B/A ISP Cytb Cyt1
E COR1 QCR2 QCR6 QCR7 QCR8 QCR9 QCR10
Cytochrome c oxidase
E COX10 COX3 COX1 COX2 COX4 COX5A COX5B COX6A COX6B COX6C COX7A COX7B COX7C COX8
E/B/A COX11 COX15 COX17
B/A CyoE CyoD CyoC CyoB CyoA
CoxD CoxC CoxA CoxB
QoxD QoxC QoxB QoxA
Cytochrome c oxidase , cbb3-type
B I II IV III
Cytochrome bd complex
B/A CydA CydB
F-type ATPase (Bacteria)
beta alpha gamma delta epsilon c a b
F-type ATPase (Eukaryotes)
beta alpha gamma OSCP delta epsilon c a
b e f6 f 8
d f h j k g
V-type ATPase (Prokaryotes)
A B C D E F I K
V-type ATPase (Eukaryotes)
A B C D E F G H
I AC39 54kD S1 lipid
00190 6/1/12
(c) Kanehisa Laboratories

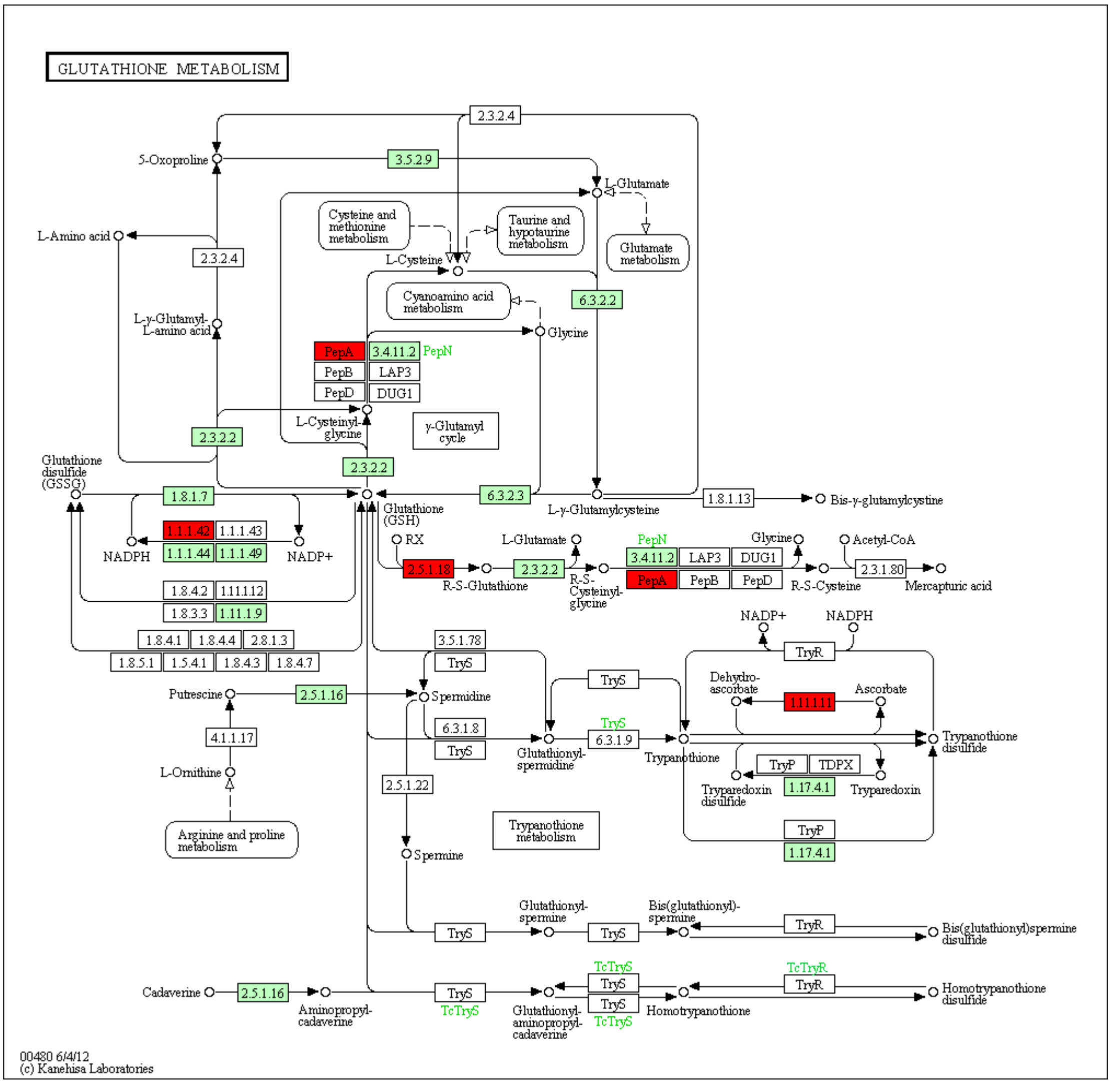
GLUTATHIONE METABOLISM
2.3.2.4
5-Oxoproline
3.5.2.9
L-Glutamate
L-Amino acid
Cysteine and methionine metabolism
Taurine and hypotaurine metabolism
Glutamate metabolism
L-Cysteine
Cyanoamino acid metabolism
6.3.2.2
Glycine
L-γ-Glutamyl-L-amino acid
PepA
3.4.11.2
PepN
PepB
LAP3
PepD
DUG1
L-Cysteinyl-glycine
γ-Glutamyl cycle
2.3.2.2
Glutathione disulfide (GSSG)
1.8.1.7
6.3.2.3
Glutathione (GSH)
L-γ-Glutamylcysteine
1.8.1.13
Bis-γ-glutamylcystine
1.11.1.42
1.11.1.43
1.11.1.44
1.11.1.49
NADPH
NADP+
RX
2.5.1.18
R-S-Glutathione
R-S-Cysteinyl-glycine
Acetyl-CoA
2.3.1.80
R-S-Cysteine
Mercapturic acid
1.8.4.2
1.11.1.12
1.8.3.3
1.11.1.9
1.8.4.1
1.8.4.4
2.8.1.3
1.8.5.1
1.5.4.1
1.8.4.3
1.8.4.7
3.5.1.78
TryS
Putrescine
2.5.1.16
Spermidine
4.1.1.17
6.3.1.8
6.3.1.9
Glutathionyl-spermidine
Trypanothione
Trypanothione disulfide
TryR
Dehydro-ascorbate
Ascorbate
1.11.1.11
TryP
TDPX
1.17.4.1
Tryparedoxin disulfide
Tryparedoxin
L-Ornithine
2.5.1.22
Arginine and proline metabolism
Trypanothione metabolism
Spermine
Glutathionyl-spermine
Bis(glutathionyl)-spermine
Bis(glutathionyl)spermine disulfide
Cadaverine
Aminopropyl-cadaverine
TcTryS
TcTryR
Glutathionyl-aminopropyl-cadaverine
Homotrypanothione
Homotrypanothione disulfide
00480 6/4/12
(c) Kanehisa Laboratories

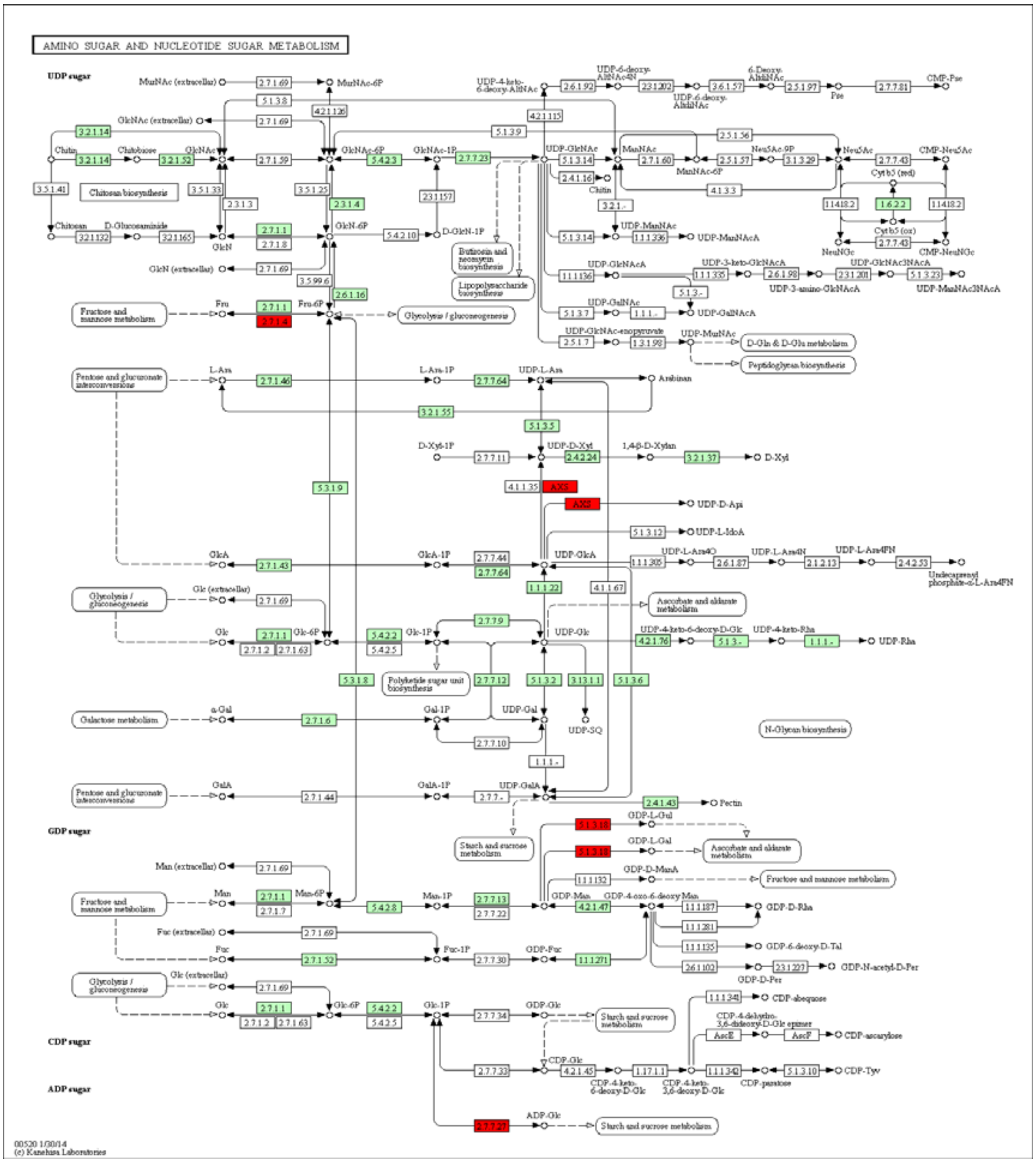
AMINO SUGAR AND NUCLEOTIDE SUGAR METABOLISM
UDP sugar
GDP sugar
CDP sugar
ADP sugar
00520 1/30/14
(c) Kanehisa Laboratories

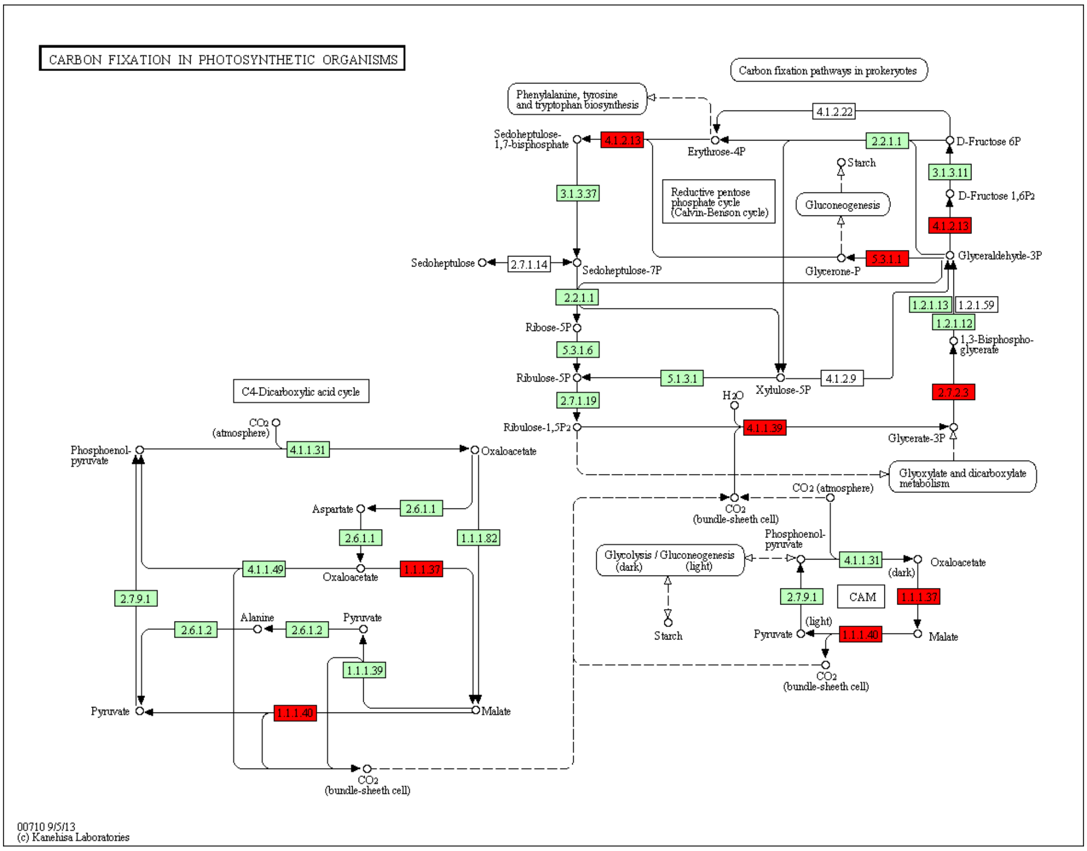
CARBON FIXATION IN PHOTOSYNTHETIC ORGANISMS
Carbon fixation pathways in prokaryotes
Phenylalanine, tyrosine and tryptophan biosynthesis
Sedoheptulose-1,7-bisphosphate
4.1.2.13
Erythrose-4P
4.1.2.22
2.2.1.1
D-Fructose 6P
3.1.3.11
D-Fructose 1,6P2
Starch
Gluconeogenesis
Reductive pentose phosphate cycle (Calvin-Benson cycle)
3.1.3.37
Sedoheptulose
2.7.1.14
Sedoheptulose-7P
5.3.1.1
Glycerone-P
Glyceraldehyde-3P
1.2.1.13
1.2.1.59
1.2.1.12
1,3-Bisphospho-glycerate
Ribose-5P
5.3.1.6
Ribulose-5P
5.1.3.1
Xylulose-5P
4.1.2.9
2.7.2.3
2.7.1.19
Ribulose-1,5P2
H2O
4.1.1.39
Glycerate-3P
Glyoxylate and dicarboxylate metabolism
C4-Dicarboxylic acid cycle
CO2 (atmosphere)
Phosphoenol-pyruvate
4.1.1.31
Oxaloacetate
Aspartate
2.6.1.1
1.1.1.82
4.1.1.49
1.1.1.37
2.7.9.1
Alanine
2.6.1.2
Pyruvate
1.1.1.39
Malate
1.1.1.40
CO2 (bundle-sheeth cell)
Glycolysis / Gluconeogenesis (dark) (light)
CAM
(dark)
(light)
00710 9/5/13
(c) Kanehisa Laboratories

Supplementary Table S1 Information for differential protein spots on 2-DE gels and their corresponding MS identities

Spot No.[a]	Exper.[b] *p*I/M*r*	M.S.[c] Score	ABV S1[d]	ABV S2	ABV S3	ABV S4	ABV S5	ABV S6	ABV S7	ABV S8	ABV S9	Protein name
1	4.78/49	92	0.04	0.00	0.19	0.12	0.03	0.06	0.05	0.00	0.06	hypothetical protein
2	4.93/61	215	0.06	0.20	0.49	0.38	0.23	0.22	0.16	0.12	0.27	Chaperonin 60 subunit alpha 1
3	4.99/54	97	0.13	0.18	0.35	0.17	0.00	0.16	0.13	0.10	0.22	V-type proton ATPase subunit B1
4	5.14/54	187	0.07	0.10	0.21	0.13	0.16	0.15	0.08	0.00	0.13	V-type proton ATPase subunit B2
5	5.15/51	101	0.00	0.23	0.31	0.24	0.29	0.22	0.10	0.00	0.00	Tubulin alpha-6 chain
6	5.05/47	154	0.08	0.12	0.20	0.13	0.21	0.10	0.12	0.07	0.21	26S protease regulatory subunit 6A
7	5.19/70	115	0.34	0.35	0.55	0.40	0.43	0.35	0.23	0.28	0.43	RNA polymerase II transcription
8	5.21/70	98	0.57	0.52	1.01	0.58	0.68	0.51	0.45	0.35	0.53	RNA polymerase II transcription
9	5.23/70	128	0.11	0.25	0.56	0.51	0.48	0.38	0.36	0.36	0.34	RNA polymerase II transcription
10	5.30/67	127	0.15	0.35	0.27	0.41	0.22	0.18	0.43	0.27	0.21	Actin-7
11	5.24/60	147	0.24	0.30	0.31	0.22	0.22	0.19	0.17	0.16	0.15	Chaperonin 60 subunit beta 2
12	5.27/60	73	0.07	0.18	0.20	0.22	0.19	0.17	0.16	0.13	0.12	Chaperonin 60 subunit beta 1
13	5.28/60	143	0.30	0.19	0.15	0.13	0.13	0.12	0.11	0.11	0.09	Chaperonin CPN60, mitochondrial
14	5.18/53	87	0.04	0.30	0.36	0.30	0.57	0.28	0.24	0.22	0.13	Tubulin alpha-6 chain
15	5.18/51	103	0.08	0.52	0.92	0.59	0.78	0.62	0.38	0.29	0.15	Tubulin alpha-6 chain
16	5.23/53	89	0.05	0.06	0.13	0.23	0.24	0.25	0.19	0.23	0.18	Tubulin alpha-6 chain
17	5.31/51	173	0.19	0.12	0.07	0.37	0.03	0.08	0.05	0.02	0.00	Actin-7
18	5.50/52	190	0.47	0.53	0.33	0.23	0.30	0.26	0.24	0.37	0.15	ATP synthase beta subunit 1
19	5.78/53	175	0.52	0.33	0.38	0.26	0.46	0.60	0.43	0.75	0.34	ATP synthase subunit beta-3
20	5.75/49	182	0.15	0.34	0.15	0.06	0.08	0.13	0.05	0.06	0.00	Glucose adenylyltransferase
21	5.80/42	101	0.55	0.49	0.46	0.24	0.39	0.46	0.35	0.39	0.15	Glutamine synthetase
22	5.20/44	96	0.10	0.24	0.28	0.22	0.39	0.22	0.25	0.09	0.11	Actin-7
23	5.26/44	93	0.14	0.25	0.23	0.30	0.37	0.31	0.22	0.20	0.25	Actin-7
24	5.31/44	195	0.34	1.32	1.15	1.40	1.71	1.33	1.38	1.78	1.32	Actin-7
25	5.28/42	128	0.23	0.26	0.27	0.35	0.31	0.21	0.23	0.12	0.28	Succinyl-CoA ligase
26	5.29/42	107	0.26	0.26	0.39	0.34	0.30	0.16	0.18	0.26	0.17	Glutamine synthetase
27	5.30/40	[113]	0.27	0.35	0.18	0.13	0.16	0.13	0.13	0.12	0.12	Adenosine kinase 1
28	5.01/79	127	0.09	0.36	0.60	0.57	0.47	0.32	0.30	0.24	0.74	Heat shock protein 90-3
29	5.54/96	81	0.00	0.00	0.00	0.03	0.04	0.13	0.10	0.10	0.12	Alpha-glucan phosphorylase 1
30	5.60/96	[387]	0.00	0.03	0.04	0.07	0.08	0.16	0.20	0.12	0.19	Alpha-glucan phosphorylase 1
31	5.60/96	76	0.00	0.03	0.03	0.04	0.04	0.13	0.11	0.10	0.07	Alpha-glucan phosphorylase 1
32	5.59/102	[110]	0.00	0.02	0.00	0.00	0.00	0.00	0.00	0.12	0.00	Alpha-glucan phosphorylase 1
33	5.73/102	101	0.00	0.05	0.00	0.00	0.00	0.00	0.01	0.15	0.02	Alpha-glucan phosphorylase 1
34	4.62/29	115	0.35	0.27	0.55	0.48	0.38	0.18	0.24	0.25	0.97	14-3-3-like protein GF14 omega
35	4.68/28	159	0.57	0.12	1.08	0.79	0.56	0.25	0.32	0.36	0.88	14-3-3-like protein GF14 omega
36	4.75/29	111	0.37	0.11	1.09	0.61	0.72	0.14	0.21	0.25	0.56	14-3-3-like protein GF14 psi
37	5.16/37	[154]	0.24	0.18	0.34	0.19	0.21	0.14	0.13	0.18	0.24	Probable fructokinase-1
38	5.18/36	[147]	0.26	0.21	0.27	0.16	0.17	0.12	0.09	0.10	0.14	Probable fructokinase-1
39	5.21/34	151	0.65	0.73	0.99	0.62	0.90	0.39	0.48	0.39	0.70	Probable lactoylglutathione lyase

**40**	5.24/34	[73]	0.19	0.17	0.29	0.29	0.28	0.12	0.13	0.12	0.16	Probable lactoylglutathione lyase
**41**	5.27/32	[187]	0.14	0.17	0.19	0.19	0.24	0.16	0.19	0.20	0.27	Carboxypho-yruvate mutase
**42**	5.28/36	[146]	0.30	0.30	0.27	0.12	0.19	0.10	0.11	0.13	0.12	Probable fructokinase-1
**43**	5.45/37	[81]	0.44	0.42	0.19	0.39	0.45	0.32	0.35	0.26	0.30	NADP-dependent reductase P1
**44**	5.45/38	[99]	0.32	0.37	0.47	0.41	0.49	0.33	0.46	0.45	0.34	NADP-dependent reductase P1
**45**	5.31/35	[151]	0.13	0.15	0.14	0.14	0.17	0.13	0.15	0.19	0.15	DNA-damage-repair protein
**46**	5.77/36	[254]	0.55	0.24	0.19	0.11	0.14	0.11	0.12	0.13	0.08	NAD(P)H oxidoreductase
**47**	5.38/32	[133]	0.00	0.05	0.06	0.21	0.23	0.17	0.28	0.26	0.39	Thiamine thiazole synthase
**48**	5.18/30	[209]	0.00	0.17	0.29	0.14	0.27	0.11	0.13	0.07	0.14	Alpha-soluble NSF protein 2
**49**	5.21/24	125	0.16	0.14	0.34	0.36	0.42	0.17	0.23	0.20	0.37	Ferritin-4, chloroplastic
**50**	5.28/23	[172]	0.33	0.31	0.49	0.42	0.46	0.17	0.30	0.15	0.41	20 kDa chaperonin, chloroplastic
**51**	5.31/27	82	0.59	0.30	0.29	0.27	0.34	0.34	0.36	0.30	0.56	L-ascorbate peroxidase 1
**52**	5.55/28	133	1.79	1.21	2.47	1.99	3.21	2.10	1.51	2.58	3.37	L-ascorbate peroxidase 1
**53**	5.71/28	[96]	0.66	0.48	0.45	0.31	0.45	0.13	0.17	0.10	0.22	Actin-7
**54**	4.85/21	113	0.45	0.20	0.39	0.21	0.28	0.17	0.21	0.11	0.45	ATP synthase subunit d
**55**	5.15/19	184	0.32	0.18	0.31	0.15	0.25	0.13	0.12	0.08	0.17	ATP synthase subunit d
**56**	5.60/22	[137]	0.38	0.20	0.15	0.10	0.15	0.11	0.10	0.12	0.09	Proteasome subunit beta type
**57**	5.25/17	[133]	0.19	0.31	0.42	0.35	0.42	0.20	0.27	0.21	0.26	regulator of ribonuclease
**58**	5.60/17	[66]	0.00	0.08	0.17	0.52	0.57	0.38	0.47	0.32	0.63	Eukaryotic translation initiation factor
**59**	5.60/17	72	0.00	0.05	0.10	0.25	0.24	0.15	0.19	0.15	0.24	Eukaryotic translation initiation factor
**60**	5.78/16	[196]	0.13	0.18	0.16	0.45	0.35	0.55	0.49	0.48	0.32	heat-shock protein, putative
**61**	5.94/17	[112]	0.13	0.19	0.19	0.16	0.19	0.15	0.16	0.17	0.12	Actin-depolymerizing factor 3
**62**	5.99/18	[95]	0.54	0.98	1.38	0.79	1.71	0.58	0.89	0.62	0.36	class I heat shock protein
**63**	5.98/17	82	0.30	0.40	0.55	0.32	0.72	0.21	0.25	0.25	0.09	Eukaryotic translation initiation factor
**64**	6.17/18	[180]	0.69	1.23	1.26	1.03	1.26	0.60	1.11	0.60	0.54	Peroxiredoxin-2B
**65**	6.39/18	[164]	0.00	1.99	3.73	4.73	4.30	3.85	4.45	3.87	3.40	class I heat shock protein
**66**	6.60/15	[80]	0.45	0.36	0.32	0.16	0.19	0.25	0.32	0.20	0.00	Superoxide dismutase [Cu-Zn] 1
**67**	6.60/16	[147]	0.15	0.45	0.52	0.33	0.32	0.35	0.40	0.36	0.13	Actin-depolymerizing factor 3
**68**	6.84/17	119	0.34	0.41	0.28	0.18	0.20	0.21	0.14	0.22	0.10	Ubiquitin-conjugating enzyme E2
**69**	7.03/16	[195]	0.00	0.56	0.66	0.38	0.68	0.69	0.21	0.57	0.14	AT3g53990/F5K20_290
**70**	6.61/22	139	1.03	0.68	0.45	0.28	0.39	0.38	0.41	0.34	0.17	Superoxide dismutase [Mn] 1
**71**	6.60/23	[99]	0.31	0.13	0.15	0.10	0.15	0.12	0.13	0.17	0.05	Proteasome subunit alpha type-2-A
**72**	6.01/24	[94]	0.29	0.11	0.12	0.12	0.14	0.14	0.11	0.12	0.08	Superoxide dismutase [Fe] 2
**73**	6.55/26	[234]	0.35	0.32	0.26	0.13	0.22	0.21	0.20	0.22	0.20	L-ascorbate peroxidase 1
**74**	6.59/26	[194]	0.45	0.47	0.33	0.21	0.28	0.38	0.39	0.42	0.00	Triosephosphate isomerase
**75**	6.81/28	131	0.29	0.29	0.24	0.15	0.16	0.18	0.11	0.21	0.00	Proteasome subunit alpha type-6-A
**76**	5.86/32	[162]	0.00	0.04	0.09	0.14	0.20	0.21	0.22	0.27	0.16	Thiamine thiazole synthase
**77**	6.22/32	88	0.00	0.02	0.05	0.27	0.26	0.36	0.43	0.70	0.18	Thiamine thiazole synthase
**78**	6.10/48	119	0.26	0.44	0.32	0.14	0.21	0.15	0.11	0.13	0.06	S-adenosylmethionine synthase 1
**79**	6.64/39	108	0.89	0.46	0.28	0.17	0.24	0.34	0.29	0.35	0.10	malate dehydrogenase
**80**	6.44/46	163	0.06	0.09	0.07	0.06	0.06	0.13	0.11	0.16	0.03	UDP-D-apiose/-xylose synthase 1
**81**	6.49/45	115	0.07	0.12	0.08	0.06	0.07	0.12	0.09	0.13	0.04	Cytosolic isocitrate dehydrogenase
**82**	6.58/47	76	0.17	0.27	0.13	0.07	0.10	0.16	0.08	0.10	0.05	GDP-mannose 3,5-epimerase

**83**	6.24/54	124	0.13	0.30	0.31	0.18	0.19	0.26	0.28	0.39	0.11	Bifunctional enolase 2
**84**	6.21/52	100	0.00	0.20	0.21	0.12	0.14	0.22	0.14	0.27	0.13	Bifunctional enolase 2
**85**	6.32/55	151	0.25	0.31	0.09	0.20	0.21	0.32	0.26	0.33	0.10	ATP synthase F1 subunit 1
**86**	6.45/64	177	0.08	0.11	0.10	0.10	0.14	0.18	0.20	0.19	0.08	malic enzyme, putative
**87**	6.43/54	[150]	0.09	0.33	0.29	0.16	0.18	0.40	0.39	0.26	0.14	Bifunctional enolase 2
**88**	6.41/52	160	0.07	0.24	0.20	0.14	0.17	0.28	0.25	0.25	0.10	Bifunctional enolase 2
**89**	5.45/69	234	0.02	0.04	0.04	0.01	0.01	0.04	0.02	0.04	0.01	V-type proton ATPase
**90**	5.55/69	194	0.07	0.07	0.12	0.06	0.06	0.06	0.04	0.11	0.04	V-type proton ATPase
**91**	5.60/49	81	0.02	0.07	0.15	0.06	0.06	0.06	0.08	0.86	0.06	Glucose-adenylyltransferase
**92**	6.61/64	154	0.04	0.11	0.03	0.06	0.03	0.03	0.05	0.03	0.02	malic enzyme, putative
**93**	4.52/50	[114]	0.47	0.13	0.27	0.18	0.17	0.17	0.14	0.04	0.09	Triosephosphate isomerase
**94**	5.72/101	[278]	0.05	0.09	0.17	0.08	0.09	0.20	0.20	0.14	0.15	Alpha-glucan phosphorylase 1
**95**	5.05/33	[108]	0.12	0.34	1.36	1.08	1.21	0.45	0.38	0.32	1.02	Actin-7
**96**	4.93/33	[77]	0.13	0.09	0.48	0.53	0.87	0.15	0.07	0.07	0.19	Actin-7
**97**	5.32/35	[165]	0.71	0.33	0.17	0.17	0.17	0.09	0.13	0.11	0.07	nitrile-specifier protein 5-like
**98**	5.53/28	[190]	0.09	0.07	0.15	0.14	0.22	0.10	0.19	0.09	0.27	Abscisic acid receptor PYR1
**99**	5.18/24	[210]	0.21	0.22	0.35	0.25	0.35	0.18	0.18	0.12	0.36	Triosephosphate isomerase
**100**	5.28/26	[161]	0.61	0.41	0.35	0.30	0.30	0.15	0.16	0.16	0.16	Dienelactone hydrolase
**101**	5.51/23	[67]	0.18	0.25	0.23	0.20	0.23	0.07	0.07	0.06	0.06	UMP-CMP kinase 3
**102**	5.31/25	[189]	0.37	0.38	0.07	0.11	0.00	0.05	0.06	0.05	0.00	Actin-7
**103**	5.50/22	[158]	0.41	0.29	0.25	0.22	0.28	0.15	0.13	0.18	0.17	Proteasome subunit type-3
**104**	5.62/18	[61]	0.37	0.38	0.35	0.46	0.78	0.41	0.50	0.43	0.56	Superoxide dismutase [Cu-Zn] 1
**105**	6.25/17	[142]	0.00	0.47	0.77	0.59	0.53	0.27	0.39	0.21	0.00	class I heat shock protein
**106**	6.36/16	[130]	0.31	0.19	0.23	0.18	0.13	0.11	0.12	0.10	0.00	Superoxide dismutase [Cu-Zn] 1
**107**	6.95/27	[257]	0.39	0.40	0.31	0.35	0.40	0.22	0.06	0.09	1.59	glutathione S-transferase GST
**108**	5.56/24	[166]	0.22	0.10	0.12	0.09	0.11	0.11	0.14	0.15	0.05	Triosephosphate isomerase
**109**	5.86/25	[77]	0.15	0.35	0.26	0.18	0.19	0.19	0.25	0.16	0.12	6-phosphogluconolactonase
**110**	6.25/26	[298]	0.43	0.42	0.37	0.23	0.23	0.40	0.36	0.42	0.13	Triosephosphate isomerase
**111**	6.87/37	[298]	0.24	0.54	1.10	0.14	0.99	0.58	0.26	0.53	0.29	annexin
**112**	6.23/41	[343]	0.14	0.69	0.76	0.39	0.58	0.93	0.78	0.95	0.30	alcohol dehydrogenase
**113**	5.63/41	[440]	0.00	0.08	0.20	0.17	0.16	0.44	0.26	0.47	0.15	Actin-7
**114**	6.65/38	[349]	1.05	0.72	0.68	0.37	0.33	0.66	0.31	0.76	0.23	Malate dehydrogenase
**115**	6.23/53	[399]	0.20	0.31	0.22	0.34	0.33	0.41	0.48	0.39	0.19	Leucine aminopeptidase 3
**116**	6.35/52	[173]	0.21	0.18	0.18	0.15	0.19	0.42	0.48	0.31	0.27	Bifunctional enolase 2
**117**	5.65/58	[345]	0.12	0.32	0.21	0.26	0.38	0.42	0.32	0.36	0.21	Betaine aldehyde dehydrogenase 1
**118**	5.84/57	[214]	0.12	0.24	0.19	0.32	0.33	0.32	0.21	0.13	0.13	Ketol-acid reductoisomerase

Note: [a]Assigned spot number (S1-S118) for the 118 differential proteins as indicated in Fig. 2.

[b]The experimental values for the mass (kDa) and *p*I of identified proteins.

[c]Mascot searched scores (M.S.) against cassava genome for MS and [MS/MS] searched results.

[d]The average abudance of the statistical percent of volue (ABV) value (Vol%) in 2-DE gels for protein spots in different development stages (S1-S9) of cassava roots produced by ImageMaster.

Supplementary Table S1-continued Detai information for the hierarchical cluster of abundance volume% of the protein spots in Figure 5A was presented in the following. The high- or low-abundance proteins were indicated in red or green, respectively.

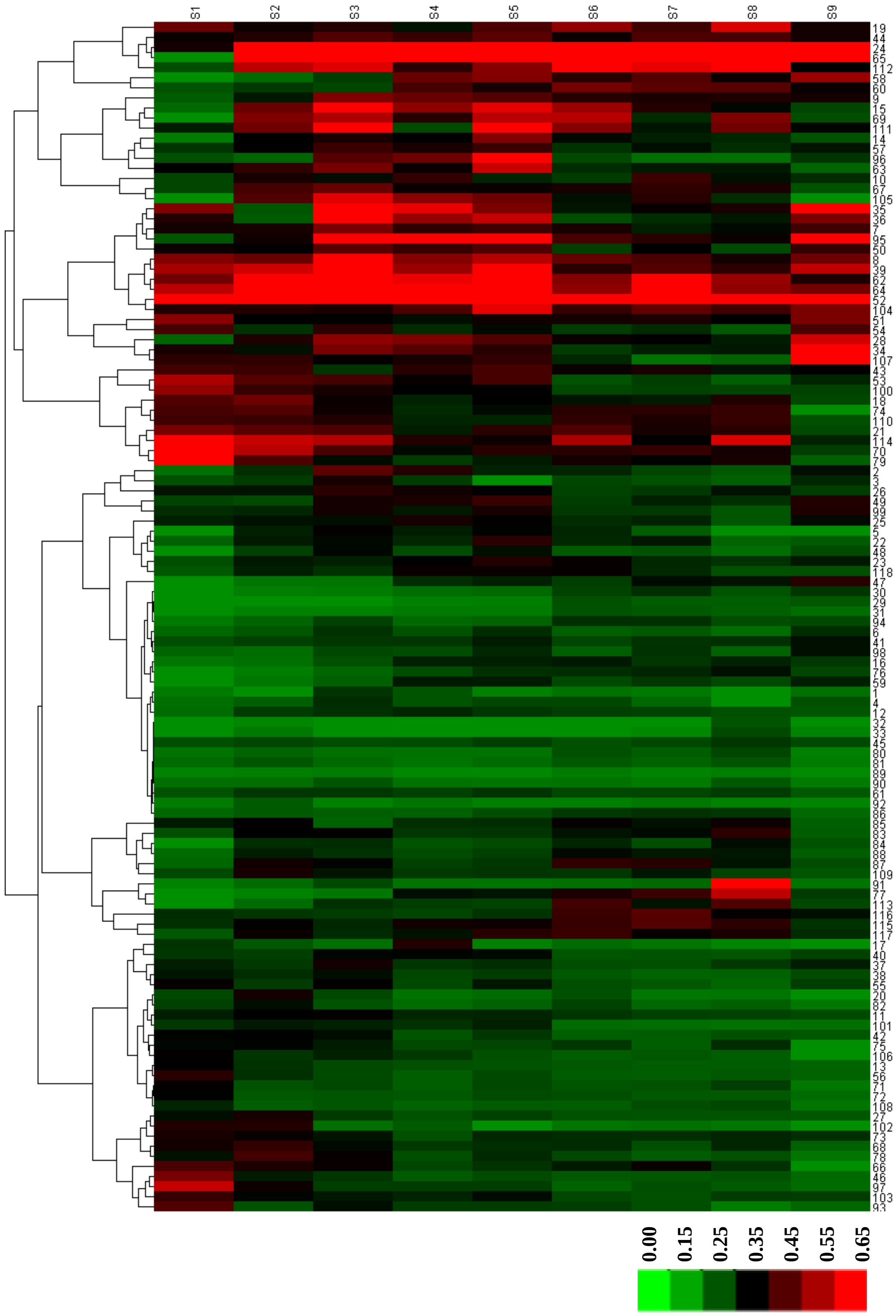

Supplementary Table S1-continued Detai information for the hierarchical cluster of the relative abundance volume ratios for the protein spots with S3 on 2-DE gels in Figure 5B was highlighted. The log values for the induced or reduced relative abundance volume ratios were indicated in red or green, respectively.

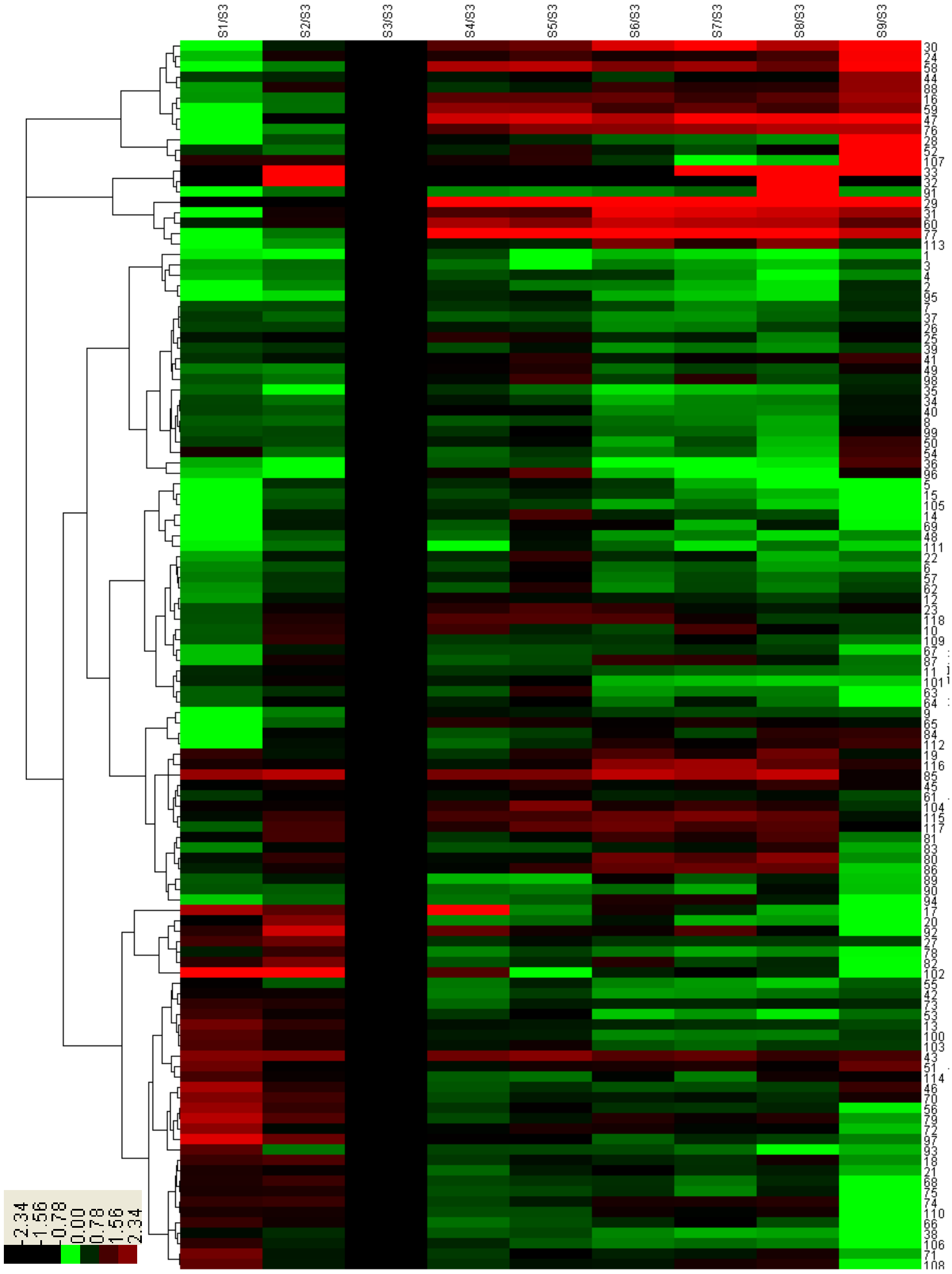

Supplementary Table S2. Information for differential proteins on 2-D DIGE gels and the corresponding MS identities.

Spot No.[a]	Exper.[b] *p*I/M*r*	Ther.[c] *p*I/M*r*	MP/ TP[d]	SC[e] (%)	M.S.[f] Score	Av. R.[g] S4/S3	Av. R. S5/S3	Av. R. S5/S4	Protein name
**2**	5.34/62.3	4.93/61	29/64	47	215	1.53	1.19	-1.12	Chaperonin 60 subunit alpha 1
**5**	4.95/48.3	5.15/51	15/65	51	101	2.42	3.63	1.55	Tubulin alpha-6 chain
**6**	4.99/47.7	5.05/47	29/93	59	154	-1.56	-1.26	-1.55	26S protease regulatory subunit 6A
**15**	4.95/48.3	5.18/51	18/79	48	103	12.27	18.41	1.55	Tubulin alpha-6 chain
**21**	6.27/47.5	5.80/42	14/54	33	101	-1.37	-1.54	-1.65	Glutamine synthetase
**23**	5.31/41.9	5.26/44	18/87	52	93	1.53	1.55	1.61	Actin-7
**27**	5.38/38.1	5.30/40	[2]	[7]	[113]	-1.21	-1.24	-1.58	Adenosine kinase 1
**28**	4.99/80.3	5.01/79	33/77	37	127	2.62	2.55	-1.53	Heat shock protein 90-3
**29**	8.50/81.9	5.54/96	24/95	24	81	1.73	1.63	-1.56	Alpha-glucan phosphorylase 1
**30**	8.5/81.9	5.60/96	[7]	[12]	[387]	1.75	1.59	-1.13	Alpha-glucan phosphorylase 1
**31**	6.71/80.6	5.60/96	16/58	32	76	1.56	1.02	-1.53	Alpha-glucan phosphorylase 1
**34**	4.77/29.4	4.62/29	16/46	44	115	-1.63	1.05	-1.57	14-3-3-like protein GF14 omega
**35**	4.76/29.5	4.68/28	21/56	54	159	-1.76	-1.11	-1.59	14-3-3-like protein GF14 omega
**36**	4.75/29.9	4.75/29	16/67	60	111	-1.36	-1.12	-1.56	14-3-3-like protein GF14 psi
**39**	5.17/32.8	5.21/34	19/77	55	151	1.30	1.18	-1.53	Probable lactoylglutathione lyase
**40**	5.17/32.8	5.24/34	[2]	[7]	[73]	1.30	1.08	-1.58	Probable lactoylglutathione lyase
**42**	5.29/35.5	5.28/36	[3]	[11]	[146]	-1.22	-1.10	-1.87	Probable fructokinase-1
**44**	5.41/38.8	5.45/38	[2]	[7]	[99]	1.28	1.22	1.54	NADP-dependent reductase
**46**	5.47/33.9	5.77/36	[3]	[15]	[254]	-1.54	-1.33	-1.56	NAD(P)H oxidoreductase
**47**	6.07/37.6	5.38/32	[2]	[8]	[133]	3.53	2.78	-1.25	Thiamine thiazole synthase
**49**	5.30/29.5	5.21/24	18/68	61	125	-1.55	-1.28	1.23	Ferritin-4, chloroplastic
**50**	7.79/26.7	5.28/23	[3]	[21]	[172]	1.23	1.02	-1.56	20 kDa chaperonin, chloroplastic
**51**	5.31/27.8	5.31/27	13/78	40	82	1.57	1.00	1.53	L-ascorbate peroxidase 1
**53**	5.31/41.9	5.71/28	[2]	[7]	[96]	1.58	-1.09	1.52	Actin-7
**57**	5.61/18.3	5.25/17	[2]	[16]	[133]	1.59	1.15	-1.63	regulator of ribonuclease
**58**	5.60/17.7	5.60/17	[2]	[18]	[66]	2.12	1.32	-1.55	Eukaryotic translation initiation factor
**61**	6.60/16.3	5.94/17	[2]	[11]	[112]	2.65	1.35	-1.92	Actin-depolymerizing factor 3
**64**	5.7/17.4	6.17/18	[10]	[22]	[180]	1.85	1.03	-1.63	Peroxiredoxin-2B
**65**	7.93/18.8	6.39/18	[5]	[25]	[164]	3.11	1.33	-1.56	18.1 kDa class I heat shock protein
**68**	5.73/17.2	6.84/17	12/37	72	119	-1.62	-1.54	-1.56	Ubiquitin-conjugating enzyme E2
**69**	6.14/18.0	7.03/16	[3]	[21]	[195]	-1.51	-1.09	-1.64	AT3g53990/F5K20_290
**71**	6.13/29.8	6.60/23	[2]	[8]	[99]	-2.58	-1.34	1.96	Proteasome subunit alpha type-2-A
**77**	7.10/36.9	6.22/32	17/70	38	88	7.84	4.62	-2.23	Thiamine thiazole synthase
**78**	5.68/43.7	6.10/48	16/54	55	119	-2.18	-2.11	1.06	S-adenosylmethionine synthase 1
**79**	6.09/36.2	6.64/39	18/67	54	119	1.62	-1.02	1.58	malate dehydrogenase
**86**	6.03/65.3	6.45/64	35/90	47	200	1.51	1.54	1.03	malic enzyme, putative
**88**	5.86/47.9	6.41/52	25/78	62	160	1.43	1.14	-1.67	Bifunctional enolase 2
**89**	5.29/69	5.45/69	41/95	61	234	1.78	1.08	1.23	V-type proton ATPase subunit A
**92**	6.03/65.3	6.61/64	28/71	37	174	1.52	1.53	1.12	malic enzyme, putative

**94**	8.50/81.9	5.72/10<br>1	[7]	[12]	[278]	1.62	1.18	-1.54	Alpha-glucan phosphorylase 1
**97**	5.49/35.8	5.32/35	[7]	[24]	[165]	1.51	-1.10	-1.59	nitrile-specifier protein 5-like
**105**	7.93/18.8	6.25/17	[5]	[23]	[142]	1.51	-1.21	-1.80	18.1 kDa class I heat shock protein
**113**	5.31/41.9	5.63/41	[9]	[36]	[440]	1.56	1.17	1.51	Actin-7
**118**	5.50/58.5	5.84/57	[4]	[9]	[214]	1.53	1.20	-1.58	Ketol-acid reductoisomerase
**130**	5.86/95.0	6.71/83	[7]	[10]	[184]	1.59	1.14	-1.66	At1g56070/T6H22_13
**131**	4.99/80.2	5.12/85	[4]	[7]	[181]	1.69	1.64	1.56	Heat shock protein 90-3
**132**	5.20/75.3	4.46/73	[9]	[16]	[516]	1.70	1.19	-1.61	Heat shock 70 kDa protein 7
**133**	5.24/75.5	4.48/73	[7]	[12]	[386]	1.59	1.21	-1.59	Heat shock 70 kDa protein 7
**134**	4.99/59.3	4.85/72	[6]	[14]	[305]	1.56	1.15	-1.55	Putative S9 Tyrosyl aminopeptidase
**135**	6.31/80.9	6.05/68	[2]	[7]	[78]	1.62	1.04	1.53	Transketolase-2, chloroplastic
**136**	4.91/54.5	4.78/52	[2]	[8]	[78]	-1.54	-1.12	-1.61	V-type proton ATPase subunit B3
**137**	4.77/50.7	5.05/47	[5]	[11]	[193]	-1.53	-1.04	-1.66	Tubulin beta-6 chain
**138**	5.30/59.7	5.56/63	[4]	[13]	[195]	1.52	1.20	0.92	Importin subunit alpha-1
**139**	4.68/50.7	4.78/51	[4]	[10]	[170]	2.98	3.42	1.15	Tubulin beta-6 chain
**140**	5.86/53.9	6.35/52	[4]	[10]	[65]	-1.51	-1.64	1.02	Adenosylhomocysteinase 1
**141**	5.70/51.9	6.34/53	[3]	[7]	[153]	1.57	1.18	0.86	ribulose carboxylase-oxygenase
**142**	6.19/48.5	6.65/44	[4]	[10]	[94]	-1.63	-1.09	0.89	Aldolase-type family protein
**143**	6.02/38.3	6.45/45	[5]	[20]	[152]	-1.53	-1.64	1.01	Alcohol dehydrogenase class-P
**144**	5.50/26.4	5.35/45	[2]	[6]	[61]	1.54	1.15	0.93	acetylornithine deacetylase-like
**145**	5.64/42.2	5.11/37	[2]	[6]	[115]	-1.65	-1.79	1.29	Actin-7
**146**	5.82/41.6	5.71/41	[2]	[8]	[73]	-1.65	-1.02	-1.53	UDP-arabinopyranose mutase 3
**147**	8.26/50.4	4.89/41	[4]	[9]	[103]	1.67	1.22	1.51	Phosphoglycerate kinase 1
**148**	8.63/43.1	6.68/40	[7]	[20]	[380]	1.61	1.06	1.52	fructose-bisphosphate aldolase 3
**149**	8.93/24.6	6.76/38	[4]	[15]	[118]	-4.15	-2.33	1.81	fructose-bisphosphate aldolase
**150**	7.72/15.2	6.36/39	[2]	[10]	[69]	1.70	-1.10	-1.66	aldo/keto reductase AKR
**151**	5.12/35.6	5.11/34	[4]	[16]	[110]	1.58	1.05	1.62	Probable fructokinase-1
**152**	4.75/34.9	4.63/33	[3]	[14]	[125]	1.92	1.16	-1.62	late embryogenesis abundant proteins
**153**	5.31/41.9	5.28/25	[3]	[7]	[62]	1.54	1.06	-1.61	Actin-7
**154**	4.93/30.9	4.88/28	[7]	[37]	[343]	-1.57	-1.17	-1.64	26S proteasome non-ATPase
**155**	5.77/27.7	6.32/24	[2]	[10]	[107]	-1.61	-1.14	-1.57	Triosephosphate isomerase
**156**	4.70/26.2	4.56/27	[3]	[10]	[123]	-1.51	-1.15	-1.75	Proteasome subunit alpha type-5
**157**	6.42/24.9	6.16/23	[2]	[8]	[61]	-1.51	-1.07	-1.68	Proteasome subunit beta type-1
**158**	6.52/26.9	5.56/30	[4]	[26]	[292]	-5.22	-2.39	2.23	3-isopropylmalate dehydratase
**159**	6.17/23.8	6.88/23	[4]	[24]	[192]	-2.57	-2.64	1.03	glutathione-s-transferase theta
**160**	7.66/29.6	4.68/25	[2]	[9]	[79]	-1.56	-1.13	-1.65	peroxiredoxin
**161**	5.98/26.2	5.21/22	[2]	[13]	[173]	-1.52	-1.20	-1.65	conserved hypothetical protein
**162**	4.95/12.4	4.75/15	[2]	[22]	[88]	-1.53	-1.02	1.51	Small ubiquitin-related modifier
**163**	4.92/32.1	4.98/15	8	31	78	-5.57	-1.36	4.17	actin
**164**	6.74/17.3	6.76/12	[5]	[26]	[88]	-1.62	-1.54	1.14	Ubiquitin-conjugating enzyme E2 35

Note:

[a]Assigned spot number (S2-S164) for the 80 differential proteins in 2-D DIGE gelss of cassava

root proteins as indicated in Fig. 3.

[b, c]The experimental (b) and theoretical (c) values for the mass (kDa) and *p*I of identified proteins. The theoretical values were retrieved from the protein database, and the experimental values were produced by ImageMaster.

[d]The number of matched peptides (MP) and total searched peptides (TP) for the proteins identified for MS. The number of matched peptides for MS/MS was presented in square bracket.

[e]Sequence coverage (SC) of the identified proteins by MS and [MS/MS].

[f]Mascot searched scores (M.S.) against cassava genome for MS and [MS/MS] searched results.

[g]The average changed ratios of the target protein spots in 2-D DIGE gels of the stage 3 (S3), 4 (S4) and 5 (S5) of cassava roots.

Supplementary Table S2-continued Detai information for the hierarchical cluster of the average changed abundance ratios of the identified protein spots on 2-D DIGE gels in Figure 5C was also highlighted. The log values for the induced or reduced relative abundance volume ratios were indicated in red or green, respectively.

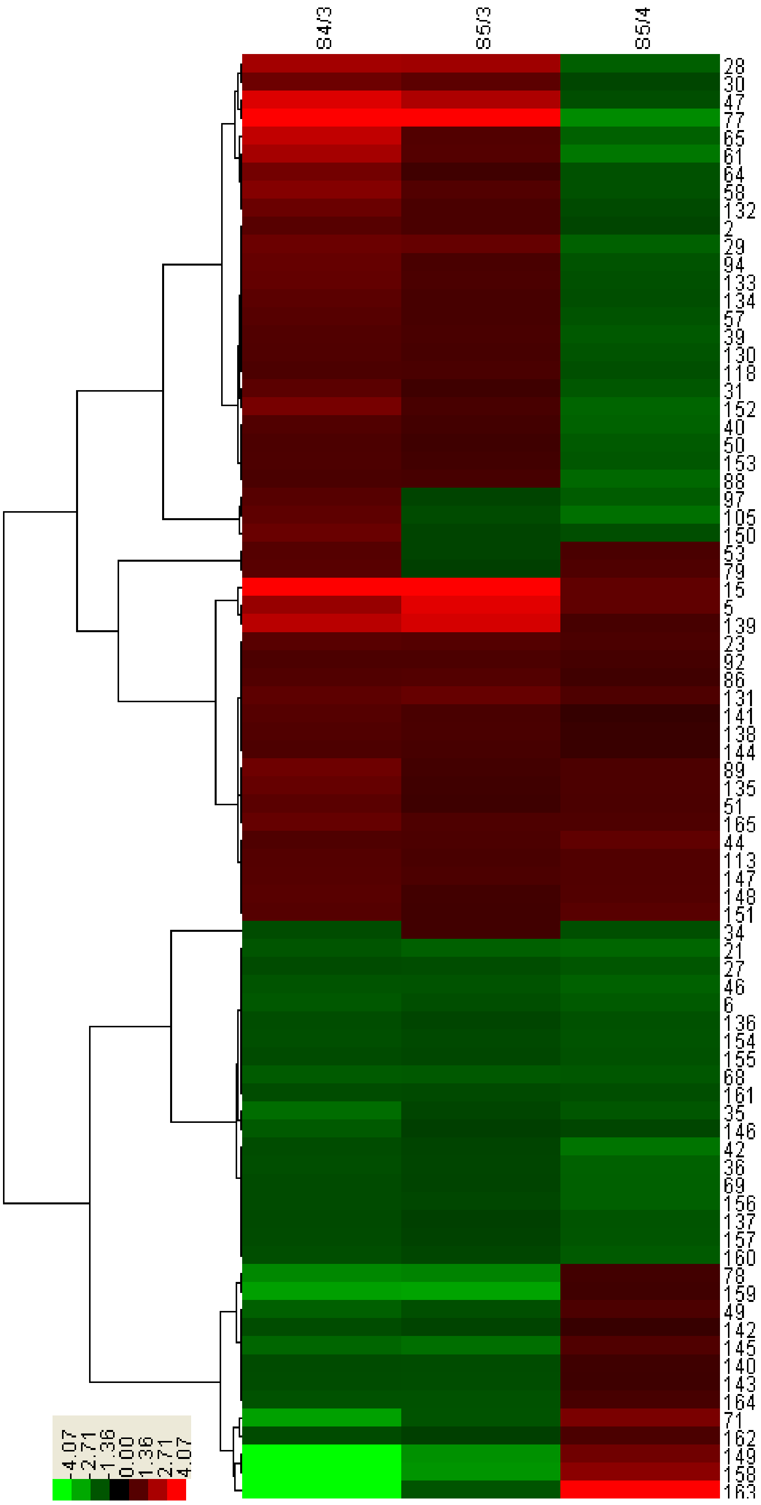

Supplementary Table S3. Primers used for RT and Q-PCR analysis.

14-3-3 protein (Spots 34, 35, 36):

5’- GAGCAAGATTTGTGATGGGATC-3’

5’-TAGGAGCGAGATCAGCAAGG-3’

Ascorbate peroxidase APX2 (Spots 51, 52, 73):

5’-GTTTCATCGCTGAGAAAGGC-3’

5’-ATCTCAACGGCAACAACACC-3’

Superoxide dismutase (Spots 66, 72, 104, 106):

5’-GGCTGAAGCTGTTCTTACCA-3’

5’-TGATCTTTGCCAGAAGGGTT-3’

Initiation factor eIF5-A:

5’-GATATTGTTCCTTCATCCCACA-3’

5’-CCTCAGCAAACCCATCTTTA-3’

S-adenosylmethionine synthetase 4:

5’-AAGACGCAGGTTTAGGTGGAAGAT-3’

5’-GATGGGTTTAGGTGGAAGAT-3’

Malate dehydrogenase (Spots 79, 114):

5’-CAAATGCGTTGATTCTAAGGG-3’

5’-GCGTGATTGACATGTGGGTAC-3’

UDP-D-apiose/UDP-D-xylose synthase:

5’-TCTGGCGGCTATTTGTACTC-3’

5’-TCTCTGCTTCTCAATGGGTC-3’

Enolase (Spots 83, 87, 88, 116):

5’- TGCTGGAAATAAACTTGCTATG -3’

5’-AACCACCTTCATCACCAACAT-3’

ADP-glucose pyrophosphorylase small subunit (Spots 20, 91):

5’- TTCATTCTTGTAGCCACCCA-3’

5’- ATTATTCTTGGAGGCGGTGC-3’

Starch phosphorylase 1 precursor:

5’- GAGGTTGCTGAAGATTGGCT-3’

5’- CCCAGCATTGAAAGCAGACA-3’

18sRNA: 5’- CCTGAGAAACGGCTACCAT -3’

5’- CGTGTCAGGATTGGGTAAT -3’

Primers specific for the vector:

S1300F, 5’-GCCTTGCTTCCTATTATATCTTCC-3’;

S1300R, CCCATCTCATAAATAACGTCATG.

Primers specific for 14-3-3 gene in the transgenic plants for Southern blotting:

P1300-1433F, 5’- TGCTGATCTCGCTCCTACCCAC -3’;

P1300-1433R, 5’- AACCACCAGATGATGGCATCTACTC -3’.
